# Analysis of Time Series Gene Expression and DNA Methylation Reveals the Molecular Features of Myocardial Infarction Progression

**DOI:** 10.3389/fcvm.2022.912454

**Published:** 2022-06-24

**Authors:** Yuru Han, Baoyu Duan, Jing Wu, Yanjun Zheng, Yinchen Gu, Xiaomeng Cai, Changlian Lu, Xubo Wu, Yanfei Li, Xuefeng Gu

**Affiliations:** ^1^Shanghai Key Laboratory of Molecular Imaging, Zhoupu Hospital, Shanghai University of Medicine and Health Sciences, Shanghai, China; ^2^School of Medical Instrument and Food Engineering, University of Shanghai for Science and Technology, Shanghai, China; ^3^School of Nursing, Shanghai University of Traditional Chinese Medicine, Shanghai, China; ^4^School of Basic Medical Sciences, Fudan University, Shanghai, China; ^5^Seventh People's Hospital of Shanghai University of Traditional Chinese Medicine, Shanghai, China; ^6^School of Pharmacy, Shanghai University of Medicine & Health Sciences, Shanghai, China

**Keywords:** myocardial infarction, time series, MeDIP-seq, RNA-seq, DNA methylation

## Abstract

Myocardial infarction (MI) is one of the deadliest diseases in the world, and the changes at the molecular level after MI and the DNA methylation features are not clear. Understanding the molecular characteristics of the early stages of MI is of significance for the treatment of the disease. In this study, RNA-seq and MeDIP-seq were performed on heart tissue from mouse models at multiple time points (0 h, 10 min, 1, 6, 24, and 72 h) to explore genetic and epigenetic features that influence MI progression. Analysis based on a single point in time, the number of differentially expressed genes (DEGs) and differentially methylated regions (DMRs) increased with the time of myocardial infarction, using 0 h as a control group. Moreover, within 10 min of MI onset, the cells are mainly in immune response, and as the duration of MI increases, apoptosis begins to occur. Analysis based on time series data, the expression of 1012 genes was specifically downregulated, and these genes were associated with energy metabolism. The expression of 5806 genes was specifically upregulated, and these genes were associated with immune regulation, inflammation and apoptosis. Fourteen transcription factors were identified in the genes involved in apoptosis and inflammation, which may be potential drug targets. Analysis based on MeDIP-seq combined with RNA-seq methodology, focused on methylation at the promoter region. GO revealed that the downregulated genes with hypermethylation at 72 h were enriched in biological processes such as cardiac muscle contraction. In addition, the upregulated genes with hypomethylation at 72 h were enriched in biological processes, such as cell-cell adhesion, regulation of the apoptotic signaling pathway and regulation of angiogenesis. Among these genes, the Tnni3 gene was also present in the downregulated model. Hypermethylation of Tnni3 at 72 h after MI may be an important cause of exacerbation of MI.

## Introduction

Myocardial infarction (MI) is a common cardiovascular disease worldwide. MI is caused mainly by the rupture of an atherosclerotic plaque leading to thrombus formation within the lumen of a coronary vessel, which in turn blocks blood flow to the distal myocardium (1), and the heart muscle is damaged by a lack of oxygen (2). Infarction leads to cardiac myocyte death and subsequent necrosis of the tissue in the infarcted area, causing inflammatory cells that phagocytose dead cells and debris within the infarcted area (3). The symptoms of MI are chest pain, which travels to the left arm or left side of the neck, shortness of breath, sweating, nausea, vomiting, abnormal heartbeat, anxiety, fatigue, and other phenomena (4). More than 7 million people worldwide experience a heart attack each year, and although the death rate from heart attacks has declined in recent years, it is still in the 10% range (5–7). MI is characterized by insignificant early symptoms, sudden onset and high mortality, and it is difficult to prevent, diagnose and treat this disease. Therefore, ideal biomarkers capable of rapid diagnosis and effective regulation are still needed in clinical practice.

MI occurs when myocardial cells are irreversibly damaged and necrotic due to hypoxia and decreased ATP supply (8). Oxidative stress, the inflammatory response and MI pathological processes have been confirmed by numerous studies to be closely related (9, 10). (ROS) generation activates the p53 and NF-κB signaling pathways, leading to upregulation of proinflammatory cytokine expression and impairment of glucose metabolism (11). Under pressure load, the expression of p53 increases, and the expression of ICAM1 increases, which lead to the inflammatory response of the heart, myocardial injury and systolic dysfunction. Moreover, in the inflammatory response after MI, IL-17A induces myocardial cell apoptosis mainly through the p38-p53-Bax signaling pathway, thus promoting ventricular remodeling and leading to heart failure (12).

With the development of sequencing technologies, transcriptomic changes in myocardial tissue have been studied through animal models at different times after MI (13, 14). However, single layer-omics data have limited ability to reveal complex molecular interactions between different layers. DNA methylation is a widely studied epigenetic modification that plays an essential role in regulating gene expression. A study by Agha et al. found that DNA methylation in blood was associated with coronary heart disease and MI (15). The experiment by Yousuf et al. (16) demonstrated that the hypermethylated state of the ABO gene promoter was associated with MI. Currently, only a few studies have focused on the relationship between DNA methylation and MI, and little is known about the modification and function of DNA methylation in myocardial tissue in early MI.

In this study, RNA sequencing (RNA-Seq) and Methylated DNA immunoprecipitation sequencing (MeDIP-Seq) were used to determine transcriptome profiles and DNA methylation of myocardial cells from mice at six time points after MI. We intend to study the characteristics of gene expression at different time points after MI and the genes with specific expression trends at different time points. First, transcriptome data at 0 h served as the control group, and differentially expressed genes (DEGs) at 10 min, 1, 6, 24, and 72 h were obtained. Subsequently, 0, 1, and 72 h expression data were selected for Short Time-series Expression Miner (STEM) analysis, and time-dependent gene expression profiles with statistical significance were screened, and transcription factors in the module were found. The TRRUST database was used to predict the target genes of transcription factors. The DrugBank database was used to identify drug targets among transcription factors. The methylation data were combined with the transcriptome data and the methylation of gene promoters at each time point was analyzed. The results can help us better understand the occurrence and development of disease after MI, and will provide new insights into the mechanism of MI at the epigenetic level.

## Materials and Methods

### Animal

Eighteen 12-week-old male C57BL/6JR mice were included in the experiment. The experiment was divided into 6 groups. Each group contained 3 mice, 5 groups of which required surgery to make models, called the operation group, and the other group was the sham operation group (0 h). The operation group was divided into 10 min, 1, 6, 24, and 72 h subgroups after MI. The mice were anesthetized with sodium pentobarbital (50 mg/kg, intraperitoneally) and placed on the operating table. The mice in the surgery group were intubated, and the left anterior descending coronary artery was ligated to induce MI, separately. The mice in the control group received the same operation, sutured through the inferior artery, but not ligated (17). Complete anesthesia of mice with 1–3% isoflurane after specific time of MI, followed by euthanasia by cervical dislocation. Total RNA was isolated from myocardial region of infarcted left ventricle.

### The Acquisition of RNA-Seq and MeDIP-Seq Data

First, total RNA was extracted from the samples and the quality of the RNA was tested. Then, mRNA was purified from the total RNA using oligo dT magnetic beads. Fragmentation of mRNA was performed under high temperature conditions using divalent cations in NEBNext First Strand Synthesis Reaction Buffer (5X). Synthesis of first-strand cDNA using random hexamer primer and M-MuLV Reverse Transcriptase (RNase H) as the template for mRNA. Second strand cDNA synthesis was carried out with DNA polymerase I and RNase H. The remaining overhangs were transformed to blunt-ended by exonuclease/polymerase activity. The 3′ end of the DNA fragment was adenylated and then the NEBNext adapters with a hairpin loop structure were attached in preparation for hybridization. Library fragments were purified using the Ampoule XP system (Beverly Beckman Coulter, USA) to prioritize cDNA fragments of 250–300 bp in length. The cDNA was ligated using 3 μl of USER Enzyme (NEB, USA) for 15 min at 37°C using an adapter of the selected size, then used for 5 min at 95°C, followed by PCR. PCR was then conducted using Phusion High Fidelity DNA polymerase, universal PCR primers and index (X) Primer. Next, the PCR products were purified (Ampoule XP system) and the library quality was evaluated on the Agilent Bioanalyzer 2100 system. Finally, the library preparation was sequenced on the Illumina Novaseq platform and 150 bp paired-end reads were generated.

Genome-wide DNA methylation sequencing was performed using MeDIP-Seq, which was carried out by Shanghai Bohol Biotech, China. To summarize, the genomic DNA was sheared to a peak of 250bp fragments using the Covaris S2 ultrasound system. Next, the end repair and adenylation of the 3' end processes were carried out. After the connector ligation reaction, fragment selection was performed using beads. Then, immunoprecipitation of the methylated DNA was performed and the eluted DNA was amplified by PCR to generate the final sequencing library. The Agilent Bioanalyzer 2100 was used to purify the PCR products and assess the library quality. Finally, the illumina Novoseq 6000 was used to perform paired-end sequencing.

### RNA-Seq Data Analysis

The cutadapt (V1.18) (18) was used to remove adapter sequences, low quality bases, and read parameters of <50 bases “-a AGATCGGAAGAGC-a AGATCGGAAGC-trim-n-m 50-q 20,20.” The clean data were mapped to the mm10 reference genome using Hisat2 (v2.1.0) (19) to obtain BAM files. The featureCounts software (20) was used to obtain the number of reads of genes from the BAM files in each sample and the fragments per kilobase million (FPKM) values in genes was calculated by R software. The annotated information of lncRNA was downloaded from GENECODE (https://www.gencodegenes.org/), lncRNAs were removed from the obtained expression data, and the remaining mRNAs were used for subsequent analysis. The differentially expressed genes between each sample group (10 min, 1, 6, 24, and 72 h) and the control group(0h) were calculated using the DESeq2 package in R software (version 4.1.1). The threshold was set as a *p*-value < 0.05 and a | log_2_FC| >1.

### Gene Set Enrichment Analysis

To identify the biological signaling pathways involved in gene expression at different time points, normalized gene expression data obtained from five different time groups were used to perform GSEA analysis against controls for MI using the Gene Set Enrichment Analysis (GSEA) tool (v4.1) (21, 22), respectively. A subset of C2 (Mm.c2.cp.kegg.v7.1.entrez.rds) from mice was downloaded from the database (https://bioinf.wehi.edu.au/software/MSigDB/) as the reference gene set for GSEA analysis. In each analysis, 1000 permutations of the genome were performed to identify significantly different pathways. The normalized enrichment score (NES) indicates the extent to which the genome is over-represented at the top or bottom of the gene ranking list in the expression dataset. The *p*-value < 0.05 was set as the cut-off criteria. The GSEA analysis results were visualized using the ggbarplot function in ggpubr package.

### Trend Analysis and Function Annotation

To obtain the obvious variation trend in gene expression, the gene expression data at 0, 1, and 72 h were analyzed using Short Time-series Expression Miner (STEM) software (23). The maximum number of model profiles was set to 8, and the maximum unit change in the model profiles between time points was set to 1. Gene expression values were transformed to log ratios relative to the expression value at 0 h. The parameter maximum–minimum was set as 1. If the maximum change in the converted expression value between the three time points was <1, the gene was filtered out. The algorithm assigned each gene to the best matched expression profile model on the basis of the correlation coefficient. The boxes in the figures were colored if the profiles were statistically significant and boxes of the same color with high similarity could be analyzed together.

Gene Ontology (GO) and kyoto encyclopedia of genes and genomes (KEGG) pathway analyses of significant profiles were performed using the clusterProfiler package in R software. A *p*-value < 0.05 was considered statistically significant.

### Mining Transcription Factors, Drug Target Prediction

In this section, we focused on the genes from profile #4 and profile #7 enriched in apoptosis and inflammation related pathways. The transcription factor database (TRRUST, https://www.grnpedia.org/trrust/) contains 6,552 pieces of reported regulatory information on TF-targets in humans and mice were used to identify transcription factors in these genes. Drug target prediction using the DrugBank database (https://go.drugbank.com/targets).

### MeDIP-Seq Data Analysis

The reads with low overall quality, containing sequencing primers, and with low end quality were filtered using FASTX (http://hannonlab.cshl.edu/fastx_toolkit/index.html). The reads from each MeDIP sample were mapped to the mouse (mm10) genome using Bowtie2 (24) and default parameters to obtain BAM files. We apply MACS2 (25) to the bam file obtained in the above step to find the peak enrichment region and obtain the enrichment region of the sample peak. Then, annotation of the peaks was performed. The distribution of the peaks on the chromosomes (promoter, 5'UTR, CDS, 3'UTR, Intron and TTR, where promoter is 2000 bp upstream of the transcription start site and TTR is 5000 bp downstream of the transcription termination site) was obtained according to the information on the location of the peaks. On CpG Island, the CpG shore is located 2000 bp downstream of CpG Island and the CpG shelf is located within 2000 bp to 4000 bp downstream of CpG Island. Differential analysis was performed on the peaks of the control and other MI groups using the R package MEDIPS (26) with parameters set to |log_2_FC| >1 and *p*-value < 0.05. Differentially methylated regions (DMRs) were annotated using the same procedure as for peak annotation above.

### Comprehensive Analysis of Methylation and Transcriptome Data

MeDIP-Seq and RNA-Seq data were interpreted in an integrated way to identify epigenetically regulated genes that affect the progression of MI. We calculated the intersection of DMRs and DEGs as differentially methylated and expressed genes (DMEGs), and classified them into four distinct groups: HypoUp, HypoDown, HyperUp, and HyperDown. A fold change > 2 and *p*-value < 0.05 were used as filtering criteria. We focused on DMEGs in the promoter region. Due to the large number of DMEGs in the 72 h promoter region, we focused on genes with downregulated hypermethylation and upregulated hypomethylation, and conducted GO enrichment analysis by using the clusterProfiler package in R software.

## Results

### Transcriptome Changes of DEGs

**Figure 1:**
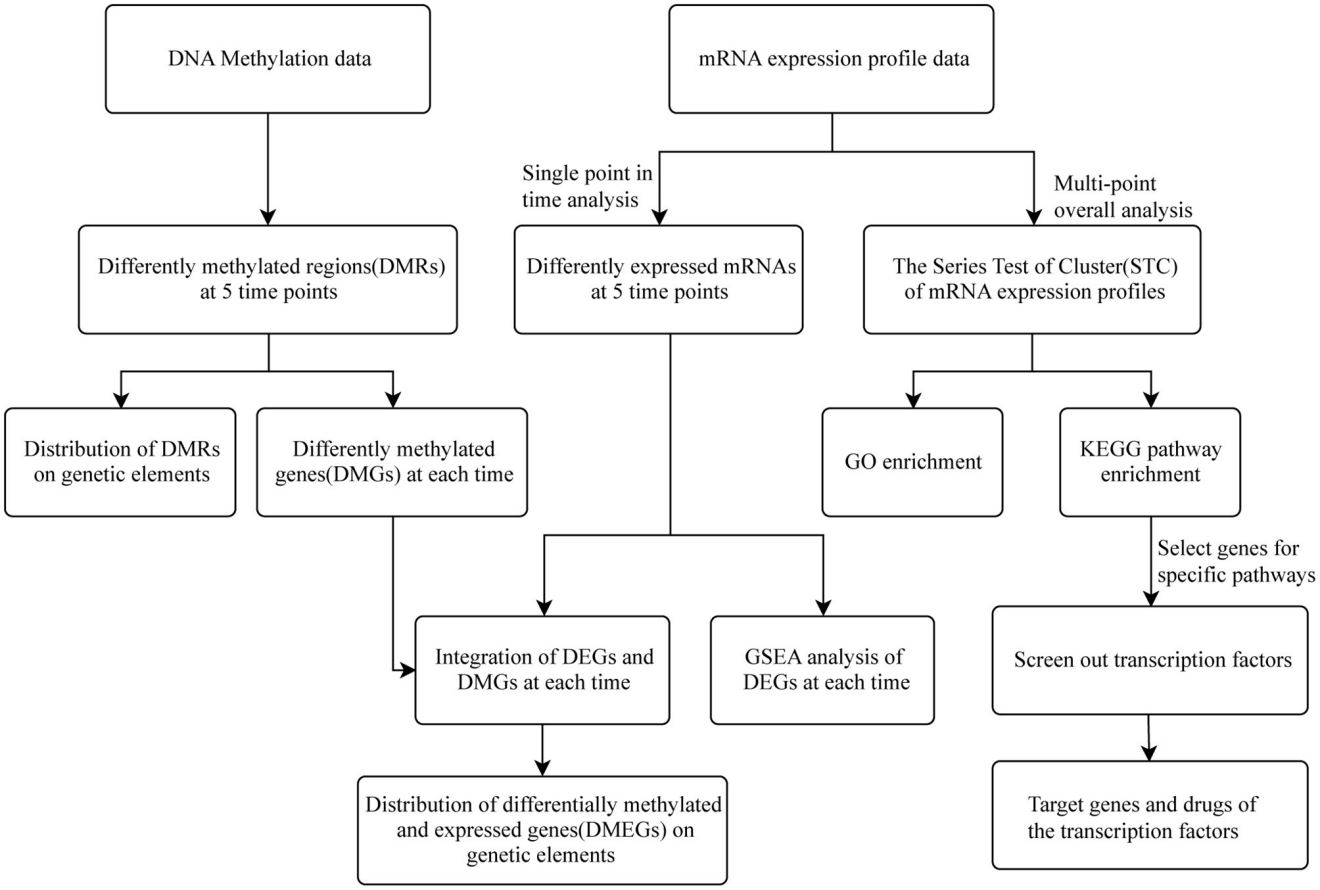
Flow chart of this research.

**Figure 2:**
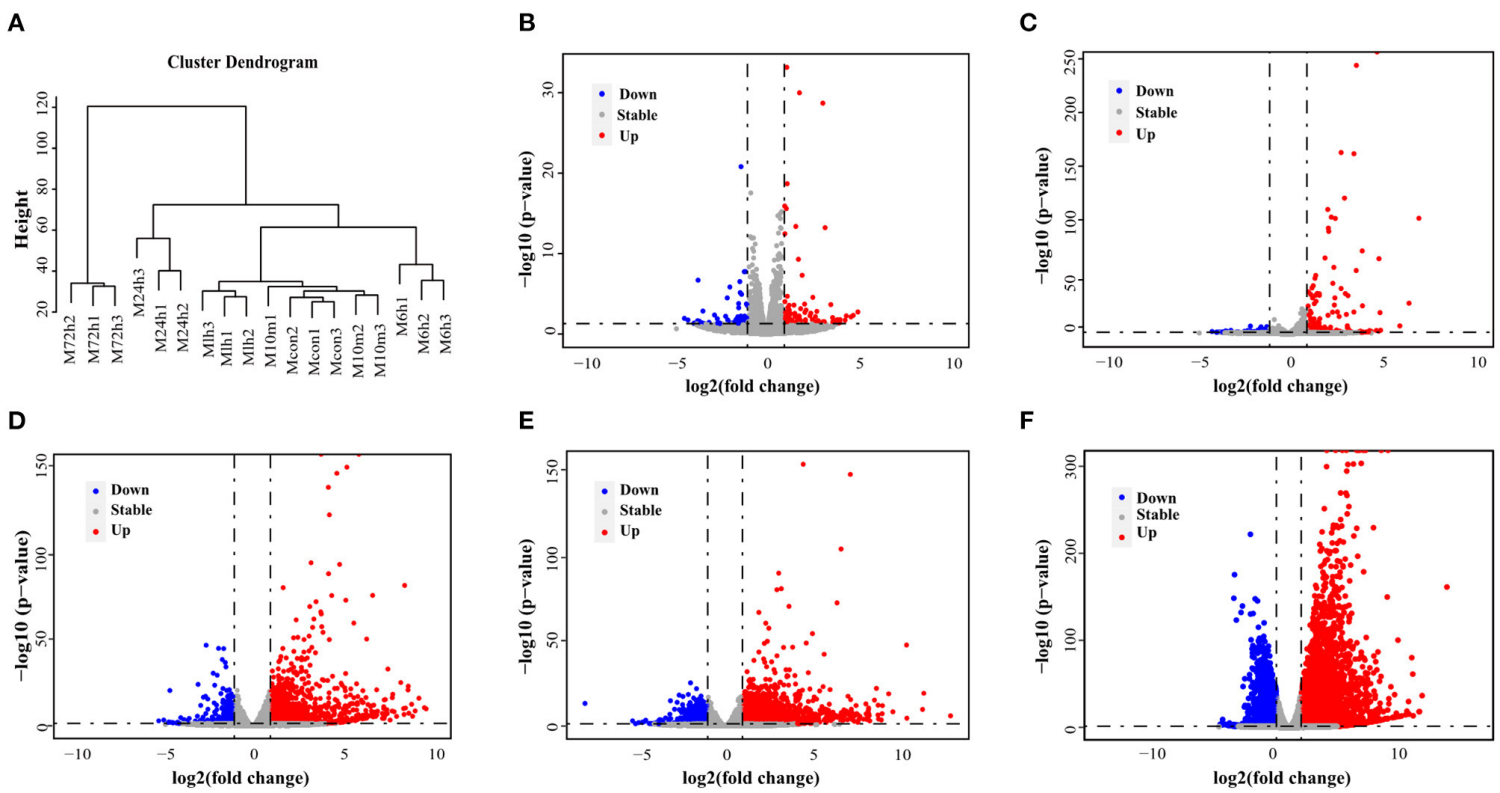
DEGs in mouse model of MI derived by RNA-Seq. **(A)** An unsupervised cluster analysis of MI at 6 different time points. **(B–F)** Volcano plot showing DEGs in 10 min, 1, 6, 24, and 72 h post-MI. Red dots indicate significantly upregulated mRNAs, and blue dots indicate downregulated mRNAs.

Figure 1 shows the flow diagram for study enrolment. Unsupervised hierarchical clustering of the cardiac transcriptome indicated that mRNA expression signature distinguished 6 different time points of MI (Figure 2A). DESeq2 packages were used to identify differentially expressed genes at each MI time point compared with the control group (0 h). There were 151, 203, 1570, 2174 and 5513 DEGs (|log_2_FC | > 1, *p*-value < 0.05) in the 10 m vs. 0 h, 1 vs. 0 h, 6 vs. 0 h, 24 vs. 0 h and 72 vs. 0 h (Figures 2B–F). The upregulation and downregulation of DEGs at each time point are shown in Supplementary Figure 1.

### GSEA Identifies Key Pathways in the Progression of MI

**Figure 3:**
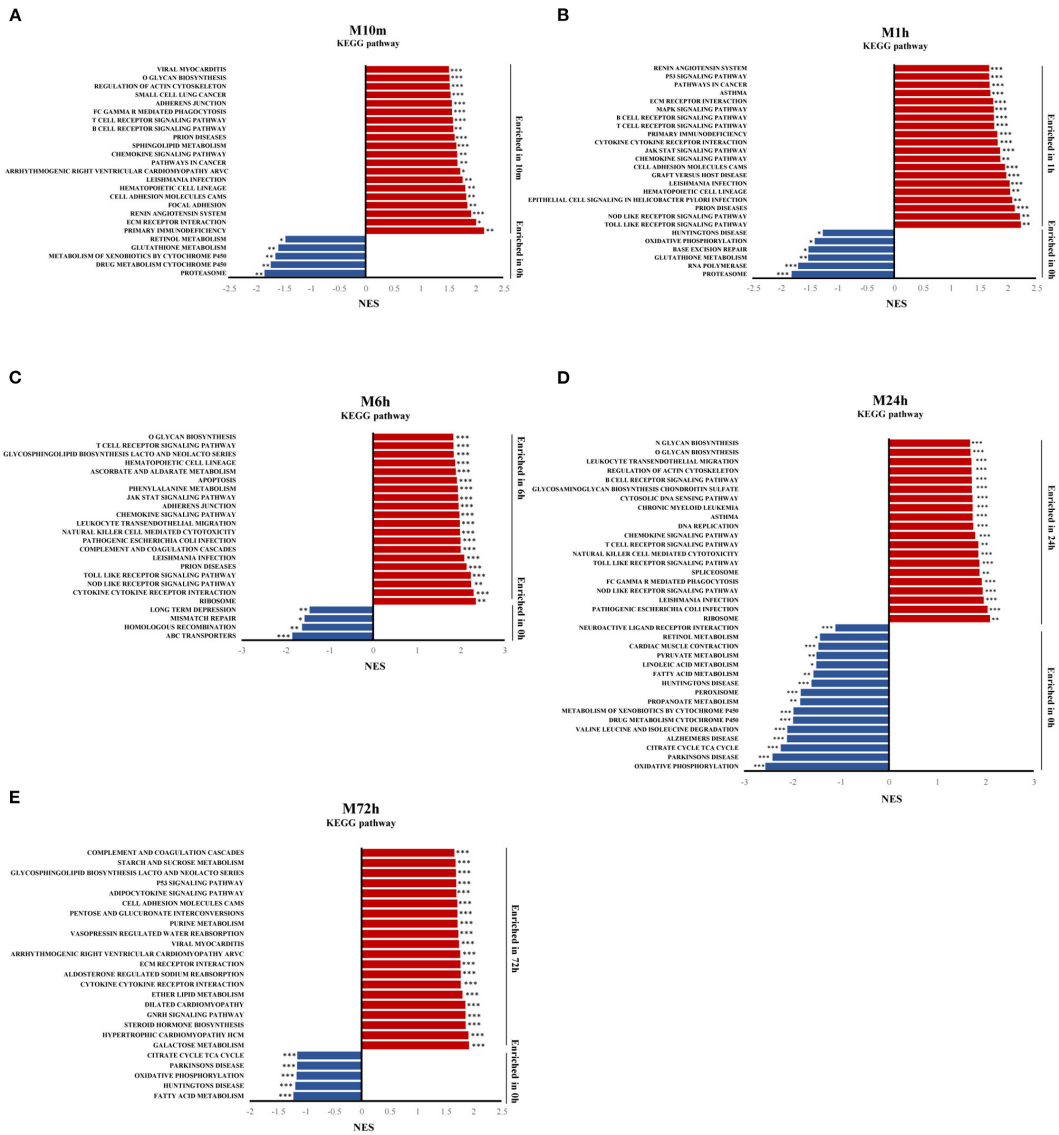
Gene expression analysis by GSEA. **(A–E)** Gene set enrichment analysis (GSEA) of KEGG gene sets from the Molecular Signatures Database (MSigDB), showing the most significantly enriched gene sets in MI, and their normalized enrichment scores. *p-value < 0.05, **p-value < 0.01, ***p-value < 0.001.

To identify the signaling pathways involved in the progression of MI (10 min, 1, 6, 24, and 72 h) over the control group, GSEA was performed on the whole set of expressed genes and ranked by their differential expression at each MI time point vs. the control group. Figures 3A–E displays the results of GSEA for each time point with KEGG gene sets from MSigDB using the GSEA tool. The results show that inflammatory and immune signaling pathways (e.g., B cell receptor signaling pathway, T cell receptor signaling pathway, chemokine signaling pathway) were among the top overrepresented pathways in the 10 min post MI and were continuously activated until 24 h post MI. Apoptosis signaling, such as the p53 signaling pathway, ECM receptor interaction, and apoptosis, was also significantly activated at different time points after MI. Oxidative phosphorylation in downregulated gene-enriched pathways lasted from 10 min after MI to 72 h.

### Trend Analysis of mRNA and Function Annotation

**Figure 4:**
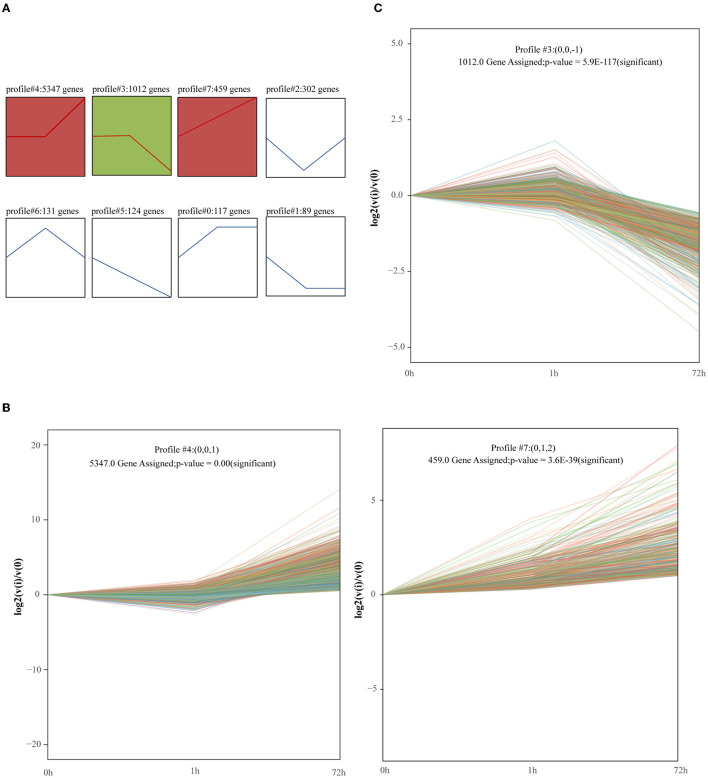
Short Time-Series Expression Miner (STEM) analysis of gene expression profile. Squares of the same color indicate that the correlation is >0.7. **(A)** Trend analysis of genes. Gene expression was normalized in the STEM software and genes with maximal values minus minimal values not <1 were included in the analysis. The 7581 genes from 0, 1, and 72 h after MI were clustered into 8 profiles. **(B,C)** Significantly enriched trend analysis of profile#3, profile#4, and profile#7.

7581 genes were considered by STEM to have undergone expression changes between 0, 1, and 72 h. These genes were clustered into 8 profiles (Figure 4A). We identified 3 significant clusters, and the profile boxes display the gene expression patterns over three time points. The expression of mRNAs in profile #4 remained temporally stable from 0 to 1 h but rapidly increased at 72 h (Figure 4B). Moreover, 459 genes in profile #7 continued to increase from 0 to 72 h (Figure 4B). Profile #4 and profile #7 were in a highly similar, which indicates that the upregulation of the gene expression in the two modules over time has an impact on the development of the disease. The expression of mRNAs in profile #3 remained temporally stable from 0 to 1 h but rapidly declined at 72 h (Figure 4C). This group of mRNAs may be involved in the process of myocardial contraction, because with the development of MI, myocardial contractility decreases.

Finally, two expression patterns were identified: type I profiles (profiles #4 and #7) with upregulation modes, and type II profile (profile #3) with downregulation modes. The genes included in the two patterns were shown in Supplementary Table 1.

**Figure 5:**
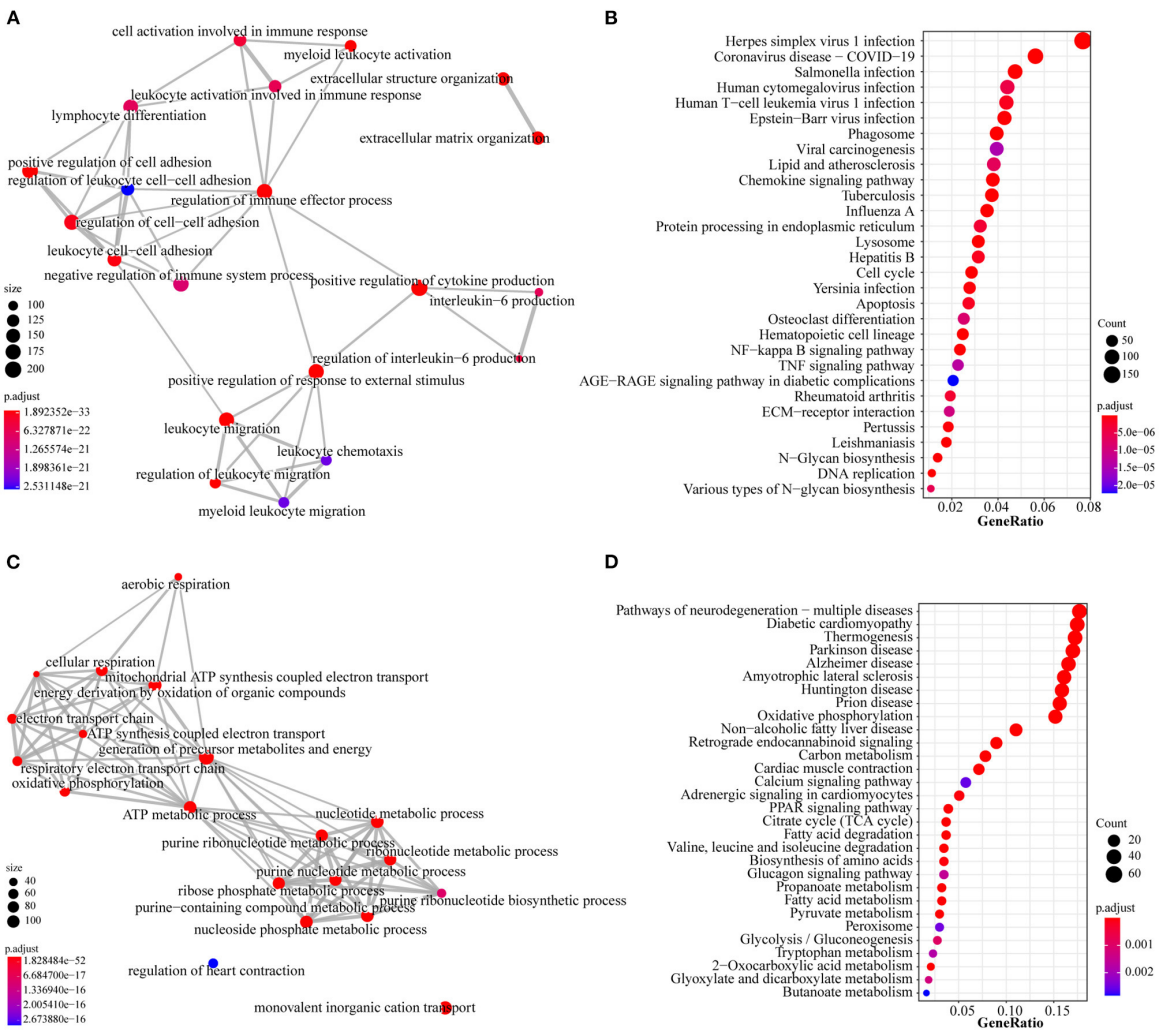
GO and KEGG analysis of type I profiles (profiles#4 and #7) and type II profile (profile #3). **(A)** Top 20 items for GO analysis of type I profiles. **(B)** Top 30 items for KEGG pathway analysis of type I profiles. **(C)** Top 20 items for GO analysis of type II profile. **(D)** Top 30 items for KEGG pathway analysis of type II profile. Colors represent adjusted *p*-value, the size of the dots represents the number of genes enriched into this biological process. The thickness of the line represents how many genes are enriched in common between the two biological processes.

To further investigate the function of the two profiles above, we performed GO and KEGG pathway analyses to determine a significant enrichment pathway for each expression profile (*p*-value < 0.05). For the type I profiles (profiles #4 and #7), the top 20 items of GO analysis and the top 30 items of KEGG analysis are shown (Figures 5A,B). The GO items included biological processes associated with immunity and inflammation, such as regulation of the immune effector process, cell activation involved in immune response and leukocyte chemotaxis. Moreover, KEGG items such as ECM-receptor interaction, apoptosis and the TNF signaling pathway are related to the immune response and apoptosis. The top 20 items of GO analysis and the top 30 items of KEGG analysis of the type II profile (profile #3) are shown in Figures 5C,D. The genes in this profile were associated primarily with energy metabolism and muscle contraction; GO analysis, such as ATP metabolic process, oxidative phosphorylation, regulation of heart contraction and nucleotide metabolic process; and KEGG analysis, such as PPAR signaling pathway, citrate cycle (TCA cycle) and cardiac muscle contraction.

### Screening of Transcription Factors in Type I Profiles (Profiles #4 and #7) and Identification of Drug Targets

**Figure 6:**
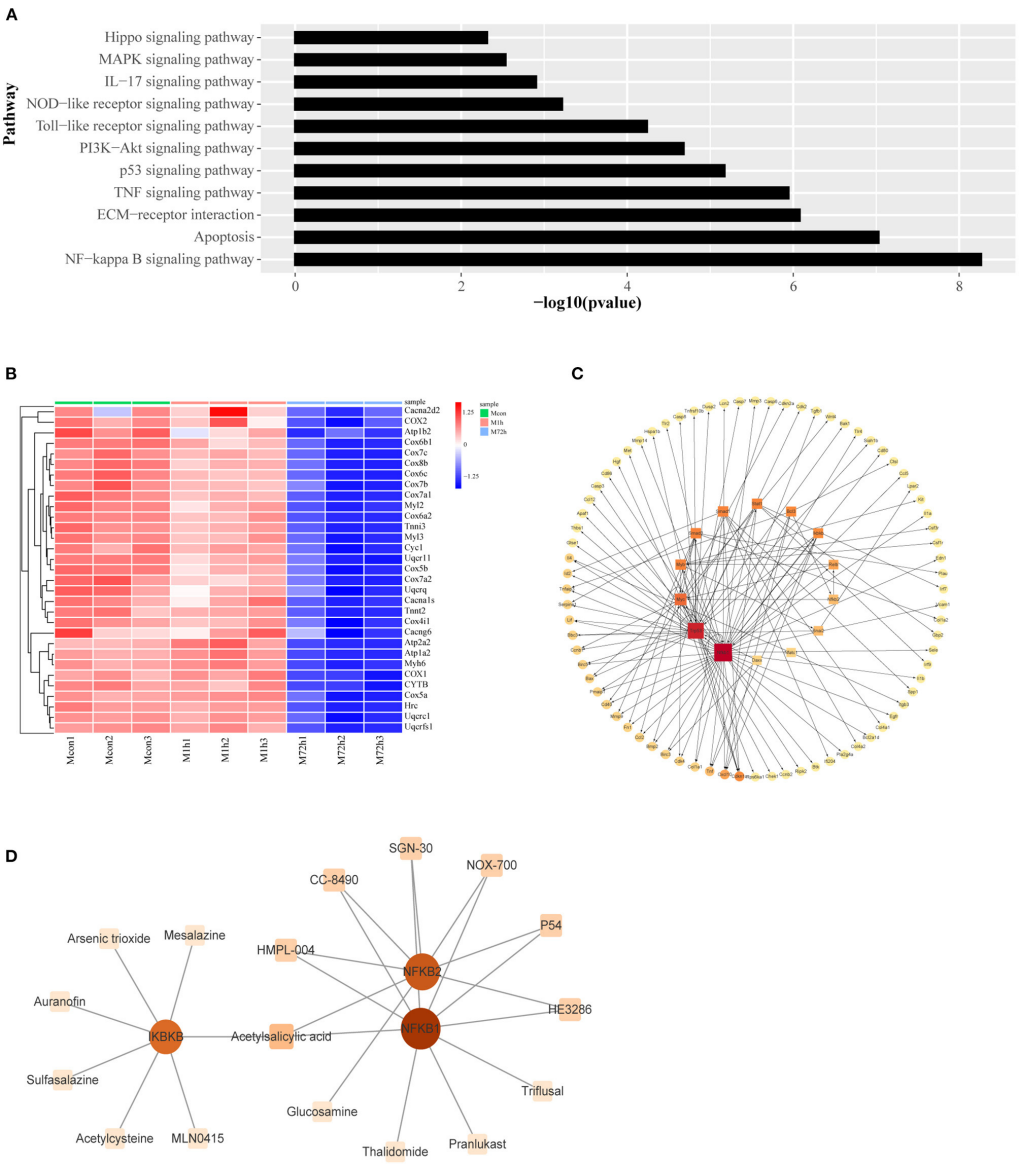
**(A)** Signaling pathways associated with apoptosis and inflammation in type I profiles (profiles #4 and #7). **(B)** Heatmap of genes associated with cardiac muscle contraction. **(C)** The transcriptional regulatory network of genes related to apoptosis and inflammation. The square nodes represent the transcription factors, the circular nodes represent the target genes, the node size represents node connectivity, and the arrow represents the regulatory direction. **(D)** Protein–drug interactions. The square nodes represent the drugs, the circular nodes represent the genes.

For the type I profiles (profiles #4 and #7), we selected genes enriched in apoptosis and inflammation related pathways (Figure 6A) as the focus of the following study. In addition, the cardiac muscle contractile pathway in the type II profile was given particular attention, and a heatmap of genes enriched in this pathway is shown in Figure 6B.

We identified transcription factors and constructed the transcriptional regulatory network of the type I profiles (profiles #4 and #7) by combining data with those in the TRRUST database. Regulatory network of transcription factors and their corresponding regulated target genes are shown in Figure 6C. Protein–drug interactions that may influence these transcription factors have been identified. The relationship of protein and drug is obtained from the DrugBank database. The results are shown in Figure 6D.

### Distribution of Differentially Methylated Regions

**Figure 7:**
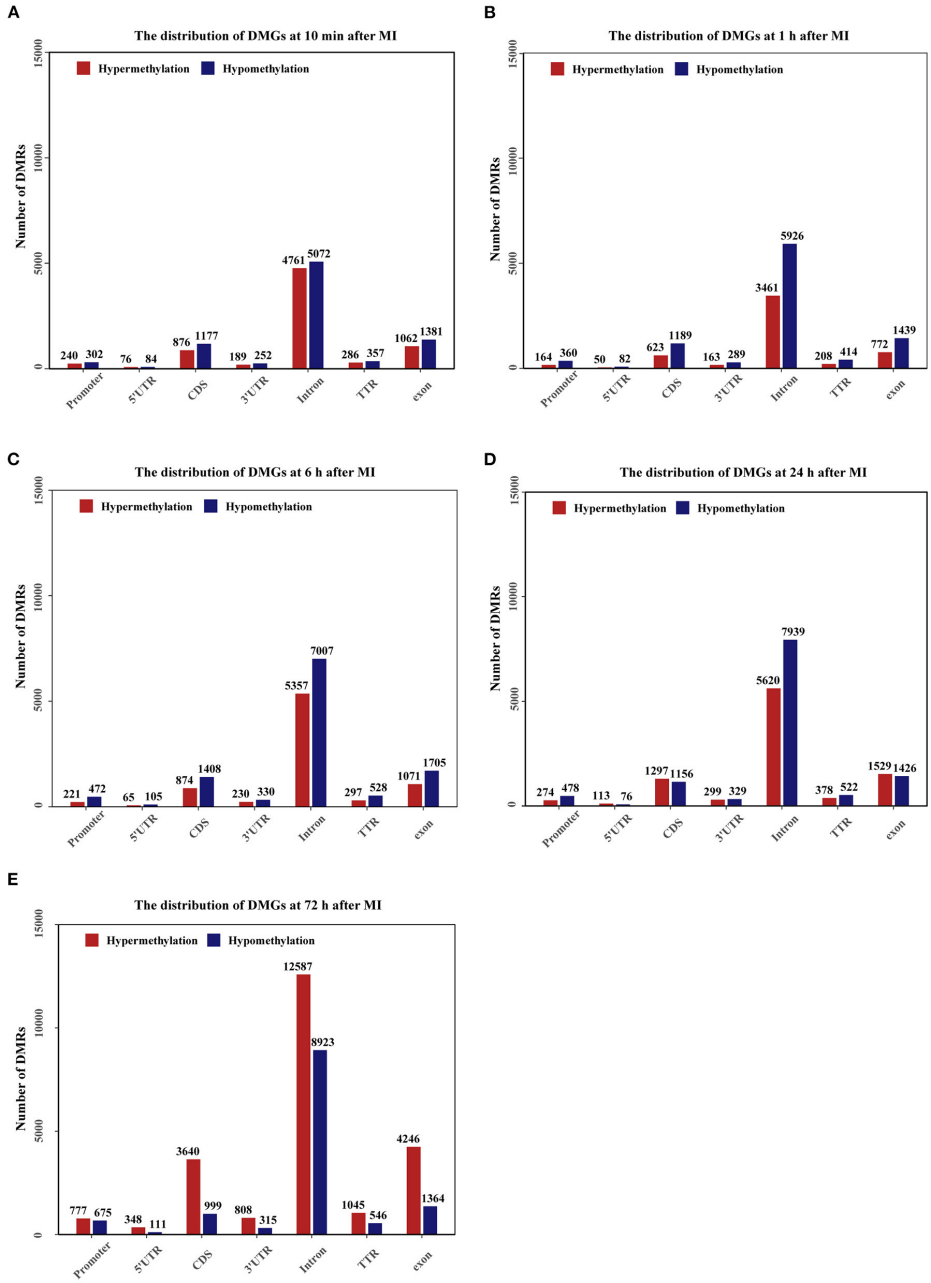
Bar plots showing the numbers of DMGs in promoter (2000bp upstream of the transcription start site), 5'UTR, CDS, 3'UTR, Intron, TTR (5000bp downstream of the transcription termination site), exon regions. The number of DMRs is given at the top of each graph bar. **(A–E)** The bar plots of DMGs distribution at 10 min, 1, 6, 24, and 72 h after MI.

The genomic distribution of differentially methylated regions (DMRs) across different genomic regions was investigated (Figures 7A–E), and the DMRs were found not to be randomly distributed across the genome. Most of the DMRs were located in the intron, CDS, and exon, whereas only a few of the DMRs were located in the promoter, 5'UTR, 3'UTR and TTR. In general, at 10 min, 1, 6, and 24 h after MI, the number of hypomethylated regions was higher than the number of hypermethylated regions, and at 72 h, the number of hypermethylated regions was higher than the number of hypomethylated regions. The distribution density of DMRs on different chromosomes is shown in Supplementary Figure 2.

### Integrated Analysis of DMGs and DEGs

**Figure 8:**
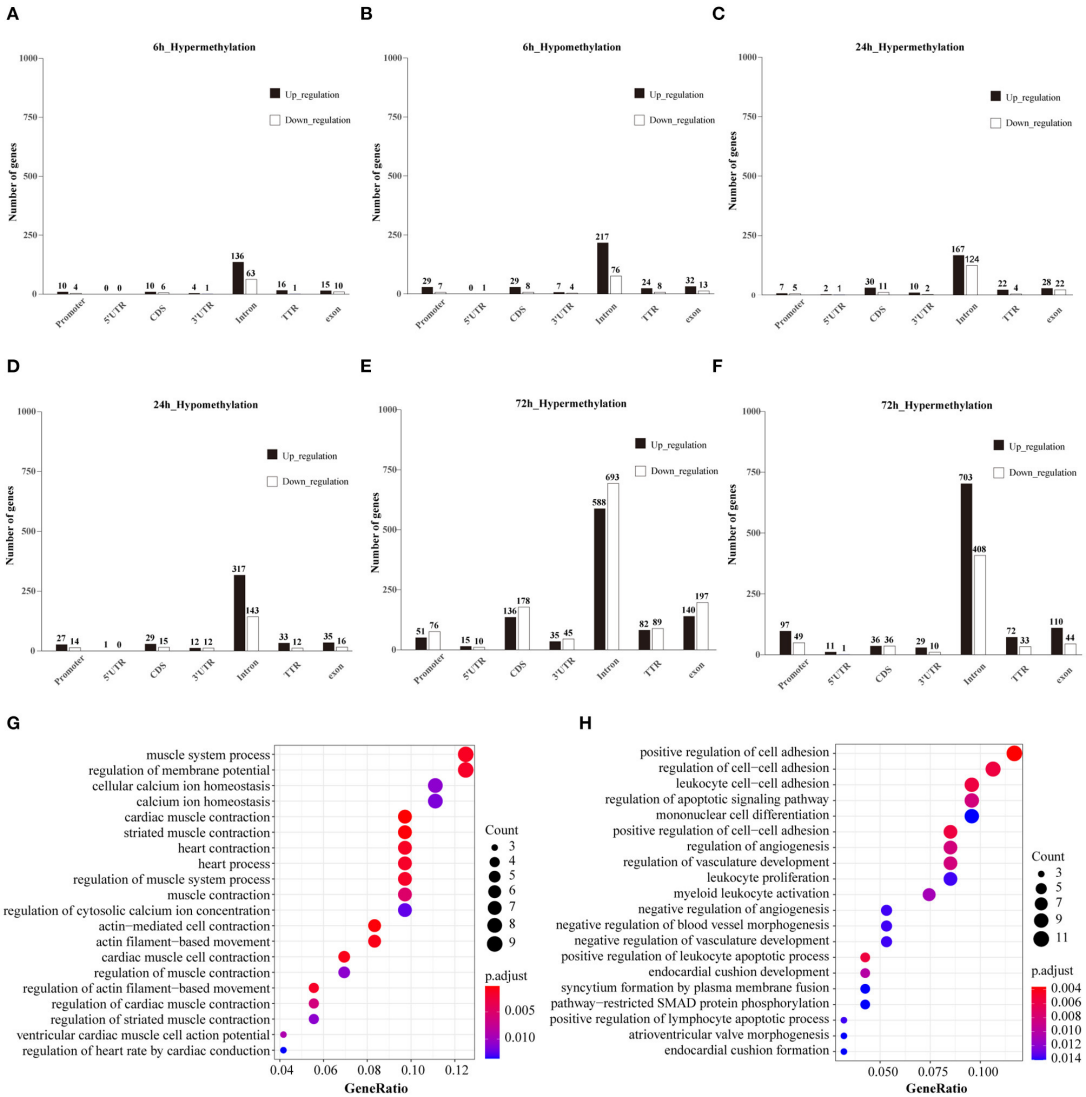
**(A–F)** Bidirectional expression patterns of differentially hypermethylated genes in different genomic elements. The number of genes is given at the top of each graph bar. **(G)** Top 20 items for GO analysis of hypermethylated and down-regulated genes at 72 h. **(H)** Top 20 items for GO analysis of hypomethylated and up-regulated genes at 72 h.

The subset of genes regulated by DNA methylation was identified by integrating MeDIP-Seq and RNA-Seq data. The distribution pattern of the genes with both differential methylation and expression (DMEGs) were shown (Supplementary Figure 3). Next, we analyzed the distribution of DMEGs in different gene elements. Due to the small number of DMEGs obtained at 10 min and 1 h, only the following three time points are displayed in bar plots. Bidirectional gene expression patterns were found in various genomic regions (Figures 8A–F). Of the methylated regions, methylation at the promoter region is considered the most important (27), so we focused on DMEGs in the promoter region. DNA methylation is often inversely proportional to the transcriptional activity of genes, so the degree of DNA methylation is bound to affect the expression level of genes, and then affect the function of genes (28). DMEGs at 10 min, 1, 6, and 24 h located in the promoter region with expression changes opposite to the methylation state are shown in Supplementary Table 2. We conducted GO enrichment analysis, and the results are shown (Figures 8G,H). The hypermethylated and downregulated genes were enriched in cardiac muscle contraction, and heart contraction, the hypomethylated and upregulated genes were enriched in regulation of the apoptotic signaling pathway, and regulation of cell-cell adhesion. The methylation of some important DMEGs in promoter region is shown in Table 1.

**Table 1:** Promoter methylation of DMEGs.

	**Gene ID**	**Chromosome**	**Methylated region**	**Fold change (MeDIP-seq)**	***p*-value**	**Fold change (RNA-seq)**	***p*** **-value**
10m	Atcay	chr10	81232001-81232500	1.297	3.17E-03	−1.32	1.12E-05
	Thbs1	chr2	118109501-118110000	−1.282	1.91E-02	1.772	2.50E-02
1h	Cytip	chr2	58161501-58162000	−1.368	1.80E-02	1.439	2.40E-03
	Ppp1r15a	chr7	45528001-45528500	−1.112	3.80E-02	1.394	5.52E-29
6h	1700029J07Rik	chr8	45976501-45977000	1.027	3.40E-02	−1.67	4.47E-05
	Bid	chr6	120918001-120918500	−1.163	1.40E-02	1.165	7.73E-03
			120917501-120918000	−1.175	3.10E-03		
	C4b	chr17	34744501-34745000	−1.228	1.30E-02	1.164	5.73E-10
	Gdf15	chr8	70632501-70633000	−1.265	2.21E-03	3.871	2.23E-27
			70633001-70633500	−1.196	1.73E-02		
	Hspb1	chr5	135887001-135887500	−1.468	2.00E-02	2.317	3.71E-10
24h	Akr1c14	chr13	4058501-4059000	1.307	1.68E-02	−1.181	8.44E-05
	Cd247	chr1	165787501-165788000	1.218	4.92E-02	−1.453	2.65E-03
	Gm5105	chr3	138069001-138069500	1.333	8.20E-03	−1.963	1.94E-02
	Lepr	chr4	101715501-101716000	1.272	9.31E-03	−1.093	7.23E-04
	Thbs1	chr2	118109501-118110000	−1.067	3.36E-02	5.574	3.00E-09
72h	Tnni3	chr7	4523001-4523500	1.382	2.58E-02	−2.198	4.23E-58
	Tnnc1	chr14	31207501-31208000	1.080	2.35E-02	−1.301	7.33E-35
	Cdkn1a	chr17	29092501-29093000	1.367	4.78E-02	2.129	1.21E-77
			29092001-29092500	1.220	3.21E-02		
	Thbs1	chr2	118109501-118110000	−1.217	2.17E-02	5.232	1.20E-220
	Tgfb2	chr1	186707001-186707500	−1.092	9.34E-03	1.274	4.02E-20

## Discussion

Worldwide, 20% of surviving MI patients have a second cardiovascular event in the first year, and ~50% of major coronary events occur in patients previously discharged with a diagnosis of ischemic heart disease (29). AMI has a high mortality rate worldwide, but fast and reliable diagnosis can reduce mortality (30). Although some biomarkers have been used in the clinical diagnosis of MI, biomarkers that meet the definition of ideal cardiac biomarkers have not been discovered (30, 31). Therefore, exploring more effective biomarkers will be helpful for the diagnosis and understanding the underlying mechanism of MI.

In our research, we analyzed the gene expression profile data and DNA methylation data of mice at 0 h, 10 min, 1, 6, 24, and 72 h after myocardial infarction. Based on the single time point analysis, the 5 time points after MI were compared with 0 h, and significant changes were found in the transcriptome and DNA methylation levels at each time point. Based on time series analysis, the genes in the type I profiles are associated with apoptosis and inflammation, and the genes in the type II profile are associated with energy metabolism and muscle contraction. Fourteen transcription factors were screened among genes associated with apoptosis and inflammation in the type I profiles, including Smad1, Stat1, Bcl3, Ibkbk, Relb, Nfkb2, Snai2, Nfatc1, Daxx, Nfkb1, Trp53, Myc, Myb and Smad3. Among these transcription factors, Ikbkb, Nfkb1 and Nfkb2 have been used as drug targets for the treatment of heart disease. Among DMEGs, we found that the Thbs1 gene hypomethylated and highly expressed at multiple time points after MI. Hypomethylated upregulated genes at 72 h were involved mainly in apoptosis, angiogenesis and cell adhesion, while hypermethylated downregulated genes at 72 h were involved mainly in the biological processes of muscle contraction and cardiac contraction.

In the GSEA of the genes at each time point, we found that there were pathways related to inflammation and apoptosis at 5 time points after MI, such as the B cell receptor signaling pathway, T cell receptor signaling pathway, ECM receptor interaction, NOD-like receptor signaling pathway and p53 signaling pathway. Acute myocardial ischemia, when it occurs, will trigger the initial proinflammatory response, the purpose of which is to remove necrotic cell debris in the myocardial infarction area (32). However, molecular evidence suggests that excessive or persistent proinflammatory states after AMI may worsen poor cardiac remodeling after myocardial infarction, which is related to poor clinical outcomes (32, 33). The dysregulated proinflammatory response leads to increased expression of cytokines and proteases, thereby inducing cardiomyocyte apoptosis, and thereby inhibiting myocardial function (32).

This study obtained 17 drugs that target the transcription factors we identified. Auranofin (34, 35), triflusal (36), and acetylsalicylic acid (37) have been found to have a potential role in the treatment of cardiovascular diseases. Therefore, further investigation of these drugs for the treatment of MI is worthwhile. Transcription factor deregulation has contributed to the pathogenesis of a large number of human diseases, from diabetes, inflammatory diseases and cardiovascular disease to many cancers, and therefore these proteins have great therapeutic potential (38). Among the 14 transcription factors screened, only Ikbkb, Nfkb1 and Nfkb2 are potential targets for cardiovascular drugs and the remaining transcription factors are also valuable to be studied. We hope that our results will provide a new direction for cardiovascular drug development.

DNA methylation plays an important role in gene modification in cardiovascular aging and disease (39). There is growing evidence that MI is associated with epigenetic changes, including DNA methylation. For example, using animal models of MI, abnormal hypermethylation of the CpG site in the upstream sequence of the ALDH2 promoter has been shown to be associated with myocardial ischemic injury (40). The Thbs1 gene is hypomethylated and highly expressed at 10 min, 24, and 72 h after MI. Thbs1 can maintain the movement and growth of vascular smooth muscle cells (VSMCs). Short-term stimulation of VSMCs with FGF-2 can cause a transient increase in TSP-1 (Thbs1) expression, which may be one of the ways FGF-2 promotes atherosclerosis (41). When overexpressed, Thbs1 instead of Thbs2, Thbs3, or Thbs4 has been shown by research to be able to cause fatal heart atrophy (42). Mechanistically, Thbs1 binds to and activates the endoplasmic reticulum stress effector PERK, induces its downstream transcription factor ATF4, and causes fatal autophagy-mediated cardiac atrophy (42). In tumors, the expression of THBS1 is induced by the hypoxic tumor microenvironment and may be regulated by the TP53 pathway (43). Most downregulation of THBS1 in cancers is epigenetic, resulting from promoter hypermethylation, altered expression of regulatory non-coding RNAs, or altered levels of oncogenic transcription factors (44, 45). In our study, the Thbs1 gene, as a target gene of Trp53, was hypomethylated and highly expressed at multiple time points. Therefore, the increase in Thbs1 expression may be the cause of both hypomethylation and Trp53 activation. Thbs1 (TSP-1) is an important extracellular matrix component that affects the functions of vascular smooth muscle cells, endothelial cells, fibroblasts and inflammatory cells, and is of great significance to cardiovascular diseases (46). Increasing the understanding of the role of Thbs1 in cardiovascular diseases may provide new directions for the treatment of cardiovascular diseases.

In the results of GO analysis of hypomethylation upregulated genes at 72 h, regulation of apoptotic signaling pathway, regulation of cell-cell adhesion, positive regulation of cell-cell adhesion, regulation of angiogenesis, and regulation of vasculature development were in the GO enrichment pathway. We found that Tgfb2 (TGF-β2) was enriched in the regulation of the apoptotic signaling pathway, regulation of angiogenesis and regulation of vasculature development. Transforming growth factor (TGF)-β family members play a key role in the inflammation and repair response after MI. TGF-β can regulate the survival response of cardiomyocytes, and mediate both angiogenic and angiostatic effects (47). The experiment showed that miR-323-3p overexpression can inhibit oxidative stress and cardiomyocyte apoptosis by regulating the TGF-β2/JNK pathway (48). Moreover, miR-19b in endothelial MPs (EMPs) induced by hypoxia was shown by a study to be able to reduce endothelial cell migration and angiogenesis by downregulating TGF-β2 expression, which may have inhibited the progression of atherosclerosis (49), indicating that Tgfb2 acts as an intermediate gene to mediate the apoptosis of cardiomyocytes or the progression of atherosclerotic disease. However, the mediating effect of TGF-β is highly dependent on the environment, with both beneficial and harmful effects (47). In our research, the Tgfb2 gene was highly expressed and hypomethylated, indicating that the high expression of Tgfb2 may be due to both upstream regulatory factors and epigenetics. The role of Tgfb2 methylation in myocardial infarction has not been reported in the literature. The mechanism of Tgfb2 action in myocardial infarction needs further experimental verification.

The GO analysis of hypermethylated downregulated genes at 72 h suggested that these genes are involved multiple biological processes related to muscle contraction and heart contraction, such as muscle system process, cardiac muscle contraction, and heart contraction. Interestingly, the Tnni3 gene appears in these biological processes. Tnni3 is expressed only in myocardial tissue, encoding mainly cardiac troponin I (cTnI), which together with cardiac troponin T (cTnT) and cTnC form a troponin complex, that regulates the contraction and relaxation of the heart. Clinically, after myocardial ischemia-reperfusion injury, plasma cTnI is significantly increased, accompanied by a large area of cTnI loss in myocardial tissue, and plasma cTnI level will increase with the prolonged ischemia time (50), and the missing area will increase with the time of myocardial ischemia or infarction (51). The prolonged time and increase suggest that plasma cTnI content is negatively correlated with myocardial tissue cTnI expression. Cardiac troponin I and T are essential proteins for cardiac function and they are very sensitive markers for detecting myocardial injury (52). In our time series analysis, we also found Tnni3, which is present in the type II profile and is enriched in the cardiac muscle contraction pathway. With the development of MI, the expression of Tnni3 gradually decreased. Interestingly, the hypermethylation state of the promoters of Tnni3 did not always exist, but hypermethylation began to appear after 72 h. Hypermethylation of the promoter of Tnni3 may be one of the reasons for the aggravation of the disease. There is no report on the methylation of Tnni3 in cardiovascular diseases, and the specific impact needs further experimental verification.

Several limitations exist in our study as well. Our study was limited to the early stages of myocardial infarction, and further studies are needed on the performance of these results in the longer term after MI. Furthermore, the genetic and methylation features we obtained that influence the disease process need to be validated by more experiments.

## Conclusion

The present study provided comprehensive mRNA and methylation expression profiling in the heart. A single time point analysis found that the inflammatory response after MI continued within 72 h. Time series analysis identified two expression patterns. In type I profiles, 14 transcription factors related to inflammation and apoptosis were screened out and 17 drugs that target these transcription factors were identified. Some drugs have been reported to be effective in the treatment of cardiovascular diseases. Integrative analysis of the transcriptome and methylation screened out genes that reverse changes in methylation and gene expression, represented by Thbs1, Tgfb2 and Tnni3. As one of the target genes of Trp53, Thbs1 is in a state of hypomethylation and high expression at multiple time points, and its expression changes may be the dual role of upstream regulatory factors and methylation. However, more experiments in the near future are needed to verify these conclusions.

## Data Availability Statement

The datasets presented in this study can be found in online repositories. The name of the repository is GEO database and accession number is GSE206282.

## Ethics Statement

We retrieved all data from publicly available resources and we required no ethical approvals for dissemination of this purely academic information.

## Author Contributions

YH: conceptualization, methodology, and writing- original draft preparation. BD, YL, and XG: writing- reviewing and editing and supervision. XW and JW: conceptualization, methodology, and software. YZ: data curation and supervision. YG and XC: visualization and investigation. CL: supervision. All authors contributed to the article and approved the submitted version.

## Funding

This work was supported by the National Natural Science Foundation of China (81772829 and 81830052), the Special Program for Collaborative Innovation, the Construction Project of Shanghai Key Laboratory of Molecular Imaging (18DZ2260400), the Shanghai Municipal Commission of Health and Family Planning (202150007), the Discipline Leaders Training Program of Pudong New District Health Bureau (No. PWRd2020-07) and Top-100 Talent Cultivation Plan of Shanghai University of Medicine and Health Sciences.

## Conflict of Interest

The authors declare that the research was conducted in the absence of any commercial or financial relationships that could be construed as a potential conflict of interest.

## Publisher's Note

All claims expressed in this article are solely those of the authors and do not necessarily represent those of their affiliated organizations, or those of the publisher, the editors and the reviewers. Any product that may be evaluated in this article, or claim that may be made by its manufacturer, is not guaranteed or endorsed by the publisher.

## Supplementary Material

Supplementary Figure 1 Bar chart of the number of up-regulated and down-regulated differential genes at different time points.

Supplementary Figure 2 Rainfall plots for differentially methylated regions (DMR) and their genomic densities. In the plot, red corresponds to hypermethylated DMRs (gain of methylation) and blue corresponds to hypomethylated DMRs (loss of methylation). **(A–E)** The rainfall plots of DMRs distribution at 10 min, 1, 6, 24, and 72 h after MI.

Supplementary Figure 3 Venn diagram of DEGs and DMGs. Each group was further divided into up-regulation group and down-regulation group. The number of genes is given in the middle of each figure section. **(A–E)** The venn diagram at 10 min, 1, 6, 24, and 72 h after MI.

Supplementary Table 1 The genes included in profile #3, profile #4 and profile #7 and their FPKM expression values at 0, 1, and 72 h.

Supplementary Table 2 The genes localized in the promoter region at 10 min, 1, 6, and 24 h and with expression changes opposite to the methylation status.

## Abbreviations

MI: myocardial infarction
RNA-seq: RNA sequencing
MeDIP-seq: methylated DNA immunoprecipitation sequencing
DEGs: differentially expressed genes
DMRs: differential methylation regions
FPKM: fragments per kilobase million
ES: Enrichment score
STEM: Short Time-series Expression Miner
GO: Gene Ontology
KEGG: Kyoto Encyclopedia of Genes and Genomes
TF: transcription factor
MSigDB: Molecular Signatures Database
DMEGs: differentially methylated and expressed genes.
