# Corrigendum: Analysis of time series gene expression and DNA methylation reveals the molecular features of myocardial infarction progression

**DOI:** 10.3389/fcvm.2022.990217

**Published:** 2022-07-27

**Authors:** Yuru Han, Baoyu Duan, Jing Wu, Yanjun Zheng, Yinchen Gu, Xiaomeng Cai, Changlian Lu, Xubo Wu, Yanfei Li, Xuefeng Gu

**Affiliations:** ^1^Shanghai Key Laboratory of Molecular Imaging, Zhoupu Hospital, Shanghai University of Medicine and Health Sciences, Shanghai, China; ^2^School of Medical Instrument and Food Engineering, University of Shanghai for Science and Technology, Shanghai, China; ^3^School of Nursing, Shanghai University of Traditional Chinese Medicine, Shanghai, China; ^4^School of Basic Medical Sciences, Fudan University, Shanghai, China; ^5^Seventh People's Hospital of Shanghai University of Traditional Chinese Medicine, Shanghai, China; ^6^School of Pharmacy, Shanghai University of Medicine & Health Sciences, Shanghai, China

**Keywords:** myocardial infarction, time series, MeDIP-seq, RNA-seq, DNA methylation

In the published article, an author name was incorrectly written as “Baoyu Duan^1*^.” The correct spelling is “Baoyu Duan^1†^.”

In the published article, an author name was incorrectly written as “Xubo Wu^3,5†^.” The correct spelling is “Xubo Wu^3,5*^.”

In the published article, there was an error in the “Data availability statement: The data repository number was not provided in the original article.” The correct “Data availability statement” appears below.

The datasets presented in this study can be found in online repositories. The name of the repository is GEO database and accession number is GSE206282.

In the published article, the reference for “To identify the biological signaling pathways involved in gene expression at different time points, normalized gene expression data obtained from five different time groups were used to perform GSEA analysis against controls for MI using the Gene Set Enrichment Analysis (GSEA) tool (v4.1)” was incorrectly written as “Chen K, She D, Tan L, Lai D, Han Y, Gu Y, et al. (2022). A Pan-Cancer Analysis Reveals the Prognostic and Immunotherapeutic Value of ALKBH7. *Front Genet*. 13:822261. doi: 10.3389/fgene.2022.822261.” It should be “Lai D, Tan L, Zuo X, Liu D, Jiao D, Wan G, et al. (2021). Prognostic Ferroptosis-Related lncRNA Signatures Associated With Immunotherapy and Chemotherapy Responses in Patients With Stomach Cancer. *Front Genet*. 12:798612. doi: 10.3389/fgene.2021.798612.”

The authors apologize for this error and state that this does not change the scientific conclusions of the article in any way. The original article has been updated.

## Publisher's note

All claims expressed in this article are solely those of the authors and do not necessarily represent those of their affiliated organizations, or those of the publisher, the editors and the reviewers. Any product that may be evaluated in this article, or claim that may be made by its manufacturer, is not guaranteed or endorsed by the publisher.

