# Supplementary material for: Analysis of Time Series Gene Expression and DNA Methylation Reveals the Molecular Features of Myocardial Infarction Progression
Source: Front Cardiovasc Med. 2022 Jun 24;9:912454. doi: 10.3389/fcvm.2022.912454 (PMC9263976; doi:10.3389/fcvm.2022.912454)
Supplement: Supplementary Figure 1 — Bar chart of the number of up-regulated and down-regulated differential genes at different time points. [file Data_Sheet_1.ZIP › Supplementary materials1/Figure S1.pdf]

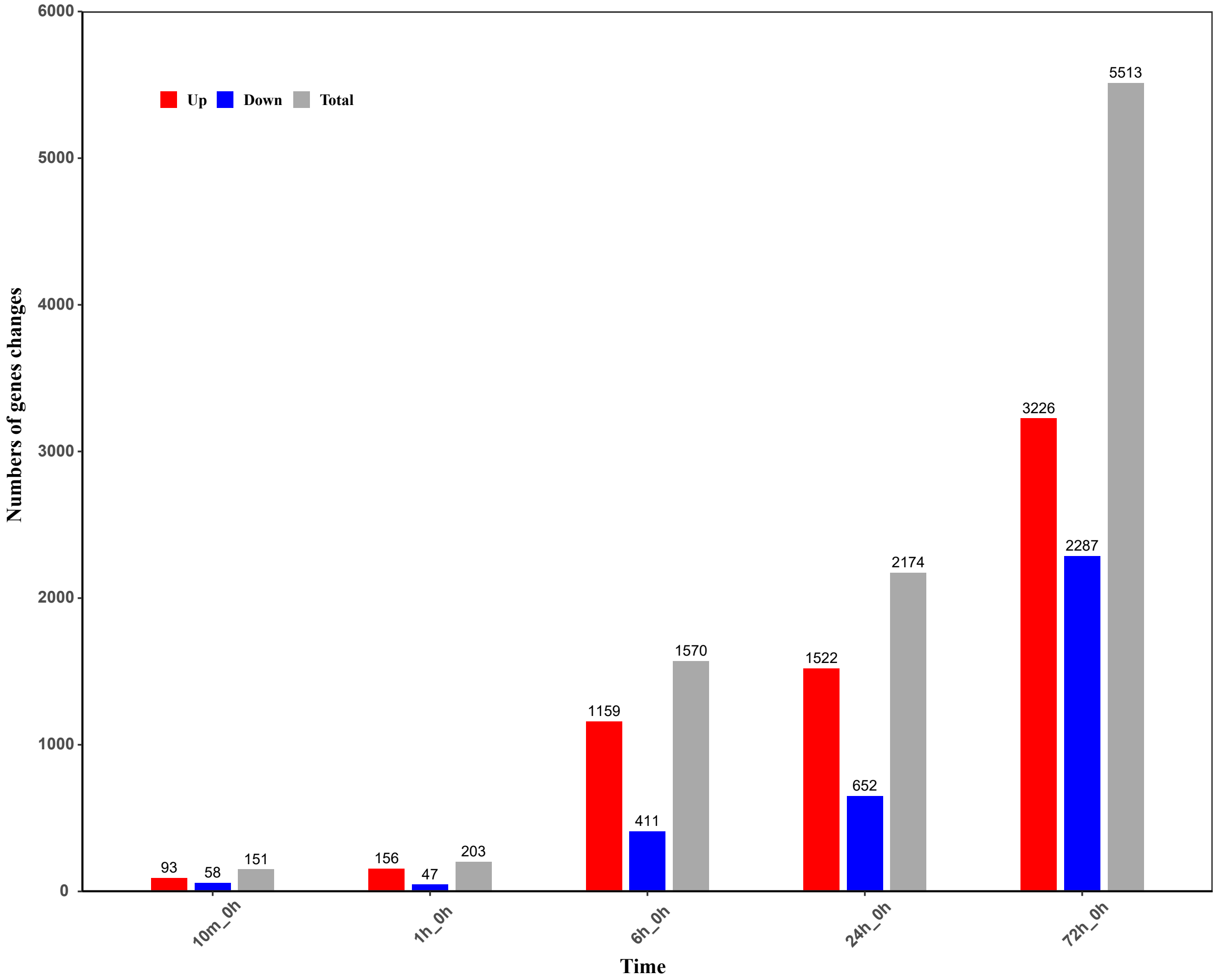

Figure S1. Bar chart of the number of up-regulated and down-regulated differential genes at different time points.
