# Supplementary material for: Analysis of Time Series Gene Expression and DNA Methylation Reveals the Molecular Features of Myocardial Infarction Progression
Source: Front Cardiovasc Med. 2022 Jun 24;9:912454. doi: 10.3389/fcvm.2022.912454 (PMC9263976; doi:10.3389/fcvm.2022.912454)
Supplement: Supplementary Figure 1 — Bar chart of the number of up-regulated and down-regulated differential genes at different time points. [file Data_Sheet_1.ZIP › Supplementary materials1/Figure S2.pdf]

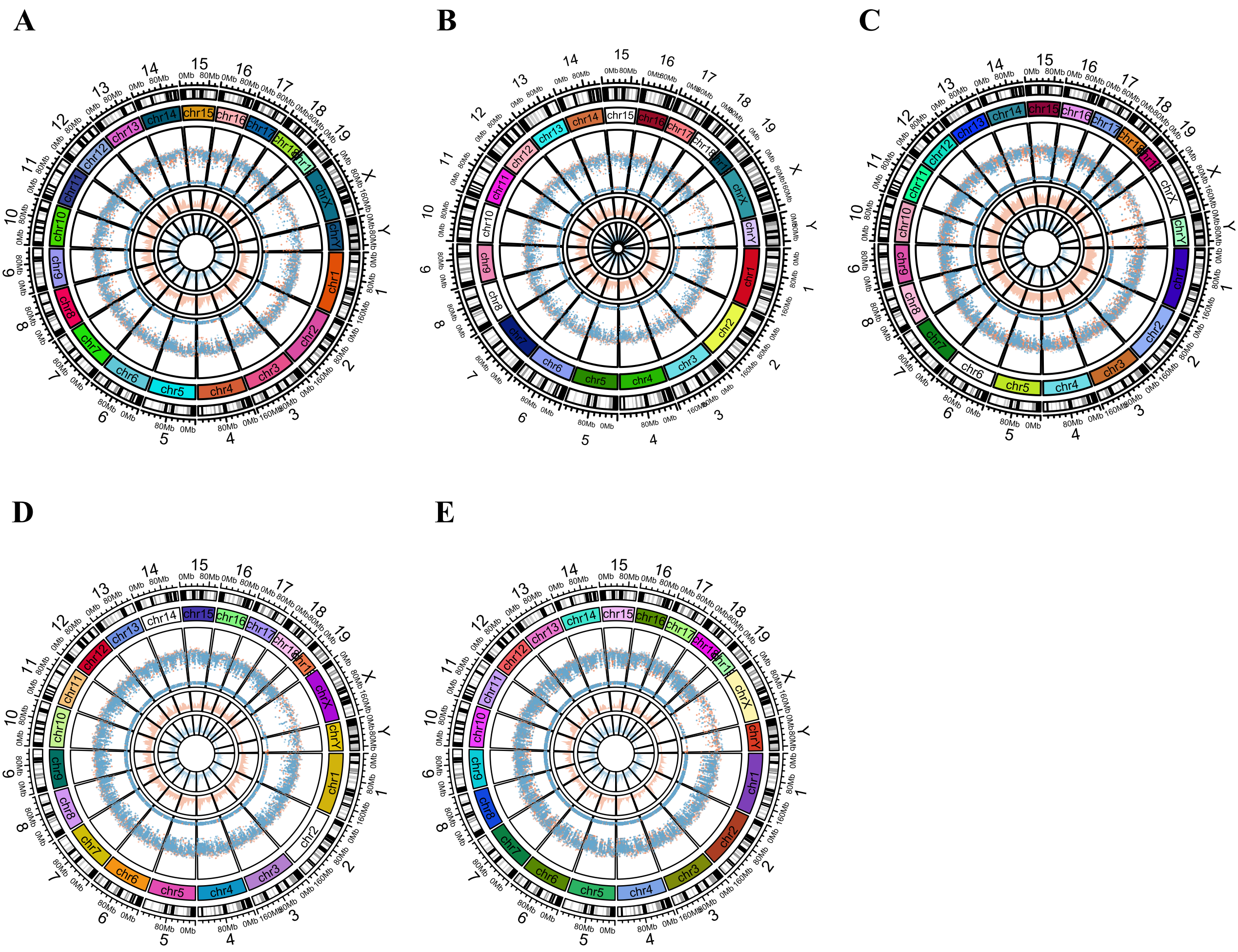

Figure S2. Rainfall plots for differentially methylated regions (DMR) and their genomic densities. In the plot, red corresponds to hypermethylated DMRs (gain of methylation) and blue corresponds to hypomethylated DMRs (loss of methylation). (A-E) The rainfall plots of DMRs distribution at 10min, 1h, 6h, 24h, 72h after MI.
