# Supplementary material for: Analysis of Time Series Gene Expression and DNA Methylation Reveals the Molecular Features of Myocardial Infarction Progression
Source: Front Cardiovasc Med. 2022 Jun 24;9:912454. doi: 10.3389/fcvm.2022.912454 (PMC9263976; doi:10.3389/fcvm.2022.912454)
Supplement: Supplementary Figure 1 — Bar chart of the number of up-regulated and down-regulated differential genes at different time points. [file Data_Sheet_1.ZIP › Supplementary materials1/Figure S3.pdf]

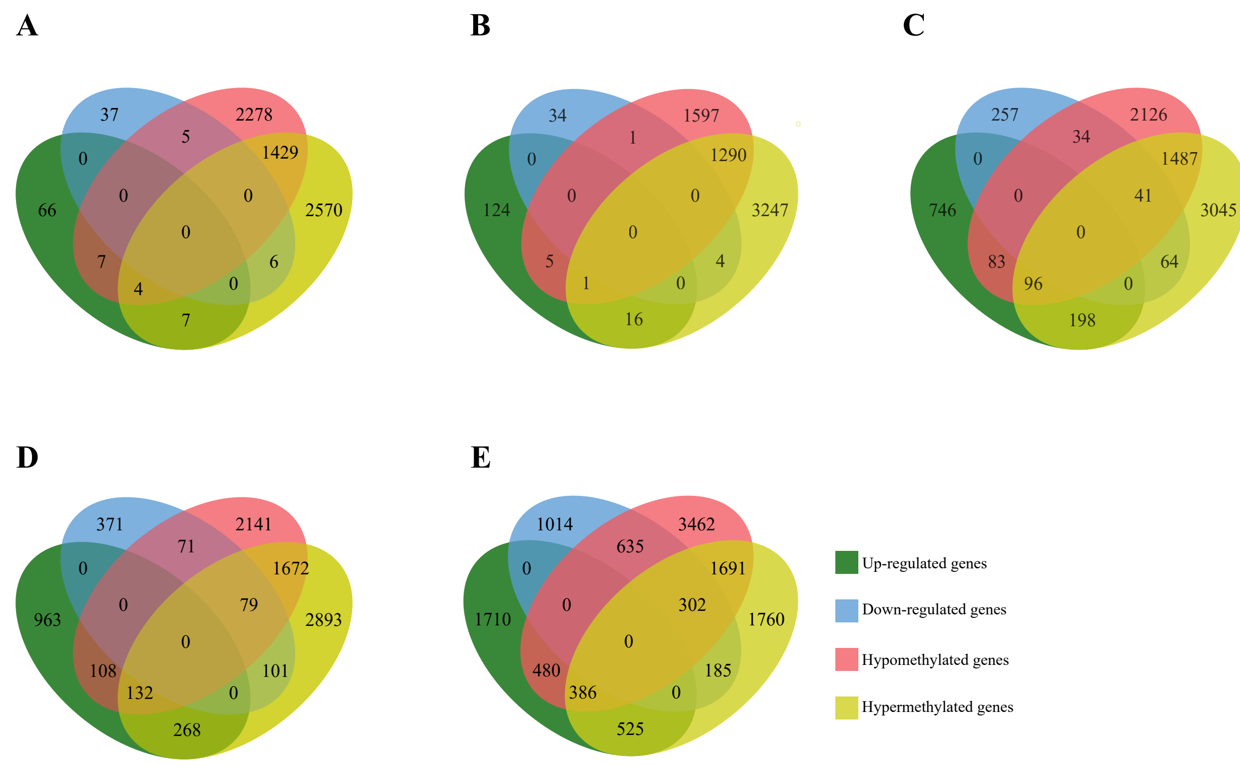

Figure S3. Venn diagram of DEGs and DMGs. Each group was further divided into up-regulation group and down-regulation group. The number of genes is given in the middle of each figure section. (A-E) The venn diagram at 10min, 1h, 6h, 24h, 72h after MI.
