# Supplementary material for: Analysis of Time Series Gene Expression and DNA Methylation Reveals the Molecular Features of Myocardial Infarction Progression
Source: Front Cardiovasc Med. 2022 Jun 24;9:912454. doi: 10.3389/fcvm.2022.912454 (PMC9263976; doi:10.3389/fcvm.2022.912454)
Supplement: Supplementary Figure 1 — Bar chart of the number of up-regulated and down-regulated differential genes at different time points. [file Data_Sheet_1.ZIP › Supplementary materials1/Table S1.pdf]

Gene Symbol	0h	1h	72h	id
Cox5a	156.1751578	162.3207745	77.53972878	profile #3
Dlat	92.53850529	106.3032199	44.61516653	profile #3
Fgf6	0.11926456	0.111947491	0.045917238	profile #3
Gmpr	53.38558	52.1651884	24.7631522	profile #3
Tpd52l1	6.052330858	5.765850779	2.652935326	profile #3
Cdh4	0.30495085	0.294542072	0.135544751	profile #3
Dbt	21.78748826	24.4281428	10.51299305	profile #3
Ndufa9	234.8155277	216.2529013	99.13583703	profile #3
Car4	12.00668356	13.13681353	2.43445397	profile #3
Dbh	1.391205367	1.315629213	0.474109609	profile #3
Scn4a	2.979862348	2.791435579	1.0427891	profile #3
Fndc5	129.8379425	131.436043	43.20663015	profile #3
Irx2	2.022877805	2.28772019	0.964282457	profile #3
Sgca	76.80106037	78.06570233	28.61041665	profile #3
Tcea3	59.86203675	61.55529565	16.08402335	profile #3
Grik3	0.051582066	0.078754409	0.023831113	profile #3
Srpk3	11.50464616	10.05600912	5.697781553	profile #3
Pnck	5.095760482	4.616078188	2.269346542	profile #3
Tmem25	4.380608579	3.952533791	1.504271095	profile #3
Slc25a42	11.96918927	13.02206244	4.857748382	profile #3
Rpl3l	157.7769188	148.3326286	34.08120477	profile #3
Slc9a3r2	98.2267657	93.0489598	41.67351073	profile #3
Gnmt	2.532987404	2.349369049	0.629805975	profile #3
Tmem143	15.71755163	15.08264619	5.741738899	profile #3
Plin4	18.68323806	34.0015025	9.26321003	profile #3
Kcnn1	4.029355961	4.236590584	1.669461174	profile #3
Crhr2	7.112353114	5.750705817	2.450658732	profile #3
Inmt	8.751037463	9.771828576	0.997111772	profile #3
Prodh	8.547141057	9.851633828	2.866806478	profile #3
Gcdh	30.19187953	27.60032473	11.55280036	profile #3
Lin7b	0.759282373	0.573590349	0.186940201	profile #3
Efnb3	34.12146421	33.54392801	8.935211505	profile #3
Hlf	7.281110911	6.718654111	0.467381833	profile #3
Gstm7	21.16197278	21.94770773	6.958156206	profile #3
9330159F19Rik	0.088555374	0.122825519	0.051218832	profile #3
Ndrg2	589.3642554	569.1712151	274.7191847	profile #3
Etfb	100.6046818	86.22473189	27.99940763	profile #3
Aqp1	315.2257989	353.5140344	67.34096751	profile #3
Mtfp1	110.3492217	107.6207967	38.99931493	profile #3
Corin	25.88959412	36.83946209	11.33849027	profile #3
Ndufs3	152.4469187	142.4707304	70.13137184	profile #3
Tmod4	10.70972421	8.790319913	2.601666125	profile #3
Cs	186.522759	188.183192	79.77485741	profile #3
Uqcc1	28.11632031	26.46772116	13.26510599	profile #3
Atp5g1	307.7674453	283.4044286	115.4589545	profile #3
Fam131c	0.445480498	0.380232309	0.208912219	profile #3
Crip2	540.6254544	524.2876576	267.3478369	profile #3
Snai3	4.236120156	3.012196614	0.671974237	profile #3
Slc5a6	3.2124357	3.04365061	1.233496182	profile #3
Tcf23	0.889214862	1.654740338	0.135076556	profile #3
P4htm	3.772044589	4.146076555	1.901991157	profile #3
Sod2	154.0560241	161.2121874	65.73904867	profile #3
Vwa7	2.597002156	2.913118747	1.268228679	profile #3
Atp1a2	130.7284063	139.4525439	42.15613541	profile #3
Scube2	3.442575532	3.977427127	1.843330001	profile #3
Tcap	2241.580174	1972.290455	446.8791709	profile #3

Mid1ip1	83.10780582	85.03615085	29.68463792	profile #3
Rbfox1	8.686026032	8.054446598	3.110643724	profile #3
Bmp7	0.381213369	0.338935617	0.181467041	profile #3
Prr29	1.974860181	1.716445828	0.846074291	profile #3
G0s2	64.24577142	72.84387272	36.26693562	profile #3
Sdhb	356.7448504	333.8528485	149.3078335	profile #3
Slc38a3	11.91179001	12.37029055	3.619284397	profile #3
Cacna2d2	0.380223332	0.314889825	0.102249779	profile #3
Uckl1os	1.771853638	1.591718436	0.50606783	profile #3
Slc5a1	0.093329467	0.190548051	0.03084576	profile #3
Plin5	52.47212996	65.97478748	24.8114225	profile #3
Cdh23	1.168869841	0.956909479	0.224200368	profile #3
Tango2	22.90527836	22.08322842	10.26248155	profile #3
Ndufs2	437.2923051	423.6531182	193.2269715	profile #3
Myl2	3851.873738	3492.160263	1105.404685	profile #3
Cox6c	291.8900497	259.471252	119.9808713	profile #3
Clec4f	0.057097926	0.105491596	0.018446885	profile #3
Slc25a11	233.5510588	231.6271906	105.7207241	profile #3
Gata2	5.991897008	8.499128948	2.50148946	profile #3
Ptgds	145.4525521	125.7031972	50.41966551	profile #3
Slc25a13	34.42705664	33.89816263	14.61007252	profile #3
Slamf1	0.062219645	0.086094458	0.027418454	profile #3
Mov10l1	13.23649682	12.37459368	5.05621925	profile #3
Lpl	448.8598939	465.6656951	180.8629647	profile #3
Rxrg	22.70906865	22.60575138	9.306955327	profile #3
Hsd11b1	4.655276443	5.922107833	2.729751844	profile #3
Eef1a2	670.2825653	629.2597387	264.1102198	profile #3
Mpped2	3.707447532	4.642585035	1.969685652	profile #3
Ndufa1	680.8637434	613.4815723	306.3562649	profile #3
Cd300lg	52.35636443	45.21031614	16.89730082	profile #3
Rhbdl3	4.656773704	5.597776532	2.791529473	profile #3
Slc12a5	0.335620754	0.320261672	0.152990333	profile #3
Cox7c	798.5219676	745.4798064	359.1035616	profile #3
Tbx5	4.69793594	4.837053669	1.803789668	profile #3
Mapt	7.949642182	8.046259299	3.601306625	profile #3
Ybx2	13.22319311	11.85582343	4.860494821	profile #3
Slc2a4	105.2943153	98.52686363	33.59037175	profile #3
Acadvl	228.397984	246.1548246	98.12233191	profile #3
Tbx3	2.655358134	3.792065312	1.459150563	profile #3
Atp5g3	586.5855844	578.5995567	268.18499	profile #3
Acsl1	65.3273647	73.53632907	24.4455197	profile #3
Sfrp5	0.716770306	1.124995692	0.133253685	profile #3
Myh11	13.70752863	13.04920861	4.824900664	profile #3
Mb	9188.813948	8530.979177	3012.105822	profile #3
Aldh3a1	0.241061074	0.363884299	0.093887235	profile #3
Mdh2	295.5300364	291.7410422	127.7633937	profile #3
Fmc1	114.4015289	102.094472	45.62665492	profile #3
Grm1	2.299829011	2.747120884	1.346965095	profile #3
Rtn4ip1	15.76359157	16.11646452	7.727777398	profile #3
Rhobtb1	23.6115981	26.20179596	6.610454215	profile #3
Cabcoco1	7.722532687	8.13818344	3.286834366	profile #3
Map7	2.981212127	3.120809664	1.430369358	profile #3
Ndufa12	241.4398328	254.3012494	117.850966	profile #3
Cry1	4.548077753	5.106144229	2.524765478	profile #3
Timp3	28.27635313	35.46766058	11.53205121	profile #3
Mypn	19.10404965	19.32000795	9.24017486	profile #3
Pbld2	0.466578508	0.55208071	0.220559286	profile #3
Ptprr	0.586978366	0.634107438	0.252463492	profile #3

Ndufs7	368.4177771	373.0064121	169.8854262	profile #3
Ptprb	22.40901676	20.79960868	10.10723854	profile #3
Uqcr11	1064.660491	981.2812389	438.6249815	profile #3
Wif1	0.911836967	0.94629409	0.450751569	profile #3
Slc36a2	6.458915217	6.051686559	2.072557099	profile #3
Stc2	4.753364431	3.959157692	2.069918709	profile #3
Mdh1	629.7192194	600.1614131	306.5241391	profile #3
Acsl6	1.110040356	1.330030678	0.473569901	profile #3
Cyfip2	20.44132797	20.73081333	10.03027742	profile #3
Rasgef1c	0.290388012	0.214095441	0.084022231	profile #3
Adcy1	0.435668454	0.549567239	0.249367355	profile #3
Osbp2	4.029423657	3.532421165	1.246865575	profile #3
Ogdh	264.873543	270.2362223	111.7082656	profile #3
Pgam2	681.1344487	610.9711705	247.0123965	profile #3
Dynll2	333.4039656	333.0402672	164.0861126	profile #3
Shmt1	2.619474278	2.792650249	1.31492069	profile #3
Ntsr2	1.522434458	1.728923621	0.726496066	profile #3
Lpin1	12.56124929	15.5146427	6.282003127	profile #3
Rasl10b	28.74555935	30.80366237	11.68046642	profile #3
Grin2c	0.112042069	0.122757672	0.042453162	profile #3
Cluh	41.03112354	47.54924606	23.56929139	profile #3
Acox1	58.47960244	61.86085002	28.92616373	profile #3
Coro6	87.51929688	77.59566811	39.31581853	profile #3
Per1	13.68726106	17.47982443	5.886107192	profile #3
L2hgdh	13.08681369	14.2591648	6.002054576	profile #3
Cdkl1	4.232886865	4.291280278	2.143996194	profile #3
Sptb	9.631398144	12.0642384	5.151737176	profile #3
Asb2	164.7010015	153.0606753	61.33399634	profile #3
Dpf3	1.522735558	1.805495995	0.733631518	profile #3
Acot2	25.16566397	27.54138968	11.60313256	profile #3
Acot3	0.185681662	0.282624215	0.049094823	profile #3
Fam161b	2.067696372	2.761812802	1.037740283	profile #3
Entpd5	24.64832506	22.37596934	11.43141125	profile #3
Aldh6a1	30.77819934	31.28457359	10.74146094	profile #3
Esrrb	5.036424493	5.523517229	1.861884628	profile #3
Slc25a29	5.914339143	5.88157342	1.766618379	profile #3
Fbp2	20.19271009	23.92396321	6.147146405	profile #3
Tppp	5.466206878	5.200962399	1.530075472	profile #3
Sdha	402.4053554	417.087209	175.7225659	profile #3
Ndufs6	528.4235429	483.1388691	201.1101159	profile #3
Ckmt2	1176.136686	1107.583242	378.3695045	profile #3
Mccc2	21.27911845	22.0989909	9.288888698	profile #3
Rgs7bp	0.770869067	0.823815178	0.367131325	profile #3
Pdhb	208.1802366	203.8651617	96.08806704	profile #3
Fam107a	1.770510631	4.266059128	0.430988452	profile #3
Nr1d2	20.8238181	18.23579722	6.44395678	profile #3
Thrb	1.678644492	1.873244607	0.874332006	profile #3
Ldb3	145.9703272	132.6486573	70.08574383	profile #3
Opn4	0.803225258	0.740677694	0.397196344	profile #3
Ppif	28.58376123	27.05921342	12.39866184	profile #3
Dnah12	0.019581005	0.021706198	0.010683816	profile #3
Asb14	31.7400784	28.65424062	8.976967719	profile #3
Galnt15	9.657865345	9.139511049	4.186419326	profile #3
Ogdhl	60.92447497	65.9103373	12.05306298	profile #3
Gdf10	1.864676262	2.343094838	0.921194672	profile #3
Mipep	16.2999074	17.35540924	8.153885941	profile #3
Rgcc	56.88339508	78.51814549	22.50175756	profile #3
Scara5	4.749647316	5.211830374	2.468758132	profile #3

Ephx2	83.26611568	87.21274992	29.91105936	profile #3
Chrna2	0.38209138	0.61949501	0.144438085	profile #3
Sucla2	151.088144	146.6640892	65.29530818	profile #3
Pdzd2	4.004419525	4.171191467	1.973876105	profile #3
Carmil3	0.641161211	0.725704079	0.304039522	profile #3
Fitm1	125.6544023	120.8388486	38.33801416	profile #3
Cmbl	27.2297127	27.33238644	10.44616295	profile #3
Ank	49.40199715	52.04982663	25.60843283	profile #3
Marchf11	0.066052891	0.076527092	0.032010009	profile #3
Grhl2	0.420310461	0.425140249	0.130284963	profile #3
Ndufb9	1044.211588	1000.235014	452.1815455	profile #3
Klhl38	4.230968373	3.276048188	1.236408294	profile #3
Ppara	5.455734628	7.996961468	2.661737368	profile #3
Tef	29.83676011	26.65360454	5.209292861	profile #3
Ttll1	20.88774415	18.95617459	7.420003393	profile #3
Aco2	635.1903979	650.2636973	229.2112698	profile #3
Ppp1r1a	1.906083197	2.019606111	0.924698716	profile #3
Plaat1	8.250029491	7.849425074	3.773089671	profile #3
Gpt	27.91894817	28.13151672	10.45081945	profile #3
Cyc1	755.8653112	700.9701679	332.8191596	profile #3
Oplah	10.07260791	10.95160976	4.814971708	profile #3
Lynx1	71.55148558	72.02865108	31.35613117	profile #3
Lypd2	1.976808619	2.327144928	0.435020622	profile #3
Sidt1	0.209095049	0.157092146	0.060846261	profile #3
Cmss1	50.81351006	44.78383742	20.96238081	profile #3
Ndufb4	217.5771231	186.9692653	97.91868024	profile #3
Atp5j	281.3748053	258.5543945	135.9606225	profile #3
Atp5o	454.6824886	404.6785106	213.2216296	profile #3
Adcy6	35.399256	41.78820655	18.50713689	profile #3
Gpd1	25.62692888	27.99492579	13.69514262	profile #3
Slc4a8	0.38894326	0.529142713	0.183931317	profile #3
Rgn	0.185381244	0.192386557	0.078774736	profile #3
Ndufa5	910.3833701	839.0433342	360.6828229	profile #3
Msrb2	37.45866603	33.39298547	16.55128991	profile #3
Lrrc69	0.132525664	0.223911009	0.065257341	profile #3
Tmem52	1.287414254	2.48126162	0.196984669	profile #3
Cpn2	0.605951403	0.751687461	0.345273986	profile #3
Gabrr2	0.74805452	0.776540948	0.243804174	profile #3
Ache	0.856135849	0.825029265	0.384535549	profile #3
Wfdc1	14.60700245	14.60021509	6.836101131	profile #3
Gnb3	5.6916791	4.43019758	1.004472128	profile #3
Prkn	0.633207955	0.725832278	0.254038109	profile #3
Slc22a3	1.546785718	1.891177158	0.433902317	profile #3
Slc22a1	0.719569824	0.737791788	0.34289558	profile #3
Cpne5	0.03901822	0.043452198	0.007598308	profile #3
Ndufv3	102.7892517	91.84494519	46.16804844	profile #3
Vit	1.236066439	1.201587429	0.569778155	profile #3
Lhcgr	0.05438847	0.102103303	0.017854388	profile #3
Nrxn1	0.096476011	0.070298224	0.028580971	profile #3
Lrpprc	19.63809	20.35375639	8.921591944	profile #3
Slc3a1	0.036894874	0.038289083	0.018290854	profile #3
Eci1	338.437455	322.5519159	99.98013753	profile #3
Epas1	95.97848379	104.1642882	43.69371254	profile #3
Myo7b	0.132820899	0.113401264	0.025837375	profile #3
Lims2	31.78482123	30.80619552	10.72216917	profile #3
Dele1	55.26571767	52.50261438	20.38307877	profile #3
Myot	11.00694135	11.14905161	4.718270294	profile #3
Camk2a	6.470676341	6.781254153	2.679354073	profile #3

Asrgl1	10.99363346	11.11099368	5.235189204	profile #3
Syt7	6.337565254	7.085820062	1.809934575	profile #3
Acy3	13.24955163	13.12874804	4.9973898	profile #3
Doc2g	60.49821387	57.29535633	17.01616955	profile #3
Slc29a2	4.51237286	3.817250952	2.014258106	profile #3
Vldlr	21.08044571	23.04832779	8.93563813	profile #3
Slc1a1	0.269006684	0.291423106	0.12994214	profile #3
Kcnk4	0.188916686	0.182392816	0.049874421	profile #3
Vegfb	157.2929638	154.5334227	69.04078233	profile #3
Gpam	21.67389794	25.95586138	11.09818411	profile #3
Sorbs1	11.3092702	17.13243467	6.029485275	profile #3
Ablim1	35.9733006	37.24725944	18.02792591	profile #3
Ppp1r27	0.300159455	0.286423196	0.110939859	profile #3
Myadml2	38.86871233	31.91241473	12.68215894	profile #3
Fn3k	4.885635289	4.357409018	1.668275208	profile #3
Got1	297.3545186	324.0480807	129.6194811	profile #3
Ndufb8	139.6389193	133.5994527	67.72137566	profile #3
Kcnip2	55.07280092	50.45598392	11.61849127	profile #3
Pitx3	0.400645846	0.347311587	0.138260158	profile #3
Fyco1	34.01009183	42.25766052	15.47985196	profile #3
Pfkfb1	2.435192673	2.482243974	1.03260766	profile #3
Atp5b	1375.182491	1315.924832	581.1871661	profile #3
Agap2	4.290924744	5.508726564	2.059776148	profile #3
Atp5a1	1524.766035	1474.600784	679.4881667	profile #3
Nnt	0.247278295	0.294986993	0.071957369	profile #3
Dpysl4	1.031413062	0.888795347	0.458433064	profile #3
Sirt3	6.094250072	6.714070452	3.228075134	profile #3
Cox8b	3058.636843	2725.157248	870.4944039	profile #3
Eps8l2	0.533688759	0.591439604	0.258973593	profile #3
Pnpla2	117.9184173	115.6268792	44.45024284	profile #3
Phkg1	2.046509164	2.110273172	0.60925733	profile #3
Clybl	55.95989737	54.65859304	27.96005798	profile #3
Uqcrc1	244.765438	239.34966	94.14085506	profile #3
Nmb	4.669062561	6.458770803	2.75907707	profile #3
A930017K11Ri	0.09261453	0.246399118	0.017952837	profile #3
Mcrip2	242.0298248	258.0706694	102.839371	profile #3
Hadha	164.2599205	170.6804503	67.18651827	profile #3
Agbl1	0.01845846	0.021729727	0.008527879	profile #3
D3Ertd751e	3.155266956	3.093205858	1.466348048	profile #3
Atp5c1	544.1592653	513.7161069	253.3589286	profile #3
Homer2	6.079748129	7.330593311	2.874849293	profile #3
Dhtkd1	0.44352497	0.542033675	0.25191518	profile #3
Rp1	0.048396988	0.137202988	0.014810252	profile #3
Adhfe1	19.24127695	19.88658983	5.305870652	profile #3
Gsta3	2.302298728	4.239572738	1.007342595	profile #3
Gm4956	1.447066415	2.07570628	0.133574366	profile #3
Slco5a1	1.183803119	1.015320847	0.327631425	profile #3
Ndufs1	61.33733526	62.80856878	30.33007246	profile #3
Acadl	460.3989143	465.5651198	195.1738838	profile #3
Ctla4	0.56622547	0.581752195	0.25432158	profile #3
Osgepl1	6.472603073	5.546852884	2.684362352	profile #3
Mstn	0.119775274	0.114293973	0.046798891	profile #3
Cnga3	0.701813313	0.633413023	0.276465519	profile #3
Ndufa10	149.7268022	142.841561	56.96755234	profile #3
Epb41l5	0.337397596	0.427957943	0.176052879	profile #3
Cacna1s	1.622702721	1.522316648	0.340997003	profile #3
Tnnt2	1138.432492	1066.733492	564.3390965	profile #3
Coq8a	104.1884817	101.7365455	43.62737404	profile #3

Efcab2	29.03518843	27.3027133	11.32144116	profile #3
Fh1	120.5418781	113.4369611	54.18267741	profile #3
Rgs7	0.367673753	0.346462513	0.136315093	profile #3
Mpc2	50.10349239	50.21377693	23.80903054	profile #3
Esrrg	3.852726988	4.752447353	2.297452659	profile #3
Spata17	0.229412968	0.265791658	0.127058543	profile #3
Lamb3	3.121709755	3.279785445	0.896202785	profile #3
Acbd7	0.885680951	1.472710359	0.085842283	profile #3
Meig1	3.244003539	2.472200084	0.892602776	profile #3
Phyh	205.1616059	202.6146043	74.2126265	profile #3
Mgst3	184.7586243	162.0711274	69.18363745	profile #3
Myoc	2.298226872	2.212047883	1.098897117	profile #3
Il2ra	0.584679698	0.591527223	0.179726068	profile #3
Ak1	263.161886	253.2507492	124.4414828	profile #3
Kcnj3	8.783679268	8.375958929	2.290038377	profile #3
Crat	83.99485732	87.56722674	37.02077517	profile #3
Gsn	447.6131633	483.0935942	202.4417996	profile #3
Grb14	21.48352137	20.95516188	10.35989119	profile #3
Ndufa8	557.5115706	536.4095693	266.822941	profile #3
Gpsm1	32.66777538	32.19564864	14.14972019	profile #3
Slc25a12	28.71510466	30.76973941	15.08026303	profile #3
Fastkd1	3.846456061	3.239633841	1.679805412	profile #3
Sord	71.68423713	73.78992712	32.15176733	profile #3
Gfra4	3.432187549	3.196252107	1.157978699	profile #3
Ivd	70.00281195	70.20099861	25.53599754	profile #3
Adra1d	2.973522768	3.372372955	1.53142573	profile #3
Gpcpd1	29.09534672	28.72122451	10.21207548	profile #3
Fahd2a	13.43704529	12.84901914	5.788057752	profile #3
Idh3b	235.985548	220.5866202	93.93513854	profile #3
Acss1	103.5398191	115.4447842	39.70279779	profile #3
Rbm38	47.70174494	55.49671555	23.43458429	profile #3
Chmp4c	2.801493933	3.413895825	1.626807859	profile #3
Sall4	0.292622937	0.301352293	0.137201229	profile #3
Car3	0.103981731	0.21836208	0.044782529	profile #3
Mfn1	53.90027259	57.9629266	25.69127279	profile #3
Ndufb5	65.27660108	58.19892271	31.83913488	profile #3
Tnik	1.134474316	1.418557178	0.699737927	profile #3
P2ry1	4.897041006	5.017786061	1.465940535	profile #3
Ppm1l	4.841323825	4.827856285	1.857375586	profile #3
Etfdh	72.17604788	75.39193585	29.87244007	profile #3
Hmgcs2	6.998178166	8.16206862	2.175201711	profile #3
Lrrc39	22.97982046	22.3956871	9.036451312	profile #3
Hadh	117.4049815	118.5348083	38.02642396	profile #3
Ppa2	6.913136601	6.940656454	3.330166652	profile #3
Myoz2	258.6317394	256.0090806	101.011458	profile #3
Rorc	6.726073666	8.680610663	3.086631872	profile #3
Cth	0.548941787	0.49056258	0.199202832	profile #3
Decr1	120.4445282	125.6449732	54.01085502	profile #3
Aldob	1.435269678	1.698709878	0.17150513	profile #3
Hsdl2	79.92421664	87.62803861	39.13224502	profile #3
Alad	19.90297023	21.34643691	10.50024094	profile #3
2310002L09Rik	16.10515182	18.74720781	9.32714048	profile #3
Spink4	0.194065359	0.327886457	0.088463313	profile #3
Tal2	0.071622748	0.122833264	0.028789846	profile #3
Aqp7	11.81337198	10.44275499	5.356691343	profile #3
Hint2	45.80370917	39.73326397	20.39361813	profile #3
Sh3gl2	0.10597906	0.148322707	0.069580602	profile #3
Prkaa2	11.43725413	13.75686124	6.837189119	profile #3

Bend5	3.715959797	3.818667527	1.663824716	profile #3
Angptl3	0.459787164	0.407751538	0.131407953	profile #3
Cpt2	75.66502095	73.06602067	29.68279675	profile #3
Cyp4b1	12.90397102	11.52058389	4.546102303	profile #3
Aldh4a1	16.33986516	15.13977462	5.322728885	profile #3
Pla2g2c	0.064070581	0.120680598	0.023364895	profile #3
Pink1	250.712514	278.847376	97.79806122	profile #3
Fabp3	4254.326909	4126.626761	1878.620194	profile #3
Hpca	0.047520111	0.060460584	0.029272632	profile #3
Rhd	4.994321918	4.448065716	1.944537744	profile #3
Extl1	7.30748402	7.170974437	3.071778839	profile #3
Per3	4.81039189	4.196564789	0.538597543	profile #3
Masp2	0.041987833	0.031529451	0.013783566	profile #3
Slc25a33	9.494974953	10.55387792	3.550922685	profile #3
Mfn2	137.2588743	145.4925061	64.50318739	profile #3
Prom1	0.249076937	0.235415468	0.082588053	profile #3
Ablim2	3.03440892	3.092378278	1.507118606	profile #3
Stk32b	0.066923927	0.076633528	0.012367645	profile #3
Rnf32	0.055648169	0.052260265	0.026967758	profile #3
Plb1	0.05954278	0.07944116	0.039092841	profile #3
Ociad2	1.865982042	1.685447212	0.852621241	profile #3
Yipf7	63.95954882	55.73884369	22.24892551	profile #3
Ppargc1a	10.30914086	13.68811762	4.466950101	profile #3
Crybb1	2.244054771	1.659044572	0.323934789	profile #3
Tesc	78.10814064	72.60405688	24.63905536	profile #3
Rilpl1	77.91145022	91.43514552	36.40287881	profile #3
Nipsnap2	310.9482776	312.727849	113.7898774	profile #3
Atp2a2	1557.299633	1749.837107	598.9323289	profile #3
Pxmp2	50.04739492	44.81579591	13.06691236	profile #3
Ndufa4	626.5344593	552.0686724	265.9019736	profile #3
Flt1	19.10141246	22.63175421	7.92702836	profile #3
Lmod2	237.6067109	268.1626692	129.7110351	profile #3
Asb15	9.643909043	9.900464863	3.128851865	profile #3
Cyp3a13	0.779585002	0.864196004	0.262144212	profile #3
Hibadh	22.82399207	22.24594009	10.47420065	profile #3
Clcn1	2.469757669	1.957446854	0.543652317	profile #3
Gstk1	58.76157702	53.02118201	16.91901511	profile #3
Klf15	17.81011132	16.69495233	5.128207546	profile #3
Tuba8	86.16746323	81.64666368	38.41176344	profile #3
Ldhb	799.2003176	776.9818123	258.0187753	profile #3
Kcnj8	30.61587451	27.23323511	13.92765984	profile #3
Abcc9	38.2632757	41.22308136	13.29460013	profile #3
Tmtc1	16.62043526	22.48998408	8.430814956	profile #3
Timp4	12.80668509	17.79696895	1.322457612	profile #3
Fkbp4	163.660316	156.6816287	69.65359574	profile #3
Ckm	364.7906398	349.2956532	93.68381231	profile #3
Rtn2	21.01717999	19.47774279	6.054436581	profile #3
Sbk2	2.258412953	2.666362453	0.521436634	profile #3
Tarsl2	5.498920686	6.342253408	2.873226991	profile #3
Apba2	0.244843413	0.277195336	0.088278243	profile #3
Idh2	782.985663	766.5180858	233.3712692	profile #3
Me3	4.884987094	4.76634893	2.119524866	profile #3
Tmc5	0.228500601	0.247015978	0.056640269	profile #3
Sox6os	2.975437801	2.482568891	1.085291724	profile #3
Stard10	12.42329549	16.33041966	5.784932407	profile #3
Sult1a1	24.90829521	27.75423665	7.773482994	profile #3
Klk7	0.073273754	0.069920512	0.024053959	profile #3
Myh14	15.60973403	17.0888669	6.010364371	profile #3

Dgat2	168.0894606	180.9635689	31.66953769	profile #3
Cox6a2	2481.583311	2224.924379	719.3951956	profile #3
Lyve1	9.183592102	13.34649272	5.590423558	profile #3
Tacc2	16.67482788	19.52744124	6.549786271	profile #3
Ndufab1	37.96640766	34.84938091	15.80114111	profile #3
Vwa3a	0.809226711	0.969597343	0.39224145	profile #3
Zp2	0.08632463	0.114832482	0.030981645	profile #3
Acsm5	1.053033501	1.462938554	0.177718348	profile #3
D7Ertd443e	0.149700706	0.207180565	0.100308665	profile #3
Art1	56.52265116	55.67053111	25.24954958	profile #3
Igsf1	3.711302559	3.344237219	1.612132367	profile #3
Fgf13	10.67397432	11.77845823	3.686049275	profile #3
Magix	13.16234131	12.79162816	3.712369012	profile #3
Fgf16	11.7216778	11.26181067	5.680463068	profile #3
Cox7b	620.4193581	568.3066081	301.728979	profile #3
Glt28d2	1.473923557	2.45312469	0.579205958	profile #3
Rs1	0.036436847	0.055980092	0.014646341	profile #3
Itgb1bp2	70.8988527	66.06555606	33.59759128	profile #3
Asb11	29.94898871	31.84645252	15.24211602	profile #3
Mcf2l	8.624561199	9.103989183	2.804237316	profile #3
Myom2	140.4355645	139.553854	46.75102813	profile #3
Ank1	22.24753391	20.48435919	10.0114491	profile #3
Nr3c2	3.835597728	3.215036257	0.971632813	profile #3
Slc25a4	2384.461649	2418.589725	864.62927	profile #3
Cbln1	0.182172236	0.25117618	0.120071788	profile #3
Got2	161.3490039	154.7483381	59.08495531	profile #3
Mylk3	125.3209182	125.094693	56.36320542	profile #3
Gpt2	6.546260789	8.94599684	3.093744937	profile #3
Tecr	197.5417376	185.636194	73.27279463	profile #3
Il15	12.01638795	11.48284187	5.281977061	profile #3
Slc12a3	0.011482571	0.032821083	0.003846328	profile #3
Nudt7	9.015831649	7.952933016	4.275871667	profile #3
Herpud1	66.03549833	90.03672041	25.82903887	profile #3
Coq9	152.5577466	143.674713	57.86185758	profile #3
Tmem38a	218.5012857	218.131161	89.05776209	profile #3
Cox4i1	1326.388078	1233.627395	612.7431042	profile #3
Rab3a	17.92332204	15.9594398	6.63461356	profile #3
Pde4c	1.986355478	1.832370885	0.268993979	profile #3
Car7	2.211577337	1.791790855	0.846956564	profile #3
Ldhd	12.56147039	11.98650239	3.524336551	profile #3
Egln1	98.3121381	101.7732824	44.5803053	profile #3
Usp2	23.42088534	24.22746091	5.515660681	profile #3
Kcnj5	19.08683544	18.80605548	7.277518946	profile #3
Acat1	162.9058643	161.4814042	73.05313574	profile #3
Drd2	1.386860488	2.032309594	0.644272119	profile #3
Bckdhb	42.10487175	39.8586407	15.63180845	profile #3
Idh3a	93.9480481	102.367781	50.67467054	profile #3
Tmem266	0.686131948	0.630385015	0.280416911	profile #3
Etfa	396.8468619	399.4717625	158.0490372	profile #3
Cyp1a1	82.39893163	46.52211175	3.682868944	profile #3
Cox7a2	404.7940239	347.0622636	199.7652484	profile #3
Hcn4	2.690483896	3.084748073	1.227699584	profile #3
Gsta4	54.65676072	48.09830154	23.47522005	profile #3
Nmnat3	3.829300447	3.979849649	1.981673676	profile #3
Arpp21	0.077017045	0.068781293	0.029447314	profile #3
Hhatl	31.79302955	35.1928966	16.16650553	profile #3
Slc25a20	39.21964689	40.56417117	15.55480249	profile #3
Pygm	307.7095897	305.399947	103.0770823	profile #3

6820408C15Rik	2.175660544	2.0266645	0.920919425	profile #3
Ank2	3.521193517	4.43759081	1.90467548	profile #3
Ccdc85a	4.71919626	6.386427843	1.843952831	profile #3
Slc16a1	50.39802763	54.82388474	25.19807317	profile #3
Slc16a8	1.874936552	1.516234952	0.710420525	profile #3
Klra9	1.728220445	1.467413668	0.56844751	profile #3
Dhrs7c	72.07770418	71.06164073	16.78068003	profile #3
Pfkm	359.5070002	354.1652761	130.3649336	profile #3
Tnxb	12.1464346	11.26499685	5.484505595	profile #3
Agl	14.09402069	16.15618279	7.843540975	profile #3
Upb1	0.069242725	0.056155923	0.031063752	profile #3
Myo5c	0.724152737	0.7473249	0.286673767	profile #3
Pank1	12.50897201	17.78363605	6.469420441	profile #3
Pdpr	11.22359231	16.05733554	7.689270746	profile #3
Clec18a	0.291255385	0.432557187	0.058537251	profile #3
Lrrc24	20.7569315	19.84774746	8.738928762	profile #3
Klf9	37.28899764	52.47727648	15.86927329	profile #3
Kcnk1	0.133292017	0.158090226	0.076541057	profile #3
Rasl10a	2.530001345	3.532066258	1.709737706	profile #3
Hdac11	5.229941818	4.445053005	1.954032645	profile #3
Flvcr2	1.060314789	0.98315743	0.455302179	profile #3
Fhod3	58.27399068	66.95203646	25.87601977	profile #3
Dhrs11	58.03619582	53.89017448	28.15123512	profile #3
Rasd2	2.157391732	1.526879344	0.457836502	profile #3
Scn10a	0.277809229	0.458278737	0.161061947	profile #3
Atp5h	723.4296903	624.3262533	332.0457473	profile #3
Pla2g3	0.11781454	0.122119551	0.036428719	profile #3
Asb16	0.613748756	0.495116624	0.112232067	profile #3
Art3	84.29398137	98.58153104	34.68481817	profile #3
Gipc3	5.499573357	4.209185698	1.929485104	profile #3
Filip1	12.65934696	15.86595693	7.492880683	profile #3
Cobll1	5.942315243	8.90268234	2.949035146	profile #3
Pygo1	2.231135462	3.34522775	1.415492255	profile #3
Mdga2	0.050484935	0.048174582	0.01399454	profile #3
Arl4d	16.17616209	25.53473156	6.233752321	profile #3
Wnk4	3.469401037	3.389035869	1.637356705	profile #3
Tnni3	1910.936076	1785.323725	577.8097349	profile #3
Plk5	0.667336454	0.529002814	0.110298358	profile #3
Ky	3.558536108	2.706291655	1.213011236	profile #3
Ndufa3	484.9640186	461.595847	193.0332568	profile #3
Thrsp	13.86204793	13.21563693	4.143841129	profile #3
Pim3	40.09517235	56.91845938	17.29079565	profile #3
Ppp1r26	0.568049078	0.556689233	0.275813594	profile #3
Krt222	8.842598433	7.823259317	4.012047722	profile #3
Aldh5a1	6.212806395	6.268629328	2.357006854	profile #3
Prickle1	8.002319805	7.895083299	3.602494249	profile #3
Ndufa13	395.475223	359.6335351	190.7458764	profile #3
Pdzrn4	0.393883237	0.475589988	0.229056545	profile #3
Macrod1	264.7362853	284.4544465	102.1486112	profile #3
Npy1r	0.394863954	0.537055931	0.145166409	profile #3
Rnf39	0.995791616	0.984210737	0.423145407	profile #3
Fgf1	41.51442473	43.85476578	19.49160062	profile #3
Cox6b1	908.95609	823.0241841	452.7965837	profile #3
Syde2	4.225249297	5.85967772	2.805793316	profile #3
Acaa2	600.1109982	627.5344239	187.5942864	profile #3
Sstr4	1.524206518	1.488756972	0.384120105	profile #3
Apbb1	23.0162924	24.71868163	10.97159025	profile #3
Cfap61	0.084265791	0.112435823	0.043764568	profile #3

Ndufc1	152.2565533	137.7671944	65.85797006	profile #3
Slc2a12	1.446397478	1.646990838	0.656246731	profile #3
Gdf7	0.239259238	0.219819874	0.090162998	profile #3
Cdkn1c	17.09283706	16.18479071	5.306586979	profile #3
Abhd18	5.050305782	5.69037505	2.422252701	profile #3
Ppm1k	12.18372641	11.60071215	4.859482905	profile #3
Vstm2l	0.12489578	0.239856008	0.033442592	profile #3
Ankrd9	21.31217436	28.32994216	8.409637077	profile #3
Ndufv1	319.9717661	313.0047329	129.423944	profile #3
6430571L13Rik	2.257009443	2.244723768	1.08680461	profile #3
Wnk2	9.183064914	12.9098746	1.660761231	profile #3
Acer2	2.864217224	3.348848657	1.644498267	profile #3
Tigar	19.1267746	17.50149988	8.946981203	profile #3
Mturn	4.855739982	5.521776518	2.321133135	profile #3
Rbm24	52.57911079	57.19752259	23.59413639	profile #3
Snrk	32.23728495	36.99895917	16.93720428	profile #3
Pde4dip	74.69214207	86.46218637	41.89662568	profile #3
Kcna7	3.499980303	3.894515726	1.274297616	profile #3
Asb10	35.74437	33.51047278	11.76106584	profile #3
Pnmt	1.181854503	1.492627224	0.219395149	profile #3
Hrc	360.0864698	353.1536483	81.80291208	profile #3
Pdss2	3.756790306	3.514648093	1.670047304	profile #3
Kcnh2	31.00734288	35.67059512	13.69987234	profile #3
Pcp4l1	140.0864544	142.4137178	45.72677447	profile #3
Hjv	94.53193468	95.73754648	46.32397546	profile #3
Uqcrfs1	633.3994432	612.7893362	285.332192	profile #3
Mfsd4b1	1.731023927	1.83395342	0.835145702	profile #3
Car14	39.47749563	46.14639238	13.24627784	profile #3
Ciart	5.404303952	5.542714139	0.705607623	profile #3
Pptc7	26.05440695	31.54466537	14.40546038	profile #3
Pln	549.1168608	506.836465	166.3562061	profile #3
Fsd2	58.87853608	64.03111127	25.30186759	profile #3
Atp5j2	230.9329338	201.8350968	94.44598581	profile #3
Aspdh	0.260560634	0.212791619	0.031008335	profile #3
Ptpn3	7.561466242	7.879875338	2.694376336	profile #3
Scgb1c1	21.70599623	18.56160424	3.806869433	profile #3
Pdk2	111.8571359	106.1139263	32.33846805	profile #3
Bmp6	28.89595162	27.00948707	12.48252451	profile #3
Echdc3	9.082260613	8.027274092	3.038816828	profile #3
Wdr93	0.061745722	0.071536923	0.01871655	profile #3
Whrn	7.172628672	8.800785048	2.391872316	profile #3
Shd	0.116143337	0.169940865	0.065804289	profile #3
Ptchd3	0.160710375	0.157659982	0.048839121	profile #3
Atp6v0e2	4.057421158	3.524167425	1.722974682	profile #3
Cdh6	0.140682638	0.269124152	0.051955455	profile #3
Mreg	11.05732339	10.09112242	3.251607731	profile #3
Ppl	2.006888342	2.033472364	0.336123947	profile #3
Cntn5	0.272387744	0.264559783	0.130781126	profile #3
Cdnf	8.495231475	8.395727194	3.216827982	profile #3
Kyat1	9.785793839	9.339080472	4.813700594	profile #3
Cpeb3	10.32097518	10.19340046	2.976669972	profile #3
Oxld1	25.85763512	22.57213929	10.09201161	profile #3
Dnajc28	11.67304813	11.58098559	4.222948708	profile #3
Txlnb	83.06670791	82.89903735	29.10175055	profile #3
Coq10a	68.21265361	65.43557158	26.78599521	profile #3
Etfbkmt	7.127140635	7.184127194	3.229017252	profile #3
Magi2	0.567770816	0.576016533	0.253056213	profile #3
Ndufb10	485.0659853	471.3128187	206.0752283	profile #3

Tnni3k	32.11239338	32.44061137	12.61404373	profile #3
Mrps35	8.841028707	8.861140055	4.224979949	profile #3
Mettl11b	0.2755035	0.286795174	0.098662884	profile #3
Abcc8	3.998054394	4.176432569	1.876253406	profile #3
Maob	13.30951581	16.01014265	6.520941965	profile #3
Fmo2	8.452464951	14.35767773	3.340043813	profile #3
Fmo1	8.407745103	8.626001789	4.172255568	profile #3
Kyat3	2.815589037	2.612722482	1.263515979	profile #3
Trim7	3.848559794	4.021389071	1.554008606	profile #3
Slc26a10	12.98772894	10.38630112	2.682640632	profile #3
Lrrc52	0.699478735	0.888738852	0.110753282	profile #3
Lrfn2	0.186075989	0.262074541	0.079069957	profile #3
Cblc	0.182151074	0.171545974	0.089693531	profile #3
Abcb1a	8.856680214	7.414806623	3.660195442	profile #3
Ppip5k2	7.946363104	11.46881485	4.358222452	profile #3
Kcna2	0.256575072	0.32840971	0.12759046	profile #3
Slc25a34	69.99893872	75.24839407	19.01821318	profile #3
Myh6	3429.870701	3579.995071	1150.766945	profile #3
C1qtnf4	5.796678839	5.617533792	2.33211524	profile #3
Kcnd3	1.472493934	1.207232311	0.579926049	profile #3
Cmtm8	4.162283802	3.532277642	2.034786255	profile #3
Ramp3	5.623111343	5.728837518	1.895807261	profile #3
Cyyr1	5.874741466	5.709294366	2.808705216	profile #3
Pla2g5	11.51020277	11.88390101	4.709955347	profile #3
Atp1b2	6.45926729	5.375367886	3.180157146	profile #3
Myzap	32.46056827	32.82774606	12.85362894	profile #3
Tssk1	0.152528622	0.147262946	0.045618379	profile #3
Pcca	16.61486638	15.95417884	8.228820025	profile #3
Coq5	41.40289119	39.7609953	19.41076801	profile #3
Slc24a4	0.040941697	0.046115831	0.018662981	profile #3
Myrip	0.845476984	1.230072464	0.255201341	profile #3
Gck	5.380577481	5.479269175	2.242005665	profile #3
Acacb	33.46954366	38.69150361	11.36716885	profile #3
Csdc2	12.10583898	16.34627031	5.689154153	profile #3
Tmc7	1.525953795	1.578930662	0.585764363	profile #3
S100a14	0.080401716	0.060375155	0.024763935	profile #3
Osbpl6	1.577484087	1.419527119	0.645993439	profile #3
Ccno	0.092034557	0.138290276	0.050216026	profile #3
Frmpd3	0.157952475	0.193163999	0.061715582	profile #3
Kcnj12	10.72119366	15.65901652	5.100974371	profile #3
Cux2	1.549183986	1.458781008	0.515411305	profile #3
Acad12	15.76316362	15.51376959	4.218247116	profile #3
Jph1	5.579949478	6.216853189	2.421598703	profile #3
Ppp1r3a	17.46140659	20.05038391	7.742111215	profile #3
Hecw2	1.765383026	2.356753095	0.86875755	profile #3
Egflam	3.733666593	3.475028632	1.690438049	profile #3
Sbk1	23.25751768	26.12163286	9.392510494	profile #3
Tmem82	3.417339255	4.655516372	1.027913485	profile #3
A530016L24Ril	11.90535888	12.98010982	3.012133142	profile #3
Ppp2r3a	17.2932346	16.10630646	8.516250613	profile #3
Zfp536	0.27040268	0.282688322	0.114441854	profile #3
Elfn2	0.171919642	0.145824042	0.028218481	profile #3
Rai2	9.705332428	9.833147902	3.888623282	profile #3
Casc1	0.511871826	0.427082385	0.216578245	profile #3
Ubxn10	0.412179303	0.532242578	0.119848166	profile #3
Rbm20	29.10436304	28.89249506	12.4394571	profile #3
Fem1a	43.17261931	50.87955092	23.40150546	profile #3
Ccrl2	5.540610805	6.766069985	3.341710958	profile #3

Ccdc141	13.01046382	14.19191858	6.372256505	profile #3
Gpr22	8.634351366	8.643735956	2.793611545	profile #3
S100a1	729.5018354	615.1410894	328.402395	profile #3
Lmod3	48.74503252	44.67279045	16.83353611	profile #3
Wfikkn2	1.818758677	1.927305538	0.9051875	profile #3
Ackr3	19.81057784	26.16952273	12.77065495	profile #3
Sowaha	0.152446219	0.132465048	0.023104979	profile #3
Dsg2	8.04529805	8.755300285	3.342892503	profile #3
Hs3st5	0.411853906	0.427417324	0.157667523	profile #3
Lingo4	0.038094311	0.044845506	0.01157668	profile #3
A730020M07Ri	0.602888195	0.567787166	0.17508019	profile #3
Zfp612	2.133858664	2.258058208	0.681897907	profile #3
Tmem201	12.59578345	13.42178328	5.821902773	profile #3
Klk14	0.368324631	0.428696222	0.071726597	profile #3
Uqcrq	568.1573163	508.2702954	247.1797839	profile #3
Mylk4	30.48118128	33.95107393	4.908391477	profile #3
Shld1	9.907996075	9.909809636	2.383818253	profile #3
9030612E09Rik	2.220628822	1.876896229	0.76011287	profile #3
Poln	0.204040966	0.201883253	0.086749119	profile #3
Dcaf12l1	1.061753506	1.210700547	0.516366496	profile #3
Adra2c	0.286682969	0.243588586	0.085741365	profile #3
Proser2	0.752435193	0.797680705	0.386088303	profile #3
Kcna5	18.11178636	16.46597116	7.652076579	profile #3
Penk	2.832594125	3.768092873	0.41844173	profile #3
Lrtm1	24.64925167	29.31264153	10.60904555	profile #3
Adra1a	1.272651218	1.904058594	0.681101841	profile #3
Teddm2	0.277648029	0.26112686	0.113348645	profile #3
Smco1	11.54633984	10.55268097	2.104294323	profile #3
Scn4b	7.088546899	5.588720417	1.228134293	profile #3
Tcaim	10.02688607	10.9651355	4.369301508	profile #3
Hrk	0.244590037	0.229584053	0.032117141	profile #3
Mapk10	1.28032224	1.428648135	0.690013842	profile #3
Gchfr	5.278541011	5.657947705	1.700964354	profile #3
Lrrc4b	10.5400547	10.30575457	3.548225863	profile #3
1700113H08Rik	0.149305123	0.210918541	0.029407905	profile #3
Dusp18	8.112893607	10.78416725	3.296643904	profile #3
Kcnv2	1.05881181	1.530435172	0.099131559	profile #3
Odf3b	1.692795327	1.415935348	0.745811637	profile #3
Cmya5	37.03464464	37.65516569	13.04767934	profile #3
Zfp30	2.17762614	2.063717299	0.892446206	profile #3
Mafa	0.740281662	0.958572692	0.464263682	profile #3
Gal3st3	5.343950441	5.125105046	1.742957469	profile #3
Cyb5r2	2.302934897	2.023499285	1.095411042	profile #3
Bcl11b	0.435499157	0.4113639	0.09490959	profile #3
Pou4f1	0.039627216	0.037813747	0.018063784	profile #3
Mlf1	77.34260156	65.0035694	21.74900657	profile #3
Inka2	7.706030549	7.382280259	2.936484783	profile #3
Spata33	2.561604313	2.367911504	1.027533926	profile #3
Fitm2	47.26464987	49.95469453	16.76392047	profile #3
Ggnbp1	3.438739868	3.575046629	1.588780799	profile #3
Rimkla	0.101795798	0.087767587	0.043850171	profile #3
Yjefn3	18.09306622	15.97468271	8.080490202	profile #3
Gpr137c	0.543690442	0.566827363	0.275272631	profile #3
Ntf3	3.054062508	3.687587878	0.983980945	profile #3
Agtr1a	10.5860941	9.955226004	4.270268443	profile #3
Apoo-ps	11.07524381	12.13447022	3.672435182	profile #3
Lrp1b	0.012530818	0.018335085	0.007669261	profile #3
Chchd10	164.1531995	158.4103585	64.30715231	profile #3

Bex1	0.897593191	0.595837529	0.180300656	profile #3
Nudt6	10.58245962	10.42958858	4.88146575	profile #3
Pla2g4e	1.236555676	1.385668147	0.510835587	profile #3
Abca12	0.196915792	0.19363635	0.082638642	profile #3
Ppp1r3g	0.1222003	0.242705077	0.050759747	profile #3
Adra1b	10.74317019	12.70206153	6.132443777	profile #3
Fam228b	0.445044115	0.412543828	0.220259068	profile #3
Kcnb1	6.631201607	7.635322361	2.813180446	profile #3
Tmem150c	1.7497324	1.500730036	0.77953447	profile #3
Gp1ba	0.692121538	0.613791867	0.279450333	profile #3
2310061I04Rik	28.61309797	26.95242978	12.91560093	profile #3
Fam131a	11.60543579	10.54236141	5.400210767	profile #3
Atp5k	485.1362655	437.552767	155.3940423	profile #3
Fam160a1	6.801075379	8.775646684	3.541676398	profile #3
Sv2c	0.042944463	0.037622436	0.017400825	profile #3
Klhdc1	8.316289201	8.363046019	3.699131364	profile #3
Sec31b	3.006012176	2.903662855	1.303998614	profile #3
Foxo6	3.079739317	2.542045645	1.129556414	profile #3
Gpr17	0.41643201	0.531876646	0.138084505	profile #3
Pkdrej	0.786929805	0.672171883	0.381036114	profile #3
Suclg1	71.21631216	66.17072433	30.61524036	profile #3
Fbxo31	24.56298095	26.90915101	9.703482046	profile #3
Cntn2	1.065785597	1.452369721	0.56998572	profile #3
Gpr21	0.125406002	0.139656969	0.048842489	profile #3
Cdh22	0.586296619	0.79479029	0.381699034	profile #3
Arhgap20	1.549735692	2.328112819	0.671958539	profile #3
Gatd3a	128.7685822	127.6012902	55.39497317	profile #3
Cbx7	5.772195422	5.064873714	1.98855826	profile #3
Ebf4	0.314666544	0.420946306	0.183117825	profile #3
Plxnb1	6.726260128	6.652622057	2.461593698	profile #3
Klk1b26	0.368469584	0.413761482	0.065775256	profile #3
Ech1	566.6011737	569.4332612	191.6785807	profile #3
Stum	0.50204293	0.671555924	0.265767709	profile #3
Lgals4	13.95321892	15.03376587	4.016751401	profile #3
Tmem179	1.599648248	1.981882728	0.641651065	profile #3
Klk13	0.06045005	0.047442133	0.01861877	profile #3
Gzmm	5.40825349	4.950723763	1.455411592	profile #3
Fgfr3	1.933374101	2.725125701	0.937318113	profile #3
Kcnn2	2.344737789	2.164829914	0.8467677	profile #3
Pm20d2	9.537314813	14.71412493	5.94010814	profile #3
Dsp	86.19600053	92.10744389	45.20943971	profile #3
Lmntd1	0.107513561	0.168195724	0.0588234	profile #3
M5C1000I18Ril	0.0914113	0.107611501	0.02777946	profile #3
Pld5	0.021080957	0.026253095	0.008473809	profile #3
Tns1	58.34864708	70.84012774	35.20194481	profile #3
Epm2a	9.856772621	10.80110061	4.505977218	profile #3
Gucy2g	0.173000963	0.107723065	0.011590058	profile #3
Hmcn2	2.945880267	2.444181919	0.907504559	profile #3
Per2	6.858491865	8.169371669	0.547737136	profile #3
Nanos3	0.506645677	0.507798182	0.092569947	profile #3
Sertm1	0.131653787	0.137376227	0.026250078	profile #3
Tcim	36.94629128	53.64205766	17.08920069	profile #3
D830013O20Ri	5.508470561	6.355952864	1.918556343	profile #3
Actr3b	20.11205752	20.27103772	5.749338098	profile #3
Cyct	0.211990479	0.238781435	0.069591225	profile #3
Adgrf5	44.72157458	47.87722352	16.20031611	profile #3
Cngb3	0.953611945	1.411947613	0.161330927	profile #3
Fry	2.32229603	3.26788112	1.395415121	profile #3

Them6	14.05089772	13.49965365	6.873108689	profile #3
Retsat	13.83714234	15.77136641	7.315302783	profile #3
Immp2l	11.52061798	9.695882366	5.021258772	profile #3
Usp13	29.73160672	31.37393526	15.68158033	profile #3
Ces1d	32.15670918	39.31914625	5.361459307	profile #3
Sh3rf2	4.009632269	3.664209934	1.629995302	profile #3
Gstm1	88.56773434	85.36449601	35.24656178	profile #3
T2	0.366092787	0.399244808	0.095360453	profile #3
Serpina9	0.050391495	0.059322036	0.022970604	profile #3
Spock2	2.476095798	4.992780762	0.930423647	profile #3
Adra2b	0.413495575	0.447952308	0.191781684	profile #3
Kcnt1	0.161290297	0.223708212	0.031516076	profile #3
Tlcd3b	3.269914985	3.243737237	1.320553183	profile #3
Vwa8	37.19869891	43.41419906	14.55089979	profile #3
Ddn	0.10241287	0.177138196	0.024375536	profile #3
Hopx	128.0115833	136.6624698	42.60557499	profile #3
Mrgprh	1.293920967	1.269572004	0.428357264	profile #3
Hadhb	201.9564188	206.6481211	77.33664032	profile #3
Myl3	2719.916909	2449.87544	793.8760293	profile #3
Dbp	38.70165349	28.05376334	3.243909043	profile #3
Gm16380	0.193659364	0.227980292	0.089471372	profile #3
Hydin	0.012830456	0.008671167	0.002527153	profile #3
Olfr1393	0.343738368	0.307735524	0.156292516	profile #3
Chpt1	36.22358343	38.88208815	17.29869479	profile #3
Lrrc10	121.9670201	136.2726953	48.55084449	profile #3
Nrg2	0.854807165	0.6937656	0.333143646	profile #3
Bckdha	45.01718486	42.41545427	16.08220596	profile #3
Eno3	681.1031002	620.3529913	245.3420832	profile #3
Intu	4.476301637	4.203780962	1.653113502	profile #3
Ctnna3	1.778412556	2.377300638	1.047435293	profile #3
Kcnd2	2.395552931	2.494650415	1.130276794	profile #3
Acyp2	71.33082509	67.53853119	31.63820301	profile #3
Irx1	4.99944214	5.495520965	1.531006952	profile #3
H4c8	39.12467335	32.3445827	13.96562627	profile #3
Lsamp	0.101370343	0.09994889	0.045018201	profile #3
Retnla	4.625895316	3.748762054	0.762245024	profile #3
Slc38a11	0.337559484	0.388591192	0.105538904	profile #3
Obscn	38.44128994	40.26456175	16.56374949	profile #3
Mrps36	55.25221651	49.02391535	27.37081876	profile #3
Cox5b	568.7800838	520.4324976	266.2391226	profile #3
Ppp1r1b	0.363372924	0.320495574	0.072057719	profile #3
Slc22a12	0.041804141	0.039259073	0.018754222	profile #3
Patj	1.26622975	1.326654765	0.627489271	profile #3
Ces1e	0.233302349	0.273805477	0.078149503	profile #3
Trim54	146.0670452	138.0315219	49.16915669	profile #3
Erbb4	0.592474039	0.701212391	0.239809503	profile #3
Opcml	0.11013213	0.12167906	0.034875956	profile #3
Cytl1	6.968986263	9.36864236	2.317638275	profile #3
4932435O22Rik	0.119153688	0.134212105	0.013038402	profile #3
Cys1	2.166678034	3.100902675	1.274906427	profile #3
Acadm	282.9475159	289.8755289	97.82769125	profile #3
Gm7117	2.152771535	2.559547771	1.17783598	profile #3
Lin28b	0.032863503	0.034105372	0.015085136	profile #3
Srrm4	1.441563628	1.419005244	0.685616161	profile #3
Cadm2	0.042467254	0.081047632	0.017440031	profile #3
Izumo1	0.12574775	0.104546685	0.057175556	profile #3
Tnnt1	8.322413841	8.257148282	2.978150793	profile #3
4430402I18Rik	1.978722454	2.193088533	0.605520696	profile #3

Paqr9	8.850404232	8.674204624	3.083489288	profile #3
2410137M14Ril	0.427057058	0.473407008	0.095793417	profile #3
ND1	31167.64074	30479.37801	12552.79188	profile #3
ND2	18059.5701	20078.80194	7366.791343	profile #3
COX1	65114.6563	67884.87328	27832.07625	profile #3
COX2	4225.709382	3572.769332	1451.462667	profile #3
ATP8	160.1809762	196.5815948	42.61069641	profile #3
ATP6	3476.258326	3780.934069	633.0316319	profile #3
ND3	567.7842133	539.1353697	69.96456663	profile #3
ND4	16556.2121	14782.91054	5612.231665	profile #3
ND5	9259.049129	11620.83889	4177.222522	profile #3
ND6	12840.62891	10826.99416	4254.173112	profile #3
CYTB	36347.60254	34263.789	14327.91031	profile #3
Gm25135	2.302151115	2.470850732	1.196288549	profile #3
Gm25788	1.059952397	0.795938118	0.347956125	profile #3
Mir208a	1.672936916	2.66062993	0.508397594	profile #3
ND4L	1097.629948	1757.903069	252.1399651	profile #3
Chrna10	0.372465544	0.373200615	0.138758759	profile #3
Zbtb16	7.185679977	25.22731611	1.93476972	profile #3
Ecsit	32.66216495	30.06648043	13.98587986	profile #3
Lgi1	0.481027073	0.503889857	0.111494628	profile #3
Gm10222	313.3965405	420.2634479	101.3905407	profile #3
Mkrn2os	0.401808688	0.450131033	0.082999607	profile #3
Tcf15	56.36795051	66.15954963	19.4198525	profile #3
Gm10250	117.6976918	101.8690432	57.7899374	profile #3
Celsr2	0.534686894	0.701881503	0.238235475	profile #3
Cry2	7.543216084	7.448885107	1.968980207	profile #3
Ptprz1	0.077677372	0.091495168	0.044798282	profile #3
Selenbp1	35.15327312	37.77589539	14.92001575	profile #3
H4c6	0.236749811	0.278707365	0.109379324	profile #3
Dsg1a	0.022454223	0.01916542	0.008016576	profile #3
Ank3	1.599079514	2.117681568	1.03487132	profile #3
Tmem100	8.067715397	11.74853279	5.391449211	profile #3
Pabpn1l	0.07884268	0.087399743	0.035848546	profile #3
Art5	6.307184522	6.010016909	2.389815984	profile #3
Ctcfl	0.092604184	0.13729082	0.04250754	profile #3
Trabd2b	17.82535643	20.65818104	7.921957123	profile #3
Armc2	3.291029247	3.225847223	1.122434429	profile #3
C1qtnf9	34.68619308	31.42479378	11.01830589	profile #3
Atp5md	1838.760451	1595.207038	837.3232527	profile #3
3425401B19Rik	23.00188634	18.46693797	8.940596834	profile #3
Klf12	1.647051618	2.260675323	0.875623919	profile #3
Mansc4	0.325947803	0.383713309	0.071333728	profile #3
Lrrc30	0.315756143	0.251088441	0.095956794	profile #3
Gm5532	43.77363533	36.91883337	19.03264521	profile #3
Prob1	21.17681636	23.02724856	10.36045058	profile #3
Gal3st2c	0.603256197	0.648188511	0.245192627	profile #3
Klhl21	26.28908862	24.83116653	11.4589797	profile #3
Tufm	101.6954077	98.84596296	46.52591612	profile #3
Mlycd	39.78823197	39.35993206	16.15651265	profile #3
Gm10619	0.03423451	0.032150287	0.008295223	profile #3
Svip	9.59681669	9.716211474	4.242214579	profile #3
Adh1	6.803981449	10.38396321	1.163541598	profile #3
Cox7a1	1924.523951	1683.585415	406.3210501	profile #3
Cib3	0.298758512	0.270542651	0.063705038	profile #3
Amy1	1.446646489	1.618784764	0.69797708	profile #3
Kcnd3os	0.649968346	0.616513782	0.235828357	profile #3
Gm15319	0.074591052	0.064814381	0.033915397	profile #3

Gm15417	16.9351856	16.93025711	6.500045582	profile #3
Myh7b	11.41900237	10.87446062	4.229026084	profile #3
Smim26	59.74740873	50.63670211	26.4291191	profile #3
BC025920	0.180222419	0.181294673	0.077617633	profile #3
Pde11a	0.114403863	0.135688039	0.03240937	profile #3
Fign	0.742856263	0.726582297	0.278851993	profile #3
Ighg2c	0.698108416	0.82565074	0.222759429	profile #3
Mir208b	127.1220063	104.7288169	47.95016164	profile #3
Ankrd63	0.744178181	0.990360789	0.196919831	profile #3
Gm6316	0.092215017	0.160256735	0.044688483	profile #3
Ppil6	0.44253742	0.758149781	0.18292265	profile #3
Perm1	69.62221939	69.65189502	25.71815883	profile #3
Fam174b	64.33057518	60.09971457	25.68368484	profile #3
Ceacam15	0.091786277	0.156685233	0.030745734	profile #3
Cacng6	0.570921499	0.661454202	0.13833764	profile #3
Zfp970	19.22131602	16.55691128	7.071688821	profile #3
Gm14412	0.208227457	0.241246698	0.106760279	profile #3
Gm14393	0.098793144	0.14140808	0.048647014	profile #3
Pdf	0.275995648	0.36714047	0.107000593	profile #3
Cpt1b	2.827587524	2.999402628	1.270679635	profile #3
Atp6ap1l	0.432232094	0.610944688	0.167059827	profile #3
Hsbp1l1	2.007143067	1.977075606	0.539689852	profile #3
Neu2	0.035740995	0.040257874	0.016292278	profile #3
Col4a3	0.390077796	0.333472929	0.165113175	profile #3
Apoo	86.37718155	74.02605022	39.54077481	profile #3
Tmem182	65.73335928	69.38817059	30.59127159	profile #3
Muc4	0.016000564	0.030052875	0.003589096	profile #3
Rhox2h	0.657339572	0.448960171	0.054342205	profile #3
Mir1b	7.20825551	8.9323426	2.615375139	profile #3
Gm12722	0.172650048	0.221043772	0.09245893	profile #3
Gm6222	0.149537865	0.165767716	0.067992553	profile #3
Gm12251	21.38908991	19.53837162	8.978322447	profile #3
D930015M05Ri	0.316165771	0.259802687	0.071877841	profile #3
Ccdc85c	12.27584837	13.61552981	6.757986698	profile #3
Gm5860	3.606630246	3.694099375	1.694721089	profile #3
C230035I16Rik	2.413335135	3.082500804	1.518539815	profile #3
Gm13715	0.572136965	0.859687724	0.308007302	profile #3
Prdm16os	2.143046989	2.356708171	0.796906502	profile #3
BB218582	8.071451688	7.107037808	2.433834988	profile #3
Sbk3	1.890293913	3.476824251	0.228125129	profile #3
Gm11732	0.755930217	0.750685968	0.235499582	profile #3
C530005A16Ri	0.521264688	0.449430575	0.210676432	profile #3
Gm12381	0.95395865	1.240148778	0.375334192	profile #3
Gm12167	1.38322525	1.172562815	0.438031145	profile #3
Wipf3	6.634394817	7.751073564	2.127257212	profile #3
Unc45bos	1.275838567	1.084739308	0.387721289	profile #3
Gm14169	0.033789787	0.043261048	0.016977894	profile #3
Gm15972	5.175512658	4.823037619	2.523744039	profile #3
A330102I10Rik	0.159242636	0.199397096	0.096540216	profile #3
Susd5	0.351413722	0.389417694	0.167458679	profile #3
Gm15743	0.268647471	0.325265858	0.071934117	profile #3
Gm15543	109.1713402	104.8993531	39.52145432	profile #3
4930429F24Rik	6.607726256	6.566854767	2.304178307	profile #3
4930481A15Rik	4.933915066	4.986560009	1.831429785	profile #3
4930522O17Rik	0.856877253	1.462746036	0.191352643	profile #3
Gm11264	0.389732132	0.245832015	0.061410916	profile #3
Kif28	0.377886558	0.410965796	0.118797861	profile #3
E130215H24Rik	0.317260552	0.44416568	0.0485434	profile #3

AW549542	0.994414124	0.87743978	0.135040466	profile #3
Gm22009	7.320513053	9.271634837	2.95255979	profile #3
Gm16299	0.353882664	0.413677906	0.122044831	profile #3
Gm614	0.119598419	0.114125211	0.05525493	profile #3
Acad11	10.17996399	10.63817495	4.789045148	profile #3
Pcp4	0.216620537	0.255010717	0.071111158	profile #3
Klhl33	3.467010338	3.905164547	1.365760372	profile #3
Otogl	0.0264091	0.024871523	0.004880442	profile #3
Gm6257	0.093534587	0.171870154	0.050090387	profile #3
Gm3646	18.80311388	16.87248464	6.925389175	profile #3
C2cd4b	0.244153818	0.471444884	0.064457145	profile #3
Ndufab1-ps	97.57649232	91.16409681	37.0112306	profile #3
Gm20619	1.32024058	1.24038537	0.381925675	profile #3
Gal3st2b	0.154027266	0.170744367	0.061422126	profile #3
Gm10406	0.207038416	0.233203539	0.104863321	profile #3
A830019P07Ril	0.105863047	0.088014541	0.051302479	profile #3
Plscr5	1.399030368	1.006482264	0.382722522	profile #3
Kcnj11	49.98885646	46.38162014	16.89654883	profile #3
Gm25596	1.758118075	2.063853123	0.788728978	profile #3
Lamtor3-ps	0.048777349	0.062449616	0.02450849	profile #3
9330179D12Ril	0.515510272	0.476672119	0.141981832	profile #3
4632428C04Rik	3.885619734	3.497050746	1.250281491	profile #3
Gm5421	5.446402242	6.02269866	1.762100667	profile #3
Gm16793	3.779168987	4.390356126	1.139556311	profile #3
1500026H17Rik	0.321102196	0.30640753	0.153342254	profile #3
D630044L22Ril	0.031231166	0.059603855	0.015378628	profile #3
9530026P05Rik	0.606917465	0.716275094	0.229893099	profile #3
B230110C06Ril	0.643666852	0.617620789	0.241767033	profile #3
4921504A21Ril	1.460718706	1.413490502	0.570835258	profile #3
Mhrt	13.84720229	13.82794962	6.045092784	profile #3
Gm2093	0.026675927	0.025051876	0.012129131	profile #3
1700101I11Rik	0.421219484	0.466700556	0.16026296	profile #3
F630040K05Ril	0.108259333	0.084723685	0.026231874	profile #3
E030044B06Ril	0.804313536	0.558185868	0.099992892	profile #3
Gm2445	0.142658216	0.133973074	0.043242988	profile #3
Gm8349	0.084861888	0.108648634	0.045445915	profile #3
Mir6931	4.44875148	3.522563967	0.615976384	profile #3
Gm7889	4.486828734	4.462404624	2.236290046	profile #3
Gm6652	0.821875862	0.825541249	0.383402123	profile #3
1700047M11Ril	0.132198618	0.114871307	0.060108666	profile #3
2310020H05Rik	1.894250134	2.024295938	0.237596198	profile #3
2310039L15Rik	1.152944603	1.323627055	0.335238317	profile #3
1810044D09Ril	4.644904543	5.157233197	2.344766071	profile #3
Gm28231	0.163748507	0.140414631	0.06494344	profile #3
Gm10925	919.2077009	947.0231381	392.2204956	profile #3
A630072M18Ri	0.068443014	0.077092712	0.03370222	profile #3
Plet1os	5.930647699	4.989197626	2.679398086	profile #3
2310040G24Ril	19.29733084	17.29532039	6.352107434	profile #3
Gm19461	2.276734707	2.588934915	0.62760193	profile #3
Gm28979	2.284174473	2.466438499	0.657615589	profile #3
Snrpn	10.54300821	10.8830732	5.40484327	profile #3
Gm37035	0.065683881	0.101466089	0.019644786	profile #3
Gm6185	0.023944432	0.028187941	0.01106242	profile #3
Strit1	19.99951334	14.12633441	3.376463142	profile #3
Gm18407	0.107742372	0.197977013	0.053413924	profile #3
Peg13	13.83517996	13.59111709	6.797514079	profile #3
Gm31520	0.755751155	1.078426249	0.234167593	profile #3
4931431B13Rik	0.148080156	0.265421732	0.031720426	profile #3

Gm30459	0.11826402	0.185106693	0.035370495	profile #3
Gm31663	2.366272126	2.521199845	0.682431272	profile #3
4833411C07Rik	0.083348974	0.097843281	0.040391894	profile #3
Vmn2r-ps88	0.115135791	0.129686435	0.053193179	profile #3
Gm19410	0.116585864	0.145825555	0.066431046	profile #3
Gm31166	3.075780928	2.793916439	1.348146975	profile #3
E330011O21Ril	2.31344783	2.341821589	0.800498861	profile #3
Gm36210	0.759950233	0.774468325	0.362913998	profile #3
Gm33543	0.455040237	0.582587793	0.230861155	profile #3
A030001D20Ri	1.230653767	1.088408145	0.502528075	profile #3
Gm5131	0.569826234	0.535134776	0.238593701	profile #3
Nudt8	0.734028709	0.643061898	0.223067843	profile #3
Gm17853	0.504767821	0.363517872	0.125120831	profile #3
Gm39460	0.134465203	0.215194668	0.03378145	profile #3
Gm19299	0.086610789	0.100344917	0.027981739	profile #3
Gm10635	0.857798379	0.952730012	0.226804624	profile #3
Gm6018	0.945014449	1.084739308	0.418826209	profile #3
Gm31013	8.803781775	8.312265629	3.060067295	profile #3
C230072F16Ril	0.286290118	0.301534532	0.094670317	profile #3
Pbld1	0.35918782	0.359808202	0.065326857	profile #3
D830005E20Ril	2.934739286	4.148561316	1.896666666	profile #3
4930567K20Rik	0.075247454	0.070666331	0.0337576	profile #3
Gm35533	1.765378716	1.701226486	0.540234103	profile #3
Gm33016	0.628629792	0.587146398	0.126343621	profile #3
Ndufb1	129.4603611	110.9332689	53.64710888	profile #3
E130119H09Ril	2.219294562	2.284231988	0.612211062	profile #3
Gm4814	0.144039174	0.11304417	0.043772822	profile #3
Gm6416	2.085663534	1.816242995	0.546998152	profile #3
Gnai3	7.96004104	8.635784444	35.31157323	profile #4
Cdc45	0.944898688	1.233948394	6.375847919	profile #4
H19	3.498674619	6.163194441	75.61809514	profile #4
Scml2	0.060910792	0.062742397	0.389912342	profile #4
Ngfr	0.185250596	0.243517899	1.640220343	profile #4
Tfe3	1.469352	1.45801665	4.964352044	profile #4
Brat1	1.597914058	1.358401913	3.031572137	profile #4
Slc22a18	0.245180278	0.256561694	0.729989495	profile #4
Pih1d2	0.060516512	0.062803351	0.225010667	profile #4
Drp2	0.037880523	0.018286197	0.177609261	profile #4
Tspan32	0.051971786	0.061182392	0.899361092	profile #4
Trim25	8.882941001	6.903238496	16.87351367	profile #4
Scpep1	6.879943877	6.908248852	55.7596032	profile #4
Itgb2	1.78749685	2.070945486	49.12189653	profile #4
Hddc2	0.616694496	0.621291789	1.6840308	profile #4
Pemt	0.285978918	0.310764011	0.982724817	profile #4
Clec10a	0.638628327	0.695422465	5.458401785	profile #4
Rnf17	0.030142386	0.033413842	0.116494851	profile #4
Ccm2	6.884079237	6.726502126	14.29076967	profile #4
Mx1	0.141888712	0.085793659	5.103915837	profile #4
Fap	0.78908823	0.69978452	4.233114833	profile #4
Septin1	1.155858332	1.000035894	2.507601291	profile #4
Zfp385a	7.739057728	8.501705572	25.14882892	profile #4
Itga5	11.89936954	10.86761309	40.8413698	profile #4
Sox9	0.402961347	0.395201669	6.790487906	profile #4
Gm2a	4.37591201	4.195117781	24.76711517	profile #4
Sema4f	0.113742844	0.072100384	0.232650438	profile #4
Cd52	2.417560621	3.030532707	52.00860276	profile #4
Loxl3	1.287364104	1.141822369	28.76742196	profile #4
Dnmt3l	0.047218533	0.017447773	0.720229925	profile #4

Sult5a1	0.170281633	0.189898773	0.416156273	profile #4
Rpl13	147.5680215	150.2944336	309.9517937	profile #4
Serpinf1	7.455762375	7.021677031	60.44140974	profile #4
Il12rb1	0.020734829	0.010759186	0.088832648	profile #4
Fmr1	1.473938661	1.697871117	5.849974504	profile #4
Il4	0.277478317	0.297422679	1.300227249	profile #4
Dlg3	0.982082292	0.901014479	2.737743087	profile #4
Mmp11	2.420893148	2.0594548	4.723998237	profile #4
Smarcb1	2.101191889	1.837948393	4.911454136	profile #4
Mmp14	2.564382602	2.28611527	103.4524333	profile #4
Slc7a7	0.711027652	0.696808522	7.678008261	profile #4
S100a4	0.237763295	0.093300154	3.54050174	profile #4
S100a6	17.9323661	16.08990161	130.5112783	profile #4
Mapk7	2.954070493	2.54207884	11.76407041	profile #4
B9d1	1.709809894	1.505437256	4.291220372	profile #4
Sec24b	3.905742643	3.719666714	8.169975143	profile #4
Nhp2	24.0712168	21.34798704	70.94299038	profile #4
Mfsd10	2.421361132	2.359898762	9.98588391	profile #4
Kctd10	8.954148895	7.716447091	16.26407447	profile #4
Col6a1	18.73574761	17.1042195	107.4943784	profile #4
Lgals9	13.92652789	11.95813708	51.35910054	profile #4
Cfp	3.439350146	3.281954454	81.35562217	profile #4
Timp1	1.821185813	1.541847029	267.0549156	profile #4
Mxd1	1.223622337	1.356018526	2.452342638	profile #4
Gmcl1	1.101152595	1.090920635	2.775006798	profile #4
Oas1c	0.273769322	0.271870053	1.175550529	profile #4
Ocrl	1.268605617	1.332600806	2.580399489	profile #4
Calm1	55.37427492	55.95772883	159.4410236	profile #4
Uhrf1	0.209805943	0.31983617	9.482478433	profile #4
Lsr	0.368095854	0.244013574	2.098630406	profile #4
Ckb	18.15524096	14.03562704	52.75374233	profile #4
Rrp15	2.986563413	2.170071131	5.82619713	profile #4
Rnd2	2.835959307	3.462295109	6.000879946	profile #4
Acp5	0.068400869	0.214728236	14.59226317	profile #4
Cnn1	1.370267408	0.77485012	5.331257593	profile #4
Ube2c	0.178017646	0.101922593	13.55172895	profile #4
Glmp	4.326074988	3.894922103	14.28976274	profile #4
Snd1	6.323394057	6.315704111	12.97344624	profile #4
Col18a1	2.030430581	1.982589912	22.95667919	profile #4
Kpnb1	18.55597595	19.66999432	39.22563786	profile #4
Cyp51	2.034578658	1.865722215	6.383615172	profile #4
Tubb6	20.44214018	20.83976026	109.3766512	profile #4
Meox1	4.798806028	3.275921247	53.67029374	profile #4
Col1a1	10.91704133	10.6185043	705.6671633	profile #4
Foxm1	0.073560467	0.053730483	3.762872767	profile #4
Tulp3	0.871985387	1.002278832	2.44364988	profile #4
Tubb5	33.31878217	29.34812847	179.6299704	profile #4
Fkbp10	8.412460209	9.059556201	47.43039145	profile #4
Ergic1	5.627850308	5.494303857	12.07633463	profile #4
Acap1	0.226837671	0.262572212	0.824134413	profile #4
Eef1e1	3.884242597	3.345408309	8.215161858	profile #4
Cldn15	1.783278288	1.282657483	3.262006765	profile #4
Il16	0.8550847	0.589634834	2.262072892	profile #4
Tcirg1	4.2867605	3.972016924	33.53103918	profile #4
Naglu	5.0603771	4.687740129	19.36807389	profile #4
Smo	3.589347713	3.385321215	13.43757883	profile #4
Rin2	4.612170467	4.625330704	14.04024556	profile #4
Folh1	0.014418875	0.013759021	0.028400157	profile #4

Pwp1	4.499453491	4.463438768	9.536151951	profile #4
Septin7	10.5653496	10.60607207	21.87603242	profile #4
Cpa3	0.186010515	0.143671166	0.366496442	profile #4
Gria3	0.138336555	0.1639266	1.129932862	profile #4
Blvra	4.80679257	4.73169917	14.30143633	profile #4
Pdzd4	0.621757617	0.723128387	1.976987925	profile #4
Ssr4	18.47159714	17.74004786	48.0027202	profile #4
Bcap31	48.10931128	46.03930152	99.20403255	profile #4
Ltbp2	0.316707836	0.26754958	26.96472978	profile #4
Ift46	1.275430265	1.161962964	3.01957512	profile #4
Cd3g	0.056634552	0.06379192	0.143909597	profile #4
Spag5	0.024689503	0.027809716	2.739473919	profile #4
Unc119	1.921146181	1.936435546	5.10133013	profile #4
Rab34	4.298727695	4.135094115	13.16193277	profile #4
Ccne1	0.367708283	0.22515409	2.602743189	profile #4
Bbc3	3.786961758	3.309256305	12.46373275	profile #4
Acp2	4.435680725	4.001024063	13.71036358	profile #4
Slc39a13	4.486819787	4.521023845	12.54036958	profile #4
Spi1	2.827014723	2.572844123	56.54172375	profile #4
Stat6	13.33231658	14.50810918	26.69799711	profile #4
Napsa	0.088401614	0.215591509	2.585756035	profile #4
Smg9	2.621090766	2.314503681	5.971742956	profile #4
Mov10	1.287746697	0.913734438	4.178576377	profile #4
Rhoc	47.11416344	38.2430198	176.3847234	profile #4
Def6	1.584973958	1.532724873	7.928133444	profile #4
Peg3	0.660017391	0.661260657	1.757680515	profile #4
Dbf4	0.102378954	0.149329591	1.822663393	profile #4
Daxx	4.782937801	4.72240132	11.45403253	profile #4
Tm9sf1	7.145481499	7.011994179	14.22683188	profile #4
Irf9	3.428806278	3.014707088	10.11614558	profile #4
Gmpr2	7.409133661	6.75747851	18.3774363	profile #4
Armc6	1.84457279	1.949828785	3.972561812	profile #4
Snx9	11.99828598	12.58496717	28.22658767	profile #4
Hltf	2.528444869	2.48964012	5.044288186	profile #4
Rgs19	2.573672158	2.220952887	19.13228173	profile #4
Snrpd1	16.03647613	13.772336	49.44884731	profile #4
Tchp	0.909044401	0.63431867	1.32979351	profile #4
Tiam1	0.574168972	0.508018977	2.147806667	profile #4
Axl	20.06570558	20.1745846	46.88641383	profile #4
Tgfb1	6.55845896	6.172940536	22.94481488	profile #4
Zfp40	0.807961558	0.581592444	1.261579215	profile #4
Gtf2f1	19.03646631	17.15531735	40.19308688	profile #4
Dennd1c	0.641751191	0.70105487	5.057404782	profile #4
Med6	6.429303518	6.137821871	13.47746781	profile #4
Lcp2	0.372229477	0.287245402	5.734779591	profile #4
Fkbp7	9.975988167	10.49384842	34.61704604	profile #4
Mea1	28.65236401	25.13601002	61.04525169	profile #4
Grin2d	0.306818169	0.281889769	0.841471665	profile #4
Nudt14	3.319043322	2.575013698	6.144264624	profile #4
Top3a	1.086917918	0.92411581	2.605772579	profile #4
Chaf1a	0.75904957	0.734327426	5.484200866	profile #4
Tmem39a	2.419370257	1.943122696	7.142775092	profile #4
Mcm2	1.237266749	1.231784449	6.788846105	profile #4
Nab1	6.389151167	6.61821228	13.63020524	profile #4
Il17ra	0.481494714	0.509887518	4.627932384	profile #4
Lamb1	11.56064581	11.17410913	26.62553258	profile #4
Relb	2.409990249	1.66898005	7.881955721	profile #4
Apoe	225.4912389	230.4878622	2963.866987	profile #4

Prkar2b	0.329517877	0.413988076	3.350802326	profile #4
Rab8a	6.065081755	5.962304722	16.93139558	profile #4
Hmgn2	11.23759981	9.74004822	27.15225282	profile #4
Fam32a	8.0116436	7.081246788	16.27010673	profile #4
Stard3nl	1.88118634	1.758771406	5.376462534	profile #4
Efna2	0.778869657	0.911930596	1.722730656	profile #4
Pafah1b2	4.350728875	4.386724058	11.23075257	profile #4
Tbc1d8	1.421586459	1.372842577	3.102038544	profile #4
Slc2a3	0.281093426	0.374184297	0.956315027	profile #4
Sri	11.41816523	10.98280492	28.78772728	profile #4
Zfp959	1.248745017	0.920134517	2.113907348	profile #4
Sh3gl1	10.3501565	10.53003363	40.76443713	profile #4
Ebi3	0.281374025	0.232851666	1.389023926	profile #4
Yju2	4.413099543	3.574299716	8.414115245	profile #4
Grk5	6.025039577	7.066497042	17.88725356	profile #4
Hck	0.862857998	0.885548688	20.87370833	profile #4
Mob3a	0.725419173	0.653043732	4.216428389	profile #4
Cacnb3	1.462140024	1.816242995	8.717952797	profile #4
Fkbp11	2.342721965	1.639836357	22.69759218	profile #4
Pld3	8.556695733	8.165987548	51.55772487	profile #4
Prkcsh	12.25594237	11.34141999	23.73475881	profile #4
Cyp4f18	0.253529156	0.619044678	9.940748864	profile #4
Impdh1	9.794276179	8.849635724	17.81322314	profile #4
Slc25a1	2.828975278	2.374758939	13.60063529	profile #4
Ddr1	7.630073474	7.218378461	16.98509225	profile #4
Homer3	2.610235487	2.312541953	7.32761209	profile #4
Sec14l2	0.228103907	0.264275015	1.372455931	profile #4
Rps6ka1	1.98810205	1.812077058	10.99024738	profile #4
Taf6l	1.809220809	1.651363104	4.202488476	profile #4
Man1a	3.471039375	3.809110821	11.83144605	profile #4
Itpkc	0.604283174	0.759567435	1.553703803	profile #4
Coq8b	1.46659006	1.217621617	3.498865599	profile #4
Brd8	2.219988704	2.162478729	4.460587639	profile #4
Kif20a	0.247785345	0.172246438	5.487642086	profile #4
Dnase2a	1.160687434	1.218941786	8.420137662	profile #4
Calr	86.06221241	79.33093098	228.6709663	profile #4
Syce2	0.564446195	0.526045989	2.941537631	profile #4
Bax	16.29945834	13.59183475	59.0918653	profile #4
Zfp81	0.676247181	0.638647365	1.297642071	profile #4
Fancl	1.26210667	0.969252018	2.041198136	profile #4
Col5a3	2.718876935	1.928541174	20.4383676	profile #4
Dnmt1	3.011603937	2.712132974	9.954318646	profile #4
Ppan	2.404394182	1.63298571	6.639388163	profile #4
Cacna1e	0.003683466	0.003514898	0.013432653	profile #4
Trmt10a	0.304139858	0.196090572	0.920103139	profile #4
Psap	416.0284791	430.5424759	1214.393993	profile #4
Ptpn6	1.717914758	1.750127083	34.16228768	profile #4
Eno2	0.280404962	0.232691367	0.836257819	profile #4
Clcn5	0.222288888	0.236724173	2.073729786	profile #4
Utp20	1.378749671	1.286149395	2.614532569	profile #4
Spic	0.033239966	0.013573014	0.71260302	profile #4
Il11	0.046749164	0.027081158	0.498414291	profile #4
Col26a1	0.040265119	0.046650077	0.392312704	profile #4
Bid	0.264955823	0.245576518	2.794934429	profile #4
Ralb	7.66020604	6.774991619	21.16384905	profile #4
Dnajb11	15.5044788	14.98287096	37.59549806	profile #4
Clec11a	0.419638642	0.252125509	7.439042809	profile #4
Zfp324	0.891085362	0.709528169	2.068519662	profile #4

Gab2	4.001909437	3.702118784	9.991521043	profile #4
Coro1c	3.563300236	3.363670554	16.36697184	profile #4
Arhgef40	3.098736647	3.211627832	11.10897298	profile #4
Cd33	0.553914128	0.483801699	3.186497972	profile #4
Nkg7	0.205819074	0.241610814	0.969515325	profile #4
Stxbp2	0.562804287	0.562832944	9.2753351	profile #4
Sgce	2.010666705	1.931137955	4.07442104	profile #4
Chn2	0.27825413	0.349832058	0.691694368	profile #4
Slbp	1.508286443	1.345143759	5.123877288	profile #4
Cnn2	21.63710415	18.90539799	107.9335855	profile #4
Myo9b	5.546877996	5.538483115	19.02129991	profile #4
Ly9	0.102953098	0.082381751	10.87143764	profile #4
Cd244a	0.038580524	0.01069195	1.140491139	profile #4
Adgre1	2.942071087	3.062389149	63.82215026	profile #4
Rab23	0.955300489	0.947033161	3.193424165	profile #4
Plod3	9.043764824	8.645139816	36.15205701	profile #4
Ap1s1	17.96212628	16.63374499	59.4228307	profile #4
Mapk13	0.084440946	0.02921062	0.27908069	profile #4
Nes	11.08059962	8.110249659	23.22339894	profile #4
Matk	0.176013605	0.115367323	0.996104216	profile #4
Nmrk2	3.948229098	2.615625026	26.2650153	profile #4
Dtx2	1.110199561	1.223279466	2.364280166	profile #4
Rasa4	1.296363128	1.163175564	7.347864779	profile #4
Syt5	0.335926132	0.262860235	0.555414	profile #4
Sgsh	1.224489372	1.308484985	5.257895374	profile #4
Cstb	17.80766902	15.99466027	209.305519	profile #4
Sh2b2	0.149575749	0.125368351	2.45875973	profile #4
Cd44	1.017769128	0.892575144	13.13113293	profile #4
Slc1a2	0.021486176	0.016815068	0.055360113	profile #4
Eif2ak4	1.015873162	1.081415971	2.404951742	profile #4
Wdr1	10.2305635	10.06512804	30.58964057	profile #4
Slc2a9	0.455288098	0.312805911	1.378064539	profile #4
Ccn4	0.293079558	0.255095426	8.74802561	profile #4
4930550C14Ril	0.026156874	0.02946253	0.166927947	profile #4
Man2b1	4.39432545	4.760028055	23.19005202	profile #4
Spc25	0.130665399	0.147178645	5.418969156	profile #4
Zfp287	0.376001632	0.24884141	0.577179443	profile #4
Cadm3	0.264227464	0.302100685	0.584871616	profile #4
Slc1a3	0.16526763	0.134032223	1.665736103	profile #4
Msh6	1.835195889	2.022120641	5.346860357	profile #4
Tbl2	1.797173691	1.470567212	3.63845605	profile #4
Bud23	4.166833299	3.534881808	8.198343687	profile #4
Nid1	23.87722112	26.99096592	59.52019307	profile #4
Mcm5	0.977576172	0.995487382	12.77771288	profile #4
Hmox1	2.793309243	3.453762318	255.9774629	profile #4
Cic	6.300165055	6.916293805	14.22416856	profile #4
Pafah1b3	0.969527941	0.817509168	6.079075904	profile #4
Il27ra	0.694082834	0.64448907	2.164993963	profile #4
Asf1b	0.462652757	0.312179322	5.634017625	profile #4
Ddx39a	3.753047098	3.661006539	10.44280119	profile #4
Por	15.93083059	14.10625932	28.83890827	profile #4
Trim28	13.00153891	12.64555909	29.95804352	profile #4
Ctr9	2.526895316	2.315709819	5.230563465	profile #4
Snx6	7.419956128	6.688233045	22.24377428	profile #4
Mthfd2	0.530938778	0.493381199	5.817064046	profile #4
Kit	0.649158119	0.626005196	1.602994435	profile #4
Ranbp1	20.58939913	18.27504494	60.27473114	profile #4
Rfx5	0.921308021	0.802887116	2.092852083	profile #4

Slc30a4	2.639511227	2.673674019	7.092605129	profile #4
Tnfsf14	0.022893081	0.04751636	2.723851554	profile #4
Ergic3	27.91041399	27.02528474	69.1577974	profile #4
Itgae	0.020532486	0.011894191	0.31318446	profile #4
Tuft1	0.450568437	0.340068965	1.358345924	profile #4
Rcn1	9.456769703	9.564258326	57.52576046	profile #4
Ankrd13d	0.41392721	0.310090267	1.582042976	profile #4
Prg4	1.773521597	2.136087983	5.645685355	profile #4
Dhx34	2.57807975	2.323652918	5.425009791	profile #4
Snf8	8.959703338	8.507914517	17.23792811	profile #4
Cdcp3	0.026997543	0.01906928	0.049891875	profile #4
Atp6v1b2	13.78504223	14.08144663	32.64401307	profile #4
Tep1	1.484642759	1.582911212	7.901062592	profile #4
Ttc5	6.888635043	6.06729735	16.09872337	profile #4
Arpc2	65.86002841	66.57473094	195.5799252	profile #4
Rps9	152.7464814	156.9156171	310.4445864	profile #4
Susd2	0.958391876	0.840706365	2.614178669	profile #4
Crip1	20.76716383	18.79565973	63.33534021	profile #4
Pgrmc1	16.15773943	16.48996064	38.6067332	profile #4
Elovl1	5.727750045	5.456724186	20.50030868	profile #4
Hyi	1.508596802	1.132833609	5.440296825	profile #4
Cdc20	0.579058964	0.614736551	19.204086	profile #4
Adamts4	0.067358028	0.184257192	9.28702856	profile #4
Nectin4	0.111201626	0.125255078	1.860937652	profile #4
Srm	10.16003151	9.268632171	35.83295265	profile #4
Rbm14	1.633573694	1.866688629	4.896303329	profile #4
Zdhhc24	1.167578516	1.108472118	2.903953529	profile #4
Nsmf	3.053497126	3.237075122	7.590849114	profile #4
Ptbp1	7.108687779	6.690193251	29.52268248	profile #4
Mvd	3.548427499	3.458579991	7.306891459	profile #4
Cyba	35.75883563	34.55950858	244.3376183	profile #4
Rundc3a	0.405178726	0.360419913	0.930672628	profile #4
Cdt1	0.456949754	0.450104019	6.342988584	profile #4
Aprt	7.221891832	6.359373066	52.93567966	profile #4
Gtf2h1	6.887386315	6.427455104	13.05222229	profile #4
Aplp1	1.311871406	1.258970927	3.356738508	profile #4
Pola1	0.57245207	0.681565656	1.703316439	profile #4
Cdc42	52.68265985	52.08842415	175.8128684	profile #4
Cdk4	11.95927064	10.76696776	39.53452416	profile #4
B4galnt1	0.531023938	0.728861621	10.90964716	profile #4
Mettl1	3.059742523	3.036443398	6.399649911	profile #4
Tspan31	14.7950279	13.55054702	28.70455568	profile #4
Saal1	0.568847721	0.483916585	1.598054715	profile #4
Cnp	3.114632509	3.009970128	10.3715595	profile #4
Sulf2	8.194074536	8.871194094	26.9152747	profile #4
Tmco6	2.12241717	2.159100093	4.330867403	profile #4
Hap1	0.519829766	0.477451026	1.249178098	profile #4
Neu1	5.691032922	5.851627021	13.11238156	profile #4
Ddah2	19.93171838	18.10464301	55.43911283	profile #4
Clic1	19.0644134	17.283594	141.6027187	profile #4
Lsm2	2.143892719	2.034414799	4.364283663	profile #4
Pole	0.157599852	0.168174022	1.029276668	profile #4
Stx1a	1.371523801	1.16495706	3.192309005	profile #4
M6pr	11.41343271	10.86685485	38.46302116	profile #4
Tssk4	0.109679119	0.0322792	0.405377399	profile #4
Tgfbr1	2.404026009	3.093783982	14.40148618	profile #4
Khsrp	11.43419297	10.65972379	21.90004458	profile #4
Dio2	0.080876455	0.113928951	1.30123025	profile #4

Cpt1c	0.53909631	0.492970415	2.182239476	profile #4
Twist2	0.044451473	0.055660311	0.412132013	profile #4
Aldh16a1	1.975755929	2.132195619	5.651786063	profile #4
Hnrnpa0	56.0185904	61.5474428	142.4109369	profile #4
Crlf1	0.197075496	0.158558232	12.90303439	profile #4
Ctsd	157.0726591	153.2957413	630.0675217	profile #4
Rplp1	887.8478486	815.2649979	2186.961107	profile #4
Ift22	2.685174515	2.36972485	5.824308803	profile #4
Ap2s1	26.38095743	24.33153627	71.18201307	profile #4
Clstn3	0.018633087	0.017780377	0.077477021	profile #4
Fbxw9	0.792400395	0.770269274	2.401947965	profile #4
Relt	1.312337404	1.171488337	3.779991664	profile #4
Snrpb2	6.010354271	4.931431478	15.05031268	profile #4
Prpf31	3.787127535	3.394296554	6.834998582	profile #4
Sertad1	7.335280689	7.82443969	16.7029793	profile #4
Carhsp1	10.86188673	10.28928113	31.80678056	profile #4
Elk3	8.341304266	8.54208396	18.03746784	profile #4
Arpc5	22.00348165	23.30576215	138.5692463	profile #4
Pou2f2	0.211681149	0.235754471	2.892309254	profile #4
Rps18	99.92714344	94.31922676	239.2634726	profile #4
Rpl10	99.00542594	91.67245378	314.6173814	profile #4
Rps15a	26.21316938	22.84913645	51.55269081	profile #4
Gprc5b	5.241318312	4.931762027	10.51443749	profile #4
Man1a2	3.245866756	3.430673458	7.5903688	profile #4
Dynll1	7.120943151	6.352568313	36.54542265	profile #4
Pdcl	4.590207075	4.677394651	11.04619234	profile #4
Tmem184b	15.55399376	14.46815702	31.03557091	profile #4
Ewsr1	13.63852082	12.60617001	30.21010909	profile #4
Derl3	0.094389681	0.110803956	0.34306763	profile #4
Gnat2	0.030907905	0.04424019	0.167413949	profile #4
Ccl8	0.27610367	0.408093516	48.88272863	profile #4
Trpm5	0.014701362	0.0085163	0.079144108	profile #4
Trpm2	0.033972785	0.061226588	0.54512847	profile #4
Met	0.116385333	0.099338724	0.917684471	profile #4
Nav1	2.46450635	1.733169057	4.872542718	profile #4
Tnpo1	5.218060576	5.83003533	11.13130638	profile #4
Rnmt	1.443929825	1.462596553	3.161379818	profile #4
Cdk9	4.978780233	4.951241477	12.73466153	profile #4
Tor2a	2.581401255	2.172120828	10.58666337	profile #4
Fpgs	2.026049679	2.180246588	4.28573047	profile #4
Apobec3	1.716354461	1.41412152	6.355688527	profile #4
Sardh	1.714262221	1.504196339	4.4667296	profile #4
Vav2	3.085639201	2.774445754	6.346331213	profile #4
Oit3	0.016868601	0.015841627	0.42951393	profile #4
Tex11	0.035230329	0.045105372	0.151368931	profile #4
Bcr	1.69519917	1.782760796	3.973352244	profile #4
Fxyd5	2.782375043	2.835529544	31.23504103	profile #4
Kcnd1	0.072446403	0.092753094	0.825092906	profile #4
Vps4b	3.789047126	3.530238053	9.9559215	profile #4
Kif19a	0.045510903	0.076893727	0.414587842	profile #4
Rassf1	1.987584532	1.98010884	4.491937963	profile #4
Slc3a2	10.89897779	12.70701036	34.77115997	profile #4
Stx5a	4.096122286	4.342122392	10.30156242	profile #4
Tnfrsf13b	0.28512218	0.109014527	4.998617949	profile #4
Tmem86a	11.54733296	11.08803355	31.68452953	profile #4
Mrpl52	35.91930545	28.93869588	62.32523583	profile #4
Eya4	0.029483759	0.038744838	0.612972461	profile #4
Gm266	0.3428488	0.472562913	2.222722651	profile #4

Psen2	3.205303879	2.977055724	7.986786318	profile #4
Fads1	4.925721864	5.108183424	16.86170644	profile #4
Kdelr3	5.237010765	2.873630855	43.00334139	profile #4
Tbrg1	5.836620544	5.343903519	14.32885965	profile #4
Odc1	19.32942266	20.19470185	38.80850214	profile #4
Thg1l	1.623282668	1.746443986	3.976163312	profile #4
Adam19	2.240766582	1.874669004	14.69651259	profile #4
Exoc3l2	1.018819793	0.71908027	2.843344937	profile #4
Fsd1	0.024346817	0.031171218	0.365075248	profile #4
Pgam1	25.58165105	25.02771117	88.05266347	profile #4
Gltp	9.822312332	9.142672833	54.92760228	profile #4
Scarf2	4.910565797	4.280213867	19.01548652	profile #4
Med15	7.409636872	6.697426059	15.7786596	profile #4
Rpl15	61.36965794	55.95370371	152.703355	profile #4
Tmem167	1.657456793	1.356830172	7.481211495	profile #4
Mplkip	1.428727012	1.216220441	3.40162361	profile #4
Kif11	0.076836834	0.075728912	6.640120606	profile #4
Mlkl	2.236016789	1.975312659	11.10362293	profile #4
Rps5	122.3578728	119.4210129	288.4450727	profile #4
Gfod2	1.396171383	1.131379052	2.324615738	profile #4
Enkd1	0.465244459	0.461630496	1.98357039	profile #4
Atp6v0d1	15.83175907	15.2722829	39.34935	profile #4
Iglon5	0.161429034	0.187027311	0.407773795	profile #4
B4galnt2	0.107513561	0.121100922	0.347701753	profile #4
Card14	0.05025471	0.057545872	0.102158626	profile #4
Tmem175	1.091236844	1.038640984	2.294198105	profile #4
Bcas1	0.074749126	0.046798895	0.260569075	profile #4
Aldh1a2	1.599506937	1.307046205	31.8154945	profile #4
Cad	2.002290621	1.83484989	4.332653628	profile #4
Pea15a	26.88845659	21.76865252	74.57972811	profile #4
Tnfaip8l2	1.38322525	1.214066173	45.51174228	profile #4
Ehmt2	11.2739104	10.67751653	23.74686355	profile #4
Elof1	14.92963503	12.43618235	27.27185685	profile #4
Clip3	1.103161414	1.077504411	4.857748703	profile #4
Mcemp1	0.061960627	0.072941486	2.257755475	profile #4
Tctex1d2	2.551709802	2.601232561	6.280895227	profile #4
Glis2	2.617920358	2.566746332	9.234513906	profile #4
Bicc1	2.970560341	3.246748003	15.45545261	profile #4
Ube2z	15.80647051	15.60869324	31.82888829	profile #4
Mertk	2.786041852	3.015053327	8.768483297	profile #4
Hps5	1.506602199	1.476473171	3.942992466	profile #4
Piezo1	4.03949848	3.511977947	8.859097687	profile #4
Blk	1.088405614	0.785763502	2.028990511	profile #4
Wdfy2	1.649508148	1.470080805	4.382791324	profile #4
Chfr	3.285924098	2.737384636	8.054200112	profile #4
Hoxa2	0.078227473	0.092091194	0.282481564	profile #4
Tbp	0.673594646	0.56695504	1.228091135	profile #4
Plekhg4	0.050695058	0.04837509	0.199703187	profile #4
Slc9a5	0.503477389	0.413006944	1.417303828	profile #4
Elmo3	0.854828288	0.583421498	1.349900923	profile #4
Tmem208	10.11145404	8.246203351	23.51414578	profile #4
Surf4	33.64484682	33.19722415	86.98277428	profile #4
Galns	1.15274823	1.160718059	12.16547481	profile #4
Npdc1	6.092423759	5.858848372	20.74860806	profile #4
Ube2i	10.16919508	9.294804216	22.26250218	profile #4
Lrrk1	1.868088543	1.728664382	5.404241046	profile #4
Aldh1a3	0.203723883	0.229585221	2.352804454	profile #4
Actn1	4.500471417	4.48504584	33.37316186	profile #4

Abca1	2.590471972	2.881311864	16.27127408	profile #4
Nipsnap3b	2.524115054	2.842253413	16.05837238	profile #4
Lage3	8.254739667	6.603805224	13.45869012	profile #4
Sash1	4.728692989	5.40740117	11.26256167	profile #4
Zdhhc12	1.449093119	1.063953862	5.132791449	profile #4
Cybb	1.554699455	1.536092335	24.85467254	profile #4
Golga7	4.466056738	4.228843028	13.09324111	profile #4
Cd48	1.12889239	1.271559676	30.591938	profile #4
Cd83	1.588487847	1.970185831	4.83875927	profile #4
Gzmb	0.032231607	0.036304977	0.893467596	profile #4
C4a	0.041145572	0.033602286	0.080793551	profile #4
Ager	0.197695902	0.127247291	0.610979454	profile #4
Atf6b	11.98665065	10.5705028	28.01515125	profile #4
Egfl8	0.367963789	0.371293287	0.922860871	profile #4
Itga2	0.077865562	0.061918487	0.817962752	profile #4
Atp6v0e	11.56632509	10.1037034	39.12140523	profile #4
Steap2	0.085198657	0.080011697	1.306210791	profile #4
Pdzd11	5.940617079	5.122705624	11.82952672	profile #4
Setdb1	2.403332509	1.924688241	4.765865789	profile #4
Arnt2	0.018349457	0.027121314	0.183775501	profile #4
Cers2	8.448574144	8.626636564	22.28084266	profile #4
Plekho1	25.74207289	24.50679539	64.84971781	profile #4
Cnih1	24.23307729	22.14862577	44.59512084	profile #4
Tnr	0.018275301	0.021173269	0.053138555	profile #4
Fcrls	2.377677679	2.166663595	87.64184686	profile #4
Cd5l	0.023099046	0.043385518	12.16221795	profile #4
Lcorl	0.196381174	0.21784569	0.651250443	profile #4
Lta4h	4.077136897	3.955755052	9.5569083	profile #4
Dstn	48.80347715	50.03331149	106.4751102	profile #4
Macroh2a1	4.907838529	5.481538193	15.12267708	profile #4
Fcgr1	0.926424327	0.69450775	37.71254598	profile #4
Ncf1	0.871380772	1.085878285	23.28150086	profile #4
Adss	2.244850722	1.832552171	9.184859073	profile #4
Cacna1d	0.118020624	0.116564205	0.660773833	profile #4
Stk32c	0.542868756	0.591738595	1.683097939	profile #4
Lbp	1.226335596	1.116589049	3.123106096	profile #4
H2-M3	4.528101701	4.471140614	12.94080469	profile #4
Lonrf3	0.123372661	0.20855095	1.351205279	profile #4
Ctsz	31.41313468	29.38003069	391.3984083	profile #4
Sertad4	1.009845557	1.259576311	9.118936174	profile #4
Pls3	15.12603926	13.74600202	42.58143873	profile #4
E2f3	1.403353228	1.402018092	3.41719962	profile #4
Ppfibp1	5.262591333	5.969869208	13.10361832	profile #4
Cd34	16.70221067	14.4626757	37.88632705	profile #4
Dyrk3	0.040174547	0.049213921	0.272518186	profile #4
Lamp2	14.35212045	16.33123174	40.71268373	profile #4
Foxred2	0.093519962	0.141296966	0.575630398	profile #4
Nup50	4.031962634	3.551270316	9.63027934	profile #4
Ift27	5.887503129	5.80448494	11.93606412	profile #4
Cmah	0.238236544	0.244448495	0.855023646	profile #4
Ttll12	2.143831385	1.696168168	6.195094157	profile #4
Sulf1	2.091360145	2.190298353	12.1553996	profile #4
Kctd5	4.290283512	4.150248756	13.12291673	profile #4
Etaa1	0.650830863	0.759853021	2.309065117	profile #4
Matn4	0.062619273	0.085362305	0.239708572	profile #4
Il13ra1	8.704034319	8.376396987	21.1824853	profile #4
Rnd3	3.947435235	4.45820528	10.70158582	profile #4
Brca1	0.01653451	0.018329057	1.42093978	profile #4

Traf4	1.841822913	1.852877207	6.383385833	profile #4
Aldoc	0.014413012	0.03195461	0.447021783	profile #4
Stac2	0.074911849	0.086790841	0.981325589	profile #4
Rpl19	185.0477755	180.0426398	373.8583147	profile #4
Nek8	0.749781271	0.688655904	1.449600701	profile #4
Zfp207	8.001435987	6.63700692	17.7043714	profile #4
Timp2	20.81380857	22.36032468	81.86823404	profile #4
Igfbp4	19.81978169	20.72302957	62.05909385	profile #4
Cdc6	0.081388864	0.104202123	1.865481358	profile #4
Atad5	0.208932022	0.144682129	0.992497265	profile #4
Crlf3	2.596108081	2.342553214	6.397386109	profile #4
Tns4	0.054611564	0.060538742	0.643991567	profile #4
Abr	3.954023244	4.061411265	8.105466969	profile #4
Rab11fip4	0.012623717	0.021938246	0.237857202	profile #4
Cd40	0.883704681	0.765067808	3.546475893	profile #4
Slc35c2	4.150712926	3.678657366	9.156619949	profile #4
Elmo2	3.061518149	2.947971563	7.804898603	profile #4
Wsb1	5.273079767	5.871136726	28.30741334	profile #4
Ada	0.681248799	0.680410713	10.98133725	profile #4
Pgs1	4.72132306	4.250274251	9.684816876	profile #4
Birc5	0.075058619	0.05192999	8.83135339	profile #4
Pigt	7.70374724	7.376132082	17.42413196	profile #4
Etv4	0.268402894	0.117570112	3.044966736	profile #4
Mmp9	0.204433464	0.499197026	7.199909326	profile #4
Pltp	9.57487574	8.994224998	38.59408028	profile #4
Ctsa	26.14871947	23.79274663	99.4124664	profile #4
Pitpna	23.15604159	24.10953707	61.49172946	profile #4
Dhx58	0.603681436	0.616146004	6.408262871	profile #4
Mybl2	0.040612831	0.056275849	2.470022746	profile #4
Eya2	0.87615675	1.14367416	2.886757719	profile #4
B4galt5	2.75103852	2.491283429	21.4156172	profile #4
Ptgis	8.943184906	8.087127946	34.66827958	profile #4
Cyth3	5.197831283	5.489801194	10.63118974	profile #4
Cyth4	3.201589078	3.493030467	61.84481756	profile #4
Rrp7a	4.571724334	4.883817332	11.38082111	profile #4
Cyb5r3	22.82546944	21.38445712	58.53888267	profile #4
Ints2	0.549708558	0.422614706	1.45223879	profile #4
Stard3	7.721484556	6.403060771	13.16802653	profile #4
Vmp1	11.68500086	12.59121413	41.47587085	profile #4
Stk4	2.535384429	2.677873631	8.377489054	profile #4
Pmp22	18.65933476	21.94305471	49.87097763	profile #4
Gdf9	0.205101572	0.205352818	0.426421859	profile #4
Pfn1	97.99630858	86.9531054	323.7571875	profile #4
Ywhab	26.24038013	27.61419717	66.30244226	profile #4
Gpx3	91.4714169	93.6858237	331.8737053	profile #4
Kpna2	14.33807286	11.79641291	32.57235093	profile #4
Srsf1	17.10341168	15.58540228	31.99877608	profile #4
Abi3	1.143750423	1.042213525	2.82076544	profile #4
Kif3a	2.872028257	2.795778398	8.025196381	profile #4
Septin8	8.850872424	8.835050943	20.6236366	profile #4
Ypel2	3.14335647	2.815645794	5.850295898	profile #4
6330403K07Rik	0.085458307	0.069791093	0.68702515	profile #4
Kcnab3	0.129241432	0.154158663	0.265112044	profile #4
Chd3	4.42278205	4.415076344	10.6054761	profile #4
Adora2b	0.576989444	0.620180535	3.72797347	profile #4
Trpv2	0.794629493	0.789670868	7.526778207	profile #4
Pcgf2	1.347134291	1.094132744	3.602351882	profile #4
Cwc25	1.889376742	1.994883955	3.817150077	profile #4

Trim37	1.745135561	1.66215799	3.391031956	profile #4
Gabarap	95.00297067	88.2850108	198.3206702	profile #4
Cldn7	0.072427605	0.027970888	0.233995136	profile #4
2810408A11Rik	0.175912708	0.149211003	0.463311659	profile #4
Phf23	6.533296566	5.721533058	15.87604292	profile #4
Atox1	75.11620753	66.94361295	194.8640166	profile #4
Sparc	120.8066847	103.2219114	1030.353845	profile #4
Tada2a	1.874623078	1.796954027	3.93025188	profile #4
Ikzf1	0.250472573	0.44342297	6.264896732	profile #4
Copz2	13.16452709	12.48667059	26.44513829	profile #4
Cpsf4l	0.103037818	0.092847634	0.209457022	profile #4
Tnfsfm13	0.399804703	0.34989226	1.321606274	profile #4
Mpdu1	4.888921429	4.696363794	18.53529394	profile #4
Cd68	4.897255533	4.497459408	179.1327897	profile #4
Lsp1	5.390140605	6.539738501	39.32047166	profile #4
Rars	9.377360768	9.194504026	20.17876044	profile #4
Wwc1	0.056890716	0.021021753	0.195671574	profile #4
Fdxr	3.048540847	2.801721068	6.947617302	profile #4
Arrb1	2.672378805	2.397928287	8.442478589	profile #4
Cxcl16	2.599785823	2.002914461	30.29528481	profile #4
Med11	4.586066125	3.887617441	19.84791985	profile #4
Ccl6	6.340488778	8.226214792	95.89885534	profile #4
Natd1	2.56531873	2.183884139	5.246473142	profile #4
Ywhah	22.137735	23.18471056	61.04261197	profile #4
E2f2	0.028441984	0.066965125	2.757239472	profile #4
Slc35b4	0.932701084	0.871104793	3.402401973	profile #4
Dnah1	0.007307281	0.004050182	0.055729413	profile #4
Rab3d	5.953690434	5.417159381	11.37471979	profile #4
Dnase1l1	5.959357077	5.455733942	20.95013716	profile #4
Ccl9	2.459852117	3.274605597	42.58472937	profile #4
Scrn1	0.956537405	1.051216028	2.143366101	profile #4
Rab5c	28.62724974	27.59900288	110.4652854	profile #4
H13	3.615657821	3.606317699	11.51345439	profile #4
Rnf145	2.168462292	2.371124963	7.012961523	profile #4
Scn1b	12.72855283	11.72399997	28.43485858	profile #4
Chtf18	0.250670244	0.213955327	1.839597884	profile #4
Map1s	2.208733322	2.141720674	6.58861893	profile #4
Nop9	3.743021007	2.949646687	6.956679263	profile #4
Psmc3ip	1.662065358	1.491179994	3.293412643	profile #4
Aoc3	4.186746979	4.04439308	11.15635739	profile #4
Calm3	96.09733357	93.52789519	188.4806385	profile #4
Gipc1	19.11347359	19.16036566	44.84507993	profile #4
Plscr3	8.189490318	7.06188509	21.7285062	profile #4
Arhgef25	6.776925055	6.302736083	15.69776782	profile #4
Ap4m1	0.850937675	0.889755146	1.983518762	profile #4
Rcn3	16.75560172	14.53351667	167.7312331	profile #4
Arid3a	0.4514448	0.499613769	2.268313216	profile #4
Mydgf	10.9774878	9.560785661	36.27194897	profile #4
Lyst	0.57621728	0.673887767	1.759749443	profile #4
Tmc4	0.175811807	0.170531601	0.419679265	profile #4
Syne4	0.062843976	0.073981385	0.130653714	profile #4
Fbxo5	0.594922932	0.468502766	6.303686431	profile #4
Frk	0.429494598	0.337243947	1.665105111	profile #4
Adat2	1.7644142	1.446104999	3.338916806	profile #4
Cep57l1	0.348063899	0.329679718	1.067299365	profile #4
Plagl1	0.413662362	0.393388266	1.646369356	profile #4
Mical1	1.506112568	1.541114998	5.38966603	profile #4
Rab32	0.436681353	0.535787801	14.64898002	profile #4

Tube1	0.112197904	0.061984055	0.418719338	profile #4
Lama4	6.980274294	6.125781938	13.59779499	profile #4
Hebp2	0.151606951	0.146097694	1.02002927	profile #4
Fabp7	0.150494907	0.083414315	2.394969674	profile #4
Pkib	0.103624671	0.110821606	0.960452427	profile #4
P4ha1	12.73557453	11.56232476	31.4555213	profile #4
Septin10	4.122453659	3.620878614	9.893254143	profile #4
Cdk1	0.077629536	0.022086457	8.175981636	profile #4
Atp2b1	3.946436714	3.899110176	9.075714604	profile #4
Uhrf1bp1l	2.654417375	2.67863883	5.36886577	profile #4
Poc1b	1.515275393	1.231804469	2.66878403	profile #4
Tmpo	4.117981555	4.569660359	9.271458326	profile #4
Psen1	7.018356444	6.357953848	14.12438227	profile #4
Ikbip	3.1244208	2.927448457	12.80701436	profile #4
Epb41l2	5.12668151	4.973466211	15.58410369	profile #4
Apaf1	0.802233873	0.826845227	4.044238391	profile #4
Ahi1	0.199491735	0.276040355	0.964738013	profile #4
Arg1	0.021108464	0.024455696	66.0615319	profile #4
Enpp3	1.156787346	1.059500235	2.598423231	profile #4
Mtfr2	0.086513249	0.048723315	1.98091097	profile #4
Stx7	10.66131762	11.1181378	23.72355995	profile #4
Moxd1	0.266155328	0.276212988	0.741174836	profile #4
Ifngr1	19.57381242	20.71029852	42.70093359	profile #4
Cdk17	3.375391948	3.553907139	7.201129357	profile #4
Snrpf	27.74804474	25.97614766	54.93941622	profile #4
Ntn4	4.157076068	3.762674582	8.722657527	profile #4
Fgd6	0.904237188	0.793407934	1.858468246	profile #4
Hsp90b1	42.51937351	41.90602328	130.6985169	profile #4
Igf1	0.845242247	0.836234659	10.95523826	profile #4
Dram1	1.760716279	1.395733533	8.457790679	profile #4
Rufy2	0.974467517	0.813142831	1.701827714	profile #4
Srgn	20.62020222	19.25416072	44.7519746	profile #4
Sgpl1	2.951295608	2.678684932	22.64748658	profile #4
Unc5b	8.194842364	6.634598094	22.08459401	profile #4
Slc29a3	0.824319638	0.728489648	7.09551891	profile #4
Vsir	7.929489649	6.368637474	27.67848806	profile #4
Slc16a7	0.332307626	0.354381394	0.971738145	profile #4
Micu1	1.920097602	1.801031947	5.300901059	profile #4
Cand1	3.400590767	3.186834208	7.657949312	profile #4
Tbk1	4.800046669	4.52005318	10.55464612	profile #4
Plek	1.005611483	1.731600102	30.02575923	profile #4
Srgap1	0.358846281	0.299859869	0.833578204	profile #4
Egfr	0.465869948	0.442594973	2.519379306	profile #4
Lgr5	0.00450941	0.005079301	0.012333496	profile #4
Slc1a4	0.530516164	0.494988309	3.987541294	profile #4
Dock2	0.245850646	0.33245648	6.076303758	profile #4
Gamt	4.892428896	3.886558764	9.329435192	profile #4
Actr2	15.10118358	16.37550372	68.80257355	profile #4
Tcf3	8.961264626	8.026288517	20.18981173	profile #4
Yeats4	13.44379199	12.86131082	26.98818749	profile #4
Rab36	0.149909597	0.13508391	0.373997578	profile #4
E2f7	0.073084162	0.109950604	0.885436139	profile #4
Csrp2	6.054731918	4.843872383	85.63312803	profile #4
Osbpl8	6.63204096	7.061183345	16.76181753	profile #4
Jsrp1	0.277776972	0.331331259	1.128442858	profile #4
Llph	7.363939495	6.143248191	19.79254073	profile #4
Irak3	1.791118659	1.744416606	4.118506454	profile #4
Helb	1.842972941	1.939058173	4.166168751	profile #4

Prmt2	4.729394821	4.222321781	10.95799062	profile #4
Dip2a	2.112470358	1.903572999	3.989487461	profile #4
4930404N11Ril	0.048610208	0.062235625	0.464065675	profile #4
Col6a2	10.09522121	9.227369529	60.24450431	profile #4
Txnrd1	6.832300178	6.476001786	18.33968412	profile #4
Ppm1m	4.116083288	4.34289838	13.35097749	profile #4
D10Wsu102e	0.074832667	0.069201131	0.178363566	profile #4
Aldh1l2	0.666231575	0.757377075	1.647308305	profile #4
Pofut2	5.91629603	5.9034373	12.89703486	profile #4
Slc36a1	1.867508261	1.966036232	7.43448892	profile #4
Sumo3	8.991350294	8.589936133	17.68584658	profile #4
Rel	1.512649273	1.703815028	3.063323624	profile #4
Il9r	0.017205675	0.017855855	0.042649081	profile #4
Pus10	1.402734323	1.388818065	3.092820602	profile #4
Rhbdf1	7.982451494	7.365140787	14.78297971	profile #4
Mpg	0.614193301	0.566365753	1.869360277	profile #4
Xpo1	5.594627822	5.720867568	12.8602762	profile #4
Asb3	1.190077272	1.266333853	2.404969283	profile #4
Tpgs1	17.05972121	15.87817064	34.77513537	profile #4
Shc2	0.63123102	0.711004802	2.506451597	profile #4
Wdpcp	0.39445619	0.46890576	0.806561595	profile #4
Fstl3	0.800686666	1.101903422	6.279536321	profile #4
Nudcd2	7.293329791	6.233940174	21.94079666	profile #4
Hnrnpab	54.13860547	57.47477158	118.0681923	profile #4
Cnot6	3.749565814	4.1328575	12.52994745	profile #4
Gfpt2	2.16179972	2.77274662	6.750510643	profile #4
Rack1	46.0190583	46.00293754	125.6618859	profile #4
Rnf130	29.61238446	29.48010722	63.94551846	profile #4
Ltc4s	1.808833019	1.719519996	4.036596678	profile #4
Rad50	1.834161348	1.700922152	4.121483661	profile #4
Mrnip	0.703689175	0.534451102	1.520153587	profile #4
Cdkn2aipnl	7.966244208	7.872366413	20.34672555	profile #4
Itk	0.034579446	0.038949531	0.10384294	profile #4
Havcr2	0.136081111	0.097390203	5.784447307	profile #4
Upp1	2.053421294	1.674879471	7.112783907	profile #4
Ascc2	3.99519307	3.822876028	7.805180209	profile #4
Zfp607a	0.397074116	0.427480005	0.942527877	profile #4
Tns3	1.842156182	1.866270078	11.88676874	profile #4
Pes1	11.1819856	10.03371593	20.84664521	profile #4
Myo1g	0.354804907	0.380909503	6.174579033	profile #4
Rtn4	15.6137659	16.64781163	90.06912244	profile #4
Rps27a	33.17355729	42.41950774	146.5554033	profile #4
Ppp4r3b	4.047160637	3.712867662	9.214115304	profile #4
Myl7	0.216397815	0.176725243	1.081612769	profile #4
Aebp1	4.380781398	3.430202999	35.28187651	profile #4
Polm	0.708566684	0.688214336	2.303215193	profile #4
Dbnl	13.58743369	13.24460027	40.21845385	profile #4
Xbp1	16.34495765	14.39782949	44.91226297	profile #4
Supt4a	34.78751849	31.00338361	63.37916411	profile #4
Ska2	3.81932725	2.852071045	5.983926696	profile #4
Prr11	0.073791305	0.069298834	4.040895285	profile #4
Galnt10	4.147124958	3.472601552	11.82158332	profile #4
Rnft1	4.301817971	4.237515444	9.755896365	profile #4
Mfap3	2.81716307	2.348185221	7.087604152	profile #4
Prpsap2	2.660699334	2.391465445	6.888109955	profile #4
Acaca	0.628910883	0.662212308	1.320040724	profile #4
Llgl1	3.779305109	3.955987931	7.575166589	profile #4
Pctp	2.573418699	2.737845898	6.310873729	profile #4

Twistnb	1.478060614	1.577977432	2.962757967	profile #4
Pdia6	24.52354479	23.3746108	132.5475912	profile #4
Pik3cg	0.206515234	0.181063864	2.969933133	profile #4
Cyria	0.621682724	0.70942486	1.894582575	profile #4
Sdc1	2.446903093	2.600538166	21.57647706	profile #4
Nrcam	0.010562133	0.015611303	0.088721258	profile #4
Arsg	0.414736452	0.316605425	1.740912233	profile #4
Gna13	4.753503873	4.709616239	13.69384285	profile #4
Fam20a	1.294156811	1.203213876	4.754539215	profile #4
Klhl29	0.131471632	0.14068243	0.923254079	profile #4
Rnaseh1	3.05529104	2.852203516	5.711001464	profile #4
Fkbp1b	0.375146065	0.714863128	5.290123449	profile #4
Cmpk2	3.445603133	3.507709268	8.646178544	profile #4
Rsad2	5.605710097	5.231434099	12.48983196	profile #4
Rnf144a	2.767958103	2.691929946	5.887190095	profile #4
Id2	2.240189247	3.17358292	18.83114699	profile #4
Rrm2	0.577051025	0.46222564	8.689281374	profile #4
Cenpo	0.275400955	0.294298749	1.189453085	profile #4
Adcy3	0.481892114	0.584274139	2.567220641	profile #4
Sh3yl1	0.499705326	0.34572569	0.974412141	profile #4
Sntg2	0.027146386	0.015288547	0.301001635	profile #4
Pxdn	9.373972323	7.669334539	28.42276436	profile #4
Ddx52	4.40342569	3.911610647	9.067542687	profile #4
Ace	12.14213805	14.64418363	24.59030793	profile #4
Cdc27	3.059464604	3.412737548	8.907799329	profile #4
Itgb3	0.414428443	0.461678443	4.711942718	profile #4
Mrc2	4.736645731	4.40076323	35.63802714	profile #4
Tmem132e	0.040084942	0.04644133	0.422599407	profile #4
Rnf135	1.946548094	1.90960128	5.363357051	profile #4
Adap2	5.401165262	6.47131958	14.98761804	profile #4
Ern1	1.334990918	1.221291967	3.201298531	profile #4
Cacng1	0.034073849	0.04174055	0.437939975	profile #4
Slc9a3r1	4.620608578	4.874862549	35.63703371	profile #4
Nt5c	9.094515539	9.530656476	39.01371784	profile #4
Jpt1	25.15607203	24.841862	56.64883478	profile #4
Nup85	4.834954552	4.836058867	9.748317357	profile #4
Gga3	2.473761409	2.16216048	4.786926119	profile #4
Sap30bp	5.5172334	4.203896997	11.15238951	profile #4
Galk1	10.53361767	10.3822938	31.15255074	profile #4
Trim47	14.73653285	16.31009923	48.93926327	profile #4
Ten1	2.171710874	2.560831693	7.517500791	profile #4
Camkk1	0.445217607	0.531408255	2.615597628	profile #4
P2rx1	0.081063308	0.117777218	0.369726098	profile #4
Ankfy1	2.270139711	2.524340556	7.159937285	profile #4
Galr2	0.056536549	0.063681531	0.204155039	profile #4
Spns3	0.008538427	0.009892391	0.124233306	profile #4
Txndc17	25.68160355	23.31064687	53.12870056	profile #4
Rhbdf2	3.095549623	2.8704442	6.398566701	profile #4
4933427D14Ril	0.652157806	0.527175542	1.328737031	profile #4
Pimreg	0.084140445	0.041228902	10.52591357	profile #4
Snhg16	0.897900541	0.901451528	1.888313771	profile #4
Mxra7	12.05993238	11.34178356	39.65565024	profile #4
Mfsd11	3.401354687	2.902402831	6.896199422	profile #4
Pld2	1.715399184	1.776156534	4.93205927	profile #4
Slc46a1	0.586071082	0.513029677	1.524341558	profile #4
Vmo1	0.126661419	0.081082277	0.318209194	profile #4
0610010K14Ril	0.381587567	0.316118243	0.721238171	profile #4
Blmh	5.499781295	4.938167079	10.36480385	profile #4

Nxn	7.259206764	7.250392692	21.6391533	profile #4
Rflnb	14.39546779	14.2736129	28.92032454	profile #4
Abcc3	0.3062281	0.3333025	8.368306488	profile #4
Xylt2	4.813070271	4.820187492	14.03031702	profile #4
Lrrc59	14.18694872	14.35805089	71.71825267	profile #4
Dlg4	1.019510863	0.868550296	4.772907964	profile #4
Dvl2	1.750172016	1.365846492	3.762002675	profile #4
Aloxe3	0.006652843	0.008149749	0.032981867	profile #4
Tmem107	2.56948772	3.000350976	5.360659343	profile #4
Aurkb	0.015710994	0.055486039	6.080728174	profile #4
Ctc1	1.6002469	1.641410121	3.215256477	profile #4
Myh10	4.22659988	3.872694676	12.54841315	profile #4
Pik3r5	0.220319121	0.137445416	9.838363679	profile #4
Top2a	0.067833747	0.122507647	11.74325864	profile #4
Acly	7.982302333	7.814258513	17.95141178	profile #4
Gfap	0.030269016	0.024719747	0.101392403	profile #4
Map3k14	0.948626349	0.81522968	3.020259168	profile #4
Gosr2	14.55427798	14.66965616	34.58445239	profile #4
Scfd1	1.458271148	1.486532471	3.480486528	profile #4
Gtf2a1	2.993601346	2.271660414	5.65917149	profile #4
Dnaaf2	0.166476667	0.123029963	0.255475751	profile #4
Pole2	0.20885097	0.108574689	1.582167734	profile #4
Sec23a	7.506901154	8.796790164	16.47176849	profile #4
Galc	0.517712271	0.661947703	2.963445285	profile #4
Ttc8	0.528883431	0.4686817	1.590919439	profile #4
Polr2h	4.92253215	3.951081102	9.20873764	profile #4
Sptlc2	3.054174988	3.175907345	10.35219855	profile #4
Sgpp1	4.083300478	4.687700986	8.605230018	profile #4
Rab15	0.221369243	0.134011789	2.805669633	profile #4
Fut8	1.886058889	1.728561635	3.508406633	profile #4
Sav1	3.153175584	3.363412954	6.839502263	profile #4
Nin	1.274958693	1.22331086	3.417663736	profile #4
Pygl	0.572950154	0.592682631	4.443077917	profile #4
Serpina3n	2.16464029	2.32379995	64.83475317	profile #4
Hif1a	8.264786509	8.877625932	28.79041859	profile #4
Vrk1	1.707832191	1.723338727	4.094476603	profile #4
Rdh12	0.079254564	0.059543589	0.475672873	profile #4
Arg2	0.032933935	0.061857783	0.590994402	profile #4
Zfp36l1	14.14035552	16.89288217	33.70114942	profile #4
Galnt16	0.828256577	0.83574986	3.922241695	profile #4
Erh	11.27943741	10.69363231	35.32711618	profile #4
Smoc1	1.35040846	1.238075735	2.751978282	profile #4
Esyt2	4.094455667	4.302725941	8.654794948	profile #4
Cdca7l	0.014435132	0.040668929	1.902758235	profile #4
Efcab11	0.008858478	0.019639837	0.515150715	profile #4
Fbln5	3.428984092	3.296561237	7.802605211	profile #4
Tc2n	0.027925352	0.021735461	0.063140722	profile #4
Atxn3	2.009219736	1.975126468	4.666877417	profile #4
Lgmn	28.37247237	28.61408418	363.3156279	profile #4
Pfkp	4.750923245	4.458286913	11.06486524	profile #4
Ifi27l2b	0.08469275	0.09539606	1.134722921	profile #4
Akr1c13	0.327961356	0.436267336	3.906916558	profile #4
Gdi2	12.98460452	13.56247374	35.16283471	profile #4
Abcd4	1.126948067	0.893700476	2.123679759	profile #4
Npc2	11.80520845	11.00584237	65.66096323	profile #4
Erg28	1.343704529	0.972510458	4.55284363	profile #4
Tgfb3	5.007436953	4.2842504	31.65710518	profile #4
Vash1	7.444000688	5.811823101	15.69835286	profile #4

Hhipl1	1.306912471	1.545549998	4.382867195	profile #4
Evl	6.45465343	6.401481211	17.33967354	profile #4
Meg3	0.701108663	0.615288138	3.84667049	profile #4
Hsp90aa1	24.82012504	23.08959583	82.78089546	profile #4
Traf3	2.408738306	2.46119459	5.17272072	profile #4
Exoc3l4	0.331426978	0.35681942	0.843940006	profile #4
Tnfaip2	5.456403166	6.185430753	13.80884983	profile #4
Gpr132	0.15589942	0.220758817	2.016285966	profile #4
Gpr137b	1.298473321	1.078776454	5.599063603	profile #4
Gli3	0.202727364	0.288153006	1.207413924	profile #4
Sfrp4	0.040735331	0.039115068	0.276314315	profile #4
Aoah	0.214312368	0.21117548	2.59130233	profile #4
Trim27	2.390999128	2.350767051	7.572443513	profile #4
Carmil1	0.304198584	0.412736517	1.274495417	profile #4
Irf4	0.147560683	0.11696378	0.31843545	profile #4
Elovl2	0.011670345	0.013145221	0.068959535	profile #4
Nedd9	3.872423261	4.766952781	9.594006078	profile #4
Dek	10.51338049	10.77052965	28.83775153	profile #4
Id4	0.276423986	0.309819261	0.828983546	profile #4
Susd3	0.212090111	0.288089301	5.924411362	profile #4
Aspn	9.310034351	9.166086144	84.1175324	profile #4
Ogn	34.4933037	35.16404989	77.88451514	profile #4
Nxnl2	0.048568601	0.046636767	0.208078756	profile #4
Ripk1	0.887528054	1.132744698	4.551450179	profile #4
Ly86	0.86656167	0.826905029	20.10016733	profile #4
Ssr1	4.830692407	4.575866735	16.13360904	profile #4
Riok1	0.979473805	0.967741272	2.676625457	profile #4
Slc35b3	0.721910936	0.764295528	2.382518617	profile #4
Sema4d	5.008792341	5.16569886	13.41352933	profile #4
Syk	2.204969692	2.758702776	12.11101686	profile #4
Aopep	5.040733421	3.72681365	7.717353392	profile #4
Ror2	0.43238017	0.454727965	2.09536521	profile #4
Sptlc1	5.529219755	5.351206566	12.44667241	profile #4
Sfxn1	0.836316455	0.830514529	3.242310405	profile #4
Ctsl	22.5324416	22.30443505	93.8816603	profile #4
Cdk20	0.608018942	0.54107984	2.594445272	profile #4
Lman2	9.455614066	10.14378598	19.6890007	profile #4
Mxd3	0.039075211	0.093217511	6.676674373	profile #4
Prelid1	13.40500538	12.79232044	96.65373075	profile #4
Pdlim7	8.039460714	7.989371642	24.5228185	profile #4
Ddx41	4.102189564	3.533974031	12.90879578	profile #4
Caml	5.922253293	5.091689765	10.77165592	profile #4
B4galt7	4.283242261	3.675454454	8.077942555	profile #4
Zfp729a	0.510197912	0.373490561	1.041717441	profile #4
Hnrnpk	31.06636436	28.63093795	57.39021296	profile #4
Golm1	1.520172054	1.699648734	5.409286284	profile #4
Nkd2	0.345264229	0.35691915	3.331342131	profile #4
Trip13	0.148999494	0.075077688	3.135606591	profile #4
Cep72	0.072855871	0.073289336	0.429179926	profile #4
Rhobtb3	2.121677188	2.03222237	4.437947794	profile #4
Spata9	0.082798905	0.03249093	0.229520477	profile #4
Arsk	0.700398537	0.6633943	1.624070751	profile #4
Srd5a1	0.307226873	0.22774041	0.720791891	profile #4
Mctp1	0.109064264	0.145550389	1.294555629	profile #4
Lpcat1	3.868095366	3.072661367	6.44069068	profile #4
Clptm1l	16.16047783	15.7530072	34.6621235	profile #4
Vcan	0.745699696	0.704544545	8.360060934	profile #4
Zcchc9	3.146473973	2.69658345	5.894262465	profile #4

Cd180	0.141577634	0.181261776	8.956725581	profile #4
Slc30a5	3.307679574	3.115053451	7.642204199	profile #4
Serf1	1.702329476	1.902555064	6.875668433	profile #4
Smn1	2.12286653	2.027703844	4.924008749	profile #4
Btf3	66.09573627	60.25247752	136.8551362	profile #4
Hexb	5.946562276	6.024805235	57.65004661	profile #4
Polk	1.050217556	0.898759161	1.97572845	profile #4
Hmgcr	0.885644428	1.063531981	3.953594677	profile #4
F2rl2	0.018499036	0.020836909	0.067461232	profile #4
Iqgap2	0.481212144	0.689234476	5.781703132	profile #4
F2rl1	0.543428782	0.513541248	1.213974618	profile #4
Aggf1	9.327026797	8.017605	16.35604584	profile #4
Ap3b1	5.839124852	6.088911266	12.94301131	profile #4
Dimt1	1.081696422	1.169820462	2.355843955	profile #4
Kif2a	1.977130668	1.900079709	4.220975041	profile #4
Elovl7	0.023438654	0.033017536	0.085257544	profile #4
Depdc1b	0.012703951	0.016264863	0.944708962	profile #4
Thbs4	0.931648847	0.993711708	19.4006795	profile #4
Serinc5	3.042212028	3.002181675	6.067169098	profile #4
Dhfr	0.484746012	0.427990682	2.255541987	profile #4
Erbin	6.4807213	6.547114859	13.40654483	profile #4
Nln	1.454725212	1.468050546	3.030220458	profile #4
Ppwd1	0.807822365	0.673840128	1.67209202	profile #4
Cenpk	0.017696268	0.008443215	0.731965055	profile #4
Rnf180	0.06102633	0.107431473	1.376564628	profile #4
Parp8	1.212541297	1.079685095	2.736486398	profile #4
Emb	0.254679169	0.326164104	4.38711935	profile #4
Gpx8	19.72658564	16.02939619	70.92246322	profile #4
Comtd1	0.524683589	0.337709776	1.74878497	profile #4
Dlg5	1.240683489	1.289987198	3.333715552	profile #4
Nid2	4.835674202	5.616839699	11.76998246	profile #4
Mss51	0.206044506	0.145125269	0.608954111	profile #4
Plau	2.085659265	1.960254016	20.72095632	profile #4
Ero1a	2.970300695	2.926237865	6.145805618	profile #4
Mapk1ip1l	4.831355649	4.87862989	12.65543716	profile #4
Rnase10	0.141230206	0.130900396	0.438027651	profile #4
Rnase4	22.14823597	26.04404795	82.23494855	profile #4
Arf4	15.6851185	15.32329991	58.20814416	profile #4
Rnase6	0.277898935	0.254138479	2.832536761	profile #4
Gpr65	0.268446136	0.405588535	5.955304057	profile #4
Eaf1	5.20947345	4.41625457	12.29365609	profile #4
Mettl6	3.838048759	3.571753693	7.535178629	profile #4
Btd	4.177304984	4.630682049	9.608498598	profile #4
Glt8d1	7.592650143	6.832573648	17.05622504	profile #4
Spcs1	65.42534927	57.46918134	115.5903899	profile #4
Nek4	1.210054588	1.305795618	2.515724448	profile #4
Ebpl	0.309601052	0.266212794	0.781173184	profile #4
Rnaseh2b	1.458827919	1.274250251	3.818885982	profile #4
Ctsb	70.59336684	69.36272341	650.9955998	profile #4
Cryl1	2.168945958	2.226523782	6.479896304	profile #4
Prkcd	2.775185062	2.671054259	19.49020973	profile #4
Anxa8	0.221575155	0.138654114	1.963916195	profile #4
Tkt	2.765851426	2.804314799	18.0893463	profile #4
Pinx1	1.26843666	1.607418706	3.302722298	profile #4
Ska3	0.104317172	0.019273178	2.291750954	profile #4
Zdhhc20	2.245890171	2.262293819	8.923075062	profile #4
Mtmr6	7.483195606	7.54338377	17.95536097	profile #4
Esd	21.8607224	20.33808916	51.8792659	profile #4

Lcp1	2.469255008	2.323572014	46.00431032	profile #4
Slc25a30	3.392684771	2.299580815	6.267191619	profile #4
Nufip1	1.443789214	1.344776366	3.132389304	profile #4
Enox1	0.225132474	0.202192649	0.507088416	profile #4
Epsti1	0.495931116	0.634940365	10.96408523	profile #4
Diaph3	0.030290961	0.0909842	2.957512881	profile #4
Esco2	0.013924365	0.00784205	1.100061818	profile #4
Clu	119.4651934	120.4681163	422.534758	profile #4
Stmn4	0.098183003	0.161359707	1.387479656	profile #4
Dpysl2	4.044781474	3.171866227	9.414486511	profile #4
Bora	0.413959656	0.366917777	3.678227114	profile #4
Tnfrsf10b	0.517952645	0.46183967	1.547003614	profile #4
Bin3	4.785264064	3.684700673	17.17950305	profile #4
Pdlim2	1.719318768	1.639720047	7.931878419	profile #4
Bmp1	11.22576864	9.58095883	69.31000506	profile #4
Dok2	1.495844302	1.389407116	8.311773261	profile #4
Rcbtb2	2.106397393	1.954885854	5.260728358	profile #4
Obi1	0.966176433	0.741001916	2.598293903	profile #4
Cln5	11.13690089	11.25676481	24.38916845	profile #4
Acod1	0.017645668	0.039751389	0.782005856	profile #4
Tgds	2.555373165	2.359444273	6.230999625	profile #4
Dnajc3	9.595802832	10.72948174	23.15121722	profile #4
Nup155	1.515663545	1.668151954	4.272001164	profile #4
Osmr	1.634565032	1.529404652	5.046297408	profile #4
Fyb	0.426223284	0.442324268	12.54259048	profile #4
Dab2	4.028309962	3.421225909	27.86961742	profile #4
Rab2b	0.333542551	0.225417017	0.733608194	profile #4
Haus4	2.038075617	1.957010411	8.083105292	profile #4
Ajuba	3.972750062	3.631156029	8.444218449	profile #4
Slc7a8	0.517072554	0.633415172	12.37603928	profile #4
Fbxo4	4.181435197	3.565585021	7.895188629	profile #4
Slc22a17	2.560172926	2.830195796	6.581021103	profile #4
Efs	1.179472839	1.137864472	6.433339463	profile #4
Cpne6	0.011047606	0.010375019	0.039649519	profile #4
Tgm1	0.056631054	0.058771067	1.209321965	profile #4
Ripk3	1.626087868	1.52586606	13.18264734	profile #4
Ropn1l	0.305173108	0.171870154	1.530220891	profile #4
Tars	10.44581915	10.36350079	25.29849594	profile #4
Rai14	1.446199563	1.392646386	11.35915556	profile #4
Mtdh	4.098114563	4.505738074	8.384632245	profile #4
Pabpc1	14.60910351	14.41532669	72.95689223	profile #4
Ywhaz	18.66892735	20.19391934	53.73211751	profile #4
Atp6v1c1	19.0568357	18.40795578	38.27310187	profile #4
Baalc	0.536166764	0.139106951	2.357253616	profile #4
Dcaf13	9.54885006	9.142768217	21.45392925	profile #4
Lrp12	0.873966107	0.798130394	7.795466829	profile #4
Utp23	1.666157688	1.550775821	3.595391568	profile #4
Shcbp1	0.036762977	0.025966937	1.729440204	profile #4
Matn2	4.857104407	5.164874523	22.91804706	profile #4
Pop1	0.740143251	0.686876768	1.836909735	profile #4
Stk3	1.840640459	2.306073127	5.914286691	profile #4
Zfat	0.334044836	0.370299854	0.801172387	profile #4
Washc5	5.349359626	5.256834265	12.49215995	profile #4
Sqle	0.306947255	0.25380753	1.943267857	profile #4
Atad2	0.778823973	0.604029432	6.055710114	profile #4
Tbc1d31	0.63533626	0.638283699	1.990553907	profile #4
Derl1	20.23571018	20.74208482	43.08935976	profile #4
Has2	0.065543434	0.135348929	1.722936289	profile #4

Mtbp	0.388449379	0.367821515	1.405473574	profile #4
Col14a1	1.549101827	1.635458188	41.40945818	profile #4
Sla	0.161235229	0.278093119	2.669633334	profile #4
Cyrib	4.692025893	4.605154764	20.5913509	profile #4
Gtse1	0.046921981	0.064926802	3.953477241	profile #4
Rangap1	10.04682586	9.222074659	25.67994227	profile #4
Slc25a17	8.603554791	9.380835147	17.96493092	profile #4
Nptxr	0.546210073	0.544637865	1.781314566	profile #4
Dscc1	0.010618573	0.009972106	0.743527501	profile #4
Fam118a	2.711916482	2.032039107	4.947189016	profile #4
Sh3bp1	1.344035143	1.312664329	7.331802654	profile #4
Parvg	0.269618959	0.299768261	7.81831514	profile #4
C1qtnf6	3.930914219	3.934896654	68.23195223	profile #4
Myh9	14.43887248	12.28577119	55.5956804	profile #4
Adamts20	0.0522726	0.040562146	0.196984997	profile #4
Twf1	4.958595297	4.977719015	26.23840983	profile #4
Naga	8.364818126	8.174517611	20.07582301	profile #4
Septin3	0.077051726	0.094388558	0.404358043	profile #4
Srebf2	7.41293002	5.862325938	15.50733092	profile #4
Slc38a4	1.725531499	1.764770522	5.349366691	profile #4
Vdr	0.0655357	0.055363483	0.718021321	profile #4
Nckap1l	1.587926584	1.647932246	35.61294431	profile #4
Pde1b	0.532727147	0.652997978	4.685381214	profile #4
Shisa9	0.018154379	0.010224347	0.125810891	profile #4
Litaf	5.41143686	5.984705355	56.20410452	profile #4
Nubp1	1.532892228	1.528101063	5.025703388	profile #4
Ciita	0.210604142	0.228153859	2.515245627	profile #4
Bcl6	1.87901611	1.700738189	3.638413418	profile #4
Il1rap	0.309650413	0.399265261	1.35623251	profile #4
Anks3	5.536255594	4.432715486	9.817708376	profile #4
Zfp263	2.569873629	2.525400205	5.855555931	profile #4
Atp13a3	3.717557047	4.343478907	9.198468974	profile #4
Adck5	1.702985896	1.673132307	3.778688083	profile #4
Bop1	9.591072776	8.687021196	17.61801921	profile #4
Slc52a2	2.24565964	2.162593345	4.876097815	profile #4
Gsdmd	5.62920665	5.222385099	18.27724806	profile #4
Rhpn1	0.032907635	0.037066439	0.136831084	profile #4
Ly6c2	0.108725423	0.099429361	3.246569944	profile #4
Ly6i	0.120115713	0.09019714	0.628931112	profile #4
Ly6e	34.61353037	32.13943736	86.53507466	profile #4
Mapk8ip2	0.014932715	0.018292613	0.037515189	profile #4
Arsa	3.758539944	3.686775863	10.686849	profile #4
Rabl2	1.191629861	0.940029318	2.223895963	profile #4
Alcam	0.283987546	0.2782039	3.334974514	profile #4
Cblb	1.763444262	1.930278812	4.918423097	profile #4
Bbx	0.943572874	1.001139222	1.938168283	profile #4
Nectin3	0.628166135	0.743766254	2.031122568	profile #4
Slc35a5	0.696305863	0.730204222	2.080867785	profile #4
Ccdc80	17.9548426	19.41274216	85.51517699	profile #4
Cd200r1	0.115608797	0.153787453	8.831009123	profile #4
Mcm4	1.676510572	1.870521438	9.639712897	profile #4
Snai2	1.968252571	1.745498954	4.906343937	profile #4
Parn	4.278370036	3.68279086	8.070067257	profile #4
B3gnt5	0.147402014	0.118978333	0.885024636	profile #4
Ccdc191	0.820648724	0.616240505	1.411648952	profile #4
Trmt2a	4.4676965	3.916362717	8.653645041	profile #4
Tbc1d23	3.628183026	4.144420106	7.734488555	profile #4
Klhl22	3.185385878	2.985564572	6.125726418	profile #4

Tmem45a	0.821641354	0.648262426	12.8722133	profile #4
Thap7	4.52785542	4.298277521	9.898338906	profile #4
Snap29	6.158155925	5.33908702	12.55203859	profile #4
Serpind1	0.128789571	0.174080798	0.519999548	profile #4
Ccdc116	0.010662195	0.012009663	0.136505248	profile #4
Sdf2l1	5.492624438	4.966801693	51.18480906	profile #4
Senp5	1.838258229	2.117445516	3.738445013	profile #4
Ncbp2	14.85926036	11.6066201	23.87919097	profile #4
Pak2	5.43562932	5.396607222	13.04075036	profile #4
Tnk2	4.655046256	3.96559606	8.102958627	profile #4
Snx4	12.13027696	11.48472567	27.55094372	profile #4
Umps	2.165438532	2.364283461	8.199589328	profile #4
Fstl1	18.3669	16.82076083	264.0783769	profile #4
Itgb5	36.00065367	36.40049168	117.9558028	profile #4
Gtf2e1	1.598049946	1.24839614	3.116536285	profile #4
Hcls1	3.79441969	4.108774414	43.43401954	profile #4
Ap2m1	29.99070386	28.55151824	66.66896076	profile #4
Ece2	0.669543058	0.517596844	2.31384193	profile #4
Pdia5	2.201589916	1.989625179	18.98420137	profile #4
Hspbap1	0.453770471	0.317536618	1.084803058	profile #4
Senp2	5.020422082	4.747503362	9.939139726	profile #4
Tra2b	8.063974512	8.785986921	20.99171535	profile #4
D16Ertd472e	0.613513686	0.765112956	2.248451913	profile #4
Fetub	0.169363364	0.136262276	0.428897305	profile #4
Samsn1	0.084478194	0.155858692	1.852051845	profile #4
Rfc4	3.079507364	2.786821141	7.388095591	profile #4
Robo1	0.257034602	0.379587425	1.419356898	profile #4
St6gal1	2.803028873	2.593577263	9.325989083	profile #4
App	96.58520659	98.42850723	227.6047004	profile #4
Vps26c	6.81737829	5.790202465	15.44051585	profile #4
Cd86	0.682700834	0.684625876	2.75473452	profile #4
Parp9	3.42683267	3.135313297	9.293386976	profile #4
Pros1	6.304367138	5.801709228	20.5927142	profile #4
Chaf1b	0.120315866	0.132472876	2.160166731	profile #4
Cbr3	2.690044431	2.840790326	11.80670337	profile #4
Rcan1	22.52018763	24.63451692	73.79324529	profile #4
Runx1	0.179919831	0.165852916	3.854084081	profile #4
Itsn1	2.184660032	2.3691045	5.128736508	profile #4
Donson	1.201361663	0.872731681	3.871284433	profile #4
Ifnar1	4.692229028	4.068705604	16.42170618	profile #4
Ifnar2	7.286931522	6.713195302	20.36965441	profile #4
Synj1	2.272724977	2.67675496	4.831662378	profile #4
Mis18a	1.11701599	1.187850917	6.278701411	profile #4
Dhh	0.486276935	0.353001755	1.176689546	profile #4
Tuba1b	63.55901581	61.8394415	229.588689	profile #4
Fmnl3	10.83714696	10.12480411	22.06081407	profile #4
Nckap5l	1.673586408	1.546820304	5.804857016	profile #4
Aqp2	0.099639107	0.038479724	1.389333699	profile #4
Racgap1	0.119583819	0.153940641	5.949710586	profile #4
Cers5	6.99735461	6.396779322	13.60501817	profile #4
Lima1	5.282388042	5.010092006	14.37364176	profile #4
Scn8a	0.005965959	0.003456	0.014455871	profile #4
Pcdhgc4	0.066168103	0.037265151	0.160492071	profile #4
Krt7	0.033330729	0.012520611	0.394120113	profile #4
Krt18	0.212791185	0.272436449	5.515750757	profile #4
Soat2	0.076750653	0.059738219	1.156915661	profile #4
Prr13	18.01602401	16.82397043	61.16657047	profile #4
Map3k12	0.628390801	0.599633623	1.43223738	profile #4

Akirin1	9.869412393	10.61317646	20.33364825	profile #4
Fhl1	21.54713319	21.80078426	59.8435818	profile #4
Rfc2	2.984410711	2.508581873	7.250412716	profile #4
Gzma	0.373993308	0.570053095	2.139211746	profile #4
Nagpa	6.250777396	5.345379346	15.96340486	profile #4
Slc38a1	1.340383135	1.45800364	4.047047796	profile #4
Vwa5a	5.683110397	6.195023566	22.26542765	profile #4
P3h3	3.650947146	3.60475709	22.91307855	profile #4
Serping1	35.81332208	34.9187363	80.04831683	profile #4
Serinc2	0.10140073	0.20233097	3.852575021	profile #4
Ccl25	0.25489162	0.172038583	0.453426464	profile #4
Kcnk5	0.179607979	0.183785946	0.535631638	profile #4
Creld2	10.63955923	9.03404799	34.64856332	profile #4
Cd4	0.286982813	0.324329106	1.287259683	profile #4
Mx2	0.533540675	0.490468785	1.798101287	profile #4
Clec4n	0.651895606	0.839784743	30.87077359	profile #4
Tmem176a	2.453933538	2.359685781	18.15246287	profile #4
Slc17a9	0.226090634	0.207030266	0.988992195	profile #4
Nfatc4	2.255512431	1.901168707	7.486056539	profile #4
Pisd	2.546092782	2.250569018	5.097284923	profile #4
Prph	0.07445242	0.094726985	0.275178438	profile #4
Cdca3	0.0593974	0.138694386	9.510881699	profile #4
Hes7	0.117629179	0.073244539	0.307338365	profile #4
Pigx	4.988063369	4.68203782	9.606251118	profile #4
Agpat4	1.559890949	1.312516985	6.581665474	profile #4
Acat2	2.107461112	1.47783923	3.081818953	profile #4
Thbs2	5.274864216	5.308471777	33.02652761	profile #4
Tnfrsf12a	47.91872715	35.03963969	175.7023063	profile #4
Pkmyt1	1.064477725	1.176145929	4.759838193	profile #4
Flywch2	0.427291535	0.5234332	1.052602215	profile #4
Pla2g7	1.08467715	1.112669431	22.74716093	profile #4
Adgrf4	0.045377047	0.053418912	0.097833729	profile #4
Cenpq	1.568967259	1.799503429	3.710872916	profile #4
Spats1	0.073376271	0.046971802	0.368683755	profile #4
Sgo1	0.012343742	0.027366901	1.878323787	profile #4
Nfkbie	0.615003423	0.449936332	6.233221474	profile #4
Polh	2.288325151	2.475659706	6.476263968	profile #4
Rsph9	0.371919673	0.272096329	0.587316444	profile #4
Rrp36	10.25671316	8.252886581	17.25008044	profile #4
Ptk7	1.22023617	1.139472866	8.63438201	profile #4
Cnpy3	8.581535122	7.93026746	22.70925441	profile #4
Guca1a	0.195445405	0.119710555	4.861038169	profile #4
Foxp4	2.963352671	3.217360901	8.355820937	profile #4
Trem2	0.484939867	0.535783949	43.04401478	profile #4
Nfya	2.970294221	2.420829693	7.612172922	profile #4
Stk38	4.269152128	3.697099654	9.096537943	profile #4
Mtch1	52.84612794	52.51832594	110.6137772	profile #4
Fgd2	0.967700597	0.769272073	7.510660651	profile #4
Abcg1	1.110390363	1.102685543	11.42284918	profile #4
Rsph1	0.255934414	0.268731684	1.135640309	profile #4
Wdr4	1.70797048	1.528588712	3.826492471	profile #4
Lpin2	0.575104829	0.699297352	2.176596682	profile #4
Emilin2	11.7664656	13.79213396	23.86656846	profile #4
Smchd1	1.142799756	1.168486926	2.37124922	profile #4
Ndc80	0.12820443	0.11843241	6.730682434	profile #4
Slc30a6	3.724580844	2.98663634	7.152692823	profile #4
Prkd3	2.898825971	2.41337996	5.080784785	profile #4
Eif2ak2	2.263083709	2.080055765	8.071672123	profile #4

Qpct	2.941915545	2.272598351	7.33535039	profile #4
Man2a1	6.146476205	7.634104574	20.137473	profile #4
Cyp1b1	1.265618735	1.572566088	13.19111985	profile #4
Hnrnpll	2.178902565	2.384431371	5.023923856	profile #4
Srsf7	20.2076196	18.64332228	47.62235323	profile #4
Washc2	2.703068705	2.82383581	6.284084859	profile #4
Tedc2	0.774302492	0.709848973	2.215679851	profile #4
Atp6v0c	8.138099842	7.308869926	18.57964849	profile #4
Six2	0.040168227	0.062529198	0.338531857	profile #4
Pigf	1.618429544	1.477480271	5.028811249	profile #4
Mcfd2	28.70636775	26.40923008	59.54312606	profile #4
C3	20.0502835	19.12477141	58.2782993	profile #4
Jpt2	1.715708646	1.912424145	17.57706358	profile #4
Ift140	0.621099558	0.72327531	1.787893419	profile #4
Pot1b	1.102152614	1.110788283	3.263467639	profile #4
Sox8	0.163287514	0.184144945	0.365476687	profile #4
Nme4	3.648280984	3.051563876	7.071999671	profile #4
Pgap6	2.185847292	2.170683026	12.43264631	profile #4
Axin1	4.938851285	5.624691742	9.887855092	profile #4
Rgs11	0.040473834	0.021001644	0.264372374	profile #4
Acsbg3	0.048550921	0.053820317	0.353205635	profile #4
Bambi	2.57543368	2.337752372	5.109915202	profile #4
Map3k8	0.8562238	0.648201824	2.229770443	profile #4
Dync2li1	1.478609439	1.850676356	3.876191747	profile #4
Celf4	0.064459639	0.061749678	0.235984623	profile #4
Slc39a6	1.238649667	1.387995697	8.024177507	profile #4
Elp2	7.690696092	7.205355992	17.53666434	profile #4
2700062C07Ril	2.577587052	1.698709878	5.031180808	profile #4
Thoc1	5.122444079	4.880651262	10.45258395	profile #4
Mib1	3.950509457	3.741436048	7.492012499	profile #4
Cyp4f41-ps	0.043667278	0.041008781	0.313441042	profile #4
Adamts10	8.585010195	8.447756186	17.45044512	profile #4
Myo1f	0.612514885	0.602188158	23.2452747	profile #4
Kifc5b	0.286438727	0.168125074	2.481256984	profile #4
Tapbp	22.63016941	22.81324636	51.23854527	profile #4
Rnf138	1.902120556	1.857123063	4.111154694	profile #4
Psmb8	6.360004987	6.352867495	22.11641026	profile #4
Btnl2	0.053715189	0.05125701	0.246867131	profile #4
Psd2	0.017788187	0.00923019	0.071502304	profile #4
Sting1	2.352431276	1.74593726	21.8867152	profile #4
Slc23a1	0.078860229	0.048301989	0.140772861	profile #4
Nelfe	6.041966932	5.361246206	15.7869169	profile #4
C2	0.175860483	0.080786074	0.565069245	profile #4
Stard4	2.386316031	1.755272721	4.316686633	profile #4
Bin1	4.305543071	4.290512829	29.61082603	profile #4
Map3k2	1.67707107	1.681425959	3.433320942	profile #4
Apom	0.04569061	0.051464903	0.224986438	profile #4
Aif1	1.66677168	1.701499566	32.55005875	profile #4
Ltb	0.396899501	0.466136748	2.623588622	profile #4
Tnf	0.344329409	0.265953825	1.513425446	profile #4
Atp6v1g2	1.47512614	1.142377318	2.385427962	profile #4
Npc1	3.139695617	3.558025054	8.895445624	profile #4
Impact	5.151722791	4.040684681	8.299235204	profile #4
Ttc39c	0.66060517	0.742334435	1.678763207	profile #4
Atat1	0.807406681	0.716188103	1.557379352	profile #4
Diaph1	4.827160804	4.383561066	12.54788302	profile #4
Trim26	3.72675847	3.393109228	7.254502429	profile #4
H2-M5	0.015815027	0.019373447	0.111249056	profile #4

Dcp2	2.161786187	2.126608299	4.918608943	profile #4
Mal2	0.015336302	0.01591584	0.032852074	profile #4
Ap3s1	5.546785535	5.270984406	26.14493407	profile #4
Hbegf	7.450784922	7.513615581	15.12085031	profile #4
Yipf5	7.015225233	7.092423726	25.69143257	profile #4
Dpysl3	6.294408457	6.122753721	45.81507478	profile #4
Rab27b	0.082349206	0.073228692	0.409766479	profile #4
Sec11c	4.651117329	4.045910953	12.19115671	profile #4
Pmaip1	0.069969143	0.150226044	2.360225264	profile #4
Lox	1.104854763	1.382006105	81.39417174	profile #4
Spire1	0.586367673	0.589355183	1.971503861	profile #4
Snx24	2.988131527	3.124541893	8.758200271	profile #4
Ppic	16.84458988	15.25305159	93.64715313	profile #4
Cep192	0.327745938	0.367493376	2.110424696	profile #4
Ldlrad4	3.768861358	4.163304195	10.12142648	profile #4
Me2	3.657754781	3.346216282	14.09243568	profile #4
Smad2	4.199390628	3.980924079	8.656646417	profile #4
Pcyox1l	0.764565337	0.788415073	2.991286725	profile #4
Lmnb1	1.640743329	1.70728995	25.5611483	profile #4
Megf10	0.107945398	0.067548519	0.892140168	profile #4
Prrc1	6.010098859	5.396911375	15.52174601	profile #4
Fbn2	0.142131516	0.13485941	2.988073227	profile #4
Rps14	354.8262252	344.7187211	809.6433788	profile #4
Cd74	54.13413883	53.34430922	245.5329219	profile #4
Tcof1	3.339943685	3.269651271	7.669175018	profile #4
Tmx3	6.619442283	7.009986328	13.89907762	profile #4
Csf1r	7.300676054	7.07024959	56.81005343	profile #4
Gnaq	4.714371588	5.320989988	10.46512505	profile #4
Psat1	0.550235785	0.742892057	6.664823932	profile #4
Cndp2	14.62602405	14.17328488	95.60537922	profile #4
Anxa1	7.773255195	8.375814291	89.6614091	profile #4
Incenp	1.071562695	0.863802233	14.6792706	profile #4
Rab3il1	2.15987952	2.054144059	24.33549215	profile #4
Fads2	3.160823961	2.55829712	13.44277692	profile #4
Tmem138	1.222611759	1.028062073	4.46501534	profile #4
Ms4a7	0.259850595	0.305902144	27.41987111	profile #4
Ms4a4c	0.158599388	0.201788495	11.7665209	profile #4
Ms4a6b	0.786449139	1.081241649	13.35942233	profile #4
Ms4a4d	8.361518927	9.779089915	17.18000806	profile #4
Ms4a6d	1.067079839	1.004952897	68.64955272	profile #4
Fam111a	1.547572251	1.327045916	12.93983457	profile #4
Lpxn	0.276325899	0.422886429	16.80322341	profile #4
Ostf1	9.368575366	8.565485391	31.24200986	profile #4
Trpm6	0.042671716	0.054291895	0.392225897	profile #4
Ccdc86	1.210421335	1.311696102	2.849877576	profile #4
Tmem132a	2.968856591	2.932591909	10.25012558	profile #4
Slc15a3	0.736301677	0.625150111	13.35673723	profile #4
Fen1	0.330100824	0.404942699	1.202144105	profile #4
Cemip2	2.422877038	2.775723026	6.672908109	profile #4
Atl3	3.952640242	4.067429819	12.86892197	profile #4
Lipo3	2.439894845	2.509178606	5.247752804	profile #4
Stambpl1	0.465472596	0.445082164	1.936338867	profile #4
Lipa	8.605285477	9.451315014	68.27699211	profile #4
Rcl1	3.417019004	2.833680964	6.421459853	profile #4
Jak2	5.609899622	5.584466511	11.83507389	profile #4
Cdca5	0.022090023	0.024487528	3.982291663	profile #4
Zfpl1	4.946980819	5.011099664	11.15565685	profile #4
Kif20b	0.047320341	0.074937396	2.256088088	profile #4

Vps51	2.803157826	2.800105039	6.052998334	profile #4
Ankrd1	412.8113226	348.4408162	1147.846341	profile #4
Syvn1	3.656598843	3.168240684	6.82914805	profile #4
Il33	1.135476175	1.095032159	12.99831368	profile #4
Frmd8	2.377071521	2.315870643	9.059046625	profile #4
Uhrf2	2.430075575	2.749322219	7.830269909	profile #4
Slc25a45	1.903781167	1.665460558	9.473363873	profile #4
Rad9a	2.714638593	2.263645962	5.877881277	profile #4
Gldc	0.112750913	0.117651723	0.53544588	profile #4
Coro1b	27.30178742	24.79219725	99.77838304	profile #4
Pold4	9.409504156	7.005508696	34.26084954	profile #4
Grk2	14.45773237	15.46220685	29.74341734	profile #4
Cnih2	0.449001662	0.357044922	0.736980637	profile #4
Rin1	0.497341667	0.442401905	1.455359896	profile #4
Aldh3b1	1.566143118	1.336754845	20.32207536	profile #4
Rce1	4.47041401	3.583356316	11.6077202	profile #4
Minpp1	5.602220084	5.160342036	12.56601231	profile #4
Apba1	1.226798993	1.312781831	3.003164076	profile #4
Papss2	4.800000909	4.796680263	10.62122911	profile #4
Cpt1a	9.036202394	10.91175629	18.31153549	profile #4
Gal	0.066052891	0.076527092	0.840930363	profile #4
Efemp2	2.884047214	2.921901495	33.15385882	profile #4
Ctsw	0.172720117	0.221133482	1.405946097	profile #4
Rnaseh2c	18.48863551	16.77684251	37.65626626	profile #4
Ltbp3	8.730753692	9.382101392	33.34037745	profile #4
Capn1	8.311530279	8.215795117	17.3616532	profile #4
Smc5	2.639513515	2.536532645	5.157214181	profile #4
Rps6ka4	9.65020245	8.979309711	24.42062962	profile #4
Bad	10.66193055	10.80603978	26.38063614	profile #4
Plcb3	7.56773367	8.000636433	16.31897332	profile #4
Fermt3	1.789122072	1.929188103	31.47621528	profile #4
Acsl5	3.711140897	3.46866499	13.63531605	profile #4
Zdhhc6	2.368342252	2.038998051	4.350101192	profile #4
Hhex	2.152319593	2.210625577	8.228195058	profile #4
Rbp4	0.142743525	0.136211124	0.813357755	profile #4
Dennd10	1.702772	1.754771114	4.878075075	profile #4
Hells	0.076787936	0.055169759	2.176307402	profile #4
Aldh18a1	1.912612317	1.594417769	8.633284241	profile #4
Tm9sf3	15.24024253	15.2802084	43.4678413	profile #4
Pik3ap1	0.84628234	0.757586988	23.75855069	profile #4
Slit1	0.046902133	0.029837161	0.207856919	profile #4
Xpnpep1	6.650159204	6.575343293	14.05239383	profile #4
Trim8	12.1723822	11.65656882	23.44919998	profile #4
Sfxn2	1.182347731	1.348384041	3.1240173	profile #4
Maoa	1.128621514	1.261293104	2.581572251	profile #4
Msr1	0.459621505	0.443763102	14.44922913	profile #4
Pcgf6	1.300152618	1.09078464	2.954807593	profile #4
Tasl	0.079357394	0.05555029	1.505647123	profile #4
Casp7	4.835340108	4.360262973	9.736388873	profile #4
Afap1l2	0.660625838	0.681691204	1.323535869	profile #4
Hdgfl3	0.889437514	1.083980925	1.902754721	profile #4
P4hb	56.12066963	56.70164137	242.3777721	profile #4
Arhgdia	79.10480203	78.61806482	287.9675956	profile #4
Alyref	5.280711005	5.513514551	13.48826224	profile #4
Sirt7	3.224838666	3.240649368	8.930626317	profile #4
Pycr1	0.262935829	0.359168137	4.320757345	profile #4
Cenpx	26.475412	23.91287806	50.39157243	profile #4
Lrrc45	2.765703826	2.856803919	6.740430246	profile #4

Cbr2	10.77393835	12.8929985	27.13638238	profile #4
Maged1	21.77535087	21.98679511	61.48523086	profile #4
Fasn	2.109802632	2.120360417	4.297437396	profile #4
Arhgap19	0.413990631	0.446820186	10.32572737	profile #4
Zdhhc16	3.39919786	3.086959767	7.538123974	profile #4
Mms19	1.648735684	1.446064501	3.067031133	profile #4
Slc16a3	0.731084128	0.891386652	15.40041546	profile #4
Ubtd1	14.45825048	13.88336561	36.66170015	profile #4
Ankrd2	0.378692084	0.284366982	1.534436375	profile #4
Hoga1	0.376788286	0.265321022	0.624754943	profile #4
Pi4k2a	6.787424336	7.368706867	14.68110963	profile #4
R3hcc1l	1.254251135	1.155869577	2.60700678	profile #4
Loxl4	0.223259713	0.197992677	1.958316565	profile #4
Hps1	0.308300415	0.34432617	3.622986941	profile #4
Entpd7	0.199424964	0.176294924	1.55619427	profile #4
Erlin1	1.427442444	1.397729031	5.852831041	profile #4
Scd2	6.009442199	5.1149205	25.82130529	profile #4
Sema4g	0.043676352	0.05350364	0.237010986	profile #4
Sfxn3	4.677675398	4.113062552	14.41542575	profile #4
Nfkb2	6.248208293	5.847982159	21.7079126	profile #4
Mfsd13a	0.789239771	0.777279456	2.608135153	profile #4
Hexa	25.54347085	23.28539992	159.1346959	profile #4
Adpgk	1.151214126	0.937658153	2.722886137	profile #4
Zfhx4	0.026470184	0.029815436	0.06250545	profile #4
Maged2	5.321422027	4.58163924	35.2922735	profile #4
Tro	0.126876612	0.104553462	1.874270248	profile #4
Acot9	9.310755093	9.249112827	21.05647384	profile #4
Prdx4	6.78870664	5.683337988	35.96562943	profile #4
Rps24	60.00491275	55.44534643	122.8261444	profile #4
Ptprj	1.014019294	1.232134153	7.307786877	profile #4
Banp	1.141703109	1.265235621	2.329973397	profile #4
Jph3	0.020769391	0.012225104	0.081816922	profile #4
Itgb8	0.114810197	0.131780567	0.667660479	profile #4
Atp10a	0.538481872	0.395260935	2.822885151	profile #4
Padi3	0.013427499	0.015124441	0.238027428	profile #4
Padi4	0.087542281	0.090154771	1.824027522	profile #4
Kdm5c	5.604926902	5.184064743	10.44352872	profile #4
Cd63	33.93748035	32.33947156	172.4209315	profile #4
Gdf11	0.854589882	0.793734946	2.311655342	profile #4
Ormdl2	1.858657162	1.348795987	6.786430321	profile #4
Dnajc14	6.389665405	6.530498602	14.02637777	profile #4
Mmp19	0.366818133	0.425038079	6.500438291	profile #4
Cdk2	2.080351185	1.76104603	4.878540478	profile #4
Rps26	122.87864	110.4132161	312.5147012	profile #4
Esyt1	4.54311549	4.740585707	18.72341041	profile #4
Rnf41	3.842337349	3.131388908	7.731699087	profile #4
Cnpy2	19.73745075	18.38163169	54.39314336	profile #4
Prim1	0.506121742	0.577554003	3.759239535	profile #4
Nab2	2.911475274	2.482589881	11.49262063	profile #4
Katnal2	0.011580807	0.012837712	0.145917085	profile #4
Rnf165	0.032485456	0.041155671	0.085461101	profile #4
Cd163l1	0.011051217	0.014148868	0.065744482	profile #4
Paox	0.858623488	0.871201066	3.651824993	profile #4
Zfp511	3.098710337	2.711809818	6.188450277	profile #4
Adam8	0.089814854	0.211464256	32.47112854	profile #4
Adgra1	0.007901641	0.008200233	0.063523677	profile #4
Bet1l	2.653077118	1.798836213	5.550122162	profile #4
Ric8a	3.229766722	2.804090552	6.852455948	profile #4

Ifitm3	134.0068529	122.2068924	513.964731	profile #4
Irf7	4.072748498	3.304446339	20.84760327	profile #4
Taldo1	42.16617576	40.43922904	92.63514673	profile #4
Pidd1	0.183993504	0.276768478	1.108975936	profile #4
Chid1	2.10536833	1.79142903	4.163333703	profile #4
Crcp	8.243141154	6.325416847	15.52960019	profile #4
Asl	1.013081607	0.958687567	4.027971145	profile #4
Gusb	4.661024914	4.457432649	45.11033764	profile #4
Sumf2	2.824555539	2.651945968	6.761630902	profile #4
Farp1	2.145451474	2.254696023	8.127899825	profile #4
Tmc6	4.552369779	3.746213768	7.532046599	profile #4
Tk1	0.605869475	0.561658526	7.225813004	profile #4
Cant1	7.094562825	6.563980536	14.16090785	profile #4
Cbx2	1.159679465	0.93375173	2.081599734	profile #4
Cbx8	1.335011246	1.093645598	2.583494984	profile #4
Klhl4	2.027533571	1.733133662	3.567436132	profile #4
Zfp202	0.625694997	0.558203865	1.153696682	profile #4
Map3k7cl	1.366479182	1.122876021	7.20311991	profile #4
Bach1	5.063664265	5.046369725	14.03835999	profile #4
Phf6	0.899570887	0.947423275	2.521128007	profile #4
Hprt	37.30774782	36.31997226	73.94297115	profile #4
Shisa5	12.57840184	14.19971012	57.94407001	profile #4
Pfkfb4	0.689661685	0.630954432	4.386391345	profile #4
Col7a1	0.095153052	0.131766561	0.766572341	profile #4
Rps6ka6	0.046907698	0.044051922	0.090928092	profile #4
Sec11a	7.305185008	6.058079692	15.99535188	profile #4
Sdc3	15.63467191	14.95568116	70.40113996	profile #4
Tyms	0.177645286	0.188590553	4.40057402	profile #4
Plk4	0.204875539	0.254353505	2.879229074	profile #4
Ly96	1.65824457	1.633972864	4.959490734	profile #4
Itih5	1.476419769	1.280268179	3.058675104	profile #4
St8sia2	0.061145544	0.041016398	0.117562222	profile #4
Hgs	7.040952038	6.807334204	14.24907065	profile #4
Ccr1	0.315468752	0.404852392	12.45346395	profile #4
Itgb1	94.27039832	99.46834341	245.2908222	profile #4
Nudt5	0.958282324	0.912755277	2.87647658	profile #4
Zfp282	1.380878659	1.216119326	3.237875009	profile #4
Pdia4	10.78494568	9.039575391	55.02551189	profile #4
Fam20c	2.423092999	2.658119286	11.52352542	profile #4
Nop16	8.304046225	7.375046119	15.76231818	profile #4
Arl10	3.128057101	2.363531949	7.22400931	profile #4
Faf2	2.327030915	2.210301863	5.888991677	profile #4
Tspan17	7.861928428	7.196706908	19.40918608	profile #4
Unc5a	0.699599099	0.78801311	1.437613735	profile #4
Hk3	0.306548562	0.355158869	14.28674584	profile #4
Casp12	2.082042173	1.64474045	5.98267063	profile #4
Casp1	1.759292502	1.383110898	12.17025815	profile #4
Sntg1	0.004311483	0.004048997	0.020893955	profile #4
Mybl1	0.204834165	0.193017204	0.775860224	profile #4
Sgk3	0.743453234	0.772896362	2.035291685	profile #4
Terf1	1.274530532	1.131379052	3.040235266	profile #4
Msc	0.885933447	0.770440862	3.897100244	profile #4
Tram1	10.78320759	10.56315898	39.56802845	profile #4
Ube2w	3.07317086	2.564107758	6.393744268	profile #4
Pikfyve	0.538572946	0.499180835	1.616625487	profile #4
Akr1cl	0.010996576	0.010493337	0.071126446	profile #4
Mettl21a	2.478433098	1.891048316	4.952754021	profile #4
Eef1b2	58.91596649	54.91153481	128.8086686	profile #4

Nrp2	6.015198526	4.785237414	12.34439145	profile #4
Rftn2	0.985849126	1.020882624	2.170781818	profile #4
Slc39a10	2.665445511	2.360072932	5.294870339	profile #4
Slc40a1	1.572880801	1.683051842	5.379177919	profile #4
Wdr75	2.389796075	2.181114097	5.218702777	profile #4
Rpe	2.929027895	2.579064501	8.463392837	profile #4
Nop58	1.689195683	1.516817747	4.118418791	profile #4
Cdk15	0.036924282	0.052851763	0.437623458	profile #4
Casp8	3.263759403	2.829253243	18.79696756	profile #4
Nif3l1	1.175287099	1.188678161	2.35907486	profile #4
Orc2	2.196027899	2.08157319	4.644497071	profile #4
Sgo2a	0.349014163	0.39079782	0.897216411	profile #4
Col5a2	2.914804625	2.611029197	86.71131221	profile #4
Col3a1	12.24070808	10.86304261	721.6818473	profile #4
Poglut2	3.671795369	2.819372548	5.770807199	profile #4
Tex30	1.129478524	1.27306752	2.958349716	profile #4
Ecrg4	0.205253162	0.195860119	6.258631553	profile #4
Il18rap	0.037818338	0.032706203	0.392709355	profile #4
Il18r1	0.019217184	0.009168872	0.397437119	profile #4
Il1r1	2.993401178	2.621854934	14.50637453	profile #4
Il1r2	0.022305156	0.05711456	2.771248051	profile #4
Map4k4	16.24116404	15.60842712	32.06794019	profile #4
Npas2	0.284671279	0.350307944	1.198781842	profile #4
Chst10	0.027516517	0.022149677	0.121538677	profile #4
Mitd1	1.219774341	1.053971486	4.678157628	profile #4
Stk17b	1.910299293	2.810979895	8.528921184	profile #4
Stat1	2.102454772	2.186617328	5.555838467	profile #4
Mgat4a	1.387413262	1.09683251	2.911803105	profile #4
Zap70	0.046408344	0.059046065	0.18281652	profile #4
Ptpn18	0.256661301	0.276968664	1.99607157	profile #4
Imp4	2.45238252	2.235949469	4.657884791	profile #4
Prim2	0.29385108	0.304955327	2.387349361	profile #4
Smap1	1.41662383	1.260133873	3.468076268	profile #4
Agfg1	3.969300904	3.847384971	8.595603659	profile #4
Rnf25	4.95634671	4.414620629	8.945072919	profile #4
Cnot9	3.822773082	3.733586356	10.13566213	profile #4
Slc11a1	1.33282433	1.203997879	23.31370031	profile #4
Igfbp5	15.41278322	14.07996568	29.00688791	profile #4
Atic	3.333598837	3.180427387	6.8330565	profile #4
Fn1	3.980516239	4.280644595	157.3007057	profile #4
Bard1	0.091416548	0.067399016	0.853811408	profile #4
Zfand2b	5.772513516	5.408646238	11.08687968	profile #4
Glb1l	0.40758655	0.273692184	1.332631003	profile #4
Ptprn	3.175090868	2.269769429	8.217464193	profile #4
Stk11ip	2.082337554	2.096977484	4.259603783	profile #4
Sp100	4.543627674	3.764319508	8.577897366	profile #4
Itm2c	36.62261059	34.34380847	114.8517311	profile #4
Htr2b	0.059913051	0.097858182	2.31677571	profile #4
Ptma	152.688487	137.708985	454.2948017	profile #4
Serpine2	3.126987068	3.126652771	12.54653286	profile #4
Ngef	0.04741059	0.067861393	0.843299181	profile #4
Capn10	1.471705834	1.595520875	2.989172099	profile #4
Gpr35	0.058313278	0.078820217	4.003400967	profile #4
Pask	0.031145469	0.048748849	0.54492815	profile #4
Septin2	0.1386783	0.110706747	0.262639236	profile #4
Bok	3.219797427	3.072449171	23.8811366	profile #4
Dtymk	7.722894008	7.28553488	19.55079567	profile #4
Inpp5d	1.198035083	1.173589093	11.74633419	profile #4

Mlph	0.055610913	0.071198624	0.144651538	profile #4
Serpinb8	0.415065127	0.326176935	1.760041724	profile #4
Cln8	2.423826733	2.404011719	7.838237965	profile #4
Tnfrsf11a	0.377180219	0.442771497	3.587174752	profile #4
Ccdc93	1.487526114	1.480607003	3.026975733	profile #4
Actr3	19.36936549	19.46058679	86.29229259	profile #4
Gpr39	0.030702878	0.017291525	1.063863572	profile #4
Mcm6	1.693309607	1.883653003	14.32138492	profile #4
Nifk	7.867202237	6.959954074	14.77653679	profile #4
Ptprc	0.336421409	0.434398751	7.226003004	profile #4
Nr5a2	0.223648982	0.221680786	0.449495434	profile #4
Cd55	1.955591764	2.002188441	4.26423709	profile #4
Tnni1	0.08999781	0.060541433	0.329205156	profile #4
Csrp1	30.14650569	29.1100296	137.888644	profile #4
Srgap2	2.64588648	2.311991108	5.516323542	profile #4
Arl8a	9.120016689	9.409070933	28.50454176	profile #4
Ube2t	0.612533836	0.560580072	1.347862303	profile #4
Rassf5	0.382134452	0.54528186	4.814934207	profile #4
Rab29	6.05904054	5.445591749	20.85926293	profile #4
Nucks1	7.571855451	7.51201132	16.49406479	profile #4
Nfasc	0.006544304	0.006244815	0.074117484	profile #4
Lrrn2	1.041759346	0.788133261	2.570554401	profile #4
Ppfia4	0.683141363	0.555485152	3.863255417	profile #4
Stx6	2.338882142	1.931156179	5.288013672	profile #4
Lamc1	25.9567461	26.39736008	54.47249	profile #4
Lamc2	0.465869492	0.642027705	1.697073477	profile #4
Ncf2	0.552182833	0.494871588	7.411103265	profile #4
Niban1	3.342775801	3.503853329	9.764978466	profile #4
Rnf2	2.859842487	3.296163971	7.964977769	profile #4
Kif26b	0.18880987	0.243432431	3.681691183	profile #4
Acbd3	3.565375291	4.067678143	9.687909401	profile #4
Sdccag8	2.040153795	1.834277862	3.893692692	profile #4
Capn2	11.88156699	13.64543792	28.2617149	profile #4
Opn3	0.600665131	0.712065994	1.29665672	profile #4
Ifi211	2.186813252	2.301109516	20.1018363	profile #4
Cfap45	0.119106488	0.082071982	0.479762991	profile #4
Tagln2	32.03076645	31.58797256	189.2484528	profile #4
Slamf9	3.259481282	2.53139732	14.36562006	profile #4
Copa	14.20893155	14.72527568	29.90625132	profile #4
Vangl2	0.50534101	0.39754675	1.219426905	profile #4
Uck2	6.507031813	6.48183353	25.28155736	profile #4
Tada1	1.087060233	0.945656497	2.06284646	profile #4
Mpzl1	4.819270771	4.111973261	12.42918883	profile #4
Dpt	61.56064983	62.59702418	233.9798845	profile #4
Nme7	0.44904879	0.381511183	1.637324043	profile #4
Selp	0.042628288	0.070057829	1.424608066	profile #4
Sell	0.175038485	0.248884592	1.020164355	profile #4
Scyl3	1.284353442	1.305365958	2.705495367	profile #4
Sec16b	0.538988733	0.473364625	3.361720359	profile #4
Soat1	0.439013576	0.428507948	9.046216289	profile #4
Cr2	0.015738993	0.010889166	0.039333798	profile #4
Nek2	0.018847758	0.01955999	2.8362739	profile #4
Pacc1	2.135808178	1.795217921	6.895057489	profile #4
Batf3	0.346435241	0.387182464	2.682987932	profile #4
Traf5	1.054226271	0.890593045	2.678811271	profile #4
Nmt2	1.655331057	1.554167664	4.839116487	profile #4
Suv39h2	0.314861143	0.280421948	1.033611166	profile #4
Dclre1c	0.310644065	0.346087521	1.606758315	profile #4

Fam107b	1.49327147	1.870835408	7.82921484	profile #4
Fcgr2b	1.558807368	1.693428915	26.5467586	profile #4
Frmd4a	1.332044674	1.263717937	2.66661057	profile #4
Dusp12	1.200173567	0.821177287	2.260002307	profile #4
Sephs1	2.682873346	2.556534444	7.58550226	profile #4
Uhmk1	4.243757606	3.685845555	10.17877973	profile #4
Ucma	0.048142802	0.061637206	0.190936653	profile #4
Mcm10	0.036103423	0.022967476	1.463704673	profile #4
Ddr2	3.665962117	3.17958548	7.574871712	profile #4
Enkur	0.271151138	0.279242997	0.767126737	profile #4
Nuf2	0.029197028	0.020895658	3.529551481	profile #4
Cenpl	0.113781252	0.143513494	1.311478324	profile #4
Mrc1	3.476843377	4.729472813	28.4220396	profile #4
Serpinc1	0.011006239	0.006375765	0.020017466	profile #4
Tnn	0.036580915	0.03796326	0.374958414	profile #4
Rsu1	2.796331084	2.63602361	9.494872883	profile #4
Vim	33.1008481	35.93471578	335.7594672	profile #4
4930426L09Rik	0.087508369	0.090815196	0.674193461	profile #4
Pip4k2a	3.584371442	3.823669464	19.15066624	profile #4
Plxdc2	3.464515009	3.34701889	8.208348608	profile #4
Nek6	0.332640776	0.345821888	4.207657984	profile #4
Kif5c	0.17497674	0.191705427	0.395217422	profile #4
Mindy3	1.703648258	1.739454847	3.640446644	profile #4
Pfkfb3	2.612413248	1.967336349	5.547390335	profile #4
Mastl	0.088130599	0.081219581	1.428143965	profile #4
Acbd5	6.003641508	6.742047903	12.28121651	profile #4
Pkn3	3.912690743	3.705622818	7.552406101	profile #4
Apbb1ip	0.362086068	0.327644339	4.632068308	profile #4
Niban2	11.70708323	11.00380046	55.08505933	profile #4
Ddx31	0.770720759	0.826118001	2.270083878	profile #4
Dpm2	4.7200417	3.994026359	11.28310711	profile #4
Ralgds	2.283141402	1.884363166	6.721100393	profile #4
Gbgt1	0.311564168	0.285273769	1.791301177	profile #4
Cytip	0.076709111	0.183372003	2.114855592	profile #4
Olfm1	0.950963953	1.168490555	7.864317685	profile #4
Acvr1	2.578288295	2.288754753	5.767396196	profile #4
Col5a1	5.882340455	5.519325003	103.5506363	profile #4
Upp2	0.242808699	0.301290841	0.582115738	profile #4
Fubp3	2.50496152	2.59171125	5.919372651	profile #4
Tor1a	5.057664254	4.737655019	17.04801116	profile #4
Dolpp1	1.949265153	1.938149809	4.629929002	profile #4
Hspa5	88.5682296	93.20431659	182.8679344	profile #4
Psmd5	2.573499914	2.502044292	5.201294364	profile #4
Zeb2	1.180157708	1.336088895	7.046940338	profile #4
Phf19	0.358225627	0.310382695	1.604092608	profile #4
Traf1	0.133319136	0.105032762	0.868051876	profile #4
Morn5	0.065867869	0.073016732	0.295497201	profile #4
Ifih1	1.670373664	1.421407562	3.797465256	profile #4
Card9	0.779383787	0.558581443	6.091237291	profile #4
Fcna	1.362854748	1.745386862	5.108927855	profile #4
Traf2	3.202991485	2.446106524	7.072816169	profile #4
Nmi	5.232047116	5.731868204	11.11651134	profile #4
Uap1l1	1.735364118	1.418730711	16.23200749	profile #4
Dpp7	0.929297991	0.979693555	8.244664133	profile #4
Anapc2	14.47844808	13.87092981	27.90507122	profile #4
Ssna1	25.46377804	20.66759271	45.93388202	profile #4
Rbms1	12.67688606	13.47881136	27.50925996	profile #4
Arrdc1	2.268129213	1.736646689	7.701524564	profile #4

Psd4	0.14804749	0.106330194	2.015689053	profile #4
Ly75	0.141758066	0.149928821	0.365426737	profile #4
Il1rn	0.016993553	0.028256882	2.70940579	profile #4
Galnt3	0.010581753	0.010097498	0.270950746	profile #4
Nup35	1.127550867	1.220285349	3.409858612	profile #4
Frzb	1.485659799	1.337137093	13.4057165	profile #4
Dnajc10	7.603316093	7.493469596	23.41965847	profile #4
Itga4	0.107665551	0.193945144	1.917467765	profile #4
Dync1i2	6.322056931	6.571627394	13.21775218	profile #4
Cybrd1	0.026007448	0.016544842	0.093913612	profile #4
Hat1	6.827937253	6.834511063	14.57810776	profile #4
Xirp2	23.54427432	21.90417846	50.97875445	profile #4
Cers6	0.318125056	0.263994756	3.399532984	profile #4
Ssrp1	12.1969151	10.77498186	30.81101676	profile #4
Slc43a1	0.437258179	0.432701467	1.188543921	profile #4
Ube2l6	6.168186578	5.537272897	22.63126861	profile #4
Clp1	2.113293315	1.853564722	4.776583338	profile #4
Itgav	1.865685925	1.819641662	7.942105039	profile #4
Hoxd8	0.421689031	0.305817303	0.953430765	profile #4
Chrna1	0.0097032	0.012423003	0.051963352	profile #4
Sp3	7.093060192	7.676599727	17.08525942	profile #4
Itga6	8.518415588	7.17919027	16.72275211	profile #4
Kif18a	0.047833257	0.058397471	0.443680451	profile #4
Arl14ep	1.568083162	1.374232419	3.867859808	profile #4
Katnbl1	1.253814643	1.188384052	3.526112914	profile #4
Lpcat4	1.070363025	1.200391972	3.832848335	profile #4
Ccdc34	0.733315153	0.675722091	3.739440653	profile #4
Depdc7	0.426318469	0.223857596	1.522308048	profile #4
Tcp11l1	0.615481935	0.635812015	1.716708358	profile #4
Cstf3	1.78803955	1.649415562	4.380672328	profile #4
Caprin1	11.22805496	10.85053673	23.73006976	profile #4
Pamr1	0.366031809	0.225303051	1.619203453	profile #4
Api5	10.64189097	11.19716804	23.61852683	profile #4
Hsd17b12	8.018559701	7.832406436	25.83057429	profile #4
Gatm	0.877594427	0.833865192	9.989966092	profile #4
Myef2	1.18296986	1.062703428	4.914528563	profile #4
Fbn1	4.426955977	3.769363142	51.44561077	profile #4
Galk2	1.321206763	1.327326173	5.309280921	profile #4
Cd82	4.03782093	3.481384244	12.60959788	profile #4
Tspan18	1.337916438	1.406338613	3.557403623	profile #4
Chst1	1.579354203	1.301921675	5.714789856	profile #4
Duoxa1	0.032103271	0.041101803	0.091691732	profile #4
Creb3l1	1.684017826	1.713719608	15.42777924	profile #4
Mdk	1.643032958	1.440373977	25.74437352	profile #4
Wdr76	0.398245199	0.272866018	1.120578767	profile #4
Arhgap1	4.201070403	4.282509799	13.28549392	profile #4
Pdia3	41.05010928	38.88229668	192.1018214	profile #4
Map1a	0.724112694	0.73332437	2.382895984	profile #4
Tubgcp4	0.821489412	0.726929894	1.757560915	profile #4
Slx4ip	0.353150484	0.231864549	1.100480618	profile #4
Tyro3	0.418189243	0.395599878	2.968359494	profile #4
Ptpra	10.9362085	11.53492771	24.91660996	profile #4
Nusap1	0.16221119	0.14145453	5.199453775	profile #4
Siglec1	0.572809784	0.465312149	6.955712605	profile #4
Rad51	0.070731927	0.086353427	1.726212075	profile #4
Knl1	0.019928933	0.015511516	1.57419392	profile #4
Cdc25b	0.931154533	0.847709982	5.495049404	profile #4
Knstrn	0.098706954	0.121288748	6.309757315	profile #4

Rassf2	0.649391925	0.57818175	7.040456737	profile #4
Slc23a2	2.619552837	2.542420392	5.153873089	profile #4
Pcna	15.40962828	15.40305182	63.834146	profile #4
Spred1	4.284599244	4.192565641	11.60385465	profile #4
Bmp2	0.562654609	0.646035374	3.47672411	profile #4
Hdc	0.224576965	0.247320614	0.746323531	profile #4
Gabpb1	1.415047389	1.251127141	3.145833984	profile #4
Usp8	9.347993367	9.175972495	19.66570347	profile #4
Bub1	0.01063036	0.010143881	3.537415048	profile #4
Bcl2l11	1.63716814	1.383052765	4.299059194	profile #4
Fbln7	0.065920441	0.050638745	0.797403042	profile #4
Slc20a1	3.081277367	2.493267725	7.365569591	profile #4
Nop56	6.499501415	5.862135845	12.9996714	profile #4
Cpxm1	1.991798859	2.142944863	17.32146623	profile #4
Lpin3	1.474676319	1.721702703	4.195442833	profile #4
Bfsp1	0.135561555	0.129357823	0.617947704	profile #4
Rrbp1	13.97940721	13.56290723	42.53686406	profile #4
Snx5	12.55400269	12.27496762	53.2589384	profile #4
Sec23b	2.386939242	2.464118948	7.13172023	profile #4
Xrn2	6.840033226	6.770472356	14.95363518	profile #4
Cd93	17.28133562	12.52827157	43.67758739	profile #4
Gins1	1.796653801	1.660612163	6.254029356	profile #4
Sdcbp2	0.943034556	0.662970403	1.387463913	profile #4
Fam110a	1.53427069	1.520911065	4.853570149	profile #4
Angpt4	0.100705907	0.113489825	0.56611791	profile #4
Tbc1d20	11.60721147	12.54789052	23.42029627	profile #4
Tpx2	0.100555182	0.066881266	11.89017489	profile #4
Mylk2	0.079684274	0.035333071	0.231879719	profile #4
Kif3b	2.316282843	2.601079143	7.653120556	profile #4
Dnmt3b	0.3584027	0.259497396	0.611550729	profile #4
Mapre1	9.898445599	9.898535951	22.79404404	profile #4
E2f1	0.238577849	0.294069624	7.182745547	profile #4
Aurka	0.204395987	0.247177938	7.347909517	profile #4
Rtf2	14.06804049	13.84850937	27.99301761	profile #4
Tpd52	2.387399426	2.330138968	17.85496154	profile #4
Pag1	0.471278415	0.528947595	1.436935306	profile #4
Zbp1	0.288540928	0.251141143	5.541169252	profile #4
Zdbf2	0.011145349	0.012354992	0.023333518	profile #4
Impa1	3.174523574	3.216924783	8.692411323	profile #4
Fabp5	10.56228053	8.716900929	93.13624434	profile #4
Ptpn1	4.980910877	5.380117822	28.27877773	profile #4
Lrrcc1	0.791383937	0.809472186	2.45561139	profile #4
E2f5	2.389552243	2.018654502	4.191284544	profile #4
Car13	0.222139558	0.1921116	6.827874781	profile #4
Dok5	0.216642609	0.206412685	1.237527215	profile #4
Arfgap1	6.359550214	6.33502308	13.86531194	profile #4
Ahcy	7.8466596	8.177873993	18.13539882	profile #4
Itch	2.489759868	2.806019665	5.920199846	profile #4
Ggt7	0.49546967	0.517495706	1.703748405	profile #4
Procr	0.75435575	0.717623376	4.986351589	profile #4
Mmp24	0.009087815	0.009431231	0.091324612	profile #4
Eif6	15.34825716	15.04349306	39.18859081	profile #4
Hps3	1.122045134	1.15522871	3.054797673	profile #4
Epb41l1	1.33698541	1.329578447	3.523055769	profile #4
Dsn1	0.304531212	0.381880515	2.378322652	profile #4
Sla2	0.013732893	0.017582217	0.081697968	profile #4
Samhd1	3.720938746	3.702259278	14.0033746	profile #4
Rbl1	0.682726563	0.626338088	3.304065931	profile #4

Rpn2	14.76300742	14.48619905	45.10893784	profile #4
Src	1.527845232	1.404515557	6.517709395	profile #4
Ctnnbl1	5.612327123	5.473582575	11.19381126	profile #4
Fam83d	0.011783928	0.026125756	2.400079948	profile #4
Ccn5	7.137557319	4.935440863	49.829306	profile #4
Skil	2.282884587	2.467497447	7.504567244	profile #4
Slc2a10	0.635278659	0.744770667	2.863134014	profile #4
Zmat3	0.732620064	0.779615644	2.59103315	profile #4
Gnb4	1.462347919	1.363730721	2.894510251	profile #4
Actl6a	4.942898617	4.637137002	9.583299081	profile #4
Ect2	0.043659171	0.070815267	2.83513824	profile #4
Anxa5	52.44937469	48.04816163	262.5380054	profile #4
1810062G17Rik	0.258121068	0.239241668	0.516400079	profile #4
Ccna2	0.046073631	0.051074158	10.73968328	profile #4
Slc7a11	0.006756164	0.003744717	0.300064864	profile #4
Rab33b	2.52606833	2.44869392	4.985400265	profile #4
Ufm1	6.020569941	5.587736506	12.8300499	profile #4
Postn	2.095624877	2.254128287	249.5028554	profile #4
Supt20	2.851236989	2.604201296	5.461501319	profile #4
Exosc8	2.456832505	2.406367261	6.248755582	profile #4
Mfsd1	7.714098002	7.557879848	47.08068281	profile #4
Ift80	0.45031617	0.469826362	1.076315506	profile #4
Dclk1	0.180850248	0.184050283	3.704927526	profile #4
Tm4sf1	29.29615407	22.02789066	58.40397176	profile #4
Serp1	5.259840407	5.167038309	40.80599099	profile #4
4930579G24Rik	0.644568924	0.423422917	2.869136701	profile #4
Slc33a1	3.091726989	2.33632963	5.316251168	profile #4
Ssr3	30.65300016	33.63298978	77.71489968	profile #4
Shox2	0.198856907	0.146732415	0.611735432	profile #4
Pdcd10	4.912591072	5.17249196	14.11359296	profile #4
Ptpn22	0.047821715	0.080424044	1.163945339	profile #4
Olfml3	2.584304216	2.750127038	22.11416207	profile #4
Nras	5.822467569	5.46799128	18.28078328	profile #4
Ptgfrn	5.825382493	6.607833604	17.1412578	profile #4
Gdap2	1.599047629	1.685599028	3.479996743	profile #4
Notch2	1.526653726	1.61713344	6.172332456	profile #4
Sec22b	9.54777347	8.597796175	27.41125894	profile #4
Prpf38b	5.603797204	4.703983967	10.31090899	profile #4
Stxbp3	3.170538775	3.217369234	6.914161884	profile #4
Gpsm2	0.261293857	0.237271833	3.848079637	profile #4
Sypl2	0.106954674	0.070634041	0.234502073	profile #4
Ampd2	1.613041904	1.293542859	6.478176543	profile #4
Slc16a4	0.384000591	0.252841252	0.871002303	profile #4
Dennd2d	0.068162581	0.075908484	0.342487239	profile #4
S100a11	57.42549667	50.29752588	577.9401437	profile #4
Slc27a3	0.662642848	0.615076452	3.958535258	profile #4
Rab13	1.931878456	1.709049852	8.145855715	profile #4
Creb3l4	0.126736858	0.162261137	0.65156287	profile #4
Nup210l	0.066962261	0.063800284	0.243444233	profile #4
Tpm3	10.93031273	10.40965411	54.14437478	profile #4
Hax1	12.9109258	12.07811606	24.90073821	profile #4
Il6ra	0.702016425	1.088573861	5.288052691	profile #4
Adar	1.969707304	1.740367031	4.772716748	profile #4
Gask1b	3.129683019	2.741963013	13.23585513	profile #4
Slc35a3	1.600607392	1.280872972	3.123056234	profile #4
Vcam1	1.894171978	2.345145968	8.998255241	profile #4
Col11a1	0.003339472	0.00855105	3.016436542	profile #4
Ndst3	0.01405687	0.011032049	0.072620875	profile #4

Mcub	1.605716717	1.107549702	16.10401454	profile #4
Tlr2	0.98281806	1.141619271	11.1006547	profile #4
Sfrp2	0.945899616	1.0256957	38.45355998	profile #4
Casp6	1.737693054	1.615181372	4.367210789	profile #4
Snx7	1.091795227	1.085593815	5.969369318	profile #4
Gar1	7.326090327	5.976435527	13.61616875	profile #4
Ctso	3.681220585	3.457105054	8.005827771	profile #4
Ints12	0.899909855	0.820232012	2.570276789	profile #4
Gstcd	0.362466006	0.425227876	1.746996158	profile #4
Pdgfc	0.240277288	0.3037583	2.379748299	profile #4
Alpk1	0.656839416	0.4706326	1.257862224	profile #4
Dkk2	0.068920222	0.028142481	1.165856647	profile #4
Papss1	3.974337338	4.012930716	14.12407822	profile #4
Fubp1	3.368323566	2.615389913	5.887462539	profile #4
Ifi44	0.583170835	0.578415464	2.244448051	profile #4
Efna3	0.057047561	0.021419041	0.187273017	profile #4
Efna4	0.088235757	0.032604094	1.63615636	profile #4
Adam15	9.22678931	9.753276148	21.81568826	profile #4
Zbtb7b	7.494799808	7.496993946	18.61282101	profile #4
Cks1b	2.004816703	2.274336255	25.07086431	profile #4
Thbs3	0.961817919	1.026897146	6.763068889	profile #4
Gba	2.94050229	2.934074897	18.69925688	profile #4
Arhgef2	5.783222598	5.7355815	11.816935	profile #4
Lmna	18.56774668	19.88734144	49.23632816	profile #4
Sema4a	0.842347641	0.816402234	3.473897151	profile #4
Pmf1	2.517571733	2.630638089	5.999844917	profile #4
Iqgap3	0.016524827	0.019145218	1.491446266	profile #4
Cd1d1	0.960458265	0.892565625	2.101640483	profile #4
Rps3a1	445.6016733	451.382861	976.6881739	profile #4
Sh3d19	1.958033876	1.722651716	5.407558885	profile #4
Rnf115	7.071478707	7.816185721	15.67983939	profile #4
Ecm1	11.56382082	11.19490913	74.73605968	profile #4
Ctsk	3.214207501	3.792813583	47.33208502	profile #4
Bcar3	1.025225193	1.182185676	2.464996956	profile #4
Gclm	3.247171151	3.115937683	6.855084141	profile #4
Rwdd3	0.218462815	0.144101728	0.332979929	profile #4
Snx27	4.4666565	4.913826169	9.099992083	profile #4
Rap1gds1	3.753466391	4.000588156	8.315640441	profile #4
Tspan5	0.572833843	0.663483111	1.574328034	profile #4
Dapp1	1.713061912	1.466558966	5.259276956	profile #4
Nfkb1	3.653339506	3.628965984	7.721480782	profile #4
Bdh2	0.050068514	0.073600866	0.472956196	profile #4
Wls	4.990621275	5.236778882	13.50047604	profile #4
Rpf1	3.795096869	3.093624817	6.497590947	profile #4
Ctbs	0.773530413	0.714989686	2.093831159	profile #4
Bcl10	3.884092289	3.689584747	9.801624042	profile #4
Ddah1	1.127191824	1.162796796	13.4123531	profile #4
Col24a1	0.018513184	0.017386087	0.948389495	profile #4
Trp53inp1	1.991528306	2.230491334	4.431499648	profile #4
Ccne2	0.542966555	0.514472265	2.569300065	profile #4
Mmp16	0.069424212	0.06015224	0.540425411	profile #4
Cpne3	3.160930355	3.54537968	8.23945076	profile #4
Tmem68	2.152460621	1.912481581	5.216729099	profile #4
Tgs1	2.098279652	2.194256686	5.103771206	profile #4
Rps20	36.21088356	36.27653792	96.37321071	profile #4
Atp6v0d2	0.017041454	0.048784746	3.495701426	profile #4
Faxc	0.066163248	0.026064238	0.283458159	profile #4
Sdcbp	12.75195839	12.71129052	80.48234361	profile #4

Ccnc	1.802113991	1.773471501	4.322070855	profile #4
Lmo4	11.53774298	12.78700252	24.74096194	profile #4
Gbp3	4.321254992	4.422398717	11.39207289	profile #4
Gbp2	8.037660237	6.817197324	13.97062849	profile #4
Gtf2b	2.42050552	2.502348829	6.01936042	profile #4
Ube2j1	8.619883584	9.8342309	18.516983	profile #4
Casp8ap2	1.191932453	1.04634496	2.477441496	profile #4
Epha7	0.396130898	0.446526758	1.135613323	profile #4
Smc2	0.447806346	0.507274878	7.314129222	profile #4
Anp32b	37.95808684	37.93785395	91.96139737	profile #4
Nans	3.09170136	3.068701328	16.20901314	profile #4
Col15a1	13.00546622	10.70178775	80.00431682	profile #4
Erp44	10.08836105	9.46911942	19.63050777	profile #4
Invs	0.295579995	0.271697622	0.900714555	profile #4
Tex10	1.709322158	1.802979696	3.838395971	profile #4
Fmn2	0.011629293	0.0110971	0.034358482	profile #4
Zfp618	0.039593317	0.0566721	0.383527056	profile #4
Tnc	0.076661775	0.060371271	47.43914409	profile #4
Txn1	67.74493848	67.03336421	217.328604	profile #4
Svep1	0.837470496	0.79447249	2.451883081	profile #4
Pappa	0.046914505	0.088942706	0.523586434	profile #4
Ptgr1	1.269964646	1.211884486	15.60932512	profile #4
Ugcg	2.941609492	3.304857668	9.777620531	profile #4
Ptbp3	4.90416225	4.662782132	18.90322821	profile #4
Snx30	0.995496299	1.209275323	4.128424658	profile #4
Zfp37	0.414358225	0.472089092	1.135993646	profile #4
Bspry	0.059036464	0.056334766	0.127283677	profile #4
Pole3	11.63921257	11.60452003	23.43910921	profile #4
Zdhhc21	0.788536382	0.727431726	1.809656182	profile #4
Dnaja1	8.684059333	9.181193586	19.83031355	profile #4
B4galt1	15.77436478	16.09910241	43.3032514	profile #4
Chmp5	23.6185607	21.77793033	44.23137118	profile #4
Dcaf12	8.377483536	8.828158717	18.32786621	profile #4
Kif24	0.052815938	0.019516077	0.240145895	profile #4
Fancg	0.706374317	0.618189091	2.368369188	profile #4
Cd72	0.64593628	0.761746737	13.38695607	profile #4
Car9	0.163297425	0.153355753	1.31865626	profile #4
Tpm2	7.937972611	8.320116311	37.12001507	profile #4
Tln1	15.36680434	15.70453036	34.98778543	profile #4
Creb3	12.29274379	10.11061807	31.11406137	profile #4
Clta	30.59540561	32.6706066	129.1572931	profile #4
Glipr2	1.100644058	1.123576438	27.09393366	profile #4
Psip1	3.260504232	3.71095574	8.520885439	profile #4
Bnc2	0.034521403	0.033329637	0.434966441	profile #4
Plin2	14.02051607	14.56247202	93.23710778	profile #4
Rps6	183.4457252	184.8307815	405.0220277	profile #4
Hacd4	1.026789908	0.919412529	6.722748546	profile #4
Mier1	2.157358266	1.963859005	4.360322978	profile #4
Dnajc6	0.023498691	0.017645607	0.064270266	profile #4
Cachd1	0.445931247	0.497807465	1.107496022	profile #4
Artn	0.078219806	0.090623351	0.248957347	profile #4
Dph2	1.831518054	1.564486541	3.47508261	profile #4
B4galt2	1.944378646	2.28519542	8.286749418	profile #4
Atg4c	1.080657626	1.12015642	3.412529719	profile #4
Cdkn2c	4.815024605	4.787416384	18.88795493	profile #4
Eps15	5.407969676	6.162020454	13.99057953	profile #4
Dock7	0.721468183	0.759473002	2.70490474	profile #4
Usp1	4.560604441	4.641563937	12.22283276	profile #4

Txndc12	7.95251564	7.013454975	15.30179728	profile #4
Ift74	1.150951733	1.353976329	3.429758368	profile #4
Caap1	0.865149344	0.77485012	1.980181392	profile #4
Laptm5	4.010184714	4.211453984	74.49280581	profile #4
Cc2d1b	3.443938114	3.114268458	7.623939275	profile #4
Pdpn	2.174183442	1.836308525	30.1038541	profile #4
Orc1	0.06651996	0.029970651	0.589595102	profile #4
Gpx7	7.863712005	6.59257821	89.66453366	profile #4
Tnfrsf1b	2.552602475	2.695333799	30.44859505	profile #4
Tnfrsf8	0.012303624	0.015071971	0.317178956	profile #4
Magoh	25.81129433	22.08322842	45.43027697	profile #4
Lrp8	0.044525452	0.025911779	0.947763414	profile #4
Lrrc42	3.024241239	2.291947971	8.305253453	profile #4
Tceanc2	1.54423743	1.430666543	3.377149349	profile #4
Col9a2	0.082503852	0.101067425	1.45883006	profile #4
Exo5	1.01420412	0.726967384	5.311876029	profile #4
Ctps	11.62697883	9.230203435	19.83570273	profile #4
Hivep3	0.339956806	0.334546394	0.954698005	profile #4
P3h1	5.162789458	5.385873182	24.8533498	profile #4
Svbp	19.90454583	19.39031254	47.74679649	profile #4
Ermap	0.026213661	0.009842165	0.087628281	profile #4
Slc2a1	4.712948636	5.038956856	12.23491659	profile #4
Mycl	0.034940311	0.032813118	0.328327584	profile #4
Cap1	15.81564074	14.39875746	91.19059657	profile #4
Ppt1	8.85806306	8.528171467	22.82834634	profile #4
Ephb2	0.030379452	0.035030502	0.755606818	profile #4
Eloa	6.612921395	7.302778229	13.65935185	profile #4
Gale	0.390587241	0.482798771	5.251176727	profile #4
Fuca1	7.321466983	7.913830942	26.89275646	profile #4
Pnrc2	23.5501338	20.31270408	44.04657512	profile #4
Ptch2	0.267661264	0.221793389	0.452060265	profile #4
Mutyh	0.708799204	0.524180766	1.309871841	profile #4
Toe1	1.522621559	1.715047592	3.794695737	profile #4
Ccdc163	1.216432337	1.103156722	2.342949406	profile #4
Prdx1	63.53293596	58.2886923	147.2804274	profile #4
Akr1a1	72.9729283	71.31923909	231.5500226	profile #4
Nasp	2.123993464	2.210511227	6.529939593	profile #4
Tspan1	0.01827318	0.0189637	0.039143197	profile #4
Rad54l	0.048823938	0.051582937	0.937928411	profile #4
Mknk1	4.784447711	4.635413598	11.32355402	profile #4
Mob3c	1.960288433	1.633045463	5.290581889	profile #4
Stil	0.024346674	0.02286443	1.093344795	profile #4
Cmpk1	8.013890224	8.226325441	24.79999142	profile #4
Slc66a1	1.64616616	1.304831431	3.563942967	profile #4
Pla2g2e	0.050473924	0.059419073	0.93276588	profile #4
Cda	0.545271403	0.526447245	2.466286121	profile #4
Ddost	32.20272043	31.43299788	106.709773	profile #4
Kif17	0.009288136	0.011891595	0.09333766	profile #4
Ptpn12	8.162352077	8.555855432	18.22510691	profile #4
Azin2	0.596051835	0.518230087	4.176827792	profile #4
Rnf19b	10.40246598	11.12747539	36.37692289	profile #4
Phc2	5.259545614	5.214242348	12.91196023	profile #4
Hdac1	7.016906958	7.148985491	24.09332355	profile #4
Zbtb8a	0.997082896	0.853550183	2.358559413	profile #4
Sfpq	15.2135089	16.53114276	31.59214593	profile #4
Tmem50a	50.17731281	49.09622068	109.7806684	profile #4
Stmn1	4.804962231	4.021399556	42.23328045	profile #4
Zfp593	0.090095131	0.063457553	0.218305807	profile #4

Sh3bgrl3	21.25213557	21.3038003	172.3715558	profile #4
Tekt2	0.099009807	0.057355042	0.533014741	profile #4
Gpn2	6.410250349	5.900781683	14.97680308	profile #4
Gpatch3	1.885997114	1.306023247	3.661388811	profile #4
Sytl1	0.14941909	0.122782048	0.253435875	profile #4
Hgf	0.058901604	0.06146181	1.50968217	profile #4
Cdca8	0.041103184	0.059718987	4.736961909	profile #4
Fgr	0.059907569	0.126944242	7.386614035	profile #4
Smpdl3b	0.891049826	0.836652722	1.918772884	profile #4
Sesn2	0.932351413	1.254383651	4.359344782	profile #4
Inpp5b	2.756945839	2.562318333	5.784649169	profile #4
Rcc1	2.213192358	2.134865117	8.422292482	profile #4
Sf3a3	5.961030324	5.690743195	14.33754152	profile #4
Utp11	4.552223366	3.758940608	8.214674656	profile #4
Casp9	2.104573193	2.411405939	4.353263413	profile #4
Necap2	5.174302892	4.806148669	26.8211178	profile #4
Cdk14	0.37868878	0.32905393	1.839999806	profile #4
Padi2	0.144322766	0.124797	1.209343621	profile #4
Kcnab2	0.312816012	0.317775939	6.529261328	profile #4
Nol9	2.222706204	2.129899755	5.021773572	profile #4
Ube4b	5.063096889	5.082286412	10.14942495	profile #4
Pgd	4.458310711	4.334387379	24.13220726	profile #4
Slc4a2	4.560605884	4.291246515	8.78266551	profile #4
Tnfrsf9	0.03400778	0.057458428	0.377081401	profile #4
Abcb1b	1.491402545	1.138845733	2.515724448	profile #4
Cort	0.068367191	0.077007307	0.189515431	profile #4
H6pd	6.258564251	7.554960359	14.16392756	profile #4
Mthfr	1.739564297	1.742997379	6.033611717	profile #4
Nppb	248.9193677	230.3675174	1218.464384	profile #4
Ssu72	11.18816743	10.70209691	23.02082539	profile #4
Mmp23	2.873590355	2.517579478	20.25050671	profile #4
Nadk	11.32125753	10.77530872	26.75083349	profile #4
Gnb1	27.08502826	27.93303426	73.73474009	profile #4
Mxra8	9.612220814	10.12066864	45.9330936	profile #4
Cptp	1.89648921	1.729755234	4.845953746	profile #4
Bst1	0.432566243	0.292933992	8.094044823	profile #4
Cd38	5.416269154	3.991999386	11.96165537	profile #4
Pacrgl	0.417291552	0.341329908	0.849195383	profile #4
Trmt44	0.465369147	0.55700809	1.052442382	profile #4
Htt	0.900641631	0.901835746	1.951287213	profile #4
Rnf4	4.096824381	4.035068745	12.02891058	profile #4
Man2b2	5.431354715	5.31979048	19.11679612	profile #4
Crmp1	0.072258085	0.096188094	0.610856377	profile #4
Evc	0.973653195	0.948097239	2.48217649	profile #4
Nsg1	1.345794271	1.410778651	4.962298086	profile #4
Spata18	0.133924039	0.12930064	0.440595162	profile #4
Cgref1	0.555770749	0.550887045	3.196449978	profile #4
Emilin1	8.578164478	7.427576681	78.20167673	profile #4
Dpysl5	0.034618956	0.023951441	0.114417029	profile #4
Pgm2	2.466128628	2.524507638	10.89398234	profile #4
Tbc1d1	2.201189026	2.26051143	5.737023532	profile #4
Fam114a1	4.646075134	4.119502762	27.53927751	profile #4
Pi4k2b	0.565934001	0.634305497	6.243313829	profile #4
Sel1l3	0.138462954	0.1216975	0.552630766	profile #4
Tbc1d14	1.593437354	1.545510579	5.389782127	profile #4
Ugdh	1.556854934	1.497924417	9.734835307	profile #4
Rhoh	0.05912248	0.055456282	0.665595215	profile #4
Commd8	5.782200193	5.183402044	11.45460121	profile #4

Tec	2.762193261	2.619581243	6.84123376	profile #4
Uchl1	5.473606303	6.165350315	23.32180194	profile #4
Chic2	1.572478838	1.848196348	3.583499105	profile #4
Pdgfra	3.654838784	4.212203984	11.09089322	profile #4
Srd5a3	1.436792493	1.371560437	6.050828258	profile #4
Tmem165	7.104002451	6.947761072	24.11490088	profile #4
Clock	1.733101059	1.648294083	4.144143005	profile #4
Rest	2.083839756	2.262751181	5.0492664	profile #4
Cenpc1	0.883797252	0.928430924	2.880241722	profile #4
Stap1	0.096814102	0.092272472	0.740060022	profile #4
Dr1	7.017939265	7.200224562	14.99582875	profile #4
Mtf2	1.613299258	1.518819788	3.623861065	profile #4
Dipk1a	1.574595902	1.752184632	4.712687923	profile #4
Glmn	0.774853594	0.648658213	1.554130592	profile #4
Brdt	0.116483933	0.127829419	0.356459452	profile #4
Cdc7	0.172959605	0.190054082	2.118424592	profile #4
Abcg3	0.078227473	0.076742662	2.18711213	profile #4
Spp1	0.057074828	0.158173382	1007.735037	profile #4
Plac8	0.080197683	0.205354108	16.57502119	profile #4
Enoph1	3.615695197	3.366094033	6.867314235	profile #4
Prkg2	0.103046578	0.10681196	0.294727263	profile #4
Asphd2	0.18764951	0.231295659	0.738864441	profile #4
Rfc5	0.664182351	0.577030171	3.569889725	profile #4
Dck	0.820935589	1.008757019	7.691477663	profile #4
Cxcl5	0.114219637	0.175482415	12.4122148	profile #4
Pf4	18.29557224	22.21811819	266.7599549	profile #4
Ereg	0.020619301	0.010699239	0.993799108	profile #4
2010109A12Ril	0.066758019	0.042735129	0.134171971	profile #4
Ccng2	2.783673612	2.705396767	5.657384	profile #4
Tctn2	0.367249676	0.432334832	0.801529941	profile #4
Rilpl2	1.816006765	1.859554787	6.181726162	profile #4
Uso1	6.732311299	6.970496462	15.03037159	profile #4
Naaa	3.682814373	3.548984304	10.95330604	profile #4
Kntc1	0.011782452	0.020476246	1.341830054	profile #4
Sdad1	2.151119457	2.033678509	4.121020526	profile #4
Slc15a4	1.603231211	1.682213015	3.969180458	profile #4
Stx2	4.339810912	4.526746061	10.88509625	profile #4
Ran	39.5826988	39.09215492	89.80358348	profile #4
Mmp17	0.802893399	0.790267386	1.597685838	profile #4
Psph	0.930182295	1.095032159	1.910513297	profile #4
Vps29	6.680009534	5.911356919	13.73392935	profile #4
Fam216a	2.067467188	1.766518763	3.648155648	profile #4
Arpc3	20.04643961	18.85655784	71.45950506	profile #4
P2rx7	0.573711811	0.606641451	4.855835716	profile #4
P2rx4	2.259873834	2.337678749	11.72524553	profile #4
Camkk2	1.63591713	1.506744944	4.217599021	profile #4
Kdm2b	0.763534474	0.822146981	1.652615011	profile #4
Aacs	0.984778468	0.961374082	4.837348532	profile #4
Anxa3	9.058718433	7.710641381	39.54448507	profile #4
Mfsd7a	0.581307581	0.623611803	1.790497663	profile #4
Gpc2	0.04331735	0.024395857	0.100063911	profile #4
Cit	0.098675798	0.061876622	0.716344102	profile #4
Chek2	1.770860184	1.295117695	4.131413748	profile #4
Pxn	9.002761841	9.399182517	19.84152946	profile #4
Srsf9	12.51848139	13.25260895	45.66874385	profile #4
Psmg3	0.959929236	0.738849573	2.261804109	profile #4
Tes	0.591741131	0.536330054	3.444905074	profile #4
Tfec	0.02482191	0.037278475	1.689591839	profile #4

Mad1l1	2.34657407	2.396535614	4.972515796	profile #4
Snx8	4.168471893	4.33107834	18.90056474	profile #4
Oasl2	1.475671745	1.025515471	8.73863201	profile #4
Foxp2	0.129471102	0.130001487	0.697001718	profile #4
Tmem168	3.821954821	3.517363271	8.446644185	profile #4
Lfng	4.264318348	3.878890421	25.25081976	profile #4
Radil	0.481829981	0.386215113	0.816171915	profile #4
Actb	105.8204933	100.6975433	822.7076226	profile #4
Fscn1	13.64565543	12.31933745	73.17071396	profile #4
Oas1b	1.086026251	0.62811984	2.70516153	profile #4
Rpl6	95.10774888	89.62696273	217.1532893	profile #4
Erp29	3.941099422	3.843871567	15.1788106	profile #4
Arpc1b	11.7634339	12.38215036	106.3050921	profile #4
Cpsf4	3.18181371	2.93379188	7.35339611	profile #4
Usp12	5.312252868	5.360145549	12.41991099	profile #4
Medag	2.480367204	2.851079121	15.93587424	profile #4
Col1a2	7.725272456	8.118395854	328.9911724	profile #4
Tfpi2	0.662343847	0.499024979	5.924264669	profile #4
Fam3c	7.169078026	6.613274683	18.56190834	profile #4
Limk1	0.885365618	0.958586781	3.206652958	profile #4
Eln	6.516087533	6.519904158	38.57028852	profile #4
Ezh2	0.630917259	0.61194171	3.2012693	profile #4
Ssc4d	0.126423813	0.089045276	0.505448364	profile #4
Lrwd1	1.422081371	1.276504528	3.579725362	profile #4
Gnb2	40.03465345	40.3769717	84.05122639	profile #4
Pcolce	15.39994663	13.49711313	92.48166313	profile #4
Ppp1r35	11.51782374	10.54592707	26.36989322	profile #4
Mcm7	2.061399401	1.649637786	7.91973613	profile #4
Tpk1	0.4715338	0.378794976	0.889226381	profile #4
Asns	0.793590524	0.667162024	3.833242434	profile #4
Pon3	3.646052792	4.442685968	10.09946509	profile #4
Cald1	5.596741146	5.391968404	20.03711171	profile #4
Akr1b8	1.76024729	1.708908937	28.46230038	profile #4
Calu	48.98628594	51.96165177	135.8963056	profile #4
Ccdc136	0.363325973	0.315911436	0.999149511	profile #4
Irf5	1.100866361	1.012844631	18.47606922	profile #4
Gars	16.3874455	15.21060965	32.97004215	profile #4
Adcyap1r1	0.981645557	0.95751032	4.301999328	profile #4
Fkbp9	41.36736709	41.23206829	97.21646352	profile #4
Tmem209	0.840598747	0.703512065	1.808305671	profile #4
Avl9	1.10593609	0.889499073	2.235114685	profile #4
Cep41	0.444472448	0.345535297	1.034503821	profile #4
Herc6	0.450456264	0.331026997	1.167517873	profile #4
Tmem176b	11.18253125	10.00461651	53.19562799	profile #4
Gpnmb	0.187714326	0.237308258	70.14263425	profile #4
Tra2a	5.578142572	5.006960756	10.09811584	profile #4
Gsdme	0.377419618	0.291161699	0.644429807	profile #4
Zc3hav1	2.734618069	2.981472999	8.569210523	profile #4
Cbx3	5.533362691	5.89248395	11.76070798	profile #4
Ptn	0.7562052	0.952297522	42.98366156	profile #4
Mtpn	11.64978	11.5854288	43.5333532	profile #4
Slc13a4	0.051932043	0.06016706	0.11073427	profile #4
Tcaf2	0.658717452	0.785609913	2.349184196	profile #4
Epha1	0.335241965	0.225630625	0.60971733	profile #4
Zyx	9.179633503	10.10895916	33.34105013	profile #4
Fam131b	0.2177299	0.252255972	1.813234141	profile #4
Casp2	1.272873002	1.122907737	2.93891524	profile #4
Ephb6	0.39478884	0.375475626	2.802831318	profile #4

Mad2l1	0.381170057	0.442190986	4.357206966	profile #4
Clec5a	0.551128811	0.567575936	4.866855076	profile #4
Hpgds	0.216114808	0.199350544	6.363510307	profile #4
Smarcad1	0.851202827	0.711664047	1.895553809	profile #4
Mkrn1	6.399169573	6.424255204	14.48057566	profile #4
Rab19	0.20570928	0.207570628	1.194769452	profile #4
Slc37a3	2.755340606	2.592721698	6.236063374	profile #4
Tbxas1	0.300284879	0.27517368	4.71691418	profile #4
Gfpt1	2.067917455	2.030288872	6.621012241	profile #4
Anxa4	3.452554047	3.99352247	21.62993709	profile #4
Add2	0.004197479	0.008712192	0.089914691	profile #4
Kbtbd8	0.166215168	0.139080064	0.447499042	profile #4
Wdr54	0.183389102	0.157765698	0.354266211	profile #4
Mogs	4.561913361	4.540607488	16.360435	profile #4
Pole4	8.997304137	9.488130824	19.58535099	profile #4
Arhgap25	0.933380683	1.069956958	6.109714526	profile #4
Aplf	0.460374716	0.387621225	1.175106338	profile #4
Copg1	4.333794409	4.305144145	11.09026013	profile #4
Tmf1	3.674020856	3.849852127	7.809101863	profile #4
Hmces	2.808314986	2.686657376	5.764334605	profile #4
Rpn1	14.5152243	14.44299616	47.41079579	profile #4
Frmd4b	1.190933323	1.114815475	3.438907171	profile #4
Foxp1	1.312245833	1.558217737	3.649629839	profile #4
Gxylt2	0.52510163	0.581274168	4.368455615	profile #4
Sec61a1	4.049557685	3.785508853	20.2790918	profile #4
Plxna1	3.793148255	3.878101744	11.77566693	profile #4
Slc41a3	5.160789291	6.518982	14.70344728	profile #4
Nup210	0.251447477	0.274090099	0.909556116	profile #4
Tmem43	7.455861144	7.190889201	19.61884008	profile #4
Sumf1	5.211977912	4.822964233	15.56891617	profile #4
Edem1	2.345430076	2.16159092	19.89203084	profile #4
Usp18	1.433861745	1.132710685	6.591378391	profile #4
Slc6a12	0.015409456	0.017852978	0.074676036	profile #4
Mfap5	6.56931269	6.10093553	71.50798439	profile #4
Ptms	140.6834204	136.2760114	467.8726811	profile #4
Plxnd1	24.83176705	20.59038936	43.2285325	profile #4
Lag3	0.24215213	0.296636966	1.102273529	profile #4
Clec4d	0.106462131	0.414319483	26.54795643	profile #4
Clec4b1	0.419907665	0.394343365	1.800154866	profile #4
Clec4a2	0.610883783	0.5283069	9.099899933	profile #4
Klrk1	0.029624277	0.022756769	0.22323347	profile #4
Clec2e	0.110253902	0.05191738	0.241240593	profile #4
Clec2d	33.80720938	29.56979005	78.12489925	profile #4
Magohb	2.406168575	2.515766033	5.061750124	profile #4
Etv6	2.823937832	2.492112445	8.487151797	profile #4
Fam234b	0.770070547	0.696490711	1.658223464	profile #4
Emp1	9.28031335	9.83249999	87.48023783	profile #4
Mgp	95.64414122	102.4103991	279.5908754	profile #4
Arhgdib	8.107259411	7.296391815	59.20434433	profile #4
Ptpro	0.275380562	0.307975162	2.092882171	profile #4
Dera	0.643553395	0.538852162	3.16204912	profile #4
Recql	0.887005786	0.942588266	1.944716284	profile #4
Golt1b	4.906571895	4.176456568	15.94893573	profile #4
Rad18	0.231582652	0.243845172	1.604344288	profile #4
Srgap3	0.069116097	0.085176886	1.087274596	profile #4
Lrmp	0.450652747	0.512372941	5.527176415	profile #4
Bcat1	0.13117047	0.095073707	2.855614183	profile #4
Ogg1	2.558998336	2.341931983	4.793743968	profile #4

Camk1	11.12568469	10.42325896	22.85456715	profile #4
Il17rc	3.084483963	3.284333084	7.409357227	profile #4
St8sia1	0.029729957	0.016743586	0.082412127	profile #4
Sec13	15.00178858	13.59394714	32.25402472	profile #4
Atg7	2.510768221	2.184717629	4.879451193	profile #4
Vgll4	3.594003406	3.352442593	7.095828768	profile #4
Efcab12	0.052981215	0.039154297	0.095073977	profile #4
Mbd4	0.322410886	0.278628187	1.158819138	profile #4
Ift122	1.067519328	0.914453373	2.714661471	profile #4
Necap1	4.728608964	4.42753306	9.201347586	profile #4
Pianp	0.50326981	0.418272303	1.305070662	profile #4
Cd27	0.079526784	0.093620773	0.195800053	profile #4
Ltbr	12.22982742	11.651389	31.22649727	profile #4
Tnfrsf1a	17.00029919	15.17136079	52.96203977	profile #4
Cd9	16.53191786	17.1782452	42.79468806	profile #4
Rad51ap1	0.107485195	0.090566865	2.519193672	profile #4
D6Wsu163e	1.350876832	1.076252482	2.546532086	profile #4
Tspan11	0.326717492	0.296682577	0.610058022	profile #4
Clec2i	0.030244153	0.017520029	2.143770435	profile #4
Zik1	0.395277742	0.220596315	0.738174128	profile #4
Vasp	20.62049843	18.64880963	63.67929494	profile #4
Qpctl	1.256449373	1.075980967	2.728773038	profile #4
Zfp583	0.179388399	0.126350322	0.62533265	profile #4
Cyfip1	3.355604141	3.490727472	10.66168922	profile #4
Nipa2	1.969549932	2.189359128	5.15820717	profile #4
Zfp719	0.816324013	0.653613017	1.817565474	profile #4
Zdhhc13	3.687402862	3.628613466	7.338553502	profile #4
Siglece	1.195453732	0.979353559	4.130696084	profile #4
Faap24	0.757649182	0.718884343	3.415294278	profile #4
Kctd15	0.753154263	0.745696747	3.285750379	profile #4
Mphosph10	3.190390347	3.113946547	6.443112978	profile #4
Blm	0.135767419	0.105535368	0.815359876	profile #4
Vps33b	2.276284249	1.89944168	4.03161956	profile #4
Iqgap1	3.344230161	3.185948077	19.83646576	profile #4
Cib1	5.828280731	4.968445884	20.17320771	profile #4
Ctsc	5.803395941	6.041634757	63.44080696	profile #4
Nox4	0.065566646	0.051457717	0.831885144	profile #4
Cd22	0.092848196	0.183953906	1.620102594	profile #4
Tyrobp	11.48498147	12.58027382	301.4230145	profile #4
Sipa1l3	0.729705676	0.714209719	1.547425071	profile #4
Dpf1	0.043740382	0.029092604	0.39245722	profile #4
2200002D01Ril	0.952390212	0.905798936	9.70517267	profile #4
Rasgrp4	0.243906763	0.326233455	1.826728645	profile #4
Psmd8	25.69347264	24.56099988	49.70633705	profile #4
Ryr1	0.012716711	0.016179671	0.048007684	profile #4
Fbxo17	0.463401962	0.417572661	2.41497457	profile #4
Pak4	2.448435066	2.323892779	7.791273339	profile #4
Zfp626	0.938025053	0.70438059	2.118703569	profile #4
Acan	0.037779884	0.02365321	0.549257293	profile #4
Sytl2	0.070843041	0.056094197	0.360192457	profile #4
Ccdc83	0.028482823	0.032082429	0.154278393	profile #4
Eed	4.005138106	3.800445766	8.870754596	profile #4
Sh3gl3	0.076223109	0.084495862	0.274401149	profile #4
Ddias	0.05001036	0.035791294	1.046594126	profile #4
Anapc15	0.744355901	0.747512508	2.243342785	profile #4
Arl6ip1	13.71856275	11.72288992	74.56730194	profile #4
Xylt1	0.330729227	0.393567018	2.304853298	profile #4
Nucb2	3.057840649	2.921883841	19.18326453	profile #4

Pik3c2a	1.301098969	1.223553621	2.771866064	profile #4
Cyp2r1	0.160976783	0.098598481	0.698295514	profile #4
Pde3b	0.417972607	0.274569831	2.000121148	profile #4
Kif22	0.104160482	0.099088522	4.500904519	profile #4
Maz	27.03889304	28.67717431	59.9978302	profile #4
Mvp	8.194888704	7.382281689	20.4513528	profile #4
Ino80e	1.858052965	1.727599502	3.750815904	profile #4
Ppp4c	7.624055828	7.440747825	22.68735663	profile #4
Gdpd3	0.344224399	0.348957941	0.750668384	profile #4
Coro1a	3.819153008	4.443624835	73.5680365	profile #4
Dnajb13	0.05082495	0.096998075	0.92359869	profile #4
Nupr1	1.454448072	1.638258469	12.25680296	profile #4
Cln3	0.829791587	0.848271248	4.11678052	profile #4
Nfatc2ip	0.983615802	0.808240946	2.216961646	profile #4
Pold3	1.590962127	1.967859955	5.70452819	profile #4
Rabep2	1.952941475	1.966826432	4.773353151	profile #4
Lat	0.123045137	0.159618565	0.442252624	profile #4
Rps3	87.80959648	84.75400482	207.2635956	profile #4
Il21r	0.294476368	0.225881398	5.509475975	profile #4
Il4ra	2.805167901	3.086584231	28.55535973	profile #4
Copb1	6.549180491	6.543471839	21.07627974	profile #4
Far1	2.673428662	2.62125989	9.637067148	profile #4
Acer3	0.530940415	0.541848988	1.879925837	profile #4
Myo7a	1.421980102	1.216820431	6.093589704	profile #4
Arhgap17	1.451284346	1.280777465	3.638546433	profile #4
Disp1	1.670068638	1.5133606	3.24590312	profile #4
Slc5a11	0.017052088	0.016013944	0.077533205	profile #4
Parva	3.931099741	3.716154122	8.971046824	profile #4
Dkk3	0.895652939	0.907259756	11.98247362	profile #4
Pak1	0.194280564	0.234918572	6.410348261	profile #4
Rusf1	0.97021011	0.933995788	2.430137007	profile #4
Tgfb1i1	2.834257672	2.565829406	11.74867243	profile #4
Itgam	0.383800955	0.477341073	12.47523827	profile #4
Itgax	0.088607614	0.056722142	4.773656666	profile #4
Pycard	1.343944323	1.304827205	4.852655037	profile #4
Tead2	1.620696252	1.491719026	5.102770089	profile #4
Cd37	0.841060089	1.021056539	12.50719338	profile #4
Fbxl19	1.854644434	1.631499652	7.702259835	profile #4
Bcl7c	3.724863565	3.840214697	7.90561705	profile #4
9130019O22Ril	0.668369502	0.491762544	1.036649734	profile #4
Nucb1	11.45842273	11.19220612	25.16459681	profile #4
Itgal	0.252667261	0.282016723	1.408615808	profile #4
Sergef	0.757669987	0.799439523	2.190040903	profile #4
Rgs10	2.116472066	1.919601778	25.4704235	profile #4
Fgfr2	0.031370267	0.012829141	0.2981455	profile #4
Chp2	0.635222044	0.74391412	2.047362554	profile #4
Plk1	0.085547355	0.180763125	11.96041821	profile #4
Dctn5	7.670416812	6.671728174	16.53472493	profile #4
Ubfd1	3.799883094	3.302700955	7.02041692	profile #4
Cdr2	1.726307646	2.063711824	6.591944316	profile #4
Polr3e	2.371876261	2.15823461	4.560702862	profile #4
Arfip2	4.758758131	4.254096018	9.910989501	profile #4
Dnhd1	0.055243192	0.043929239	0.090746907	profile #4
Rrp8	3.004985899	2.446984008	4.996368309	profile #4
Ilk	0.884045009	0.714847826	1.558575642	profile #4
Tpp1	7.227647902	7.090238714	20.73078811	profile #4
Crym	0.0251007	0.03074842	0.252240139	profile #4
Trim30a	2.928628698	2.358772583	13.9402099	profile #4

Rexo5	0.212936206	0.249371463	0.924608633	profile #4
Eri2	0.201810328	0.220380949	0.835700704	profile #4
Eef1akmt2	2.492650715	2.328503677	5.9803005	profile #4
Abraxas2	2.138981024	1.838710625	3.943598059	profile #4
Trim21	2.000723221	1.530048517	3.964207736	profile #4
Ctbp2	2.389288117	2.221140816	5.047358238	profile #4
Rrm1	1.306853932	1.307506804	7.616358923	profile #4
Knop1	2.807382676	2.362969203	6.651287853	profile #4
Vps35l	1.438372953	1.284469594	3.037157179	profile #4
Mki67	0.126900617	0.100966952	12.48177173	profile #4
Atp6ap2	6.735839584	6.826520655	29.43152124	profile #4
Cask	1.131385989	1.221420932	2.826149494	profile #4
Swap70	1.976524585	1.736085695	4.991659989	profile #4
Akip1	6.587558509	4.77200798	13.69864975	profile #4
Tub	0.010143782	0.006493539	0.04889051	profile #4
Mrgprf	1.326340717	1.13207558	3.411591264	profile #4
Cttn	12.5538112	12.49556723	36.17656341	profile #4
Dock11	0.478440249	0.524724788	2.982104027	profile #4
Cul4b	2.692168356	2.799860809	7.79123447	profile #4
Tnni2	1.051219768	0.885099335	2.618619712	profile #4
Sash3	0.316224824	0.366369525	10.16063232	profile #4
Elf4	1.774704094	1.595268734	7.309662548	profile #4
Rab33a	0.079938839	0.038140291	0.182197756	profile #4
Slc25a14	0.73810817	0.639545032	1.53774189	profile #4
Rbmx2	1.260958327	1.274032409	3.381176661	profile #4
Enox2	1.753204091	1.743946413	3.568307639	profile #4
Stk26	0.114152845	0.079125702	0.769649111	profile #4
Slc9a9	0.489007797	0.460537051	4.6013083	profile #4
Cacna1f	0.005174349	0.009718661	0.112929428	profile #4
Plp2	17.93788006	16.53122898	66.97242485	profile #4
Praf2	1.970292395	1.418340287	13.23580294	profile #4
Ccdc120	0.296323858	0.175240157	0.646900572	profile #4
Slc35a2	2.90685613	2.523306509	6.736886286	profile #4
Was	0.983075611	1.050999346	12.19300135	profile #4
Rbm3	10.0824431	10.10941368	38.10164012	profile #4
Ebp	4.173627703	4.378406327	9.535426047	profile #4
Aff2	0.064716745	0.089974883	0.229520477	profile #4
Msn	29.69324209	29.26312801	101.0489446	profile #4
Ophn1	0.756099377	0.700425544	1.602958165	profile #4
Pbdc1	0.879203193	0.866721403	4.305571892	profile #4
Magee1	1.364522486	1.10509433	2.811975572	profile #4
Itm2a	6.687910653	6.18791684	22.09481156	profile #4
Sh3bgrl	10.76634086	11.72934762	66.41077104	profile #4
Srpx2	2.566085988	2.265917602	26.58184741	profile #4
Cstf2	1.403717476	1.477026389	2.934543702	profile #4
Cenpi	0.016556824	0.018353792	1.275757461	profile #4
Btk	0.358434997	0.311454817	6.180889738	profile #4
Gla	0.69462322	0.814343425	11.57994251	profile #4
Acsl4	2.645618427	2.500450655	7.885181501	profile #4
Pak3	0.01336628	0.010915818	0.305408726	profile #4
Lrch2	0.338843557	0.309577968	1.163375243	profile #4
Adgrg2	0.175386844	0.150624521	0.727534488	profile #4
Map3k15	0.26427608	0.213036782	0.58032926	profile #4
Il2rg	1.997198393	1.638058652	10.8465519	profile #4
Rps6ka3	5.334775009	5.146240066	11.74885021	profile #4
Nono	19.9405752	19.22934298	52.52117359	profile #4
Rps4x	156.0642642	156.5187741	386.4266892	profile #4
Flna	19.64140655	18.43545094	74.90521842	profile #4

Gpm6b	2.644833601	2.9418015	12.77966296	profile #4
Gabra3	0.3355837	0.47272966	3.138885549	profile #4
Nsdhl	1.566286321	1.470667157	3.46699922	profile #4
Zfp185	0.160727088	0.14063649	0.65760372	profile #4
Arhgap6	0.399410853	0.382579259	0.930950802	profile #4
Ctps2	1.803467837	1.674956732	5.757387332	profile #4
Zfp275	1.372411802	1.235960763	3.547914384	profile #4
Ap1s2	2.94864723	3.061823539	12.86732296	profile #4
Zrsr2	1.876217958	1.847943218	3.918209093	profile #4
Haus7	3.17237613	2.979239439	13.02802903	profile #4
Car5b	0.109264844	0.094328012	0.516512775	profile #4
Bgn	21.50689005	19.00220783	224.3612425	profile #4
Vegfd	0.600905338	0.568588107	4.724296492	profile #4
Piga	0.433247517	0.518791584	1.356402186	profile #4
Renbp	1.22485277	1.191513618	14.8890779	profile #4
Arhgap4	0.252041378	0.326957114	2.358666265	profile #4
Plxna3	0.149205769	0.110634091	0.621174116	profile #4
G6pdx	1.833350564	1.769067853	16.39928876	profile #4
Mpp1	1.998078323	1.859901327	12.58881125	profile #4
Morf4l2	17.90851842	18.27237465	82.28069106	profile #4
Psmd10	7.56181152	6.824820513	16.08599059	profile #4
Morc4	0.576727058	0.491098159	1.258012916	profile #4
F7	0.074692719	0.205169999	7.331411229	profile #4
F10	0.033895707	0.039902812	3.354827073	profile #4
Proz	0.108433888	0.103347164	0.220528565	profile #4
Lamp1	93.08825551	93.20891155	210.9747652	profile #4
Rasa3	5.436794673	5.157116612	19.88799747	profile #4
Angpt2	1.455016859	1.005415278	4.139651522	profile #4
Erlin2	4.34070274	4.159784664	9.071095009	profile #4
Adgra2	4.022969922	4.179717646	19.95358167	profile #4
Rab11fip1	0.039191382	0.040360955	0.523351787	profile #4
Ggn	0.197591814	0.194597464	1.06918289	profile #4
Cd209a	0.059418394	0.137681086	3.196228692	profile #4
Col4a1	49.70136293	42.65397034	161.9359424	profile #4
Col4a2	50.37530926	43.99978309	134.4660228	profile #4
Ptpn7	0.177266822	0.188188771	4.078647955	profile #4
Gpm6a	1.140999491	0.975371209	2.311013109	profile #4
Eri1	1.012930173	1.034683651	3.582637566	profile #4
Ikbkb	1.233604414	1.237901135	2.73601475	profile #4
Plat	3.32987983	2.889808195	11.69745357	profile #4
Ap3m2	1.278579779	1.168014832	3.090588332	profile #4
Gins4	9.65694248	11.90308049	24.03085636	profile #4
Sfrp1	2.452598842	2.98112687	52.27571218	profile #4
Adam5	0.059149633	0.049968639	0.314264619	profile #4
Adam9	11.40746909	12.96551151	45.70410048	profile #4
Slit2	0.227482936	0.26994057	0.991113341	profile #4
Tenm3	0.353744623	0.414181875	2.502415343	profile #4
Dctd	0.287133895	0.336851827	1.818476483	profile #4
Fgfr1	4.105272469	4.087913068	11.81215027	profile #4
Plpp5	3.992850458	3.242378776	11.83132087	profile #4
Star	0.641434845	0.468990038	6.627324811	profile #4
Mak16	2.480638929	1.909327213	5.252443557	profile #4
Wrn	0.834694919	0.788943252	1.962010865	profile #4
Gsr	7.684584722	8.052406308	23.51938834	profile #4
Gtf2e2	5.460319795	5.116386823	11.76441438	profile #4
Asah1	8.524201433	8.338210141	44.08901922	profile #4
Pdgfrl	0.681091186	0.633745993	7.842756618	profile #4
Slc7a2	0.354671172	0.353837405	1.989721854	profile #4

Msmo1	1.069728434	0.848534289	2.967594559	profile #4
Galnt7	1.621238187	1.804833131	4.625041032	profile #4
Sap30	7.190220378	7.81693717	18.55521746	profile #4
Hpgd	2.514955	2.046930263	8.258691368	profile #4
Casp3	0.896186542	1.056034667	11.67739449	profile #4
Cenpu	0.042568879	0.039110238	0.718100001	profile #4
Ufsp2	3.777739757	3.371494171	8.957166396	profile #4
Pdlim3	2.958544678	3.089344034	10.13719158	profile #4
Tlr3	1.537782548	1.511854418	3.168960126	profile #4
Adcy7	2.385378292	1.869514362	16.54538554	profile #4
Nkd1	0.680357042	0.597710507	1.499761749	profile #4
Snx20	0.14376328	0.12346579	3.637107498	profile #4
Eif2ak3	1.506260221	1.591397779	4.054445287	profile #4
Gins3	0.67552757	0.505411651	2.439803101	profile #4
Cdh11	0.488628849	0.503539917	3.136461938	profile #4
Smad1	2.409577721	2.637856094	6.412961416	profile #4
Slc10a7	0.750004445	0.680946855	2.265820712	profile #4
Tnpo2	4.448033597	4.109303961	8.964147016	profile #4
Orc6	1.504796688	1.288039996	6.609334292	profile #4
Tbc1d9	0.841252067	0.795975357	7.117652679	profile #4
Zfp330	2.83275354	2.529835477	6.100614508	profile #4
Hp	0.263553503	0.551853732	22.68851364	profile #4
Txnl4b	2.789229057	2.030910013	4.071564831	profile #4
Zfp821	0.825689962	0.807609642	1.76240705	profile #4
Ist1	6.681995517	6.120681861	12.45552336	profile #4
Mmp2	13.88125734	14.5168894	40.61055392	profile #4
St3gal2	7.239637968	7.140579711	14.89771994	profile #4
Nudt21	5.309516586	5.10245462	10.7437795	profile #4
Cenpn	0.242715299	0.160897839	2.476995502	profile #4
Cdyl2	0.682720053	0.700583155	1.702887722	profile #4
Cx3cl1	2.046328652	1.539644933	6.899358294	profile #4
Ccl22	0.02022781	0.023435393	0.55183633	profile #4
Usb1	4.023522219	3.478076359	8.315443099	profile #4
Tpm4	42.85999071	39.93626056	244.5054366	profile #4
B3gnt3	5.26485866	5.689507517	11.4589754	profile #4
Jak3	7.395209328	5.90041417	13.80029037	profile #4
Mvb12a	6.70807439	5.997860622	13.35696792	profile #4
Mthfsd	1.058083346	0.90123864	1.931114383	profile #4
Gins2	0.214646613	0.203598523	4.138783964	profile #4
Cotl1	7.661473189	8.525664217	240.5704925	profile #4
Klhl36	2.453736175	2.435858458	5.963304507	profile #4
Mast3	1.951032523	1.772591635	6.772389357	profile #4
Ifi30	5.331456111	5.10179295	113.5146624	profile #4
Mphosph6	2.543868765	2.836214312	7.142937779	profile #4
Comp	0.840098382	1.089418502	5.269535754	profile #4
Ntpcr	4.282890283	3.816854295	10.39983358	profile #4
Pbx4	0.038392636	0.024577036	0.209367164	profile #4
Lpar2	0.031718066	0.046674053	0.395654453	profile #4
Bean1	0.401952438	0.405457795	1.517786816	profile #4
Cmtm3	5.82751964	4.644216284	30.63606433	profile #4
Ces2g	0.654925302	0.466784078	1.842799833	profile #4
Cbfb	6.83628685	7.04577024	26.66823734	profile #4
Tradd	2.98774332	2.840286613	14.20134845	profile #4
Ctrl	0.160154284	0.141403001	0.33296353	profile #4
Pla2g15	3.825045607	3.092375229	26.93157372	profile #4
Slc7a6	1.601754212	1.671946834	6.269532433	profile #4
Zfp90	0.15776414	0.201985352	0.553536237	profile #4
Nip7	4.64286405	3.581085796	10.86401288	profile #4

Mtmr2	4.587949188	4.513805612	9.695390552	profile #4
Cep57	2.430240726	2.789540677	5.138342863	profile #4
Mre11a	1.48381072	1.453089098	3.007028969	profile #4
Wwp2	2.428089685	2.463014681	6.440619822	profile #4
Ankrd49	1.454079864	1.196037094	2.620406244	profile #4
Panx1	0.265175521	0.33718473	5.157411151	profile #4
Med17	3.346554896	2.683399387	6.745742321	profile #4
Vstm5	0.085501229	0.082100387	0.529846815	profile #4
4931406C07Rik	0.92390356	0.842044402	2.332848606	profile #4
Taf1d	1.077336014	1.176785924	2.937616628	profile #4
Cfdp1	22.83260248	20.44429721	54.55940231	profile #4
Bmper	1.138520597	1.398193233	6.460550985	profile #4
Tbx20	5.641050145	6.108644233	11.69097197	profile #4
Ccsap	0.301092369	0.489874454	2.158032189	profile #4
Acta1	159.8566012	133.309398	286.2541711	profile #4
Urb2	0.764337047	0.838820205	1.60559888	profile #4
Adamts8	0.160536182	0.27166869	1.719712998	profile #4
St14	0.02786319	0.021403937	0.399896676	profile #4
Birc3	1.750248912	1.717329049	5.805844888	profile #4
Sesn3	1.071865948	0.976508531	2.213622193	profile #4
Thy1	3.182986667	3.463991785	23.83365853	profile #4
Nectin1	0.624860015	0.693982818	3.139649032	profile #4
Trim29	0.027083017	0.025434182	0.0861996	profile #4
Oaf	6.905077285	7.005212618	33.73833955	profile #4
Pou2f3	0.029977065	0.035289696	0.068324159	profile #4
Ubash3b	0.353075514	0.373781098	3.85762988	profile #4
Clmp	0.715793019	0.510336193	5.021037489	profile #4
Rexo2	15.32792412	15.0179001	31.42165847	profile #4
Tirap	1.517085758	1.536565894	3.098002548	profile #4
Abhd12	14.81026086	15.28655103	41.18056532	profile #4
Ppp2r1b	1.128463097	1.180269223	4.619488638	profile #4
Alg9	2.285576101	2.143624415	4.957905059	profile #4
Bco2	0.111023745	0.077177464	0.710205066	profile #4
Cadm1	0.366881353	0.355362296	0.995474478	profile #4
Tagln	0.307538008	0.399897497	1.528261581	profile #4
Il10ra	0.9129893	1.028371161	15.12383345	profile #4
Mpzl2	0.006749377	0.00633847	0.049101396	profile #4
Arcn1	5.951983896	6.4003843	13.74038263	profile #4
Trappc4	8.097209558	7.047838857	16.14328777	profile #4
Chek1	0.082637353	0.078735195	1.337472779	profile #4
Hyou1	5.300641614	5.041769974	12.76050884	profile #4
Stt3a	3.957052953	3.705542032	19.15901334	profile #4
Slc37a2	0.752481636	0.800491128	13.45374781	profile #4
Mcam	18.41712114	15.92330053	48.72538684	profile #4
Olfm2	0.052102726	0.033145559	0.192573279	profile #4
Tyk2	3.027207577	2.635073596	6.497641687	profile #4
Ldlr	1.053453001	0.945684619	3.182435874	profile #4
Ccnb2	0.138988364	0.204313312	24.08624573	profile #4
Myo1e	2.994216861	2.187515669	12.40246419	profile #4
Mns1	0.025891062	0.050799263	1.0709237	profile #4
Tcf12	2.038679773	2.113186775	4.810954454	profile #4
Anxa2	16.20146662	16.89675539	165.0295003	profile #4
Itga11	0.69458454	0.48770794	4.655057546	profile #4
Fem1b	3.472196801	3.300556541	8.793982081	profile #4
Kif23	0.08333315	0.122979629	3.692272061	profile #4
Lca5	0.366988487	0.322426883	0.657854363	profile #4
Sh3bgrl2	0.829768202	0.609647998	1.684660916	profile #4
Elovl4	0.042192568	0.036012749	0.210889469	profile #4

Nnmt	1.402534232	1.765537298	11.88584025	profile #4
Tle3	2.187425977	2.323353475	6.148674723	profile #4
Ptpn9	2.713268741	3.287723524	7.487743849	profile #4
Commd4	5.244321564	4.954939809	13.02240636	profile #4
1700017B05Rik	1.335047333	1.915145311	7.56656683	profile #4
Csk	5.239063615	5.105973908	21.65259864	profile #4
Pstpip1	0.306982105	0.553522174	10.18016405	profile #4
Col12a1	0.074120817	0.058006904	5.293957836	profile #4
Loxl1	16.2564613	13.03926955	163.3329482	profile #4
Nptn	12.9165077	13.1110218	26.66730014	profile #4
Cgas	0.224355734	0.237705094	2.076942185	profile #4
Tmed3	6.487627263	6.105179299	34.97889735	profile #4
Ctsh	7.870294089	8.575043468	59.97310384	profile #4
Plod2	4.675055074	4.618080761	15.42700832	profile #4
Dapk2	1.352504416	1.370769873	2.920275283	profile #4
Snx1	6.581606546	6.178323718	25.11803769	profile #4
Ppib	8.382878132	7.844390368	41.73736583	profile #4
Csnk1g1	0.523856384	0.494790983	1.27231209	profile #4
Spg21	3.507117791	2.963424231	15.46158661	profile #4
Parp16	1.533235503	1.329767229	2.909006331	profile #4
Igdcc3	0.038527681	0.03023712	0.105375417	profile #4
Tipin	3.34318854	3.469228193	9.365213981	profile #4
Rpl4	192.4537047	190.1776383	419.2157397	profile #4
Zwilch	0.064839488	0.06360879	2.330729227	profile #4
Smad3	2.978601612	3.037995772	6.859545024	profile #4
Atp1b3	20.24587462	19.52272014	56.85856518	profile #4
Rasa2	0.426182508	0.378002137	0.809024224	profile #4
Rwdd2a	0.301856009	0.315868347	0.867742636	profile #4
Tbx18	0.837323297	0.861236547	2.978296123	profile #4
Crtap	13.24282512	12.7863388	61.43807045	profile #4
Cmtm6	11.0601726	10.70738603	27.97280817	profile #4
Cmtm7	2.08264479	1.77688495	10.28643684	profile #4
Stt3b	11.80964257	12.94734701	28.66117032	profile #4
Tgfbr2	7.617115814	7.676666432	22.5668503	profile #4
Eomes	0.033418475	0.021259426	0.120675036	profile #4
Copb2	15.58769188	14.9317665	31.08435447	profile #4
Faim	0.736757052	0.55957356	2.873487176	profile #4
Mras	1.208051187	1.161369629	3.134112798	profile #4
Cdc25a	0.744455519	0.665555828	2.136224219	profile #4
Smarcc1	3.042979145	2.901072507	6.53004178	profile #4
Cspg5	0.012673686	0.013152608	0.038207846	profile #4
Kif9	0.032826658	0.063386796	0.407971135	profile #4
Nradd	0.53871186	0.659725029	3.988760669	profile #4
Pth1r	3.593806672	4.046704422	7.559394246	profile #4
Ltf	0.024671442	0.015111289	0.073386088	profile #4
Fbxl2	0.114918732	0.070387867	0.481180419	profile #4
Myd88	3.431815338	3.206183008	15.92216006	profile #4
Rpsa	126.370066	127.4177539	377.9308556	profile #4
Slco2a1	0.321207181	0.286943389	1.124630278	profile #4
Trf	0.84603724	0.738040812	3.323484165	profile #4
Topbp1	1.064095248	1.119212645	4.730386965	profile #4
Uba5	4.209444742	4.219345481	11.43197197	profile #4
Dnajc13	2.761584068	2.915326519	6.300110278	profile #4
Gnai2	75.2673656	72.5377856	194.3032659	profile #4
Nudt16	2.214231834	2.232587996	4.570426861	profile #4
Manf	11.71737803	11.02424451	45.32226056	profile #4
Mst1r	0.517662658	0.608645001	1.283622447	profile #4
Traip	0.074532814	0.042190534	0.805545298	profile #4

Bsn	0.011803152	0.003271047	0.140321309	profile #4
Uba7	3.617280264	2.931175187	6.5186285	profile #4
Ip6k2	4.342677235	3.781537811	8.191723349	profile #4
Eml4	1.696076659	1.641123147	6.228318788	profile #4
Chsy1	2.781952009	2.760403552	13.04377195	profile #4
Fhl3	6.160277905	5.773393051	18.01665286	profile #4
Marchf3	0.531741021	0.589689284	2.129108786	profile #4
Fam189b	1.103524022	1.090044439	4.148980467	profile #4
Oas3	0.039917662	0.030831682	2.31012012	profile #4
Pon2	4.54634933	4.610520098	9.291504636	profile #4
Oas2	1.902961044	1.850632123	5.540327693	profile #4
Nlrp3	0.136370983	0.217729637	1.943734205	profile #4
Lmo2	14.18607897	12.37158292	33.32928382	profile #4
Resf1	1.560188766	1.532236672	4.705448012	profile #4
Syde1	5.76427992	5.561104967	11.35776925	profile #4
Mdfi	0.471513394	0.354079198	4.448972221	profile #4
Sbspon	0.015486986	0.018971596	0.046066594	profile #4
Folr2	2.339877191	2.056911315	11.29984355	profile #4
Ccdc88a	0.539776633	0.745441758	2.276810699	profile #4
Gab3	0.265909146	0.281370568	0.656028305	profile #4
Slc8b1	3.247806782	3.101239683	10.62081762	profile #4
Bet1	4.513838537	3.773770941	9.910072999	profile #4
Cntrob	0.614351395	0.681029034	1.653666229	profile #4
Troap	0.020772817	0.019508152	2.798557017	profile #4
Srxn1	7.467903385	7.392399868	21.20324449	profile #4
Slc10a3	1.697066273	1.481364776	5.644524601	profile #4
Arap1	3.330776279	3.048129714	19.3880526	profile #4
Fanca	0.110943539	0.136824606	0.811646088	profile #4
Prr5l	0.217773736	0.175517745	1.648121213	profile #4
Zswim6	1.376028292	1.423541807	4.443242984	profile #4
Cyb5r4	4.36364854	4.076839978	8.520447973	profile #4
Acsl3	1.079404602	0.963005141	2.877006981	profile #4
Rims3	0.017417682	0.011080396	0.074104115	profile #4
Adgre4	0.148188554	0.149338146	0.770367486	profile #4
Itgbl1	2.451583758	2.46554489	10.36568893	profile #4
Hspa13	2.545378531	2.777410802	7.377921413	profile #4
Nup93	3.555334808	3.683131825	8.18060626	profile #4
Fkbp1a	48.78061972	47.10573095	101.5349518	profile #4
Fam207a	3.956390688	3.8742506	10.87236256	profile #4
Chpf	8.488755788	9.495330062	42.23687446	profile #4
Nfatc1	2.201845979	2.501557693	4.833115177	profile #4
Gmppa	10.91054591	10.32869712	22.67828427	profile #4
Cdo1	0.389421776	0.511117125	1.323827461	profile #4
Cip2a	0.138332981	0.201166155	3.774479763	profile #4
Calhm2	1.284208931	0.899583781	3.824639552	profile #4
Gm7879	0.972189323	1.05595523	2.453313972	profile #4
Ankrd54	2.202794309	1.735426562	4.507094151	profile #4
Gas7	0.787976525	0.887846249	5.204867171	profile #4
Senp1	1.192278886	1.018797132	2.219029891	profile #4
Nol12	2.573152912	2.391133066	6.470793193	profile #4
Lss	0.217992299	0.236818707	0.608495814	profile #4
Slc7a6os	2.78584961	2.483157177	5.639599096	profile #4
Slc35d2	0.575399669	0.474232384	2.17510311	profile #4
Phldb2	3.726223926	3.112694728	11.89337641	profile #4
Cnppd1	6.10926531	5.782380957	12.31789765	profile #4
Lpcat2	0.360912373	0.307756474	6.015688063	profile #4
AA467197	0.141507022	0.045292843	12.76909316	profile #4
Eefsec	1.28889606	1.283884731	2.782779423	profile #4

Rac2	0.945205684	1.058876845	22.57178146	profile #4
Ttf2	0.443777236	0.38733511	2.186427123	profile #4
Wdr3	1.915958415	1.973571825	5.021912074	profile #4
Kctd17	15.97429605	12.91966667	50.48216581	profile #4
Ptprf	0.725741687	0.810042788	4.511418842	profile #4
Lpp	1.901273873	1.965685022	6.982400802	profile #4
Fbxl8	1.072556612	0.892449872	2.806414434	profile #4
Ctdp1	2.775537189	3.246940802	7.764138136	profile #4
Chst2	0.397916081	0.408952094	2.743145922	profile #4
Rtp4	3.994541618	3.033603656	19.38567026	profile #4
Pus7l	0.646943497	0.562919433	2.20537346	profile #4
Prrg3	1.878149045	1.747219505	3.953906568	profile #4
Usp37	0.755462432	0.619564888	1.587956785	profile #4
Atp6v0b	37.72348839	35.25133586	70.9591297	profile #4
Frrs1	0.344265531	0.236972485	3.185117931	profile #4
Ctdspl2	0.950607688	1.119077405	2.135823134	profile #4
Antxr1	0.951219382	0.929303863	5.180996291	profile #4
Armcx2	1.695331335	1.65914519	10.86582069	profile #4
Lpar6	2.490444974	1.881163902	5.235553744	profile #4
Adamts15	0.857725124	0.875355276	1.878184096	profile #4
Zbtb1	1.080254666	1.047096475	2.405817492	profile #4
Armcx1	1.424322772	1.149669159	4.262851598	profile #4
Crlf2	8.525108665	8.744663026	46.68646092	profile #4
Cysltr2	0.025685121	0.024509687	0.210750834	profile #4
Fndc3a	1.56392011	1.506838768	5.757372189	profile #4
Cryzl2	3.360838277	3.04491875	7.156014924	profile #4
Cdc14a	0.284419836	0.300353619	0.590085693	profile #4
Casp4	0.539147476	0.666663499	4.023124838	profile #4
Angptl1	0.602791185	0.544113337	1.251466276	profile #4
Dph5	0.583207432	0.571544451	1.175473558	profile #4
Apol6	0.072014251	0.036332934	0.437274176	profile #4
Igf2bp2	0.382946388	0.435531555	2.096617416	profile #4
Ndn	2.543522297	2.385121005	9.161341164	profile #4
Reep4	2.283814352	2.219130825	15.19408414	profile #4
Rfwd3	3.542902729	3.220381843	6.85343654	profile #4
Piwil2	0.242546435	0.170804535	0.417504856	profile #4
Zfp7	0.484742886	0.526360807	1.235603196	profile #4
Gabrb3	0.014377762	0.007969114	0.04576142	profile #4
Qsox1	4.072765023	4.836948837	12.5150674	profile #4
Ucp2	5.151562229	7.65581895	37.15597945	profile #4
Arhgap39	0.831869976	0.935247355	1.68352403	profile #4
Smyd5	1.65168743	1.49050545	3.156233926	profile #4
Adra2a	0.02474594	0.011619694	0.157522341	profile #4
Sfxn5	0.3698924	0.327998216	0.985399592	profile #4
Vav3	1.065117994	1.043470164	3.956188374	profile #4
Rbm4b	0.599274455	0.482498213	1.085838177	profile #4
Recql4	0.217023136	0.236176422	0.957555591	profile #4
Rpap2	1.432405045	1.093434492	2.295533141	profile #4
Tlr13	0.351155709	0.400053523	15.72955847	profile #4
Atp7a	0.41905294	0.505320641	1.523931498	profile #4
Atp6v1h	1.913881487	1.818750449	5.738705492	profile #4
Tmem87a	2.278290312	2.139929534	4.489877426	profile #4
Tcea1	6.45867138	7.480629581	15.60704897	profile #4
Dnah8	0.029024681	0.02422901	0.09689706	profile #4
Engase	1.312468063	1.229747861	2.499709998	profile #4
Lgals3bp	9.445502506	9.961667372	65.42239531	profile #4
Zfp267	0.713476325	0.552885735	1.390018834	profile #4
Cfhr2	0.044033354	0.028187941	0.212229835	profile #4

Map9	0.127591776	0.14067022	0.416710465	profile #4
Ccp110	0.538192884	0.52426269	1.594654082	profile #4
Zdhhc15	0.184835893	0.120257189	0.965115636	profile #4
Rbm34	1.033720175	0.986691366	3.140859195	profile #4
Vhl	3.351269803	2.89071708	7.234911249	profile #4
Trim36	0.271567424	0.25803562	0.793783956	profile #4
Aspm	0.020288895	0.023506165	1.923185629	profile #4
Slc16a2	3.342713236	3.01488129	7.623773394	profile #4
Cdkl4	0.032091203	0.015068733	0.100777551	profile #4
Rnf225	0.026278627	0.016095692	0.118834776	profile #4
Rfc3	0.825000438	0.574861111	3.812196132	profile #4
Tesk2	0.478182437	0.475941929	1.145798698	profile #4
Ttc37	1.13170844	1.171335451	2.653364465	profile #4
Fancd2	0.047402957	0.01313694	0.683417921	profile #4
Lyl1	3.814627856	3.783839574	21.95388868	profile #4
Poglut1	2.921354417	2.88248237	8.026307919	profile #4
Golim4	2.858648859	2.792315732	6.801109195	profile #4
Kctd7	0.858299366	0.698554998	1.81134464	profile #4
Scn11a	0.007175558	0.007446713	0.030741698	profile #4
Vav1	0.663107983	0.720941237	19.46233665	profile #4
Tpst1	8.465001279	8.732814115	18.79512022	profile #4
Tspan8	0.276319968	0.183785946	0.421474151	profile #4
Scx	4.318808388	3.837133089	14.30596825	profile #4
Zfc3h1	3.286224715	2.659101939	6.672588864	profile #4
Nsf	1.977882834	2.200121817	5.417876157	profile #4
Gas2l1	4.325186626	4.286318434	11.2012781	profile #4
Loxl2	3.759284762	2.369012603	40.67840325	profile #4
Vps18	3.498236194	3.355047134	8.129050209	profile #4
Plekhm1	2.915712742	2.745602461	8.732120543	profile #4
Ints14	4.703316389	5.304072464	9.862013112	profile #4
Batf	0.501738889	0.482035069	4.897253068	profile #4
Setd5	2.36380111	1.912364209	4.141582536	profile #4
Jdp2	5.171182417	6.34163057	11.94402989	profile #4
Nipsnap1	0.37282855	0.267397043	1.13012914	profile #4
Kif4	0.061745719	0.085093493	2.703191248	profile #4
Trim59	0.30866721	0.158113807	5.362745268	profile #4
Slc26a2	0.860586519	0.847123952	1.698740369	profile #4
Brip1	0.069402501	0.065177224	0.721586502	profile #4
Plcg2	2.121466691	1.911345038	10.69030024	profile #4
Zbed4	0.7914429	0.680618472	1.566647826	profile #4
Cbl	1.969569335	1.871414411	5.527471193	profile #4
Smc4	2.478579665	2.811830907	14.47083407	profile #4
Cpne2	1.822455653	1.661769551	8.887582775	profile #4
AI661453	0.143095338	0.134046936	0.793361988	profile #4
Cmip	1.860899042	2.070909661	5.8039296	profile #4
Lif	0.097302742	0.112732324	1.720524191	profile #4
Spata6	1.348889987	1.532940135	3.922157081	profile #4
Tbc1d10a	4.905175494	5.322126283	10.28105611	profile #4
Pkd1l2	0.005575785	0.006563943	0.164866173	profile #4
Parp14	3.658873066	2.756275712	8.630670397	profile #4
Gbp8	0.049443203	0.051311597	0.208349987	profile #4
Cyb561a3	4.992706576	4.524514914	10.47768122	profile #4
Eda2r	0.635736691	0.524809367	1.679061072	profile #4
Ifit1	2.422273243	2.100275714	13.31647967	profile #4
Scara3	0.627585101	0.530179356	5.791239743	profile #4
Snx2	17.31196073	17.42084785	59.41770013	profile #4
Gbx2	0.02126072	0.020287762	0.844737975	profile #4
Poglut3	2.948386716	2.662524461	9.382505539	profile #4

Edil3	0.013536474	0.015935451	0.05332427	profile #4
Rsrc1	1.014304646	0.956939898	2.134464933	profile #4
Hdx	0.026783031	0.034290302	0.086058375	profile #4
Washc4	2.891837985	2.50496005	8.895191929	profile #4
Ptpn13	0.279799654	0.310661296	0.589265663	profile #4
Tent4a	1.597890393	1.753187455	3.306987813	profile #4
Hid1	0.648330051	0.57671108	1.768398252	profile #4
8430429K09Rik	0.349449513	0.373931668	0.860367165	profile #4
Slc41a2	0.190364729	0.173169551	2.939965804	profile #4
Myo5a	0.521033844	0.599974887	7.679437876	profile #4
Ppp1r18	5.468818292	5.667875165	29.37270928	profile #4
Chst11	1.386979254	1.462052061	5.872003569	profile #4
Ppm1h	1.294309767	1.097631695	3.478590971	profile #4
Ssh3	2.43730347	2.60504926	10.70743741	profile #4
Cd300ld	1.452408622	1.462607781	15.26364686	profile #4
Cd300a	0.215575704	0.264080777	6.932312872	profile #4
Cacna1a	0.331729119	0.305411329	0.629261606	profile #4
Bmp2k	0.584015438	0.623262632	3.965920903	profile #4
Pbx2	6.731442879	6.37351821	13.7835806	profile #4
Tdg	2.049819863	2.149479987	5.632046075	profile #4
Dbn1	2.799222979	2.852699343	25.47718716	profile #4
Sema3f	3.491159996	3.400694234	7.644955975	profile #4
Fam171a2	1.839569842	2.375848484	6.209818259	profile #4
Prr7	0.865151922	0.882060616	4.129654925	profile #4
Dnaic2	0.046583009	0.034238581	0.110850264	profile #4
Gns	9.947683631	9.771629775	24.36986261	profile #4
Grn	20.73080568	20.69746452	176.732177	profile #4
Ppp1r21	2.530489762	2.168674164	7.552406101	profile #4
Ttyh2	1.6711472	1.380385323	7.274675776	profile #4
Nostrin	3.554695583	2.954625158	6.206697287	profile #4
Nagk	4.132044387	4.147525273	11.21034865	profile #4
Sirt6	2.769430519	2.895554362	6.137646197	profile #4
Glis1	0.012902513	0.024233996	0.152530892	profile #4
Hrob	0.077555505	0.08740064	1.593574788	profile #4
Gpsm3	2.416266892	2.596564818	24.39297376	profile #4
Gna15	0.440558862	0.535940556	4.266715796	profile #4
Ccdc122	0.616005603	0.534002501	1.788255514	profile #4
Zfp661	0.318690813	0.252342433	0.55554618	profile #4
Colgalt1	10.7626819	10.73771302	37.55320102	profile #4
Grip1	0.042220473	0.027027426	0.084855785	profile #4
Nrip3	0.023444496	0.025989006	0.249952531	profile #4
Nup54	3.462999524	2.652326009	5.705735616	profile #4
Tet3	1.915702325	1.863566488	3.775996113	profile #4
Plvap	5.804369502	6.279612168	56.80773823	profile #4
Mfsd12	2.65289436	2.74719588	14.92993842	profile #4
Cxcl10	0.170081764	0.235345241	7.104516252	profile #4
Ankrd27	1.889759942	1.850247366	5.453986671	profile #4
Myl12b	29.82301547	32.78281717	76.83619415	profile #4
Tbxa2r	2.40172305	1.561647182	3.877807669	profile #4
Ncaph	0.204180908	0.229984905	4.381484212	profile #4
Tjp3	0.022917737	0.042111427	0.699566071	profile #4
Dhcr24	1.024863203	0.778505073	3.546247124	profile #4
Dhx8	2.833268951	2.696211283	5.444144218	profile #4
Tmem106a	1.437595001	1.30894853	22.32218628	profile #4
Cebpa	3.871561417	4.237440979	61.89579251	profile #4
Rubcnl	0.51371693	0.393315328	1.306734276	profile #4
Hrh2	0.174330663	0.156071428	0.360971502	profile #4
Vat1	14.50918923	13.73016668	98.65799028	profile #4

Foxn2	1.174299591	1.451611909	3.538881229	profile #4
Dpp4	0.572471932	0.511521553	1.298434157	profile #4
Igsf6	0.851210814	0.670111759	13.60688846	profile #4
Baz1a	0.840871112	1.099626478	4.246872799	profile #4
Creb3l3	0.014110821	0.029288101	1.133510919	profile #4
Ccl5	0.221657484	0.108612389	1.851779005	profile #4
Dhx57	2.516160388	1.974721555	5.103202324	profile #4
Zc4h2	0.603395472	0.651819595	2.337007107	profile #4
Mtmr9	4.884292036	4.489901979	9.096939622	profile #4
Fam167a	0.059744834	0.048791714	0.580370684	profile #4
Dcbld2	1.608748923	1.731711272	5.029652163	profile #4
Shc4	0.089622826	0.105506068	0.486521562	profile #4
Wdr78	0.064362472	0.067111757	0.216558188	profile #4
Elmod2	0.976555771	0.950187752	2.034457638	profile #4
Ap2b1	4.853141267	4.535196425	12.00081003	profile #4
Kcne3	0.185052057	0.234441582	0.793322887	profile #4
Heatr5a	1.287175378	1.420422192	3.291463671	profile #4
Slfn8	0.528810631	0.479477496	5.906510222	profile #4
Leprot	20.81396899	18.84869865	63.67778394	profile #4
Spcs2	10.55637859	10.33980554	27.28159533	profile #4
Pdk3	0.451821437	0.447851163	5.02541394	profile #4
Lcat	0.689785216	0.39493106	1.561548782	profile #4
Pcyt1b	0.035116415	0.020342464	0.401726156	profile #4
Abi3bp	0.973369912	1.127720044	2.411588863	profile #4
Impg2	0.025352916	0.023498562	0.049145286	profile #4
Hpse	0.147134882	0.238669704	7.392876052	profile #4
Tpbg	0.024571399	0.042701638	0.882606566	profile #4
Ssc5d	1.391562777	1.491459871	7.230378412	profile #4
Nat14	1.424456003	1.440769684	3.701017697	profile #4
G2e3	0.608296369	0.700760633	2.589506605	profile #4
Mid1	0.174961527	0.202705691	0.636418387	profile #4
Gnptab	4.99153212	5.217692929	10.25077681	profile #4
Fbxo33	1.254015931	1.335080984	4.081751935	profile #4
Nup37	1.19605606	1.253219873	3.842376012	profile #4
Ccl12	3.804088215	3.495013187	33.68822068	profile #4
Parpbp	0.026171207	0.043517485	1.42048199	profile #4
Rmi1	1.367202799	1.001949158	2.292036403	profile #4
1810055G02Ril	3.197128821	3.097842728	10.58956911	profile #4
Hacd2	2.158148983	2.026449304	4.933965708	profile #4
Brsk1	0.355590101	0.356036709	1.11153074	profile #4
Dennd1a	0.993066303	1.004568133	2.045829618	profile #4
Klf16	2.232876535	2.119176569	6.781354457	profile #4
Oser1	4.610657036	4.059916609	8.643096325	profile #4
Tmem98	9.296598204	8.970997566	23.09906452	profile #4
Myo1d	1.891370644	2.073298268	4.18806774	profile #4
Fignl1	0.077751037	0.056300155	2.660252531	profile #4
Rcbtb1	2.777958198	2.329988934	6.340081746	profile #4
Tgfbi	5.11327499	5.562876318	78.82308885	profile #4
Tstd2	1.452262345	1.308342079	2.742426233	profile #4
Tdrd7	4.572327921	4.240336938	8.741013034	profile #4
Prdm4	2.842451887	2.579522408	5.435734619	profile #4
Capn5	0.489426819	0.576164597	1.677109584	profile #4
Dcaf10	1.783328598	1.989153224	3.783577716	profile #4
Iqcg	0.110636664	0.102059981	0.342327719	profile #4
Fam174c	4.644999308	4.559958671	10.48795188	profile #4
Mboat7	1.985268516	2.150626894	4.210085654	profile #4
Zswim4	3.042273461	3.065390842	6.648583767	profile #4
Sbno2	1.790770011	1.793038612	14.07243033	profile #4

Tnfsf9	0.65000341	0.551009193	3.224110537	profile #4
Isg15	6.428415001	4.068654571	51.06900503	profile #4
Arhgap45	1.587142823	1.67670783	20.7533222	profile #4
Alg8	0.897761405	0.9785792	4.666286617	profile #4
Dok3	1.10765643	1.166070382	14.38331436	profile #4
Usp35	0.320171654	0.412158017	1.204944601	profile #4
Supt16	4.283305073	4.149332695	10.37298196	profile #4
Dagla	0.688692541	0.671291466	1.895625253	profile #4
Xylb	0.072554228	0.039810489	0.385384968	profile #4
Ggta1	5.582851134	5.336284203	14.21964935	profile #4
Acta2	63.64455701	53.68537802	258.0861502	profile #4
Twist1	1.762167794	2.273654924	14.43718009	profile #4
Tk2	2.618170478	2.227707734	5.844427336	profile #4
Plppr3	0.50990595	0.324380679	1.904259635	profile #4
Ddx11	0.300322512	0.246660434	1.142268728	profile #4
AI182371	0.043155793	0.050804	0.119628855	profile #4
Hykk	0.145027805	0.192526885	0.430736169	profile #4
Cerk	8.49309502	8.609338428	26.87621829	profile #4
Uba6	0.829361401	0.811982905	2.905107398	profile #4
Cd276	1.054246035	1.050432407	10.55444718	profile #4
Pknox2	0.918286099	0.902948859	2.050870573	profile #4
Pip4p1	4.948852676	4.726780198	11.383508	profile #4
Apex1	4.638907679	4.099839875	11.12792319	profile #4
Gm7008	0.092578068	0.086941844	0.280625431	profile #4
Fam122b	0.745340383	0.83143931	2.119383476	profile #4
Parp2	6.331370815	5.219226208	12.57885972	profile #4
Zfp57	0.501232992	0.42687595	1.965534315	profile #4
Adamtsl2	1.522938402	1.506529596	6.022126681	profile #4
5031439G07Ril	5.073170159	4.833293017	15.60638746	profile #4
Fmnl2	1.649459046	1.571488221	3.996852566	profile #4
Ptpn23	3.46131238	3.033956322	7.397053803	profile #4
Slc2a6	0.073232106	0.123792623	1.904617294	profile #4
Sigmar1	6.643539131	6.550289629	15.42405419	profile #4
Zranb3	0.304690538	0.350277646	1.435898688	profile #4
Colec12	3.864869588	4.051282539	12.60990066	profile #4
Prr5	2.153442347	1.849300144	6.581587427	profile #4
Mbnl3	0.034073849	0.035777614	0.207534143	profile #4
Mgat5	1.588024776	1.604280325	5.036150132	profile #4
Gxylt1	2.665762266	2.635230122	7.173221406	profile #4
Gmip	0.925358298	1.058022111	7.41803403	profile #4
Rbm43	1.810301	1.696907622	6.890139181	profile #4
Igfbp7	167.1128312	170.976922	846.3821213	profile #4
Gramd1c	0.08301341	0.0831151	0.340911244	profile #4
Slc2a13	0.045777953	0.039072994	0.257206528	profile #4
BC031181	40.58190238	40.201178	80.49817071	profile #4
Srp72	7.568180133	7.705647187	15.93026768	profile #4
Igsf10	0.176071628	0.150309765	1.832271822	profile #4
Tmem260	1.230097233	1.173805405	2.428448231	profile #4
P2ry12	0.609575307	0.491443331	3.347950198	profile #4
Csgalnact1	1.245039968	1.153374977	3.367306805	profile #4
P2ry13	0.567572084	0.366409781	2.791379381	profile #4
Glb1l2	0.020473112	0.023060465	0.055995135	profile #4
Gng12	7.879731232	8.325008048	19.449842	profile #4
9530077C05Ril	0.058109133	0.036380936	0.176142157	profile #4
Arsi	0.505865234	0.366301108	7.460919933	profile #4
Exoc1	1.14506467	0.990924803	3.016862247	profile #4
Calm2	66.61733495	61.20998888	225.3958381	profile #4
Lum	16.88404293	15.58816124	104.0531584	profile #4

Wtip	5.857785021	5.263648234	11.5275901	profile #4
Megf11	0.092536668	0.075052561	0.233987481	profile #4
Marchf1	0.157950154	0.20427519	2.728101616	profile #4
Btg1	4.314320454	4.939008631	18.81109643	profile #4
Fam13b	2.687007112	2.259187592	7.220067061	profile #4
Tmem255a	0.230434852	0.242705077	0.571500877	profile #4
Commd2	3.148178756	2.879008371	7.803483431	profile #4
Card11	0.03865981	0.036306171	1.526902414	profile #4
Slc38a7	3.964160098	3.287119321	7.303719983	profile #4
Adamts2	2.493696205	2.352011334	31.64783207	profile #4
Iqce	0.492293509	0.402871878	1.160356161	profile #4
Ttyh3	4.046006863	3.512896127	23.01384606	profile #4
Fut7	0.039940262	0.018754334	0.254243065	profile #4
H2-Aa	17.39143722	14.35491809	88.02216452	profile #4
Cpz	0.146640361	0.145623046	2.518662826	profile #4
Chst12	2.329580883	2.131657951	12.38941831	profile #4
Plxnb2	21.5451448	21.26739908	48.5724072	profile #4
Eipr1	1.481699496	1.613469167	3.022514229	profile #4
Atp13a2	4.491332387	4.497617521	24.74801997	profile #4
Alg5	2.96458416	2.665625748	9.585967248	profile #4
Mag	0.0313543	0.032539138	0.393783402	profile #4
Clcn7	5.580995	5.007534789	11.03564786	profile #4
Man1b1	6.559344322	6.276499166	14.81359789	profile #4
Tcaf1	1.450295246	1.406837752	4.090957496	profile #4
Cenpt	0.685509532	0.654138417	3.934991802	profile #4
Aaas	3.819929854	3.429223796	7.881512809	profile #4
Cc2d1a	4.244352468	3.75195789	8.369021869	profile #4
Zcchc12	0.06188113	0.045731532	0.684775882	profile #4
Micall2	0.975635257	0.603868109	2.177916501	profile #4
Kif15	0.02520032	0.016031382	1.154033432	profile #4
Anln	0.086818713	0.090180884	4.4259497	profile #4
Rps27l	77.99179965	69.06421437	143.76317	profile #4
Amdhd2	3.613862627	3.115842089	18.00487844	profile #4
Topors	3.384920596	3.393573813	8.002900646	profile #4
Wnt4	0.03044223	0.031592601	1.42376611	profile #4
Proser3	0.470990084	0.438947851	0.888530195	profile #4
Dna2	0.152045419	0.176124278	1.447889725	profile #4
Arhgap33	0.290850587	0.165541442	0.822430291	profile #4
Arhgef26	0.143038756	0.134656213	0.270357369	profile #4
C1qa	67.37902997	65.59431433	750.5663889	profile #4
Rap2b	0.545085682	0.483503407	5.615499528	profile #4
C1qc	31.02837127	29.06365021	380.0488162	profile #4
Zfp157	0.674610256	0.440514414	1.143576187	profile #4
Neto2	0.12498748	0.129710593	0.376804543	profile #4
C1qb	43.83095011	45.22244737	713.4830383	profile #4
Unc93b1	8.012092473	6.964650709	79.36264973	profile #4
Ttc7	2.884390403	3.285718256	8.967280196	profile #4
Stag3	0.008073363	0.009093659	0.166967958	profile #4
Rab8b	3.178214896	2.827474418	27.10308071	profile #4
Map11	1.660325746	1.368736319	9.482849029	profile #4
Slc39a12	0.015687011	0.018174546	0.099856974	profile #4
Clca2	0.010122613	0.019012681	0.166788268	profile #4
Spryd3	7.840954339	6.776007348	16.52298251	profile #4
Cnpy4	2.554558614	2.595860948	12.15428396	profile #4
Anapc10	1.147301985	1.095715282	2.425215235	profile #4
Pml	1.595513455	1.703173272	6.3549243	profile #4
Nxt1	5.071904577	4.251065547	14.60066958	profile #4
Apln	4.324749384	3.512518209	10.3207831	profile #4

Ss18	7.275777598	7.625570303	15.08658675	profile #4
Wdr62	0.161186098	0.142314008	1.404850893	profile #4
Zfp146	7.405393516	6.61512357	16.0583348	profile #4
Inhbb	0.500096791	0.882172541	7.048912361	profile #4
Cavin3	8.660954796	7.201630447	45.21832586	profile #4
Rbmxl1	8.146962331	7.565429701	24.66234094	profile #4
Scd1	2.938248246	3.166031524	8.175886953	profile #4
Selenof	17.71593254	16.66736139	63.33538423	profile #4
Trmt12	1.570310398	1.17332901	2.421377172	profile #4
Slc35b2	11.77611096	10.68687907	27.07131229	profile #4
Dcaf15	3.749861515	4.180727974	8.97897267	profile #4
Zcwpw1	0.148592918	0.124334697	0.355530282	profile #4
Fam91a1	2.980790048	3.032876814	14.32909447	profile #4
Psd	0.606136564	0.485219449	1.344179642	profile #4
Aff3	0.415732702	0.485768665	1.171410454	profile #4
Lypd8l	1.687286625	1.363155929	7.887038904	profile #4
Mgarp	0.091010466	0.13180283	0.408292214	profile #4
Ppp1r14a	11.85047974	10.08433382	23.73774195	profile #4
Krt80	0.365267324	0.373448057	4.869784528	profile #4
Grhl3	0.016862552	0.015835947	0.065374331	profile #4
Cyb561d2	1.810665677	1.531797826	5.680463068	profile #4
Islr	13.21678295	11.99507783	29.76592358	profile #4
Syn1	0.08553439	0.079014772	0.652333136	profile #4
Mospd3	6.641769435	5.980123846	15.6046051	profile #4
Zfyve28	0.147874346	0.151458763	0.83150391	profile #4
Spred3	0.253083949	0.177575011	1.499764727	profile #4
Mex3c	3.736706414	3.862796098	11.19435734	profile #4
Itih2	0.085121554	0.093262503	0.891037714	profile #4
Tmem97	3.76237268	2.932406872	17.97782431	profile #4
Galnt6	0.186900033	0.140757659	7.995440298	profile #4
Stag1	2.255101733	2.109006381	4.233668991	profile #4
Ldlrap1	3.012313362	2.961434727	9.636647374	profile #4
Ttc13	1.46895227	1.409199718	3.28848141	profile #4
Man1c1	7.184770508	7.541757002	24.70299978	profile #4
Tacc3	0.423690617	0.368157364	9.637463843	profile #4
Jade3	0.417618667	0.324266843	0.910069927	profile #4
Traf3ip3	0.05628772	0.069058046	0.735574052	profile #4
Tap1	2.739675139	2.676603538	9.895213455	profile #4
Map4k1	0.442594599	0.545442106	3.644209976	profile #4
Slc9a7	0.25444407	0.289047492	1.237189538	profile #4
Taf2	3.162483728	2.803935639	6.113256584	profile #4
Slc12a9	1.021161908	0.911739512	4.523793743	profile #4
Letmd1	3.427823307	3.881239925	8.739191674	profile #4
Sf3b6	6.098920425	6.109296691	12.9222769	profile #4
Ccn3	1.097702869	1.044545466	2.235602666	profile #4
Enpp1	0.514423616	0.503193987	7.914789077	profile #4
Hhat	0.190698111	0.184601747	0.841901711	profile #4
Trmt6	1.677134924	1.48479359	3.471159638	profile #4
Spon2	0.852031211	0.702584667	18.14319039	profile #4
Icam1	2.462849637	3.221314971	8.698264682	profile #4
Tbc1d2b	2.773996223	2.590054748	6.459946445	profile #4
Best1	0.065561792	0.048451623	0.190632097	profile #4
Endod1	2.012806626	1.986589573	9.067384551	profile #4
Vnn1	0.035230329	0.02157862	0.141613628	profile #4
Arid5a	0.535188617	0.79246035	2.370277859	profile #4
Azin1	10.59249278	10.82672557	21.34633487	profile #4
Ints7	1.732798825	1.659681593	3.873775243	profile #4
Fbxo27	0.092154785	0.071178693	0.206712934	profile #4

Klf10	3.104420807	4.276670698	11.06249082	profile #4
Tedc1	0.079196533	0.072761963	2.449290605	profile #4
Uggt1	3.178705939	3.302261342	7.181518801	profile #4
Dtl	0.012417605	0.015898254	0.761422117	profile #4
Cib2	0.361009242	0.472952761	1.64901048	profile #4
Fam168b	12.07506081	11.99576472	28.813154	profile #4
Arhgef4	0.022256164	0.011397822	0.123576614	profile #4
Shank2	0.008596692	0.005060112	0.05503058	profile #4
Dlgap5	0.007072129	0.016387155	2.666390405	profile #4
Plekhg2	3.663543968	3.358860446	8.461749513	profile #4
Rps16	166.1273048	166.8615735	470.3391274	profile #4
Vash2	0.064373558	0.08190349	1.095517857	profile #4
Wdhd1	0.196174883	0.196978523	1.774614944	profile #4
Kcnh3	0.023755644	0.03041434	0.141324031	profile #4
Clba1	0.9918126	0.66497381	2.051085545	profile #4
Nme1	3.183590433	2.828133382	9.929347625	profile #4
Tnfrsf23	0.343731343	0.302645324	3.290389751	profile #4
Kcnk2	0.543092475	0.653915577	1.263206166	profile #4
Cdkn3	0.179629708	0.493416596	10.6142373	profile #4
Slc25a43	0.119545609	0.091832511	0.864956945	profile #4
Zfp60	0.874448954	0.623454091	1.508831954	profile #4
Prkci	2.490852027	2.848422185	5.115788733	profile #4
H2-DMa	2.03076803	1.992222664	14.90731537	profile #4
Esyt3	0.012968503	0.007303719	0.089402008	profile #4
Aspg	0.078871777	0.062718627	0.427212249	profile #4
Ddhd1	1.530056453	1.561737414	3.132093686	profile #4
Tecta	0.00624539	0.007352218	0.022775334	profile #4
Gnpnat1	3.561358737	3.647684677	8.956229053	profile #4
Ckap2	0.043189351	0.032431693	5.983113693	profile #4
Themis2	1.036657281	1.066722521	13.36218175	profile #4
Eef1a1	264.954494	277.7543964	1453.503012	profile #4
Xkr8	0.621681478	0.677064243	1.532798377	profile #4
Ptger2	0.069313089	0.059943617	1.248499738	profile #4
Pced1a	1.487458171	1.353588037	2.942782561	profile #4
Dzip1l	0.565026692	0.575314568	2.049602783	profile #4
N4bp2	0.439743844	0.514031036	1.229067826	profile #4
Rpl10a	70.59926728	67.5466105	192.9718085	profile #4
Fam76b	2.727289844	2.716043093	6.211557579	profile #4
Fbxw17	0.902885592	0.969489318	3.760714907	profile #4
Tgm2	48.47833887	52.3234066	97.9898694	profile #4
Tspan14	6.671967026	6.764648439	27.42008851	profile #4
Rtkn2	0.030689192	0.023886672	0.208169735	profile #4
Ifi206	0.060621595	0.034141419	2.014932573	profile #4
Zfp365	0.155351873	0.178193198	0.753136159	profile #4
Aim2	0.498396947	0.391076881	3.483271378	profile #4
Ackr1	0.1004192	0.077561978	1.034938598	profile #4
Wdr19	0.154716059	0.178757734	0.767014104	profile #4
Pcdh18	1.267952746	1.070003613	3.4063517	profile #4
H2az1	24.33572982	24.77536657	144.8145929	profile #4
Rcor1	2.995203444	3.700227636	6.696286821	profile #4
Sirpa	6.119314148	5.746765612	66.90858105	profile #4
Tmem156	0.02797497	0.016466392	0.491132666	profile #4
Ddx60	0.509072766	0.432248291	1.485464928	profile #4
Fgd3	0.292550623	0.320850969	4.282422488	profile #4
Rflna	0.109686178	0.068298645	0.739535057	profile #4
Zc3h7a	3.274280899	3.175845225	7.992554101	profile #4
Ninj1	43.94070192	48.69150807	113.2380452	profile #4
Sh3rf3	0.115040401	0.120380646	0.70503135	profile #4

Rmi2	0.133859765	0.081187459	0.431826922	profile #4
Rara	3.032621345	2.721243801	7.550050058	profile #4
Dhx38	5.721509369	5.6464063	12.82029528	profile #4
Slc9b2	0.007742084	0.016069295	0.272304174	profile #4
Igsf9	0.252003202	0.165579686	0.619042503	profile #4
Parp11	0.749689973	0.597183803	2.228164976	profile #4
Ccdc138	0.158488142	0.103880338	0.471428659	profile #4
Dnah10	0.002877321	0.002745646	0.010492851	profile #4
Rapgefl1	0.220131033	0.163377769	0.369138359	profile #4
Igsf8	3.050945103	3.397650351	14.23117882	profile #4
Socs1	0.662792214	1.071881112	5.949085292	profile #4
Haus6	0.648032142	0.629578548	2.335922829	profile #4
Smim3	8.022570868	7.969552192	17.28799754	profile #4
Cntln	0.791000742	0.777359491	2.51594522	profile #4
Fkbp14	2.266900544	2.39013901	10.12938943	profile #4
Tmem237	1.338642435	1.117571035	4.278521737	profile #4
Cnbd2	0.254607587	0.182607571	2.18301843	profile #4
AW551984	0.023197793	0.026876335	0.314773858	profile #4
Cdon	0.804655207	0.8358137	2.760612207	profile #4
Ccdc50	3.905714164	3.741526445	8.87509545	profile #4
Camk4	0.030511497	0.032235675	0.088972671	profile #4
Cd84	0.264631894	0.253506449	12.9914553	profile #4
Prdm1	0.656033048	0.312527922	1.38412817	profile #4
Atg5	5.678751282	5.28193116	12.88156728	profile #4
Plekhg6	0.33540896	0.237044317	0.674113492	profile #4
P3h2	0.998479716	1.190912515	2.011362599	profile #4
Slc43a2	1.924834659	1.828541793	7.941877177	profile #4
Slamf7	0.080899433	0.056952094	3.807594742	profile #4
Spag4	0.14975989	0.109707614	0.23016978	profile #4
Chpf2	1.733734726	1.930652003	7.887981047	profile #4
Btbd10	1.72340476	1.92323127	3.449510225	profile #4
Mfsd14b	4.18343735	4.335994588	11.03942496	profile #4
Tlcd2	2.223381552	1.804222329	3.837761955	profile #4
Primpol	0.238979337	0.203976752	1.207437861	profile #4
Cep250	1.472468335	1.628367864	4.222547608	profile #4
Mical2	4.710616318	4.866458208	11.34021262	profile #4
Ncapd2	2.852155657	2.553872928	8.488030729	profile #4
Fau	317.6463253	336.8852191	833.1492883	profile #4
Asic3	0.11645809	0.06454884	0.244560166	profile #4
Ostm1	3.352273536	3.317143288	7.8825501	profile #4
Snx10	1.025058609	0.845336486	4.16525229	profile #4
Edem2	2.679092014	2.78509389	10.26267377	profile #4
1700066M21Ril	0.776178046	0.75743292	2.564442022	profile #4
Satb2	0.050157026	0.064216016	0.181308311	profile #4
Tsr1	2.684257287	2.272078806	5.880431313	profile #4
Zfp384	3.376525863	3.17096038	6.939734051	profile #4
Arl5c	0.328476503	0.262842691	2.372523538	profile #4
Lasp1	8.450132636	8.06193773	36.14998957	profile #4
Gmds	0.94031248	1.166829604	3.261421392	profile #4
Gpr162	1.330637944	1.024183079	4.323532688	profile #4
Upf3a	2.596356258	2.582688249	6.209038581	profile #4
Pmepa1	6.217754546	5.846896934	27.79654554	profile #4
Foxf2	0.072041027	0.046117046	0.196431233	profile #4
Cdc16	10.33652178	9.845469794	25.20758787	profile #4
Poli	0.443316013	0.391375732	1.195669191	profile #4
Spsb2	1.786618596	1.380257041	13.98063031	profile #4
Dennd2a	1.958213751	2.313957392	4.804769172	profile #4
Olfml2b	1.443559792	2.126268843	21.42881334	profile #4

Tfdp1	5.554603672	5.047868815	18.38501458	profile #4
Slc19a3	0.092064584	0.057639748	0.165208484	profile #4
Prr3	1.137844749	0.868210056	2.294941863	profile #4
Parp12	5.3062181	5.455548421	13.11856681	profile #4
Gdf15	0.54981692	0.739287345	13.16272673	profile #4
C1s1	7.751078337	6.747922153	16.2866597	profile #4
Fchsd1	0.338275812	0.342923823	1.476890672	profile #4
Armc10	2.585722054	2.022297407	5.486724727	profile #4
C1rl	0.461308186	0.425524003	1.90657543	profile #4
Osbpl7	0.884233638	0.723673364	1.936087936	profile #4
BC028528	1.419570103	1.189933163	3.294486509	profile #4
Cul7	4.258691073	4.193229722	10.0054156	profile #4
Fndc4	1.233646472	1.147478299	3.013079655	profile #4
Reep2	0.366274599	0.216548065	0.970338707	profile #4
Susd4	0.047988168	0.045792076	0.177224932	profile #4
Susd1	0.116084154	0.185339727	0.707739547	profile #4
Atp6v0a4	0.037228705	0.010956624	0.212130506	profile #4
Gng10	0.070448384	0.039675759	0.242827829	profile #4
Dock10	0.288351768	0.29828264	2.260771922	profile #4
Rassf7	0.240170029	0.278254498	0.999093322	profile #4
Tm6sf1	0.857828596	0.942126718	5.245227536	profile #4
Polr3k	5.217607149	4.62200122	11.74574686	profile #4
Ctss	8.173048164	9.367218701	541.0724349	profile #4
Pold1	3.424628654	3.106190428	7.382842695	profile #4
Creb3l2	4.080008994	4.439083675	10.75789128	profile #4
Rnh1	16.32438154	15.24971952	60.83916007	profile #4
Lpar1	2.374122024	2.159673415	6.552390351	profile #4
Mybpc2	2.448511714	2.064567815	6.016559493	profile #4
Scube3	0.059737442	0.050334679	0.352657149	profile #4
Trps1	0.493013955	0.460169189	2.401755453	profile #4
Rtel1	1.154316358	0.971810793	2.327978404	profile #4
Hoxb4	0.729759323	0.548564521	1.5333566	profile #4
Hoxb5	0.066453106	0.058672602	0.121168702	profile #4
Dsel	1.036042314	1.065267656	4.012485889	profile #4
Bud31	18.46780951	15.90911612	36.42736697	profile #4
Mboat1	0.360564456	0.484436783	1.469993382	profile #4
Nudcd1	1.396459865	1.201344366	4.87956227	profile #4
Nup205	1.50600024	1.433297869	4.408341627	profile #4
Gabpb2	1.59818752	1.374073265	3.283454846	profile #4
Ascc3	0.59755994	0.630003999	1.535344673	profile #4
Vill	0.077220661	0.09015607	3.239801182	profile #4
Lefty1	0.856057493	0.652415715	1.654253391	profile #4
Ost4	29.76477436	29.2228805	78.97832104	profile #4
Gngt2	6.643347583	5.166176925	28.84572273	profile #4
Hace1	0.572659676	0.646120449	1.195332586	profile #4
Abitram	0.632799495	0.467652913	2.233246339	profile #4
Tmem214	4.146073253	4.092308482	8.227858375	profile #4
Gcnt1	0.357249557	0.467747434	4.230124664	profile #4
Garnl3	0.144709031	0.132336358	0.39628012	profile #4
Zfhx3	0.348634864	0.353273809	0.845323045	profile #4
Ctu1	2.00099027	1.408964263	4.160027507	profile #4
Rpl12	44.98303917	42.59972299	149.8357077	profile #4
Ccdc68	0.610466513	0.66235752	1.756037593	profile #4
Prc1	0.095664194	0.066245945	3.995720851	profile #4
Slco4a1	0.017736294	0.010863502	0.254993334	profile #4
Ppp1r9b	22.44618366	19.69808335	55.3467246	profile #4
Txndc5	9.891095131	8.288584843	61.12811411	profile #4
Tlr4	1.199600553	1.407503961	6.399236301	profile #4

Arhgap18	3.377842493	2.434678962	7.870913021	profile #4
Pigk	2.666893203	2.668413879	6.075123691	profile #4
Ak5	0.027631997	0.02594974	0.371888957	profile #4
Anpep	2.283447631	2.292814616	20.05250439	profile #4
Zfp503	1.25171285	0.953308248	2.432639183	profile #4
Chad	0.08265105	0.078868673	0.305480826	profile #4
L3mbtl3	0.630946784	0.713869547	1.393794808	profile #4
F13a1	4.808344667	6.727025547	25.84167092	profile #4
Adgrg6	0.168134632	0.176695903	0.450080123	profile #4
Camk1d	0.53290912	0.454855523	3.126787717	profile #4
Runx2	0.068088678	0.06165813	1.179434093	profile #4
Stim2	3.032693739	3.278281717	6.109602507	profile #4
Akna	1.607084601	1.347434182	5.400291137	profile #4
Dap	6.563996118	5.919452397	50.54319117	profile #4
Nubp2	14.5327133	12.84071097	27.89123837	profile #4
Fanci	0.007370668	0.007649196	0.718390192	profile #4
Rbpj	1.849197117	1.891363563	5.670074693	profile #4
Nlrc4	0.098029512	0.108668987	0.835734411	profile #4
Abhd2	1.885990278	1.896464832	5.199299766	profile #4
Daglb	0.827410062	0.706221798	3.017449361	profile #4
Metrnl	8.638233323	9.466946186	55.29650294	profile #4
Rpl39l	0.111394917	0.125472796	0.720508326	profile #4
Il18	0.182462303	0.264244859	4.907497593	profile #4
Ppp1r10	5.054016902	3.876029867	7.887079378	profile #4
Rpl22l1	10.24906761	10.40582043	32.23137562	profile #4
Tbcd	1.620710811	1.706459471	4.943332217	profile #4
Suv39h1	0.783329977	0.830977519	2.842492432	profile #4
Stx11	0.379902207	0.453089842	3.422188588	profile #4
Sec24d	6.710350546	6.641849878	19.133736	profile #4
Isg20	2.098635299	2.01808826	9.59641459	profile #4
Tgfb2	1.660880943	1.599523273	6.029356822	profile #4
Lyplal1	0.674243297	0.552469352	1.92425818	profile #4
Gimap3	0.330292875	0.317155354	0.829788817	profile #4
Fndc3b	3.288185962	4.288768832	9.144232441	profile #4
Cybc1	2.087478315	2.224498898	10.38649165	profile #4
Cdk5rap2	0.582183075	0.596984173	1.590404442	profile #4
Ndst2	3.382907684	2.720327302	8.382481968	profile #4
Rab3gap2	1.666864279	1.826333234	4.279667033	profile #4
Exosc2	2.426391661	2.676291533	5.571514926	profile #4
Fut11	3.798993701	3.767465479	9.83041521	profile #4
Picalm	8.002420505	9.63883344	23.20405859	profile #4
Prss23	2.159843621	1.688436819	10.68466389	profile #4
Mtmr7	0.162436019	0.159033401	0.322114506	profile #4
Snx22	0.176108775	0.168049473	0.547350632	profile #4
Morc3	2.578561343	2.626755486	8.34520322	profile #4
Zdhhc2	0.452128754	0.394264037	1.855283429	profile #4
Prrx2	0.021702314	0.045044831	3.335305992	profile #4
Asb6	2.621006271	2.265571145	5.632473292	profile #4
Dse	1.001032892	1.109678384	13.031925	profile #4
Cyp7b1	0.534217313	0.305861889	2.707933077	profile #4
Atp8b1	1.453245659	1.269113894	5.903749003	profile #4
Tusc3	5.344279375	5.574929616	10.93070913	profile #4
Zup1	0.614542442	0.534441914	1.54626097	profile #4
Ncam1	0.29963671	0.402491523	2.707933077	profile #4
Ubald1	5.223522482	5.102311144	10.51034968	profile #4
Nphp4	0.070414025	0.069005886	0.335118467	profile #4
Rbms3	0.681981987	0.80163837	2.698369956	profile #4
Mocos	1.460910351	1.554618383	4.389424444	profile #4

Prex1	1.352559556	1.303568714	9.870382538	profile #4
Ap5z1	1.107276311	1.109795115	3.058373566	profile #4
Ccdc151	0.067713981	0.052704602	0.126919557	profile #4
Coro7	1.783775159	1.637556016	5.25081193	profile #4
Vasn	2.92453333	3.171026181	12.21792333	profile #4
Spout1	2.812404827	2.649997956	6.194866979	profile #4
Tbc1d13	4.913248917	4.710916351	13.38574383	profile #4
Ncoa7	0.350474874	0.392962323	1.589626704	profile #4
Batf2	0.568922374	0.367281494	0.953699056	profile #4
Lmbrd2	1.059171703	1.120144338	2.557403548	profile #4
Dock3	0.005891229	0.003608385	0.032560941	profile #4
Slx4	0.792324141	0.88512643	2.563693074	profile #4
Alg2	2.10628415	1.752909512	4.426102771	profile #4
Htatip2	8.75839783	8.319794855	17.53798022	profile #4
Orai2	0.312270546	0.248787964	2.770545192	profile #4
Galnt12	0.141604946	0.27342979	2.821739969	profile #4
Kmo	0.088179782	0.092273141	1.08638397	profile #4
Cercam	0.981242234	1.079115702	10.03761413	profile #4
Cplane1	0.570844745	0.592749797	1.325848746	profile #4
Zc3h10	2.048965505	1.702727808	3.431145826	profile #4
Tbc1d2	0.549138541	0.522274483	1.109098123	profile #4
Xkr5	0.011357078	0.013158003	0.506060967	profile #4
Trim14	0.565487129	0.564844512	2.721018697	profile #4
Lrrc17	0.908255056	0.606880754	11.97608878	profile #4
Tmem120a	7.838950455	7.714618703	21.48254177	profile #4
Alg14	3.977292735	3.375579283	7.673715087	profile #4
Slc26a11	1.21156738	1.041446814	3.601458157	profile #4
Gsap	0.306764199	0.240423533	1.325068867	profile #4
Pik3cd	1.183117626	1.288403722	9.896979785	profile #4
Hip1	4.187348654	4.443566498	8.514361015	profile #4
Rhou	0.815543211	1.202557107	9.301966178	profile #4
Zc3h12d	0.052001756	0.050961825	2.516145669	profile #4
Dtx4	1.461196013	1.474038736	11.79905716	profile #4
Sinhcaf	0.536892273	0.500366606	1.560624615	profile #4
Slc7a5	1.085965253	1.107734759	4.890329509	profile #4
Ptger3	0.024411703	0.018331205	0.111279009	profile #4
Lats1	2.315180845	2.61347834	4.872434821	profile #4
Saa3	0.017760778	0.033895976	20.77963004	profile #4
Stat2	3.396674627	3.035135271	8.710791418	profile #4
Nup43	1.0655157	0.957784392	2.813279642	profile #4
Plcb2	0.348362103	0.421229617	2.213151753	profile #4
Bub1b	0.18914772	0.114710084	7.431773085	profile #4
Bmf	1.41456045	1.16117871	4.209157085	profile #4
Gorab	1.102399995	1.068752446	3.070858039	profile #4
Gpr176	0.043660669	0.013974683	4.886735448	profile #4
Hs2st1	1.764543162	1.613110301	5.102946763	profile #4
Tax1bp3	7.332717299	6.811889128	19.69381581	profile #4
Arntl2	0.454512602	0.384752374	0.946877836	profile #4
Scamp2	6.260855327	5.40161212	15.88915401	profile #4
1700012D01Ril	0.763322917	0.599067621	1.719095431	profile #4
Pclaf	0.061896459	0.067925148	10.92913345	profile #4
Emp3	12.04028551	9.361028323	82.14822192	profile #4
Gas8	1.324614011	1.156510782	3.1447914	profile #4
Gpr34	0.585057953	0.570571452	4.604136848	profile #4
Tm7sf3	6.152430692	5.064787144	12.43634461	profile #4
Lrp1	10.81027404	12.33479869	50.23387021	profile #4
Sema3d	0.224341594	0.247237266	1.011353353	profile #4
Nxph4	0.421161121	0.404621676	1.046841755	profile #4

Klhdc4	4.040048379	3.747103267	7.579072874	profile #4
Bach2	0.335260597	0.330426423	0.727130416	profile #4
Cdk6	0.747753012	0.775106918	2.69340232	profile #4
Pacsin1	0.10452289	0.081354505	0.210072774	profile #4
Cdin1	0.659840068	0.451032981	1.131957522	profile #4
Ddx58	2.281438753	1.866894204	6.405997913	profile #4
Suco	1.72634048	1.86860024	3.808732113	profile #4
Alx4	0.053291398	0.050046977	0.109038482	profile #4
Cchcr1	0.714268333	0.766675687	3.401290077	profile #4
Slc25a24	2.644062737	2.572702115	13.75040258	profile #4
Olfr56	0.056281556	0.060047708	0.301403476	profile #4
Fam102b	4.03323448	4.965047147	10.51594834	profile #4
Arhgap9	1.588855675	1.467574219	10.24292455	profile #4
Ankib1	2.048358853	2.041439264	5.000652209	profile #4
Sec1	0.068920222	0.063320583	0.207776432	profile #4
Plekha4	0.538873814	0.596188242	3.155199077	profile #4
Mterf1a	0.382760588	0.265312706	0.76840311	profile #4
Usp45	1.760024162	1.984539892	3.799741248	profile #4
Os9	24.73558241	24.68876518	74.41053386	profile #4
Blvrb	4.026193493	4.191329989	21.21455627	profile #4
Dgkz	12.02083534	9.837258711	21.82019819	profile #4
Xaf1	2.178284678	1.834527875	4.131468816	profile #4
Tlr8	0.13114796	0.243111165	5.6887924	profile #4
Milr1	0.228114754	0.286311547	5.86732745	profile #4
C3ar1	1.077548855	1.050509886	43.79470324	profile #4
Mettl27	0.305168116	0.374427149	1.060557871	profile #4
Plppr2	1.504949072	1.156358143	2.923729213	profile #4
Btaf1	2.017401532	1.896043037	4.618967833	profile #4
Ranbp17	0.081355001	0.052761219	0.213523559	profile #4
Mis12	2.631029846	2.685354279	8.430985961	profile #4
Bace2	1.870710061	1.895971007	4.201179023	profile #4
Ildr2	0.078534024	0.039622317	1.031474464	profile #4
Apobec1	2.381035431	2.239379936	35.82528451	profile #4
Tmem51	2.973402152	2.760689353	13.79560597	profile #4
Pck2	4.274517834	3.974367025	12.22642082	profile #4
Dhx33	1.088940561	1.047913142	2.54502893	profile #4
Gemin8	0.775318053	0.701082941	1.5648676	profile #4
Dnph1	1.00436719	1.43760619	5.993611139	profile #4
Efhd2	18.90272288	19.47701793	170.0767453	profile #4
Clcf1	0.481402087	0.463350456	2.04563966	profile #4
Mthfd1l	0.786871573	0.667227195	2.528350416	profile #4
Hmgn1	14.42831254	12.65620604	37.33668144	profile #4
Madd	1.146162481	1.133402794	3.060513231	profile #4
Col16a1	1.323797795	1.426412976	25.8704606	profile #4
Limd2	3.333688966	3.050070934	17.98523348	profile #4
Ap1g2	1.130946683	1.200103104	3.939476448	profile #4
Sh3pxd2b	0.537675568	0.622936366	10.91082033	profile #4
Klc3	0.017485677	0.016685477	0.589833401	profile #4
Virma	2.8141652	3.099164988	6.411401153	profile #4
Scamp5	2.497145293	2.461138569	7.697461564	profile #4
Hnrnpul1	10.06664097	10.54401431	20.22073805	profile #4
Hesx1	0.075368042	0.043659687	0.182621204	profile #4
Ints8	1.718294536	1.575114375	3.172212428	profile #4
Cd53	2.672753267	2.564843725	71.0497195	profile #4
Siah1b	1.566560868	1.234256684	3.420282272	profile #4
Lat2	0.264886997	0.39781722	10.97382727	profile #4
Snrnp25	5.873060139	4.607418921	11.59302795	profile #4
Cept1	5.736347721	5.855866928	14.65770761	profile #4

Chil3	0.057634385	0.127779281	27.27988698	profile #4
1700123O20Ril	5.24346312	5.30739086	13.96899615	profile #4
Zmynd15	0.871313405	0.494327874	3.88970142	profile #4
Gpr161	0.49312914	0.422624771	1.40766795	profile #4
Sft2d2	6.713809544	6.049456821	12.34566845	profile #4
Reps2	0.212007515	0.177333272	1.531086395	profile #4
Erf	11.42167476	13.11402142	27.48583283	profile #4
Begain	0.128029923	0.070137295	0.650047674	profile #4
Osbpl10	0.648470585	0.466659184	0.936068892	profile #4
S100pbp	1.376584381	1.271515269	2.742269073	profile #4
Ulk4	0.027654745	0.010388441	0.150100326	profile #4
Arhgef1	8.440412454	7.99128126	16.95868982	profile #4
Rcc2	5.536327374	4.993817278	28.99492013	profile #4
Rps19	39.04305055	38.51908303	105.86394	profile #4
Asgr2	0.088217131	0.099365845	0.231675997	profile #4
Npnt	0.221016735	0.108410843	0.571365281	profile #4
Trim62	0.313988986	0.391135082	1.199772148	profile #4
Iffo2	1.191366487	1.33579374	5.272464526	profile #4
Fzd7	1.746653147	1.829059472	4.847891807	profile #4
Ostc	14.09904935	13.52774227	62.61553323	profile #4
Elmo1	0.849899828	0.733767049	2.532722586	profile #4
Ccdc8	1.482184769	1.302981562	4.236903064	profile #4
Pde9a	1.344178666	1.134350777	2.73143406	profile #4
N4bp2l1	0.401808688	0.464871279	2.119859074	profile #4
Smc1a	8.079143721	8.113118736	17.34322137	profile #4
Ripk2	1.770292543	1.795807113	4.823690065	profile #4
Brca2	0.106060255	0.110068133	0.426825921	profile #4
Hectd2	0.134874379	0.126211459	0.325495784	profile #4
Rpusd1	2.745004926	2.90823543	5.701127285	profile #4
Yeats2	1.691714335	1.737250085	4.62262757	profile #4
Arhgap11a	0.299889045	0.364464385	5.65931191	profile #4
Elovl6	0.172730541	0.178346576	1.4034032	profile #4
Rbbp8	1.065251702	1.253803218	5.477862468	profile #4
Wnk3	0.008038198	0.004170975	0.081788913	profile #4
Sntb2	2.335579176	2.626971753	6.633286911	profile #4
Thoc6	3.403950451	3.146600648	9.31443855	profile #4
Inhba	0.186483667	0.326347084	3.680820383	profile #4
Ankrd42	0.108926271	0.04274351	0.438603847	profile #4
Tmem29	0.128439765	0.205224826	1.10738749	profile #4
Ssr2	23.61575126	22.74201261	104.1495778	profile #4
Pum3	4.93118603	4.595692848	11.25731537	profile #4
Shtn1	0.907269446	0.81758701	6.057218432	profile #4
Mdfic	2.897850199	2.934157765	17.66335297	profile #4
BC055324	0.123119297	0.087979177	1.274720656	profile #4
Meis3	1.444114155	1.043959742	3.419028073	profile #4
Ccnb1	0.020184129	0.037088414	7.080003637	profile #4
Tardbp	11.67207543	9.225657082	23.72904687	profile #4
Serpina3g	0.080870759	0.104908449	5.352303604	profile #4
Piezo2	0.054888384	0.048514576	1.355012721	profile #4
Cep78	1.384310984	1.490704587	2.99751062	profile #4
Kif14	0.004540419	0.010066412	0.80972528	profile #4
Irf8	1.016883223	1.189458086	11.71175815	profile #4
H2-Ob	0.268485206	0.328340789	1.185456612	profile #4
Sox5	0.104684827	0.12329954	0.263169654	profile #4
Ptchd1	0.015402525	0.014697656	0.142320518	profile #4
Fmod	0.502048167	0.148332005	3.309056437	profile #4
Tmtc4	0.635456232	0.599086532	1.431960103	profile #4
Mbp	0.440624374	0.291597703	1.80636559	profile #4

Nppa	155.3252646	174.1073632	974.1897069	profile #4
Ccdc74a	0.241702525	0.21423574	0.691302175	profile #4
D11Wsu47e	2.29027724	2.012964529	4.36291255	profile #4
Fam104a	9.535222858	9.415561967	19.06034799	profile #4
Kctd12b	3.259291458	2.143507326	5.110117985	profile #4
Kif21b	0.151952029	0.134161088	2.129937229	profile #4
Klf8	0.071124991	0.084837604	0.288938596	profile #4
Pnpla3	0.293935366	0.30531671	0.726958336	profile #4
Slc39a11	0.937573543	0.762624143	6.975075244	profile #4
Lrrc29	0.166350485	0.123897324	0.280937394	profile #4
Tmem273	0.664521011	0.353930392	11.03298027	profile #4
Alg13	0.494326338	0.556804422	1.262829937	profile #4
Kirrel	1.947228256	2.423928746	8.831828622	profile #4
Tspo	48.81955987	48.36978253	326.4144744	profile #4
Utp15	1.576503004	1.202131074	2.884459278	profile #4
Ydjc	0.262404502	0.196138552	1.010115379	profile #4
Tram2	0.881704953	0.828825567	4.311187363	profile #4
Cpsf2	3.770918109	4.058979897	8.26237967	profile #4
Abca9	2.703001536	3.100792589	7.49320576	profile #4
Phlda3	10.80666455	11.53701835	63.09589527	profile #4
Efhc1	0.251546674	0.141668333	0.755400955	profile #4
Poldip3	4.564072774	3.782212721	12.11148275	profile #4
Oasl1	0.799598776	0.592724689	6.358673745	profile #4
Ptpre	0.626141863	0.450280513	3.449612672	profile #4
Fhdc1	0.047513183	0.024944472	0.845796991	profile #4
Rhod	1.454242649	1.780214838	5.983667908	profile #4
Tcf20	2.310835269	2.611309703	4.85788807	profile #4
Mcm3	1.813388709	1.481264277	14.77292616	profile #4
Ankrd13a	7.27886742	8.371178682	19.01609595	profile #4
Ipo9	3.758716785	3.910767282	8.365697001	profile #4
Shisa4	2.878572977	2.37454069	8.847758133	profile #4
Gpr45	0.012789331	0.007527948	0.104938658	profile #4
Ammecr1l	2.494262763	2.330762661	6.346532944	profile #4
Slc16a6	2.10045773	2.060588105	4.182541093	profile #4
Rnpep	2.613749129	2.353761389	9.428855311	profile #4
Mvk	3.374016696	2.981866858	6.874746061	profile #4
Mfsd9	0.055059112	0.053158332	0.195670157	profile #4
Pigs	8.920100528	9.193777017	23.18297684	profile #4
S100a10	27.80130369	32.90816516	131.124872	profile #4
Dcaf17	0.570725001	0.536970081	1.155755521	profile #4
Spidr	0.726825638	0.880703374	4.156478644	profile #4
Ncapg2	0.175559685	0.178343313	5.10444179	profile #4
Igsf3	1.204620457	1.246672277	3.909030499	profile #4
Csgalnact2	3.30239429	3.443651655	7.837250888	profile #4
Tbca	59.71796991	51.7482455	114.3637567	profile #4
Myo3b	0.006575449	0.007740771	0.017984231	profile #4
Hnrnpf	22.78167901	23.03236933	51.8354134	profile #4
Arsb	0.662170113	0.76817591	2.691996013	profile #4
Inpp5f	0.892789384	0.944566781	2.017830846	profile #4
Inka1	4.764589942	3.912149704	7.994068401	profile #4
Vwa1	5.94047293	5.583283807	15.00338342	profile #4
Ssh1	1.608138507	1.480162409	2.961193005	profile #4
Rassf4	1.155343601	1.272099828	6.102318679	profile #4
Msantd2	2.333320202	2.294010896	4.834670065	profile #4
Dzip1	0.604899759	0.436449624	1.80300656	profile #4
Tent2	2.738607001	2.428621115	9.120942797	profile #4
Armc5	2.753746598	2.97280615	6.733435505	profile #4
Bend6	0.137473424	0.127418392	1.221135058	profile #4

Cmklr1	4.595327547	5.308034106	15.1718558	profile #4
Slc35f2	0.031338908	0.032523164	0.067131447	profile #4
Zfp451	0.859124242	0.787776335	1.755655232	profile #4
Kdm5b	0.807988278	0.780985731	1.720505903	profile #4
Zfand4	0.58020079	0.390963572	0.867960303	profile #4
Sgsm1	0.102604911	0.124066961	0.240587631	profile #4
Lyn	4.954598667	4.847141224	22.10861741	profile #4
Rabif	4.504063764	4.430539361	9.479706463	profile #4
Cilp	1.382441834	1.201582027	30.61565052	profile #4
Nxt2	2.023392246	1.869998298	4.190811621	profile #4
Stab1	7.414668166	6.885058011	62.70017703	profile #4
Mrtfa	1.482647115	1.458113328	5.673198277	profile #4
Tmem183a	5.025851728	4.900356944	15.4498768	profile #4
S100a13	42.88578649	38.8650599	84.68908457	profile #4
Hps4	2.110239575	2.237409161	4.387349524	profile #4
Specc1	0.269573738	0.242401774	2.604612689	profile #4
Tnfrsf14	1.962294513	1.848978638	5.38309049	profile #4
Ikbke	0.198173323	0.15105148	2.157109908	profile #4
Grap2	0.053905303	0.086268667	0.460446083	profile #4
Gnl3	7.910452956	7.810637349	15.66209098	profile #4
Snx18	6.009345253	6.001952427	22.25846004	profile #4
Rbm45	8.388776752	7.167159487	15.73044011	profile #4
Esm1	1.298868461	1.091304804	2.782321559	profile #4
Dlgap3	0.124829883	0.120520438	0.255411971	profile #4
Rbm7	4.492612591	3.893427714	12.55405596	profile #4
Atf4	33.74694149	36.86515742	76.2923724	profile #4
Agps	3.055105722	2.990120885	7.286580209	profile #4
Nfkbil1	2.968999918	2.028450081	5.168329664	profile #4
Mfap4	4.695886921	4.819375274	114.6395474	profile #4
Tbc1d8b	0.888209327	1.0905009	3.350221201	profile #4
Leo1	1.940852323	2.102584235	5.198090981	profile #4
Clspn	0.026178491	0.043529596	1.845871897	profile #4
Tbc1d10b	6.008541539	5.961654468	15.7070606	profile #4
Pwwp3b	0.300045156	0.278963288	1.69796001	profile #4
Adprhl2	6.431022466	5.613841985	12.28588339	profile #4
Ube2q1	6.177528162	5.865579148	13.65257151	profile #4
Hirip3	2.551677134	2.269608398	5.877527801	profile #4
Shc1	6.24780444	6.196966916	19.00096148	profile #4
Arrdc4	2.980835003	2.54966521	6.130793696	profile #4
Wdr55	7.539011665	6.194821438	13.4928993	profile #4
Dcst1	0.032481005	0.025281315	0.185180501	profile #4
Zc3h12a	2.200204218	2.287634307	5.139190532	profile #4
Npl	0.281365277	0.372901527	5.575205615	profile #4
Mapk6	3.262132118	3.500233059	10.2773468	profile #4
Sipa1l1	1.301144126	1.103644088	2.437930688	profile #4
Commd10	6.02522124	5.176657104	18.0822731	profile #4
Tceal9	14.59504366	16.34093264	113.5697473	profile #4
Map3k9	0.028843265	0.037416517	0.464724673	profile #4
Ttc9	0.275602854	0.185491229	3.481893213	profile #4
Dpm3	20.93179878	17.68580644	40.39393901	profile #4
Sgtb	0.40175419	0.492149888	0.87706409	profile #4
Krtcap2	23.59996843	20.40014399	47.63104242	profile #4
Nmnat2	0.044170459	0.018850472	0.328466531	profile #4
Apobr	0.336015025	0.245113503	7.972974028	profile #4
Trim46	0.256322877	0.172428132	2.003022167	profile #4
Hebp1	2.965491805	2.598827891	6.730291651	profile #4
Exog	0.543362293	0.430957957	1.093686546	profile #4
Gpr153	5.375595538	6.248158258	27.40299135	profile #4

Flt3	0.015229812	0.016882755	0.5602581	profile #4
Snai1	1.160509772	1.142854938	17.1755228	profile #4
Nrep	12.22959269	10.28986771	37.78083992	profile #4
Aida	4.954512174	5.121163255	11.10952063	profile #4
Greb1l	0.043039863	0.0381689	0.230920804	profile #4
Nhlrc3	1.206155292	0.985507947	3.46175187	profile #4
Gng2	0.739190532	0.943287934	8.199098241	profile #4
Klhl6	3.76528498	2.762522051	11.58623362	profile #4
Nemp2	0.33671394	0.274558364	0.977720833	profile #4
Ptgir	1.833789924	1.563179936	3.841815516	profile #4
Edem3	4.265058416	5.11485451	8.536403643	profile #4
Zfp513	5.439747622	5.171451178	13.4036498	profile #4
Dpy19l1	1.683738556	1.662328061	7.652075761	profile #4
Synpo	10.11337459	10.60253044	28.27150331	profile #4
Tuba1c	13.01680877	13.06898192	61.30663085	profile #4
Hic1	9.181230308	7.521190712	20.49086336	profile #4
Lrrn4	0.103102054	0.047554731	0.60121948	profile #4
Mob1a	7.465492307	6.842166979	34.94259025	profile #4
Arl11	0.381466385	0.238708547	14.21660766	profile #4
Simc1	0.314912251	0.355247529	0.758430667	profile #4
Hoxa6	0.052957195	0.059649825	0.156460821	profile #4
Fam124b	0.076064767	0.062119686	0.760383121	profile #4
Uevld	1.06683247	1.049723129	2.360930017	profile #4
Ifi209	0.238661926	0.249035562	8.520167284	profile #4
B3galnt1	1.141138758	0.925454675	8.010771758	profile #4
Filip1l	4.953795643	4.612412905	11.39769911	profile #4
2510009E07Rik	5.100040426	4.986259554	11.28963998	profile #4
Gpr135	0.27917688	0.259463285	0.766939485	profile #4
Rnf227	0.245410304	0.148565689	1.048117778	profile #4
Hilpda	3.794777522	3.35914389	11.13266282	profile #4
Pcdhb12	0.062003505	0.058228684	0.126864153	profile #4
Gm6863	40.38985274	34.95411048	73.318623	profile #4
Tril	1.570724411	1.48333849	3.790729924	profile #4
Lgals2	0.557978999	0.449150409	1.001284753	profile #4
Zc2hc1a	2.520403591	2.43857027	5.112236356	profile #4
Pxylp1	0.706073547	0.611519681	4.612294115	profile #4
Mmp3	1.464864807	1.476308319	10.82837086	profile #4
Vps37d	0.700571338	0.572104363	2.07705442	profile #4
Tmem221	0.175921416	0.101908896	1.709978193	profile #4
Dpy19l3	0.306166511	0.210912356	0.74871961	profile #4
Rpl7	106.387673	102.4359356	236.7402502	profile #4
F830045P16Rik	0.022108175	0.02708257	0.094716369	profile #4
B430306N03Rik	0.033897692	0.05093436	3.338011277	profile #4
Prr33	6.209388937	4.640542956	9.920158002	profile #4
Ly6g5b	0.039357643	0.037556511	0.179409015	profile #4
Clec4a3	1.240420533	0.865356351	26.10514367	profile #4
2900005J15Rik	0.116279336	0.075420862	0.547638665	profile #4
Mgat5b	0.092598427	0.05927686	0.15819068	profile #4
Taf10	6.777380659	7.238821886	14.76890502	profile #4
Zmym1	0.508245589	0.392559725	0.985207185	profile #4
Slc36a4	1.740979916	1.785627532	4.746408853	profile #4
S1pr2	0.661954966	0.464372126	5.038008328	profile #4
Zfp469	0.570673051	0.429913597	3.431240286	profile #4
Naalad2	2.91410651	3.124853548	8.283319402	profile #4
Cep164	0.813938668	0.774029128	2.013602356	profile #4
Mgat2	11.33461548	12.36073561	45.58225242	profile #4
Gls2	0.133231399	0.135062038	0.320077779	profile #4
Adgrd1	0.661923564	0.65286314	3.11875438	profile #4

Pcdhb21	0.162815784	0.103258454	0.32723132	profile #4
Fmn1	0.03054469	0.050789712	0.816698454	profile #4
C130050O18Ri	0.247433332	0.119940575	1.447738206	profile #4
Arf6	22.99143159	27.17260544	88.69819001	profile #4
Bclaf3	0.603105021	0.578195342	1.544685079	profile #4
Hepacam2	0.028980754	0.018552022	0.085360023	profile #4
Vsig4	0.34860259	0.367165026	1.251123999	profile #4
Kcnj4	0.064643279	0.061684996	0.137513502	profile #4
Pcsk9	0.106855231	0.088839443	0.275640729	profile #4
1700001O22Ril	0.091588745	0.078174017	0.168737615	profile #4
Alkbh2	1.412246426	0.935138058	3.433999286	profile #4
Lacc1	1.016127069	0.822393233	6.278955502	profile #4
Pcare	0.009398413	0.00882623	0.051279755	profile #4
Sptssa	23.69019116	21.85952538	57.58655672	profile #4
Gm9493	14.77410297	12.98660795	36.71045917	profile #4
Camsap3	0.098333003	0.110760142	0.357566288	profile #4
Rin3	2.769626408	3.236552989	5.675194624	profile #4
Tent5c	0.307128493	0.265073372	3.432788611	profile #4
Tnfaip8l1	0.972774023	0.679341046	3.851211743	profile #4
Tram1l1	0.059507056	0.056042468	0.618469791	profile #4
Ackr2	0.421116226	0.316771662	0.845465507	profile #4
Dact1	0.996823087	1.268273687	4.914194667	profile #4
9930012K11Ril	0.213777852	0.252645857	1.125035944	profile #4
Cage1	0.114376795	0.097648589	0.241869126	profile #4
Acp1	7.167209957	6.70220654	14.71325869	profile #4
Garem2	0.121680876	0.04570914	0.94348776	profile #4
Tlr7	0.3917218	0.747498342	8.10447042	profile #4
Gm9294	0.234649369	0.276234676	0.867271292	profile #4
Cnrip1	0.705310984	0.748638756	1.933770703	profile #4
Pard6b	0.708090196	0.675685718	2.252962167	profile #4
Zbtb7c	0.499157113	0.530009586	1.38306937	profile #4
Fzd1	2.150463346	2.515078195	10.75206812	profile #4
Palb2	0.086948182	0.118741029	0.515299231	profile #4
Kcnj10	0.026093763	0.030231528	0.177034825	profile #4
Slc38a6	0.416281789	0.419930336	2.082591882	profile #4
Gskip	2.051602764	1.820879494	5.983376003	profile #4
Serpinb1a	1.225948705	1.265734325	37.68502328	profile #4
Macir	0.088769562	0.105483089	0.840386489	profile #4
Scml4	0.231384589	0.195713198	0.710111332	profile #4
A730008H23Ri	3.416759288	3.067403546	10.90000537	profile #4
Fads6	0.332240185	0.335785192	1.092806802	profile #4
Zfp354c	0.397008618	0.419232952	2.443142978	profile #4
Cd300c2	1.034749289	0.858676769	41.09016598	profile #4
Tlr1	0.262823423	0.202552692	6.444311765	profile #4
Lsm11	0.925579828	0.89503374	2.249460423	profile #4
4930503L19Rik	2.230273695	1.629639243	7.584008089	profile #4
H1f10	0.76063415	0.813923572	3.536642591	profile #4
Ubtd2	1.533575821	2.36972485	9.999268812	profile #4
Fbxo48	0.083719908	0.04849781	0.568003145	profile #4
Napepld	1.165042096	1.357380311	2.477296327	profile #4
Fzd5	0.693712967	0.708689948	1.903899364	profile #4
Cetn4	0.158326774	0.087631859	0.680259182	profile #4
Rpsa-ps2	4.260285942	4.024629365	13.17038924	profile #4
Pcdhb7	0.158644689	0.138769216	0.439766741	profile #4
Rpl18a	195.0973344	199.1822553	465.1283727	profile #4
Tubb2b	1.371993019	1.140100224	17.76689267	profile #4
AI467606	0.550701823	0.502930668	5.179892263	profile #4
Vcpip1	1.609444593	1.699060018	3.82462637	profile #4

BC106179	0.022785687	0.01319944	0.077295486	profile #4
Rln3	0.067126557	0.069663181	0.215688892	profile #4
Zfp688	0.928895962	0.823880504	2.222519403	profile #4
Cenph	0.026361916	0.058446129	3.755936719	profile #4
Lca5l	0.180300792	0.159895558	0.419737494	profile #4
Insig1	5.668927774	5.390595117	11.37714157	profile #4
Lhfpl2	0.485512863	0.415089059	13.48398501	profile #4
Tlr9	0.18630944	0.179875607	4.33804645	profile #4
Cenpe	0.043647015	0.020494876	2.770893303	profile #4
Zfp423	0.54471876	0.592675585	1.828817189	profile #4
Nyap1	0.326569366	0.278365848	0.579363849	profile #4
Sh2d5	0.012574775	0.035427639	2.193798245	profile #4
Wdr81	2.122469615	1.85187364	7.476507446	profile #4
Cxcr4	4.189914916	3.70682337	32.93139793	profile #4
Kcnk13	0.226685372	0.29803843	6.332261938	profile #4
Gprin3	0.033302258	0.037074613	0.136653833	profile #4
Zfp956	0.665514035	0.461307332	1.195986252	profile #4
C1ql1	0.031421988	0.029984017	0.174204952	profile #4
Glb1	4.954666669	4.889438207	18.25572914	profile #4
Pigz	0.636447463	0.504064246	1.596544851	profile #4
Pcdhb10	0.04870353	0.036572393	0.159881629	profile #4
Pid1	1.166549812	1.126817424	6.24162267	profile #4
Plekha7	0.218184451	0.181160865	0.433195573	profile #4
Mfsd5	13.33676883	12.85549721	32.50767763	profile #4
Col27a1	0.516372403	0.512451634	3.985740316	profile #4
Pqlc3	0.87297115	0.755648436	5.867630223	profile #4
Tcf21	5.380926505	4.662955	17.83806783	profile #4
Prr15	0.22345419	0.189851184	0.721923542	profile #4
Mms22l	0.046679357	0.076838861	1.485601458	profile #4
Tssc4	8.679928158	8.646605795	21.39524694	profile #4
Zfp764	1.170860585	0.893843584	2.288194595	profile #4
Basp1	1.682052677	1.891161034	81.40114356	profile #4
Ifitm10	0.154636335	0.141520423	0.571086896	profile #4
Zfp36l2	12.11047014	14.18831786	48.43689669	profile #4
Zswim3	0.746778182	0.519799321	1.481254879	profile #4
Npas4	0.028702874	0.026955421	0.090137016	profile #4
Ifit2	4.646678762	4.240069832	16.6982782	profile #4
Mtmr11	0.497668344	0.533341197	2.243808747	profile #4
Tmem104	2.252540088	2.148687608	7.910152264	profile #4
Gapt	0.061352576	0.026183224	1.074695347	profile #4
Pofut1	2.016505737	1.804622177	4.725451005	profile #4
Stard5	1.379635152	1.431769755	4.746702062	profile #4
Snx12	9.985640325	8.824317486	19.72709739	profile #4
Sbsn	0.152622643	0.165340742	0.402381263	profile #4
Ppp1r15b	5.33671063	5.288987914	12.57300956	profile #4
Lrrc8d	0.714166076	0.589379736	1.94657283	profile #4
Il17c	0.064373558	0.075782044	0.25358694	profile #4
Cep295	0.344656322	0.375820696	0.88144028	profile #4
Fut10	0.807779235	0.801740343	1.853506588	profile #4
Tmem229b	1.570305588	1.073360637	5.136405788	profile #4
Adamts6	0.447521855	0.43321518	1.227529239	profile #4
E2f8	0.119049096	0.100105254	1.540902893	profile #4
Cd109	0.394743067	0.339558473	3.725853676	profile #4
Plaur	1.096220546	1.502030821	11.72214592	profile #4
Pilra	0.206593383	0.168718274	4.853256002	profile #4
Usp27x	0.252906744	0.278815603	1.045798933	profile #4
Ermp1	1.44236615	1.400401538	3.475536916	profile #4
Rpl37a	180.046877	162.7267683	336.921519	profile #4

Fam178b	0.02668228	0.013845284	0.128601966	profile #4
Zfp322a	1.973173399	1.687774905	3.564140695	profile #4
Rpl27a	80.68935386	77.52216235	181.7482912	profile #4
Pcdhb17	0.746822903	0.736630743	1.985664644	profile #4
Rbp1	4.061007465	3.597478223	53.10436286	profile #4
Kcnk6	1.699457667	1.557361935	7.150208873	profile #4
Lrrc75a	1.595256265	1.747557884	3.294839692	profile #4
Bex3	5.655159347	4.703938693	27.06159116	profile #4
Hnrnpa1	71.96070983	71.81328842	169.1629159	profile #4
Cmtr2	0.610191003	0.595454555	1.333076559	profile #4
Tmem150b	0.022807201	0.027938878	0.252111721	profile #4
C1qtnf2	4.685310485	3.624154601	11.29101666	profile #4
Golph3l	4.188139247	4.147227017	11.33537087	profile #4
Spin2c	0.03090649	0.039569564	0.459662313	profile #4
Arsj	0.121311378	0.080115366	0.885895033	profile #4
Unc119b	4.404225407	4.60472643	10.0809761	profile #4
Zfp518b	0.797735042	0.757540638	1.578450765	profile #4
Tifa	0.764470848	0.919760442	6.724588856	profile #4
Chtf8	10.20834814	9.673468127	21.19965099	profile #4
Slitrk4	0.136295394	0.106084364	0.498943863	profile #4
Csnk2a2	2.166843844	1.896770879	3.992502383	profile #4
Foxc2	1.018256826	1.264214177	7.443707276	profile #4
Bst2	32.24031656	27.87721807	169.2085694	profile #4
Rpl14-ps1	58.4050544	54.10898235	110.8806513	profile #4
Cdc42se1	7.159825235	7.680876126	32.49609387	profile #4
Kctd11	1.706615451	1.276005689	13.64859561	profile #4
Tmem45a2	0.015334091	0.016998352	0.041833044	profile #4
Fam83h	0.239142892	0.157742653	0.581230364	profile #4
Rhoj	4.48283795	4.502471926	13.68633249	profile #4
Riox1	1.342726956	1.627180158	4.392520512	profile #4
Mpeg1	2.326713298	1.803942793	176.1177225	profile #4
Gltpd2	0.143074461	0.126322929	0.634065335	profile #4
Ddit4l	0.525410332	0.411207399	5.455510673	profile #4
Ckap4	8.913946027	8.776190446	66.84610019	profile #4
Fbl	6.659462216	6.205537491	14.73923629	profile #4
Irgm1	2.834774128	2.485559938	8.097082129	profile #4
Ltb4r1	0.132810869	0.158416273	2.550513407	profile #4
Slc26a1	0.06304924	0.040360955	0.104190124	profile #4
Gm5637	1.293101032	1.420904778	13.32028288	profile #4
Zfp41	0.728335419	0.575423356	1.781607211	profile #4
Pcdhb15	0.082621542	0.110376722	0.341744841	profile #4
Tmem164	4.139378788	5.305647931	10.80331901	profile #4
Tmem198b	1.062817141	1.037511178	3.026649157	profile #4
Fam221a	0.114023858	0.096076332	0.306921096	profile #4
Ticam1	2.887473961	2.892458273	6.718127786	profile #4
Cltc	14.92717446	15.93780475	42.12295688	profile #4
Cd24a	0.681710986	0.909313024	10.6358589	profile #4
Neurl3	2.618525029	1.49646994	21.56414937	profile #4
Samd14	1.614513284	1.385511816	7.239687638	profile #4
Dync2h1	0.231690915	0.266637093	0.479578384	profile #4
Mageh1	6.237266766	6.100780998	14.93546143	profile #4
Ptgs1	2.191649894	2.805353981	5.659732292	profile #4
Prss45	0.037477399	0.042213722	0.110726119	profile #4
Gap43	0.195605816	0.10732887	1.163673023	profile #4
Sfn	1.31922619	1.790344924	10.65868622	profile #4
Zfp286	0.07062394	0.078288995	0.23234489	profile #4
Cstad	0.26786624	0.22167327	0.618019533	profile #4
Abhd17b	3.758869572	3.644110214	7.385245746	profile #4

Tgif1	0.633584583	0.503704724	5.123653551	profile #4
Ctdspl	1.303807258	1.455195399	3.089568425	profile #4
Flrt2	0.694513386	0.531988147	2.302432126	profile #4
Gpr68	0.077582771	0.060888157	0.382027538	profile #4
Adamts12	0.207887145	0.167256807	2.968622792	profile #4
Mis18bp1	0.01177213	0.020458307	1.255080793	profile #4
Lxn	1.565327579	1.481418358	12.18146401	profile #4
Nxpe5	0.019028946	0.034965759	7.888002544	profile #4
Nr1h4	0.204210415	0.165614863	0.494710816	profile #4
Tssk6	0.414424779	0.244886696	0.993098715	profile #4
Msl3l2	1.076847321	1.120299418	3.43737303	profile #4
Rps8	133.8683153	134.4441276	320.4380552	profile #4
Rpsa-ps10	23.01154658	22.5619227	65.43157665	profile #4
Rtl3	0.159530728	0.194529652	0.725361527	profile #4
Champ1	2.01635829	1.94857078	4.177920824	profile #4
Bola2	7.267220535	7.037012424	15.9769667	profile #4
Samd9l	3.803343449	3.03562321	8.038080325	profile #4
Zc3hav1l	0.345383049	0.241758171	1.234403103	profile #4
Fancb	0.570676995	0.458987651	1.390541391	profile #4
Lrrc49	0.593522163	0.650947779	1.589238595	profile #4
Phf13	2.339501796	2.163536263	5.016242472	profile #4
Ccdc88b	0.476006047	0.480941454	7.244312384	profile #4
Tigd4	0.026761832	0.015071971	0.060995953	profile #4
Trim16	1.36786764	1.085451703	3.033494907	profile #4
Angptl8	0.267540971	0.301352293	0.682957231	profile #4
Pygo2	4.870050013	4.622212503	12.49291527	profile #4
Cdca4	1.455351505	1.09416663	5.429043881	profile #4
Diras2	0.049132531	0.018456522	0.38793732	profile #4
A4galt	1.927977709	2.484680652	5.626920152	profile #4
Rell1	2.963746633	3.161292035	8.653062441	profile #4
Klk9	0.144167469	0.105963306	0.285887536	profile #4
Pcdhb16	0.239173948	0.278946043	0.572959252	profile #4
Marcksl1	4.123798184	4.705585121	50.42607266	profile #4
Kcna3	0.146008164	0.137050333	1.054479026	profile #4
Stbd1	5.173671303	4.712944742	15.18028062	profile #4
Palm3	0.128141163	0.129901861	0.307848894	profile #4
Tmem196	0.185265957	0.183111347	0.367386819	profile #4
Zfp473	0.009222793	0.005194176	0.046870626	profile #4
Isg20l2	2.942152569	2.937377571	7.200514814	profile #4
Arxes2	0.241694977	0.272239931	0.977060082	profile #4
Gm4737	2.034487385	2.369012603	4.842739512	profile #4
Entpd1	4.751573762	4.628490214	13.7707415	profile #4
Col6a3	2.139545028	1.853742955	19.27979044	profile #4
Selplg	0.790911733	0.890865662	18.71070058	profile #4
Tmem81	0.991197787	1.033378527	2.013327544	profile #4
Gpr85	0.045343706	0.040034746	0.379573006	profile #4
Rnf149	1.872569207	2.194316566	23.11235421	profile #4
Zfp738	0.373238135	0.296352662	0.672184353	profile #4
Ckap2l	0.029508822	0.03473847	6.233594609	profile #4
Lhfp	9.713601869	9.573539939	29.2251129	profile #4
Pcdhb18	0.183632565	0.148944607	0.351571913	profile #4
F2r	9.448382624	10.97541748	27.84490693	profile #4
Socs4	2.545551327	2.203909003	4.8281507	profile #4
Osr1	1.232164734	1.346799023	3.419772084	profile #4
Fam171b	0.225621251	0.275092515	1.388445867	profile #4
Gli2	0.291197719	0.225043819	0.802948398	profile #4
Cyp4f16	2.07360319	1.824069034	8.391846269	profile #4
Mypop	1.025885937	1.138896011	2.145610186	profile #4

Zbtb8b	0.00655852	0.006806358	0.119417212	profile #4
Tyw5	1.153818077	1.183662636	2.524606857	profile #4
Mmgt2	0.710533836	0.600717365	2.619005328	profile #4
Cxcr6	0.071907698	0.026998419	3.942121261	profile #4
Mlec	18.2732184	19.03018714	54.42249282	profile #4
Igf2	6.016007978	6.482583216	12.9131437	profile #4
Myof	0.776671784	0.748730013	11.91366655	profile #4
Ctxn1	0.840149287	0.731251728	6.754305333	profile #4
Exd1	0.062270319	0.065789161	0.128251952	profile #4
Rhno1	1.029909333	0.998161925	2.801880822	profile #4
Tpcn2	0.817804781	0.798290221	5.828581095	profile #4
Mex3d	4.492411022	3.678284219	9.289541713	profile #4
Ccdc6	3.572625682	3.11905523	8.657489495	profile #4
Lurap1l	1.343551315	1.101830389	2.775830335	profile #4
Rpl29	100.3104447	92.22985954	194.7582688	profile #4
Hoxb3	0.110124819	0.122639254	0.235899683	profile #4
P2ry6	1.872149666	1.759315046	19.21344245	profile #4
Slc35e4	3.082617027	3.28496628	9.005850006	profile #4
Dact2	0.378901682	0.298847108	0.876581777	profile #4
Slc39a9	2.232229975	1.995989348	4.192400174	profile #4
Gm12185	0.053321578	0.048762565	0.163172552	profile #4
Arhgap30	0.624069499	0.491543439	9.423644331	profile #4
Cdca2	0.024687485	0.04767042	1.954094811	profile #4
Ccdc125	1.028595204	0.536651966	2.269144733	profile #4
Mrgpre	0.763000387	0.622393569	1.478264033	profile #4
C1galt1c1	5.078847132	4.93786474	10.5068822	profile #4
Elfn1	0.051004598	0.049984609	0.786654404	profile #4
Atxn7l2	0.541991616	0.579734547	1.106796684	profile #4
Tmem121	0.02940571	0.034617084	1.822996202	profile #4
Clec4a1	1.366553946	1.672542332	17.92494037	profile #4
Armcx3	3.830899516	4.02049928	12.01541704	profile #4
Ccr2	0.273873302	0.333208476	19.21736973	profile #4
C5ar1	4.044184642	3.574561487	46.38038525	profile #4
Purg	1.52399906	1.229951963	4.200491109	profile #4
Rtl5	0.715114252	0.714307325	1.985324107	profile #4
Gm9833	0.953490554	0.651636248	2.813133355	profile #4
Gm7324	0.040299622	0.0756923	0.687012002	profile #4
Scn3b	0.135607656	0.105012528	0.254990114	profile #4
Mblac1	1.138790743	1.114291342	3.548677397	profile #4
Prmt6	0.719951113	0.701051621	1.715986629	profile #4
Fut4	0.24994132	0.216821094	1.946811959	profile #4
Sorl1	0.960770089	1.127471604	3.849185877	profile #4
Smcr8	2.503435647	2.461978311	5.242128312	profile #4
Retreg2	10.60088334	11.03056891	24.82757468	profile #4
Rd3	0.035407234	0.039250101	0.072446046	profile #4
Brd8dc	0.133110976	0.111929519	0.277383733	profile #4
Krt8	0.235779706	0.120747457	3.787998816	profile #4
Ogfr	15.47603879	15.13501011	31.03116854	profile #4
Rarres1	0.780869802	0.566663975	1.307264598	profile #4
Prokr1	0.080488391	0.062647457	0.394308584	profile #4
Zfp260	4.108856345	3.707770674	8.415790646	profile #4
Cyp20a1	4.978642764	4.767041982	13.89800085	profile #4
Tmem67	0.489111969	0.358839331	0.877034557	profile #4
Dtx3l	2.68670481	2.236986918	5.972517351	profile #4
Espnl	0.008637219	0.009574646	0.023563259	profile #4
Spty2d1	1.551951017	1.860873108	4.284248239	profile #4
Rps23	181.4424995	182.0253627	390.5219234	profile #4
Tmem202	0.110398259	0.095928327	0.702749318	profile #4

Sall2	0.61337941	0.521505443	2.003833257	profile #4
Ap5b1	1.079917773	0.771341743	5.076741307	profile #4
Tsku	0.880165936	0.717910086	9.212500444	profile #4
Vsig8	0.049265128	0.027745581	0.546256293	profile #4
Zbtb45	1.820863263	1.46871828	3.183178673	profile #4
Slc17a5	1.750483567	1.558672863	5.353746769	profile #4
Tifab	0.966192429	0.669380101	11.43478413	profile #4
Zbtb5	1.048645614	1.176054597	2.311540605	profile #4
Aftph	3.086342398	2.995516926	6.233716483	profile #4
Catsperg1	0.060089001	0.066610664	0.16824795	profile #4
Pheta2	0.154243082	0.213428967	1.720505903	profile #4
Gal3st1	0.041703319	0.025543335	0.125724593	profile #4
Mmp12	0.041164524	0.043222838	6.670112659	profile #4
Zfp668	0.57590169	0.521291907	1.271030644	profile #4
Arhgap15	0.096525827	0.09015607	1.052450384	profile #4
Rpl36al	0.102626581	0.065286706	0.808554367	profile #4
Tmsb4x	234.6337448	231.3515184	1336.33044	profile #4
Armcx4	1.285039837	1.34222724	3.665506292	profile #4
Fam161a	0.320948534	0.332063355	0.673986899	profile #4
Arl4c	1.635590818	1.600350494	17.80233972	profile #4
Nlrc3	0.153039353	0.137473408	1.408954736	profile #4
Calhm5	0.37389391	0.315296893	1.315931252	profile #4
Vcpkmt	2.518628255	2.752340056	9.943207477	profile #4
Rasl11b	13.47200641	14.12263844	27.37888183	profile #4
Slc35c1	2.326083788	2.129579573	7.042631977	profile #4
Lpar4	0.58814816	0.427282853	1.32293937	profile #4
H2ax	7.323934085	7.116416095	40.45911809	profile #4
Lrrc4	0.046330919	0.052186133	0.235455692	profile #4
Rpp38	2.662907727	1.907380574	4.333089285	profile #4
Plekhf2	3.249828327	3.429840018	8.164473903	profile #4
Ankrd55	0.03679942	0.013823617	0.198107983	profile #4
Lrrc25	1.536417859	0.865293721	24.58793699	profile #4
Amz1	0.133451243	0.146449338	1.45550352	profile #4
Rap2c	7.63527184	7.331576097	15.00549748	profile #4
Rspry1	0.95404954	0.986563218	2.37000789	profile #4
1600012H06Rik	4.116995837	3.754892167	11.33450714	profile #4
Grrp1	8.41000952	7.891648403	29.22349121	profile #4
Haspin	0.131771069	0.096952473	2.368330553	profile #4
Vwa3b	0.026195502	0.008200233	0.11002029	profile #4
F2rl3	0.554643689	0.339719953	1.629231071	profile #4
Eif5a2	0.65371111	0.571080106	1.650282463	profile #4
Eva1b	9.46712582	9.202796639	26.33867635	profile #4
Snip1	2.944196084	2.939800235	7.473109613	profile #4
Pigm	1.525663544	1.521951041	3.351628302	profile #4
Cxcr3	0.053477283	0.06023564	0.963561704	profile #4
Heatr1	1.430872547	1.198787737	4.145958575	profile #4
Evc2	1.501969642	1.393334879	3.006538835	profile #4
Fzd2	1.99006199	1.603641485	14.92965122	profile #4
Lgals3	1.511756532	1.521341893	370.7267951	profile #4
Gpr18	0.106076214	0.101221826	0.522747937	profile #4
Carmil2	0.069805747	0.0665095	0.138426464	profile #4
Ch25h	0.3428488	0.654317879	6.752926285	profile #4
Septin6	0.618251935	0.575045557	1.831867349	profile #4
Kif7	0.414247568	0.398316554	1.197469994	profile #4
C77080	1.039984645	0.939675989	2.88345271	profile #4
Armcx6	0.321602653	0.419420327	1.906900343	profile #4
Tcf19	1.45153267	1.140099335	13.04542578	profile #4
Fbxo46	1.115856153	1.247379205	2.408694031	profile #4

Cyp8b1	0.043649474	0.0558844	0.187004113	profile #4
Fam167b	0.482633869	0.322382897	13.11856274	profile #4
B230217C12Ril	0.494801832	0.354118585	1.560067273	profile #4
Fam241a	1.931019091	1.813457168	4.078472482	profile #4
Hyls1	0.27088995	0.27944494	1.053157965	profile #4
Prokr2	0.011877676	0.0065834	0.074828802	profile #4
Tor1aip2	6.823687905	6.221234691	13.74687153	profile #4
Mmp13	0.159215252	0.253550807	1.423977213	profile #4
Zfp61	0.57693046	0.46780773	1.081368392	profile #4
Zscan29	1.148024862	1.324562452	2.784095572	profile #4
Ism2	0.017801765	0.02095665	0.081148078	profile #4
Prkaa1	3.894695794	3.698814951	8.450587291	profile #4
Ftl1	95.88434853	81.24298891	1486.483376	profile #4
Zbtb26	0.438993879	0.307672148	0.653520999	profile #4
Plekho2	2.823405657	2.71110366	17.6675397	profile #4
Vamp8	21.98811548	21.7694758	50.87293406	profile #4
Ptges	0.32967908	0.247179357	1.043602828	profile #4
Tmem37	1.460809047	1.583389376	8.318585416	profile #4
B3galt6	1.133856113	0.884181257	2.705730436	profile #4
Zfp940	0.129201641	0.101532085	0.242511496	profile #4
Pdik1l	0.812569359	0.722120709	2.031631516	profile #4
Rtn4rl2	4.493200311	5.572129349	12.38451138	profile #4
Tvp23a	0.116556505	0.105029356	0.665769489	profile #4
Cdr2l	1.766789873	1.408551867	9.325882455	profile #4
Tmem123	22.72066487	23.25522349	56.46100163	profile #4
Sgms2	0.193255065	0.234422757	1.270588307	profile #4
Insl6	0.196121136	0.230878347	1.026898821	profile #4
Selenon	5.102942972	4.637216158	18.80472585	profile #4
Adgb	0.007595229	0.007132826	0.161952447	profile #4
Gatd1	2.498410154	2.204688029	4.878265934	profile #4
Olfml1	2.601482731	2.123135314	6.611280763	profile #4
4930579K19Rik	0.100294352	0.081907222	0.572911264	profile #4
Vtcn1	0.016231212	0.016844568	0.034769071	profile #4
Gimap9	1.861709989	1.640775802	3.542238077	profile #4
Camk2n2	1.821443009	1.772630654	7.288869924	profile #4
Nat2	0.727013245	0.357997962	3.683716851	profile #4
Gpr183	0.519167711	0.336691022	3.17619366	profile #4
Ercc6l	0.097571906	0.088495949	1.487312273	profile #4
Bzw1	19.01943293	19.85464574	53.24522469	profile #4
Gen1	0.065584242	0.047009871	0.918625925	profile #4
Zgrf1	0.092104269	0.092937669	0.587433557	profile #4
Gdf6	0.301400205	0.354815308	6.401131711	profile #4
Ffar2	0.061015393	0.015830271	0.50584997	profile #4
Nup160	0.912571846	1.094596851	2.157647928	profile #4
Plekhm3	0.697628851	0.68346591	2.015953477	profile #4
Kif18b	0.0238288	0.02919034	3.130516541	profile #4
Vamp7	5.206414144	5.002278261	11.29662767	profile #4
Plagl2	0.861048674	0.779068486	2.567230607	profile #4
Fhad1	0.007658306	0.008489488	0.213012052	profile #4
Cd14	4.060900468	7.382819809	39.31904217	profile #4
Bbs12	0.611468921	0.362614588	1.645613724	profile #4
Crebzf	8.338766215	6.983876395	16.21961203	profile #4
Spn	0.217530642	0.244442018	2.044920532	profile #4
Tlr6	0.086931075	0.073098055	2.054686989	profile #4
Siglech	0.062900143	0.039166234	0.296426117	profile #4
Wdfy4	0.295543312	0.305817171	4.080374765	profile #4
Arhgef39	0.063638485	0.097446629	1.87057044	profile #4
Pusl1	2.663003346	1.972224779	5.472159747	profile #4

Tceal8	5.90852518	5.858991543	25.10746215	profile #4
Map3k19	0.00947872	0.011158568	0.03503364	profile #4
Pcdhb2	0.033437368	0.037066439	0.076017269	profile #4
Rap2a	3.830877789	3.984362482	16.70839631	profile #4
B3gnt2	0.457342247	0.421888543	0.982497321	profile #4
Treml4	0.04262217	0.026106133	1.597775498	profile #4
Tmem198	0.305929818	0.353944526	1.058935131	profile #4
Apon	0.026113081	0.028947222	0.227784715	profile #4
Kctd14	0.066290089	0.017198776	0.36638666	profile #4
Rinl	0.791844497	0.670296668	5.717088068	profile #4
Nlgn2	2.596898466	2.328568597	9.292742562	profile #4
Arf3	9.746663308	9.530838223	35.21130992	profile #4
Mest	0.915132539	0.740050955	8.920025062	profile #4
Spats2	2.124440537	2.29117998	6.736952805	profile #4
B3glct	1.485582858	1.635300071	3.832835705	profile #4
Xkr4	0.020950888	0.02566489	0.15707701	profile #4
Slc6a7	0.027698736	0.046798875	0.277777979	profile #4
Zfp217	2.68403866	2.201488157	5.258300252	profile #4
Dock8	1.585716638	1.889104599	8.025044441	profile #4
Rgs14	0.166417758	0.172180999	2.150613466	profile #4
Gnpda1	5.017317227	4.511591542	24.02979606	profile #4
F730043M19Ri	0.11078066	0.106374319	0.955538722	profile #4
Rasal3	0.103967831	0.178026677	1.921341522	profile #4
Rps10	74.684166	71.16822062	161.4427526	profile #4
Plpp2	0.671602244	1.07167726	7.806700181	profile #4
Pld4	1.991459959	1.83718703	28.12858973	profile #4
Zeb2os	0.275953986	0.375604803	1.571421539	profile #4
Fpr2	0.10714025	0.126127986	1.266173678	profile #4
Slc39a1	23.65408797	24.50119069	67.98774198	profile #4
Ankrd44	0.747583844	0.764999006	1.914121426	profile #4
Cx3cr1	1.40932384	0.813721624	17.11296772	profile #4
B630019K06Ril	0.364147569	0.403669738	0.833157962	profile #4
Tmem106c	6.975906722	6.965041945	16.58803681	profile #4
Nrros	1.844156303	1.797291914	16.55608076	profile #4
Rexo4	3.501046117	3.823256914	7.789677638	profile #4
Ccdc171	0.117593745	0.139883761	0.29633403	profile #4
B4galt3	1.496828348	1.43801524	3.62196216	profile #4
Atp6v1a	4.158261299	3.975660958	31.19262012	profile #4
C130026I21Rik	0.011120522	0.011540752	0.055130651	profile #4
Tmem171	0.861566707	1.067028418	2.350446278	profile #4
Gan	0.704931058	0.586842236	1.99081664	profile #4
Cpne8	1.788590648	1.426300335	3.393491415	profile #4
Hook2	0.815748224	0.710571452	1.96742553	profile #4
Dlg2	0.080523418	0.103094093	0.530137142	profile #4
Serp2	0.606613211	0.478293089	1.064979026	profile #4
Adam17	4.852312134	4.569803455	15.89708132	profile #4
Sh2d6	0.024501	0.025426861	0.098485471	profile #4
Rap1b	21.36229287	22.66850526	86.75484866	profile #4
Rab7b	1.098443581	0.98982101	15.74177577	profile #4
Map1b	0.654281693	0.549436394	2.38180645	profile #4
Traf7	6.896971136	6.124252607	12.43252857	profile #4
A630001G21Ri	0.683419535	0.342375634	4.720349711	profile #4
Oas1a	1.746459467	1.101232611	16.38631525	profile #4
Nup107	2.284297699	2.317305008	6.428873523	profile #4
Atad2b	0.715406497	0.807753337	1.806847069	profile #4
Cysltr1	0.256093045	0.273748845	2.994211128	profile #4
Sae1	11.95878265	10.67973032	25.99434572	profile #4
Nrk	0.080407536	0.081523319	0.320715529	profile #4

Dnah6	0.025701358	0.015742114	0.148508834	profile #4
Prkcb	0.177998115	0.202042346	2.239182085	profile #4
Ubxn8	4.631620317	3.870681793	8.624510875	profile #4
Glis3	0.099457948	0.098597222	0.804210742	profile #4
Uba2	5.439378899	5.418197345	12.40800242	profile #4
Hrh1	0.105684626	0.089325418	0.297052881	profile #4
Creb5	0.268783868	0.245865701	0.599949154	profile #4
Sv2b	0.050779426	0.042158651	0.114213972	profile #4
Aph1c	0.277998089	0.246076321	1.451155247	profile #4
Cd8b1	0.062420213	0.059563664	0.346060544	profile #4
Clec12a	0.43112541	0.330081066	10.39784229	profile #4
Cfap300	0.324953671	0.410708215	1.231178928	profile #4
Rnf26	0.16364057	0.164614169	1.010159061	profile #4
Mapk11	2.086600279	1.900605725	5.361519035	profile #4
Fes	2.919012251	3.114173051	14.68282692	profile #4
Rpl18-ps2	0.816306667	0.827522964	2.250975428	profile #4
Bcl3	1.558154342	1.159927149	13.77582879	profile #4
Mterf1b	0.473672684	0.322854217	0.801989772	profile #4
Mllt11	4.851633963	4.262523945	8.772433485	profile #4
Prx	0.335644815	0.341827414	0.847116694	profile #4
Ddx10	3.26533787	3.247533591	6.746989409	profile #4
Rab4b	0.259237991	0.134517127	0.694697512	profile #4
Sec61b	7.881445524	6.850015028	60.45691392	profile #4
Slamf8	0.082932582	0.07172208	1.036296742	profile #4
Gas5	6.499537891	6.347189617	20.20073205	profile #4
Phgdh	0.205429945	0.146797115	5.363737773	profile #4
Adamts19	0.100330177	0.09465593	0.488451055	profile #4
Ggcx	4.579948444	4.220592361	8.613415763	profile #4
Lgals7	0.229548203	0.056239425	2.65527684	profile #4
3110082I17Rik	2.422520898	2.681870073	6.572692204	profile #4
Tanc2	0.540753392	0.571863187	1.262028585	profile #4
Zfand2a	5.728590631	5.860669677	11.61297185	profile #4
Zfp472	1.253820111	1.00378311	2.237719714	profile #4
Sh3pxd2a	7.415800509	6.560969536	14.81510326	profile #4
Tll1	0.093257286	0.062623231	0.264608295	profile #4
Dennd4a	0.387133522	0.435426879	1.525525122	profile #4
BC048403	0.575605527	0.581852084	1.759242256	profile #4
Dpep2	0.159359037	0.120016057	3.108637478	profile #4
Tmem39b	1.599511134	1.333108277	3.62903322	profile #4
Ptrh1	2.365203616	2.451530203	10.88351578	profile #4
Gm9920	0.095288524	0.060998925	0.295249093	profile #4
Ppfia2	0.042451241	0.039979668	0.086295602	profile #4
Txlna	5.833173582	5.919430705	15.04350702	profile #4
Slc39a8	0.155484164	0.127971192	1.00206864	profile #4
Cnn3	14.92018823	14.25193536	57.09169389	profile #4
Tusc1	2.911553325	3.33110069	8.199853775	profile #4
Pkp3	0.039017969	0.036642525	0.45380561	profile #4
Utp18	2.680311369	2.362477985	5.981324199	profile #4
Skp2	0.129302976	0.089947272	1.800405347	profile #4
Syne3	0.847686707	1.154314391	2.713427378	profile #4
Cthrc1	0.062546741	0.059684401	122.6716969	profile #4
Tprkb	2.471968659	1.762137134	3.561946361	profile #4
Msi1	0.081085618	0.071908892	0.421970003	profile #4
Arfgap3	3.465156322	3.325580252	15.51328759	profile #4
Prr14l	0.628448619	0.665245704	1.84576381	profile #4
P2ry10b	0.257882687	0.240733953	1.183954608	profile #4
Kcnn4	0.099843371	0.122308379	5.596413396	profile #4
Zfp747	1.205615776	0.688145928	2.241342085	profile #4

Cklf	0.234294943	0.221339421	1.564317227	profile #4
Spcs3	12.23216463	11.20594065	38.58578436	profile #4
Slc30a7	0.878557761	0.834818506	3.101380826	profile #4
Tmem62	1.188911162	1.253142568	2.423429191	profile #4
Sh3bp2	0.602102239	0.583430012	10.11443032	profile #4
Adam12	0.026265727	0.033418297	6.121895246	profile #4
Pla2r1	0.481228821	0.406534207	0.92571918	profile #4
Pabpc1l	0.152922648	0.113496769	0.392504732	profile #4
Gm9949	0.192451509	0.162579875	0.789548863	profile #4
Cggbp1	6.601848577	6.687405102	20.73457203	profile #4
Xlr	0.26205982	0.378623719	1.07459232	profile #4
Tmem119	2.463414145	1.671100467	29.34650995	profile #4
Adam10	10.14684266	9.560896801	30.65413434	profile #4
Ap1s3	0.016082206	0.018932345	0.454519461	profile #4
Hmgb2	3.822370239	4.01513244	32.93822436	profile #4
Vmac	3.016841011	2.226413865	4.542295729	profile #4
Zfp182	0.556753492	0.508457379	1.230875122	profile #4
Set	22.61917581	22.62126533	48.75173091	profile #4
Actn4	32.35351895	30.5928583	71.74187528	profile #4
Nsd3	1.874009283	1.811513482	3.847977949	profile #4
Elp6	0.638633942	0.522861954	2.079134773	profile #4
Smim10l2a	0.077366633	0.108930102	0.38793732	profile #4
Tmem158	1.392091861	1.573688007	7.961233122	profile #4
Pcnx3	3.292828404	3.127007336	6.837300657	profile #4
Klhl5	3.567920629	3.165621205	7.288953294	profile #4
Zkscan4	0.195058586	0.173779822	0.409853441	profile #4
9130008F23Rik	0.304963047	0.245482854	0.996779535	profile #4
Naaladl1	0.055742177	0.070921649	0.206024804	profile #4
Lrtm2	0.034395167	0.03984931	0.077785414	profile #4
Pdlim1	10.43603068	9.198614714	19.77541826	profile #4
Rab39	0.031157582	0.035095218	0.326668128	profile #4
Rps27a-ps3	14.09572178	11.68411099	27.5127317	profile #4
Arntl	0.130112914	0.159388645	4.212408247	profile #4
Sugct	0.446805637	0.309126557	0.773278964	profile #4
C1ra	4.133435266	4.179809884	10.29079365	profile #4
Fam72a	0.699268155	0.463636927	1.23259152	profile #4
Sertad3	4.069534031	3.074903491	9.203500575	profile #4
Zfp811	0.068001241	0.031513759	0.185514672	profile #4
Foxd2	0.426895895	0.336341295	0.756187058	profile #4
Zfp101	0.653507409	0.464033356	1.264809267	profile #4
Them7	0.029745879	0.028384612	0.058589013	profile #4
Sec23ip	2.504678502	2.469394353	5.429748492	profile #4
Stam2	2.508009966	2.638375195	5.683553698	profile #4
Map6	0.849629675	0.803200009	1.63555075	profile #4
Pcdh9	0.163130497	0.11720422	0.638406897	profile #4
Maf	2.261923039	2.439729397	18.95562374	profile #4
Cd47	11.23434224	11.75930867	25.31660092	profile #4
Zfp458	0.11029532	0.096508602	0.232230271	profile #4
Soga1	2.868667797	2.811718865	5.661955486	profile #4
Pprc1	1.502586412	1.408088236	4.160188563	profile #4
Lair1	0.289413283	0.366330478	4.854771944	profile #4
Cdca7	0.505063399	0.386215113	4.085604761	profile #4
B4galnt4	0.033145044	0.022931701	0.088821009	profile #4
Klhl25	1.744097813	1.653270984	4.031003132	profile #4
Kbtbd11	0.393560829	0.452894031	1.334831268	profile #4
Rras2	10.44074298	9.536042288	19.65082341	profile #4
Gemin6	2.470045089	1.855733481	4.947653449	profile #4
Fmnl1	5.775234364	5.279003437	14.41354482	profile #4

Mta3	2.758648891	2.806591918	5.775411964	profile #4
Izumo4	1.17616152	0.915203464	2.093291261	profile #4
Fancm	0.211723558	0.228447191	0.655437787	profile #4
Zfp709	0.502579956	0.334275997	0.974224656	profile #4
Clca3a1	0.141897174	0.293468348	4.458877695	profile #4
Rgs9bp	0.014273037	0.013404084	0.041501292	profile #4
Otulinl	0.706797574	0.53423479	21.21778497	profile #4
H2-T22	6.006130963	5.45830496	16.86303011	profile #4
B4galt6	3.70563592	3.672933652	11.52280346	profile #4
Ticam2	0.059864637	0.079634338	1.525451641	profile #4
Pgm3	0.730328558	0.848178763	3.240637068	profile #4
Trim34a	1.535676917	1.327484754	3.516416694	profile #4
Col8a2	0.082485343	0.097103658	4.718844768	profile #4
Cfl1	81.16515688	72.33338182	559.2055513	profile #4
Npm3	18.35704911	17.12909791	35.7899404	profile #4
Cebpg	5.401652684	5.166647482	10.56819096	profile #4
Pla2g4a	0.456241406	0.528588949	3.887937783	profile #4
Ncoa4	5.343728265	6.547776297	11.94844757	profile #4
Lman1l	0.101028456	0.052423094	1.109123056	profile #4
Ms4a4b	0.053503593	0.079080693	0.472055938	profile #4
Tyw1	1.359467232	1.136107934	2.344512171	profile #4
Lig1	0.86751795	0.900300315	8.33040227	profile #4
Adap1	0.85764273	0.818394249	8.254820234	profile #4
Slit3	1.790646302	2.234894058	5.138845077	profile #4
Mok	0.12498748	0.129710593	0.260245817	profile #4
Cd248	5.635641528	4.107830837	24.67473639	profile #4
Tmem154	0.44911605	0.428800552	1.665436695	profile #4
Rab31	4.113065646	5.313921537	29.00133308	profile #4
Ptafr	0.633613766	0.659379035	18.25017364	profile #4
Ccdc18	0.036553442	0.013731216	0.28586889	profile #4
Pign	0.563477944	0.571383454	1.994956984	profile #4
Rlim	2.33117742	2.149629053	7.391716609	profile #4
Zfp658	0.304422295	0.265031167	1.235908119	profile #4
Ppp1r14b	58.12014642	61.38077295	164.6096259	profile #4
Fkbp2	26.3186873	24.7811794	75.0846989	profile #4
Capg	3.923787245	3.911936074	87.1804376	profile #4
Cspp1	0.28400523	0.288098492	0.745559083	profile #4
Ttc26	0.301923765	0.256520517	0.942656432	profile #4
Gm6851	1.523290238	0.965597542	3.311663317	profile #4
Gulp1	1.732679682	1.469081188	3.772802584	profile #4
Glipr1	0.487669822	0.565001061	23.51874289	profile #4
Sipa1	15.38830925	14.78685667	35.81265131	profile #4
Cep162	0.432658909	0.417718363	1.087816311	profile #4
Commd7	3.176641121	2.856784627	7.858643971	profile #4
Mab21l1	0.037146539	0.021864884	0.067731943	profile #4
Gm7536	33.47155875	38.74820363	87.75005523	profile #4
Ero1b	0.629135669	0.673909427	2.410169867	profile #4
Zfp607b	0.115899338	0.120279022	0.248466612	profile #4
Cntrl	1.307628149	1.13080687	3.191474127	profile #4
Npm1	35.04776683	33.47701419	124.5959458	profile #4
Gja5	2.029821669	1.823690095	3.704648778	profile #4
Rpgrip1	0.011426412	0.008580307	0.021116186	profile #4
Scimp	0.197028875	0.32715894	3.038816828	profile #4
Trim12c	4.244816494	3.530954705	10.7947449	profile #4
AB124611	0.676461264	0.866073025	25.34152957	profile #4
Armc7	0.721091013	0.475643698	4.056081817	profile #4
Snrpg	12.79457823	11.75504276	30.86237855	profile #4
Cep170	0.966773211	0.911693808	2.932073526	profile #4

Sphk2	4.692788718	5.594150702	10.98232582	profile #4
Apol9a	0.089694199	0.112311397	2.57903399	profile #4
Uxs1	2.389774682	2.330935234	7.127536822	profile #4
Yipf1	1.617197016	1.494122942	4.149402804	profile #4
Nsd2	0.999899152	1.089551234	3.40449484	profile #4
Zfp53	1.199406094	1.437877847	2.591410852	profile #4
Bloc1s2	14.1946008	15.08019526	29.41294328	profile #4
Lgals8	10.41844658	9.654958135	20.53785672	profile #4
Eif1a	4.819029257	5.486830793	29.25877127	profile #4
Zbtb8os	2.508248453	2.432917513	5.813808934	profile #4
Arl16	2.315464587	1.884564351	4.133238015	profile #4
Trim30d	0.770079859	0.679766735	4.659860334	profile #4
Gm6807	2.189135919	1.392636026	4.773816977	profile #4
Gnai1	1.696899398	1.451082952	3.169674969	profile #4
Bloc1s3	1.578473482	1.572406106	4.010568099	profile #4
Mex3b	1.076064937	0.631889695	5.408259356	profile #4
Tmem178b	0.029003798	0.021434452	0.051537134	profile #4
Prtn3	0.026723203	0.062918349	1.364337193	profile #4
Megf6	0.611095233	0.549335734	2.372644017	profile #4
Gm6169	1.322197651	1.467848985	5.222044452	profile #4
Bak1	11.42212101	9.504871061	37.11724751	profile #4
Rpl32	185.479668	180.3304013	444.3270952	profile #4
Zfp595	0.432257891	0.350561608	0.954086588	profile #4
Zfp105	0.879137288	0.665103069	1.669209244	profile #4
Unc13d	0.197839658	0.211700147	1.938951714	profile #4
Septin11	2.810260421	2.796035149	18.39266984	profile #4
Gm10036	3.678297313	3.551313119	11.0796593	profile #4
Zfp729b	0.594853054	0.544704504	1.35378553	profile #4
Nfam1	0.732623833	0.930105817	12.37093052	profile #4
Tpm3-rs7	3.428488001	3.439363211	15.67025202	profile #4
Chsy3	0.101640279	0.13013	1.415211522	profile #4
Gm5431	0.143815395	0.197519953	1.77041374	profile #4
Idi1	0.558538915	0.60484734	1.625615279	profile #4
Espl1	0.160564936	0.123274411	3.073192584	profile #4
Mcm9	0.469625691	0.383295778	1.088148258	profile #4
Ube2e2	1.524297488	1.717796506	3.583280278	profile #4
Zfp85	0.558215531	0.485050058	1.033377274	profile #4
H2bc8	0.599153243	0.428800552	1.660866626	profile #4
Phtf1	1.1231659	1.00100039	3.042997281	profile #4
Rpl10-ps3	1.163494156	1.561303091	4.844734971	profile #4
Dhcr7	1.153733069	1.129255163	2.495404523	profile #4
Wdr91	2.042130485	2.062047763	6.91337518	profile #4
Rpl23a	153.4508329	135.4784326	308.1064461	profile #4
Rpl5	68.82057748	67.28902301	143.4913302	profile #4
Tmed9	33.75450617	31.02242325	76.84963639	profile #4
Gpc6	1.40171129	1.644296388	5.478269877	profile #4
Anks1b	0.035218181	0.021320267	0.061108683	profile #4
Rpl30	101.8720717	86.81882079	208.6535193	profile #4
Rpl28-ps1	28.06376492	26.03047215	56.50657401	profile #4
Gda	3.119978975	2.532341666	14.54768545	profile #4
Gm17383	0.533539919	0.78511945	3.081218704	profile #4
Tubb2a	16.53021	15.63548318	93.03309407	profile #4
Fcer1g	7.7317504	7.336928427	188.5872247	profile #4
Lin9	0.337595561	0.406172509	1.182973972	profile #4
Kcnj14	0.407255856	0.360975789	1.463879408	profile #4
H1f5	0.114557838	0.059443414	0.283963663	profile #4
Nap1l1	12.01584182	13.23705335	38.84253314	profile #4
Pirb	0.797117709	0.645901669	13.17168275	profile #4

Abi1	6.491397742	6.70280153	24.19296136	profile #4
Rps27a-ps2	5.236707157	4.242162687	16.44508364	profile #4
Zfp516	2.041619399	1.947436669	4.612107849	profile #4
Rsl1	1.333040207	1.083661024	2.327472958	profile #4
Gm10051	0.822381389	0.960140366	2.642449416	profile #4
Kcp	0.604717302	0.673217416	1.353084021	profile #4
Eno1b	0.803010674	1.005497025	4.488178525	profile #4
Frem1	0.012178634	0.011694224	0.208298256	profile #4
Rad51b	0.075107253	0.087017232	0.449262713	profile #4
Rpl18	89.173723	90.04485527	189.3165094	profile #4
Fcgr4	0.336995961	0.373571274	21.86036355	profile #4
Ifitm6	0.255715956	0.244013574	4.914697042	profile #4
Pde1a	0.962183632	0.962376222	4.976326219	profile #4
Skap2	0.83234669	0.681271036	6.695341603	profile #4
Hnrnpm	25.72678759	24.70845209	53.43517186	profile #4
Septin9	7.138982502	7.187799923	23.04118087	profile #4
Rpl11	101.1803541	92.28307808	207.3350581	profile #4
Csf2ra	13.0918548	12.30163617	91.03956422	profile #4
Actg2	0.755132603	0.502253822	5.328175941	profile #4
Ptk2b	0.750763796	0.876451405	7.312321495	profile #4
Zfp426	1.49610575	1.482571512	3.132303394	profile #4
B3gnt8	0.682852626	0.652278295	3.659730128	profile #4
Nhs	0.138872359	0.105199765	0.383391258	profile #4
Fcgr3	5.391072605	5.712377163	77.70052617	profile #4
Trp53	3.810325787	2.260187037	9.848921784	profile #4
Tor4a	3.182854129	2.702703508	13.01319186	profile #4
Cdh24	0.118765438	0.099866734	1.144640038	profile #4
Rcan3	2.27302516	1.929602713	5.333239473	profile #4
Fdps	0.020227299	0.0129485	0.172776703	profile #4
Rps3a3	2.425428929	2.535886446	5.997812625	profile #4
Rps26-ps1	3.886164172	2.557523113	10.28701578	profile #4
Rpl13-ps6	1.003732002	0.771046555	2.723467912	profile #4
Nrm	1.894321474	1.671415754	8.951751092	profile #4
Eif4a1	40.3895505	35.54587275	109.8538594	profile #4
Kmt5c	3.100292324	2.832630915	7.610679509	profile #4
Tnip2	4.406402182	3.863931246	9.604726746	profile #4
Irak4	1.143056943	0.882570299	4.958420672	profile #4
Adamts14	0.416271751	0.645882378	3.250546405	profile #4
Unc5c	0.004915564	0.002724533	0.077182911	profile #4
9430015G10Ril	0.87822637	0.800853209	2.546666055	profile #4
Fcrl1	0.035863683	0.061221674	1.256427538	profile #4
Atxn7l3	7.783358285	7.877298174	16.87903839	profile #4
H2aj	26.17180489	24.87786692	55.60970059	profile #4
Rpl3	63.61719423	67.04784177	258.6341953	profile #4
Tmem26	0.01611444	0.029610345	0.293410939	profile #4
Alox5ap	3.013872481	3.483551525	51.08214335	profile #4
Rp2	1.602539713	1.805065651	8.142272772	profile #4
Atp8b4	0.125093481	0.109590879	3.606883938	profile #4
Serpinb6a	14.25689134	13.04511746	44.66999886	profile #4
BC002059	0.953443189	0.887776617	2.046908181	profile #4
Kif27	0.026625842	0.029990765	0.072823203	profile #4
Gm11353	2.625260044	2.607047362	8.681679493	profile #4
Arrb2	6.083273929	5.405804667	48.13389968	profile #4
Pyroxd2	0.395700183	0.311529994	2.879492234	profile #4
Golm2	1.65132791	1.598376383	3.351006805	profile #4
Alyref2	0.902649172	0.819072218	2.353485486	profile #4
Zfp941	0.019632911	0.011057041	0.038669991	profile #4
Zfp937	0.182588074	0.157906531	0.521498776	profile #4

Rpl36a-ps1	1.193221595	1.375902082	5.784012506	profile #4
Chst8	0.021400387	0.014806053	0.053482361	profile #4
Trim5	0.79580443	0.66403672	1.738508765	profile #4
Adgrg3	0.265627325	0.151003466	0.507219555	profile #4
Irak2	1.897388761	2.025366169	4.565595116	profile #4
Xcr1	0.06690044	0.0856526	0.274806425	profile #4
Tor3a	4.49512666	3.778887494	12.75767046	profile #4
Tmem219	2.893185798	2.433827355	8.542499193	profile #4
Fam78b	0.587406011	0.67588674	1.362062563	profile #4
Mfap2	1.827438744	1.787082069	19.45895202	profile #4
H2-Eb1	35.84710068	34.2066224	170.2745995	profile #4
Ifitm2	74.13579894	64.23220609	200.9994519	profile #4
Layn	1.501639741	1.673748215	6.426636883	profile #4
Rpl35a	59.33248378	56.43436828	113.4680197	profile #4
H4c9	3.055928782	3.584347942	9.592963715	profile #4
Atp8b2	2.438307346	2.685558988	8.609566893	profile #4
Gm8894	0.206270432	0.342986491	1.781971484	profile #4
Cd302	6.842572175	6.654325846	24.21496991	profile #4
Nsa2	10.82680421	10.31067226	22.12998239	profile #4
H3f3a	19.7164864	19.38584778	39.40313501	profile #4
Gmfg	0.717882611	1.073400394	7.940115749	profile #4
B2m	269.2174521	246.2065214	967.138086	profile #4
Ube2s	8.858983583	9.104178562	19.240126	profile #4
Tmem263	3.245444794	3.402863487	11.08012913	profile #4
Rpl26	126.3484499	117.4457955	271.3236692	profile #4
Trmt61a	3.100858136	2.523278874	5.938994664	profile #4
Dmkn	0.021115232	0.020148931	1.63628879	profile #4
Copz1	20.45039305	19.6478445	53.27086859	profile #4
Mcrip1	39.57052917	32.89638249	67.02226663	profile #4
Prcp	2.037633309	1.719414547	14.53136649	profile #4
Blnk	0.270399029	0.181897145	3.232919905	profile #4
Prpf40a	2.89337411	3.063542402	6.956327679	profile #4
Rpl15-ps3	0.761204237	0.71815377	3.557639213	profile #4
Fnip2	1.903622462	1.732017915	7.716938	profile #4
Sfmbt2	0.023311918	0.029031413	0.089020486	profile #4
Stk19	3.154100292	2.676925314	6.804815352	profile #4
H2-K1	93.73255858	88.68809258	267.8040764	profile #4
Exoc5	2.260171686	2.463229274	5.911190205	profile #4
Taok3	0.655342316	0.766194754	3.905066163	profile #4
Slc38a10	16.98517023	16.53086988	34.84127518	profile #4
Krt79	0.060884459	0.024861205	0.26159323	profile #4
Rps7	122.4578946	115.9068108	291.3695825	profile #4
Snrpa	9.361663449	7.390878068	15.15036484	profile #4
H4c4	0.149589585	0.143639604	0.296639721	profile #4
C1qtnf7	1.67236652	1.903978832	4.089714467	profile #4
Adgrg5	0.040699621	0.049857137	0.381729307	profile #4
Mdc1	1.913887194	1.615794063	4.176786369	profile #4
Rpl21-ps8	9.882770494	9.488814568	20.93630095	profile #4
Tmem91	0.052195758	0.06225889	0.142758388	profile #4
Tnnt3	0.215076371	0.263469093	3.973689725	profile #4
Ext1	4.448515426	4.701948871	11.37722185	profile #4
Mospd2	1.711465965	1.50196359	4.05482761	profile #4
Gm5805	25.783804	19.27439673	51.28003398	profile #4
Sphk1	0.394048249	0.593493731	4.198486626	profile #4
Rps12	34.26494456	37.14920305	155.0484748	profile #4
Rpl34	59.46769531	49.43033649	139.5641684	profile #4
Hsh2d	0.023332367	0.029872418	0.32487351	profile #4
Gmfb	7.552507629	7.059082087	21.05846749	profile #4

Pgghg	1.658547431	1.726136499	6.00736352	profile #4
Iah1	4.169307244	3.725268712	10.11070707	profile #4
Lmnb2	1.90927685	2.143402478	7.210631624	profile #4
Zfp954	0.844726531	0.910397491	2.913151471	profile #4
Cttnbp2nl	3.179292787	3.90342713	10.60369448	profile #4
Tgif2	0.096627532	0.10373124	0.446422919	profile #4
Hs6st2	0.070866796	0.041875719	1.839171194	profile #4
Gak	3.910361217	3.923468444	11.07515617	profile #4
Cks2	0.869508156	0.629910865	38.23340925	profile #4
Rpp25	0.986944355	0.918970454	5.861948133	profile #4
Rpl17	86.43440631	54.15203497	159.3140941	profile #4
Gm15772	17.8783461	15.22633558	38.37341332	profile #4
Tubb3	0.445997101	0.449230557	20.44856934	profile #4
Ftl1-ps1	2.114988731	2.16679265	38.15138294	profile #4
Arf2	10.54167129	10.65980965	21.2098873	profile #4
Rpl27a-ps4	0.517724698	0.296419173	1.019737191	profile #4
Fbxl12os	0.070676981	0.059012254	0.163265187	profile #4
Ifit3b	1.758814344	1.634158903	6.778619524	profile #4
Nsl1	0.104736009	0.039324116	1.615310403	profile #4
Cnr2	0.104611073	0.068416989	1.928642406	profile #4
Armc9	0.188611653	0.184333443	0.394593101	profile #4
Tubb4a	2.253982026	1.585869186	3.621211256	profile #4
Kcnj15	0.227493507	0.178594098	1.710862021	profile #4
Rps3a2	3.087805861	3.457475157	8.737762694	profile #4
Rpl7a	31.32725917	32.31268572	75.91195342	profile #4
Ncs1	2.048795415	1.727551073	4.770805272	profile #4
Zfp677	0.317343764	0.31754677	1.258532406	profile #4
AI413582	5.875350302	5.62348306	22.91324706	profile #4
Gm16477	1.96586113	2.108746549	4.837067985	profile #4
Zfp599	0.103602883	0.076227322	0.251290886	profile #4
Actg1	61.97398372	70.48687356	376.5766294	profile #4
Tcp11	0.081451171	0.052140947	0.130858141	profile #4
9130023H24Ril	0.55966344	0.444877573	1.033061681	profile #4
Nrg1	0.160974318	0.221610826	0.570725538	profile #4
Rpl35	103.4969635	101.1171714	242.5778683	profile #4
Zfp780b	0.463854259	0.356025905	0.759376083	profile #4
Ing2	1.686420895	2.039438143	3.717920532	profile #4
Lrrc40	1.639508567	1.675566692	3.678299556	profile #4
Mapk3	28.10944143	26.31419847	59.19171601	profile #4
Zfp26	0.354802314	0.365089406	0.88240336	profile #4
Clip2	4.167129863	3.678409361	12.90925946	profile #4
Numbl	0.976603206	1.061726275	4.329494189	profile #4
Hspb11	1.340934467	1.215590555	3.056283298	profile #4
Cd300lb	0.17453907	0.232341895	11.47145055	profile #4
Zfp35	2.466481335	1.864038864	4.228353679	profile #4
Gm9396	0.580613068	0.735575968	2.941667211	profile #4
Slc39a4	0.084585717	0.039718037	0.455363313	profile #4
Alg11	1.465056516	1.131490092	2.812130895	profile #4
Zfp947	0.22925321	0.254134838	0.65072157	profile #4
Gm10131	8.153140494	8.287597506	28.3565897	profile #4
Nmral1	0.809383071	0.74916118	3.079914699	profile #4
Arhgap22	0.322415861	0.21520721	2.063335402	profile #4
Ly6m	0.014017137	0.016501299	0.041413312	profile #4
Eno1	27.80172966	24.29085724	94.08807374	profile #4
Sema3e	0.024924388	0.028074285	0.088366241	profile #4
Gm10132	11.82173247	12.58789739	26.05754935	profile #4
Jazf1	0.738392123	0.696631811	1.761459281	profile #4
Gng8	0.243282986	0.411042944	3.849620393	profile #4

Ccdc102a	3.4558535	3.72682766	11.51164965	profile #4
Jakmip1	0.009913402	0.006346062	0.019658514	profile #4
Slc22a21	0.228565867	0.194330526	0.50452526	profile #4
Brwd3	0.489560557	0.360615533	0.886212103	profile #4
Pcdhb5	0.186256073	0.174916661	0.525224332	profile #4
Gm8730	4.193369255	4.645058145	11.36753573	profile #4
Mapk15	0.0830891	0.085103147	0.183694054	profile #4
Gm10136	3.424225958	2.335237948	7.865253445	profile #4
Utp14a	2.353453628	2.322674623	4.812297131	profile #4
Prpf38a	2.734076032	2.663685933	5.945137113	profile #4
Alms1	0.109587147	0.126320852	0.24282954	profile #4
Dupd1	0.03786607	0.041975803	0.256904804	profile #4
Cdc42ep5	2.263850021	2.434641643	8.93199371	profile #4
Ppcdc	1.17341339	0.985897886	2.255178111	profile #4
Gpx1	40.43180175	39.72222628	236.9564523	profile #4
Crem	1.603555786	1.335407286	3.271251764	profile #4
Dhrsx	14.98253623	14.76898929	31.43092638	profile #4
Dpp3	8.073851131	7.22977085	25.24270798	profile #4
Pepd	6.547722379	6.140370751	13.52956934	profile #4
Nr6a1	0.082553909	0.073447545	0.165948564	profile #4
Pym1	6.100378026	5.779555178	13.62014792	profile #4
Trerf1	0.399259504	0.35062522	1.819269612	profile #4
Dzip3	0.509686026	0.579840174	1.997768579	profile #4
Fbln2	20.01790266	22.05152798	104.7175325	profile #4
Vrk2	0.435388326	0.422164585	1.59623117	profile #4
Hcst	0.325690156	0.301868642	3.093532367	profile #4
Prr36	0.038462203	0.018060297	0.1025158	profile #4
Med14	2.265900748	2.487152339	5.066428604	profile #4
Cenpj	0.873168586	0.737825427	2.066819406	profile #4
Fam172a	1.352955506	1.384613535	3.524863139	profile #4
Rab44	0.033792593	0.053743559	0.246465809	profile #4
Ano6	7.402416738	7.284293715	17.23881993	profile #4
Ifi27	18.93030184	17.94356392	52.55209243	profile #4
Hvcn1	0.17916743	0.183510378	3.336882891	profile #4
Rpl19-ps1	0.144021106	0.184390152	0.651278918	profile #4
Tank	2.452415014	2.547388059	5.142461014	profile #4
Selenop	87.14951129	86.53210208	308.3906783	profile #4
Snord58b	2.10384491	0.825564998	7.672181877	profile #4
Gm22573	1.25171285	1.022234249	2.48217649	profile #4
Snord104	22.4810856	15.1427852	46.88460249	profile #4
Mir142b	0.402474678	0.384056146	3.71889701	profile #4
Gm22581	0.340327853	0.324753359	1.241088245	profile #4
Dhrs3	26.455113	22.80387088	56.56039587	profile #4
Med18	0.926618379	0.574047461	2.217560077	profile #4
Gm1976	0.688718848	0.518067462	1.406565321	profile #4
Gm10154	13.58497393	9.850809466	29.84047163	profile #4
Cdc26	3.360197049	2.672046294	5.485891041	profile #4
Fkbp15	2.636035086	2.721100401	7.119866455	profile #4
Gm10155	1.071068827	0.863502223	3.491808157	profile #4
Anks6	0.633863372	0.691550403	1.902765808	profile #4
Gm12918	5.553983747	4.44442333	15.2599215	profile #4
Wdr6	3.835739338	3.443871089	9.619253737	profile #4
Rps13-ps1	4.350749126	4.845162333	9.062219293	profile #4
Plekhd1	0.035251019	0.033637819	0.224964967	profile #4
Zfyve26	0.625038483	0.622062772	1.26655553	profile #4
Rdh11	1.305694099	0.945967527	4.696753072	profile #4
Mthfs	0.197034443	0.149807945	0.635330589	profile #4
Hmgn3	2.476435956	1.961378233	9.221630921	profile #4

Gm6254	0.086351843	0.101655392	0.275540114	profile #4
Rpl17-ps9	0.766815079	0.657545274	3.120947013	profile #4
Flvcr1	0.661591561	0.819003256	3.676037695	profile #4
Pilrb2	0.239213391	0.157979151	2.113084329	profile #4
Pilrb1	0.096126027	0.08352676	1.358411354	profile #4
Fxyd6	7.29510562	5.917051687	22.16985998	profile #4
Zfp575	0.085789235	0.076080185	0.523421383	profile #4
Tnfsf18	0.022763629	0.025234243	0.183820427	profile #4
Zbtb6	1.293781791	0.900970935	2.76606321	profile #4
Rnasel	0.621615567	0.591901018	4.676553567	profile #4
Zfp772	0.694352178	0.588461974	1.329550755	profile #4
Oas1g	0.281374025	0.267353573	3.214133184	profile #4
Gm10184	0.286444072	0.296113429	0.783440955	profile #4
Myo1h	0.031739267	0.027579192	0.107668556	profile #4
Bub3	4.914303976	4.431775501	9.830828349	profile #4
Rps12-ps3	80.92147718	83.89977253	301.7952676	profile #4
Gm7027	0.38191138	0.505682504	1.240351381	profile #4
Rpl7a-ps11	2.656569095	2.993959486	7.691338386	profile #4
Polr1c	6.874835944	6.018976288	14.42996631	profile #4
H2-T23	15.46492751	14.18707925	36.96753805	profile #4
Rplp0	391.6019907	396.9459827	865.4516695	profile #4
Capn6	0.024133244	0.020970092	1.586109185	profile #4
Tmem128	2.576325924	2.239357415	7.569646517	profile #4
B3galt4	1.627466038	1.096507483	4.009146485	profile #4
Tspan6	3.078149785	2.354062523	17.92348315	profile #4
Hdac8	0.879484127	0.775810876	1.587074614	profile #4
Syngap1	0.606943912	0.637559684	1.871503276	profile #4
Ankrd23	2.87723161	3.818352904	8.30797448	profile #4
Lpar5	0.056182491	0.034411846	1.213857803	profile #4
Xlr4b	0.13299449	0.101824115	0.735063237	profile #4
Pi15	0.362694782	0.330559898	2.218867408	profile #4
Nnat	0.642832948	0.427561072	1.295941567	profile #4
Myl9	35.05201157	34.00820276	107.6350335	profile #4
Map7d3	0.220814097	0.106940573	0.547350632	profile #4
Sptbn2	0.180528214	0.105659513	0.404019274	profile #4
Zfp991	0.700306949	0.74716887	3.533398376	profile #4
Zfp760	0.838019574	0.544997305	1.503813269	profile #4
Zfp948	1.466401731	1.454178901	4.636500419	profile #4
Gtf2f2	5.834627026	4.967597395	10.11605898	profile #4
Gm14648	2.02658271	1.917772107	4.869984179	profile #4
Cenpm	0.154153499	0.272209608	8.617560681	profile #4
Ninl	0.221241673	0.315326291	1.105969767	profile #4
Zfp442	0.480637172	0.374679288	0.834270017	profile #4
Zfp120	1.407880545	0.97691582	3.317131653	profile #4
Col8a1	4.33125158	4.778838265	70.3718261	profile #4
Lgals1	192.8215999	154.9482305	1154.717521	profile #4
Il2rb	0.149573175	0.17444561	0.66032121	profile #4
Phf11d	2.00585732	1.659433349	5.897925947	profile #4
Apol9b	0.152199542	0.035266854	2.376141766	profile #4
Aup1	9.762713771	9.150494309	19.54294001	profile #4
Dok1	1.427303748	1.407428822	28.81434013	profile #4
Cep152	0.686185165	0.714039839	1.470457189	profile #4
Rpl34-ps1	35.82586044	41.23589005	80.70216618	profile #4
Gm10240	0.681589254	0.731535517	1.947993672	profile #4
Pnp2	0.13494049	0.06438259	1.476279895	profile #4
Zfp467	2.130412329	1.691923023	4.09078671	profile #4
Rpl7a-ps3	0.366430084	0.320626979	0.841000967	profile #4
Zfyve19	2.48841871	2.293320648	4.887313701	profile #4

Mgam	0.021270491	0.012520057	0.06808011	profile #4
Gm4841	0.163088753	0.124500231	0.550137073	profile #4
Gpr88	0.053686825	0.062200103	0.24716404	profile #4
Myoz1	0.134962696	0.163406553	1.204354132	profile #4
Trp53i11	5.674431587	4.164069306	14.63820301	profile #4
Psrc1	0.027960558	0.078774893	3.574953951	profile #4
Il3ra	2.183971123	1.741649966	13.03055386	profile #4
Rap1a	24.07437719	22.76051349	51.14234305	profile #4
Sf3b4	6.180983528	6.700554044	16.36069745	profile #4
Gm128	0.04344894	0.027813828	0.116340614	profile #4
Ssb	21.90362799	20.50841049	45.43453216	profile #4
Syt11	2.88627428	2.924709402	6.497988722	profile #4
Zbtb34	0.834019681	0.789429841	2.223585117	profile #4
Gm5641	2.64394488	2.433904208	8.791738202	profile #4
Gm10259	0.207048811	0.277776458	0.630697832	profile #4
Zfp874a	0.698494854	0.598850356	1.238619715	profile #4
H2ac6	0.12002726	0.147033693	0.312207208	profile #4
H2ac8	0.394470921	0.313682222	1.518725696	profile #4
H4c17	0.105866261	0.129686435	0.45355475	profile #4
Lyz1	0.513051649	0.349694828	4.294764876	profile #4
Lyz2	85.19658501	92.46093358	2357.564522	profile #4
Tmem19	3.161469027	3.143212367	11.35587301	profile #4
Scyl2	1.611274566	1.667152447	3.252331754	profile #4
Gm4925	0.059301202	0.056587389	0.164384301	profile #4
Cd300ld3	0.162973901	0.063952218	0.451767418	profile #4
Strada	1.494747631	1.320844699	3.091466355	profile #4
Marcks	14.31924868	12.80074423	68.90369865	profile #4
Gm10275	46.24438341	48.11101224	147.3620261	profile #4
Zfp820	0.388314541	0.268077128	0.90280125	profile #4
Wfdc17	4.500023609	4.401552724	156.6262665	profile #4
Slfn9	0.225021467	0.274034987	4.509747111	profile #4
Gm10277	0.037087788	0.022716317	0.079446192	profile #4
Cacng7	2.755117233	2.455387908	8.029647088	profile #4
Tlcd3a	0.60431156	0.520157291	2.418650129	profile #4
Nlrp1a	0.011288924	0.013079042	0.667430964	profile #4
Irgm2	4.868652994	4.761284564	9.640127717	profile #4
Atxn1l	3.099483207	3.091426092	6.4927152	profile #4
Gm12166	1.055162898	0.9125301	3.060791743	profile #4
Spdl1	0.049200274	0.031495543	3.859999988	profile #4
B3gnt9	2.060363218	1.719840054	11.59484958	profile #4
Fcho1	0.057163515	0.118647298	1.265323327	profile #4
Ssbp4	14.58157237	13.17407609	40.40186674	profile #4
Sp140	0.409213816	0.29763972	4.677675145	profile #4
Sp110	0.749034526	0.570505468	2.989486447	profile #4
Fam149a	0.808464419	0.805321046	2.495769951	profile #4
Fat1	1.726617656	1.862016066	5.331274621	profile #4
Gmppb	2.542743973	2.40644045	9.904818157	profile #4
Mpzl3	0.237551344	0.227564398	0.465194788	profile #4
Rnf213	2.976377462	2.5940183	9.068555021	profile #4
Gm10288	5.516226504	6.004006237	14.85145821	profile #4
Evi2	0.035773067	0.022396783	0.477120154	profile #4
Capza1	6.304301197	5.90843397	31.82680412	profile #4
Nlrp1b	0.053999349	0.033074669	1.058160386	profile #4
Serpinh1	94.46375596	79.77938429	589.0801774	profile #4
Tlnrd1	6.757927468	7.761877922	23.90179739	profile #4
Adamtsl3	0.81284917	0.878310221	2.224960152	profile #4
Cldn3	0.110288931	0.041409012	0.871423359	profile #4
Peg12	0.480985144	0.392143374	2.34817664	profile #4

Mkrn3	0.167091625	0.171141861	0.352616265	profile #4
Zfp992	0.475464679	0.217702195	1.353515043	profile #4
Runx3	0.204157043	0.163363851	2.648382591	profile #4
Utp3	15.02209099	14.52965734	29.32486221	profile #4
Tmem35b	0.678517521	0.647466383	2.474379077	profile #4
Rbm47	0.065656371	0.076067695	2.367248067	profile #4
Gm1673	3.562423418	3.094943221	10.03657481	profile #4
Skint3	0.036131607	0.0271319	0.2016389	profile #4
Ccnyl1	4.953355236	4.761331463	9.93154979	profile #4
Il1rl2	0.393524741	0.359942461	2.366893421	profile #4
Gm19680	0.275321409	0.344573342	1.385061775	profile #4
Camkmt	1.269741636	1.045091057	2.551959162	profile #4
Lrrc73	0.135923869	0.157477716	0.74162975	profile #4
Nr2c2ap	0.739643078	0.416558886	1.428822909	profile #4
Spx	0.096014071	0.030731692	0.410064504	profile #4
Gm4799	0.214747299	0.201673319	0.513814311	profile #4
Srsf3	21.02691892	19.34624071	48.91906245	profile #4
Smim15	8.259115795	7.621114156	26.83737688	profile #4
Wfikkn1	0.094961451	0.110019768	0.213008583	profile #4
Naip5	0.222351018	0.210379109	2.027142598	profile #4
Zfp65	0.964136026	0.642229766	2.32360121	profile #4
Tbpl1	4.259144296	3.537637326	7.269528425	profile #4
Rpl23	120.3922278	116.1552862	241.2124925	profile #4
Dtnb	0.216891228	0.179370178	0.454260772	profile #4
H2ac7	0.080917112	0.0952575	0.398446262	profile #4
Gprin2	0.026582284	0.027586794	0.142355567	profile #4
Gm10335	13.45858774	15.60106163	32.81988914	profile #4
Nt5dc2	13.88360394	11.7400532	39.91770251	profile #4
Mta2	11.54570348	11.43813441	24.71219597	profile #4
Eml3	3.932161932	3.322160458	9.42171875	profile #4
Ganab	16.02343194	15.91913721	39.56284571	profile #4
Ints5	2.892042912	2.619985396	6.076194441	profile #4
1810009A15Rik	0.032569016	0.030586189	0.175333796	profile #4
Bscl2	2.527325308	2.127060156	6.23974636	profile #4
Ttc9c	3.290144821	2.776335302	8.008929893	profile #4
Gm960	0.022985815	0.014714364	0.069296218	profile #4
Csf2rb	0.259065674	0.287397179	11.13384328	profile #4
Csf2rb2	0.214141984	0.141770919	6.732235718	profile #4
Ncf4	0.767926141	0.490126169	14.85534664	profile #4
Gspt2	0.171379062	0.172929775	0.636135683	profile #4
Smpd5	0.350698026	0.18488174	0.773329537	profile #4
Apcdd1	1.74855207	1.861965669	3.572950724	profile #4
Ccdc112	0.288684934	0.268057753	1.572664587	profile #4
Ppia	25.98624386	23.94178681	142.2431349	profile #4
Fndc1	1.448454594	1.449469703	13.17611632	profile #4
6720489N17Rik	0.220590639	0.115810322	1.124836736	profile #4
Ccnf	0.060703012	0.070328867	4.971539632	profile #4
Septin5	1.440617328	1.432762352	13.17292347	profile #4
Tuba1a	42.18164335	34.72431059	193.9805751	profile #4
Trim6	0.160776052	0.126344584	0.293154477	profile #4
Nanos1	0.035927929	0.044987478	0.687702577	profile #4
Ppp1r3e	0.848919876	1.09183426	2.285016065	profile #4
Ear2	0.236763488	0.307138087	1.643276786	profile #4
Gm10384	0.020800702	0.026631129	0.089114863	profile #4
Slfn2	5.002553819	4.093095693	42.43319596	profile #4
Slfn10-ps	0.063911334	0.014397665	1.08259423	profile #4
Zfp9	0.955566277	0.870672704	2.757434457	profile #4
Zfp783	0.142938004	0.06449541	0.44597122	profile #4

Spef2	0.004667667	0.005494886	0.034503683	profile #4
Rpl37rt	18.39285505	17.74772654	37.02330115	profile #4
1500011B03Rik	0.808069734	0.561724638	2.297387293	profile #4
4930522L14Rik	0.300327948	0.165005443	0.665623085	profile #4
Zfp951	0.195706503	0.195831697	0.452085789	profile #4
Ahnak2	0.558909275	0.44593683	2.866166371	profile #4
Rybp	1.279921086	1.37942506	2.586840908	profile #4
Nfxl1	0.599411787	0.636242581	2.84188735	profile #4
Sod3	9.286634833	8.459920572	32.37867929	profile #4
Adam4	0.067984404	0.035276723	0.136636791	profile #4
Oip5	0.052901753	0.049681054	2.15349224	profile #4
Gm10451	0.273027983	0.174778797	0.712793843	profile #4
Gpr174	0.031512948	0.040879727	0.095003293	profile #4
4930599N23Rik	0.748199113	0.557256374	2.041167336	profile #4
2410018L13Rik	0.0722726	0.032144437	0.221165708	profile #4
Zfp449	1.270731811	1.034937822	2.280308739	profile #4
H2-T-ps	0.132229593	0.124179354	1.683436978	profile #4
H2-D1	107.4599607	101.3898814	364.260122	profile #4
Lst1	2.973949085	1.489952761	62.14142485	profile #4
Ly6g6d	0.24076617	0.263792831	0.510872371	profile #4
C4b	1.585547749	1.849175985	8.372714726	profile #4
H2-Ab1	34.36896102	32.51929273	194.0335083	profile #4
Zfp414	2.82273637	2.965483222	6.504286555	profile #4
Gm4924	0.141815769	0.181566659	0.368882081	profile #4
Arhgdig	0.130155881	0.14428214	0.657554053	profile #4
Sft2d1	5.300748906	4.824712538	17.94315867	profile #4
Ifi204	1.201825028	1.14193986	19.04256656	profile #4
Ifi207	2.02026879	2.826549985	16.01106587	profile #4
Ifi213	0.182702321	0.109770434	1.452767694	profile #4
Pappa2	0.008268552	0.005064498	0.041545867	profile #4
Gm4951	0.289621272	0.295655652	1.482069455	profile #4
Csnk1g3	2.886289166	3.310280185	7.074896712	profile #4
Pcdhb22	0.392805636	0.315533041	1.069034517	profile #4
1700066B19Rik	0.048943872	0.031136029	0.080335306	profile #4
Ecscr	6.942473398	6.129820109	22.08568659	profile #4
Gm10548	0.123237777	0.099151667	0.244012832	profile #4
Rpl27-ps3	4.774620658	4.939005477	11.19330956	profile #4
Wdfy1	3.72583916	3.893836824	8.697723258	profile #4
Catip	0.425415274	0.359564099	0.889776806	profile #4
Pgap1	0.270279034	0.263635634	0.853542358	profile #4
Tmem88b	0.257482851	0.305386046	0.604086318	profile #4
Faap20	4.479015095	4.130311225	12.85718496	profile #4
Rpl31	194.508043	188.1464575	487.9899959	profile #4
Cenps	0.490456371	0.323902774	3.514574673	profile #4
Cplane2	0.181195261	0.221964648	0.546256293	profile #4
Efcab7	0.585837187	0.580681597	1.481283578	profile #4
Cdkn2b	0.465171739	0.273805477	3.315771777	profile #4
Iqck	0.03603035	0.033932611	0.146486213	profile #4
Rhog	12.90753299	10.46350339	70.08418551	profile #4
Ggh	3.29762968	3.57377437	14.00124387	profile #4
Omp	0.019849924	0.024316206	0.059842326	profile #4
4632427E13Rik	0.135235222	0.084667999	0.216676801	profile #4
Gm5921	0.157430571	0.150226044	0.382739232	profile #4
Fam169b	0.018169163	0.01483818	0.060861414	profile #4
Snrnp40	3.75733619	3.533759652	7.179747811	profile #4
Rbm15b	3.788924191	3.419013246	7.336354534	profile #4
Rpl13a	5.012772464	4.414369187	15.090318	profile #4
4930513N10Rik	0.064988013	0.060034485	0.315189797	profile #4

Nlrc5	0.48175913	0.595430688	2.125021451	profile #4
Dnajb14	1.159234324	1.202250343	2.852271669	profile #4
Zfp382	0.543813697	0.379371905	1.507464127	profile #4
Rec114	0.144979132	0.172930528	0.396526236	profile #4
Phldb3	0.156713291	0.236032912	1.0784205	profile #4
I830077J02Rik	0.164152386	0.15214601	2.40332908	profile #4
C5ar2	0.3107313	0.248884329	2.46475037	profile #4
AI429214	0.455651385	0.479939859	1.322593853	profile #4
Mir100hg	0.561010241	0.341563248	1.578545791	profile #4
Pira11	0.065250829	0.041509828	0.364143934	profile #4
S100a16	29.05309637	25.03652218	63.05967759	profile #4
Spc24	2.158628279	2.032949797	8.905157861	profile #4
Mex3a	0.144188748	0.084082905	1.8695798	profile #4
Arfip1	1.622255467	1.484054076	4.381147503	profile #4
Cass4	0.070535671	0.082801756	0.308526999	profile #4
Ripor3	0.25083542	0.197729189	1.451105902	profile #4
Zfas1	2.775221321	2.533275776	8.759227107	profile #4
Mgst2	0.049996854	0.044143092	0.273488525	profile #4
Mafb	2.838840522	4.774682006	37.7928531	profile #4
Tspyl3	0.50520141	0.560706972	2.478054095	profile #4
Foxs1	1.363178507	1.352062691	9.14107406	profile #4
Syndig1	0.093959781	0.066366979	0.295186506	profile #4
Fndc10	1.081415608	0.925742979	2.498090865	profile #4
Atxn7l3b	6.885921444	6.757522231	14.06960453	profile #4
Ism1	0.571017776	0.457161084	1.93705356	profile #4
Plxnc1	0.346623561	0.411150948	4.397100285	profile #4
Gas2l3	0.127071034	0.111207224	2.184258834	profile #4
Hps6	2.529414126	2.142304423	4.700247097	profile #4
Morrbid	0.773305156	0.868136744	2.882252111	profile #4
Platr25	0.24791206	0.229234553	1.051882196	profile #4
Zfp934	0.342686616	0.298476794	0.607625091	profile #4
Zfp808	0.149332108	0.138409712	0.398340789	profile #4
Grk6	6.323597585	6.190871746	15.22385828	profile #4
Ifit3	5.956191707	5.213919833	31.09761743	profile #4
Chst14	3.543317742	3.367503735	9.770741189	profile #4
Ano3	0.025649536	0.022646421	0.112576752	profile #4
Fibin	5.47166722	5.571948238	17.0908313	profile #4
Wt1os	0.011243045	0.013025888	0.070614153	profile #4
Nrbf2	3.066023519	2.5193048	8.59820598	profile #4
Fjx1	0.295370696	0.399238903	2.574364607	profile #4
Zfp408	1.444091871	1.441031119	2.884725072	profile #4
Cd80	0.108840889	0.214542991	3.751625714	profile #4
Lrrc55	0.166895047	0.160256735	0.55848971	profile #4
Ccdc162	0.004173604	0.005112675	0.013410489	profile #4
Heg1	12.32704238	10.869852	23.72651582	profile #4
Cerkl	0.052556307	0.041792635	0.189782277	profile #4
Tnk2os	0.066500845	0.063457553	0.196475226	profile #4
Cenpw	0.201029174	0.160945454	1.442975076	profile #4
Wipf1	4.618414565	4.546768421	17.4953591	profile #4
4930594M22Ril	0.021319479	0.012006897	0.049248406	profile #4
Commd6	10.30153995	9.933008212	19.89553638	profile #4
Gm5464	0.016104589	0.015367591	0.059523056	profile #4
Gm12359	0.304670903	0.218046139	0.609956229	profile #4
Gm16409	0.250824845	0.176665827	0.627253962	profile #4
Hoxb2	0.134214623	0.074733295	0.365912577	profile #4
Zc3h3	1.16174454	1.070286051	2.251980328	profile #4
Selenos	17.32168871	15.74254586	40.5827095	profile #4
Selenom	25.6613812	24.5763063	81.66506991	profile #4

Sox4	4.056123934	3.193102909	11.53102653	profile #4
Ywhaq	13.88919023	15.54991615	43.22942789	profile #4
Cep295nl	0.095235778	0.090877483	0.375162571	profile #4
Selenoh	13.87309932	13.5375141	41.75350443	profile #4
Ass1	1.639567536	1.21885404	6.174563605	profile #4
Ighm	2.257784781	2.773478169	12.37439726	profile #4
Trdv4	0.104244568	0.122719121	0.770582263	profile #4
Gm22270	2.13771175	1.67297185	6.80389112	profile #4
Snord89	0.8550847	0.495338999	3.836090939	profile #4
F630028O10Ril	0.542472215	0.429945231	3.790686524	profile #4
Fam170b	0.02776249	0.030775647	0.075739048	profile #4
Srsf3-ps	0.09738727	0.083123249	0.27815257	profile #4
Psme2b	29.94839663	23.85094204	49.96084507	profile #4
Dnm3os	0.249148039	0.150415532	4.348505205	profile #4
Gm2000	29.43491777	28.38563293	75.25125421	profile #4
Sf3b5	8.808652391	9.007922171	18.90680488	profile #4
Smim1	0.554354552	0.680972899	1.818780129	profile #4
Gm4294	11.50597284	9.051634472	30.99486506	profile #4
Sarnp	8.406097969	6.089755925	13.96227068	profile #4
Smim24	1.246980476	0.834904666	4.365661707	profile #4
Gm10941	0.132852143	0.187146004	1.449739512	profile #4
Abracl	14.79059938	14.24099621	60.26303563	profile #4
Zfp984	1.198411891	1.047832497	4.419672081	profile #4
Ddi2	4.18457131	4.699835323	8.891715865	profile #4
Zfp995	0.116917178	0.197539425	1.137037556	profile #4
Trim30c	0.026972371	0.0317525	1.009397198	profile #4
Smarcd2	6.77468193	6.652924935	18.92927571	profile #4
Tomt	0.014896128	0.019071505	0.088618132	profile #4
Gm11627	0.877811152	0.751548826	2.000719843	profile #4
Vps25	0.155511219	0.13448982	0.334081001	profile #4
D030028A08Ri	0.135773606	0.077497372	0.508973946	profile #4
Haus5	0.294720966	0.252328983	0.525172508	profile #4
Slfn1	0.223477088	0.223750844	7.266758103	profile #4
Evi2a	1.329901092	1.248309564	18.49907676	profile #4
Rad54b	0.008133805	0.018847212	0.55184325	profile #4
9530053A07Ril	0.007816362	0.008804179	0.021667136	profile #4
Zfp59	0.929193067	0.719616405	1.657432367	profile #4
Dact3	9.47201553	10.80995747	29.50358139	profile #4
Gm14322	0.664372077	0.77485012	1.749795867	profile #4
Gm14410	2.510340404	2.635748801	5.564388019	profile #4
2210418O10Ril	0.05974296	0.055373262	0.220036279	profile #4
Mon1b	1.753751968	1.619003423	4.218260622	profile #4
Ifi47	1.483345476	1.264875375	8.906928496	profile #4
Naip6	0.081039561	0.114546536	1.0569338	profile #4
Naip2	0.410696922	0.344260482	3.611965524	profile #4
Sec61g	16.24844506	13.30012621	29.33310202	profile #4
Zfp429	0.494437348	0.475112488	1.997276263	profile #4
Zfp456	0.064287367	0.078752185	0.254966769	profile #4
Serpina3i	0.041622831	0.024499681	1.188745792	profile #4
Ifi27l2a	29.96490121	25.41955639	202.4127667	profile #4
D130040H23Ri	0.123110165	0.068795104	0.275979562	profile #4
Serpinb1c	0.028343287	0.063850516	2.880835712	profile #4
Cyp4v3	0.887283331	0.788686729	5.17745076	profile #4
Ccdc82	1.788321652	1.927969179	3.706986099	profile #4
Esd-ps	0.166574942	0.12865948	0.398554903	profile #4
Kdelr2	12.52644673	10.79409853	58.34675441	profile #4
Gm4204	14.90056873	10.44597921	29.06748022	profile #4
Tlr5	0.154604052	0.141490878	0.599038836	profile #4

Ccr5	0.513010266	0.426388562	15.03554971	profile #4
Tmppe	1.191439483	1.058422451	2.27833237	profile #4
Clec7a	0.754449312	0.686438488	9.67857749	profile #4
Ifit1bl1	1.240342642	0.934067826	2.724917068	profile #4
Gm11110	0.22845305	0.154394709	0.874765061	profile #4
Ms4a6c	0.768124276	1.222641626	21.7800378	profile #4
Arpc4	10.63497519	11.64398784	51.79631793	profile #4
Mroh2a	0.204910194	0.208732241	0.503618884	profile #4
Rpl36a	58.98138738	58.53679841	157.2700198	profile #4
St6galnac4	3.917928971	3.645333911	8.794839161	profile #4
Cldn34c1	0.073073896	0.089515705	0.242625313	profile #4
Utp14b	0.146986822	0.115031793	0.538521426	profile #4
Nhsl2	0.44111112	0.338292481	0.809359248	profile #4
Zfx	2.405246804	2.353738505	4.764791836	profile #4
Tmsb10	31.81861913	27.62795967	354.3423656	profile #4
H2-DMb1	2.533584296	2.091431328	16.559735	profile #4
Kifc1	0.110430663	0.103707559	6.635983916	profile #4
Haus3	1.123934713	0.957152082	3.732775515	profile #4
Pglyrp2	0.021014146	0.019734789	0.094273906	profile #4
Rpl39	94.95764151	85.5917598	252.9653912	profile #4
Gm17396	0.475430813	0.283507867	1.129228006	profile #4
2610203C22Rik	0.312035953	0.188899306	0.566296822	profile #4
Ulbp1	0.337190724	0.374292007	2.446505074	profile #4
Rps19-ps7	0.388659698	0.238054551	1.445102751	profile #4
Rps19-ps3	2.497549114	2.447603132	5.243029035	profile #4
Eef1akmt3	0.034045302	0.01624364	1.16395048	profile #4
Brms1	13.74334565	11.96197432	29.29821622	profile #4
B230307C23Rik	0.215159419	0.178793404	0.623219202	profile #4
Gm12174	3.871281654	3.591022159	9.205756742	profile #4
Rpl10-ps1	1.312455586	1.689528368	7.491036832	profile #4
Gm15427	94.65400713	100.1498962	225.5955763	profile #4
Rps15a-ps7	1.799604279	1.250983696	4.800412977	profile #4
Rpl7-ps9	0.944133671	0.683654829	1.931773352	profile #4
Gm13680	5.527740733	4.996443821	14.26077691	profile #4
Rps6-ps4	13.8853764	13.77993453	36.00810694	profile #4
Gm11841	0.047156992	0.027757152	0.10893356	profile #4
Gm14681	6.937717452	7.230255383	20.73937032	profile #4
Pira1	0.013584535	0.01505891	0.395307366	profile #4
Rps2-ps13	24.97898401	28.97340016	50.96195998	profile #4
Gm15931	0.436612582	0.262327767	1.177717654	profile #4
Rpl17-ps5	5.93166864	7.232998012	16.27055508	profile #4
Dnajb3	0.132115855	0.155529847	1.257740552	profile #4
Rpl17-ps8	0.753849607	1.00741926	3.057753647	profile #4
Gm11585	5.759199019	4.500707598	15.78407079	profile #4
Gm15753	0.320589355	0.245451726	0.724869756	profile #4
Gm11824	0.100610489	0.06440578	0.161639193	profile #4
Gm14276	0.177325987	0.306711506	2.168456961	profile #4
Rpl5-ps1	0.310634819	0.345822369	1.580592646	profile #4
Gm715	0.339564321	0.262273357	0.771973206	profile #4
Actr3-ps	1.178647379	1.016186054	5.047527236	profile #4
Gm8401	0.024472822	0.025397618	0.147558306	profile #4
Gm9703	0.137112707	0.158855072	0.374058154	profile #4
Gm12366	0.265089278	0.312069243	0.580027496	profile #4
Rpl13-ps1	0.063709935	0.07804483	0.13647381	profile #4
Gm14150	0.314314896	0.308029227	2.221882319	profile #4
Rpsa-ps9	0.313793817	0.349339207	1.014817659	profile #4
Gm16166	0.058811421	0.056120021	0.173756998	profile #4
Gm7816	0.348878804	0.410708215	0.777499168	profile #4

AA414768	0.744853788	0.705073265	6.345977863	profile #4
Gm11539	0.506603144	0.475760756	1.227529239	profile #4
Gm7363	0.348004421	0.409678871	1.179048801	profile #4
Gm11703	6.432197296	6.400935774	13.19322987	profile #4
Gm15542	2.403387317	2.509883536	4.848363532	profile #4
Rps8-ps2	0.376840212	0.353897891	1.644896754	profile #4
Gm11263	1.363681823	0.835256823	2.616871634	profile #4
Rpl21-ps15	96.30673486	92.17886229	216.6700234	profile #4
Gm14414	0.323103581	0.272952601	1.178275751	profile #4
Gm14586	10.02838306	9.526098534	19.28722077	profile #4
Gm13436	4.65363437	4.44001418	11.76666884	profile #4
Gm11478	1.847269048	1.84334127	4.860756298	profile #4
Gm6136	44.03893875	39.20950321	103.7638919	profile #4
Gm15710	12.05319864	11.59716713	33.77086976	profile #4
Esrp2	0.159969774	0.091589427	0.346593164	profile #4
Rpl3-ps2	0.309915377	0.179529785	0.775025489	profile #4
Rps15a-ps3	1.508393207	1.655310072	5.020908899	profile #4
Gm14438	0.682023027	0.707795783	1.607066592	profile #4
Rpl3-ps1	11.85803494	12.8396001	45.40536729	profile #4
Rpl10a-ps1	9.110325943	7.3316231	30.51862562	profile #4
LOC115489189	1.356624764	1.047832497	6.897586592	profile #4
Gm15880	0.044270011	0.036153916	0.110636535	profile #4
Tmem240	0.1930623	0.108697894	0.449855696	profile #4
Gm5577	0.044790151	0.042063292	0.244384661	profile #4
Far1os	0.270579163	0.19795564	0.61321708	profile #4
4933421O10Rik	1.161377062	0.849814763	2.08407332	profile #4
Abhd11os	2.178875042	1.95315287	4.392648679	profile #4
A730046J19Rik	0.009041478	0.009383144	0.029051786	profile #4
Gm11520	0.270211028	0.248256889	0.651175035	profile #4
Snhg15	1.033422971	1.523559246	4.626045176	profile #4
4933439K11Rik	0.028892218	0.018495346	0.174204952	profile #4
1700125H03Rik	0.054214315	0.056263002	0.217922627	profile #4
Brip1os	2.685268872	2.230473854	8.551056587	profile #4
6330418K02Rik	0.222485182	0.194674932	0.408504129	profile #4
D330050G23Ri	0.039797582	0.056964476	0.215565252	profile #4
Gm16104	0.346268738	0.11014079	1.364057429	profile #4
2410004I01Rik	0.079241211	0.074416945	0.42241346	profile #4
3010003L21Rik	0.161521437	0.187134367	0.386463655	profile #4
Gm13056	1.500946548	1.29410506	3.791428689	profile #4
Foxd2os	0.291747261	0.194046948	1.353060233	profile #4
1110046J04Rik	0.199102042	0.156258359	0.932123885	profile #4
Appbp2os	0.123835801	0.050566389	0.53054028	profile #4
5430405H02Rik	0.265229209	0.183501249	0.437949452	profile #4
4930455G09Rik	0.2938704	0.280421948	3.190577514	profile #4
Zfp703	4.187192473	5.273412447	10.14581385	profile #4
Arhgap27os3	0.04678443	0.032368235	0.27832393	profile #4
Trp53cor1	0.063639592	0.052909959	0.202551562	profile #4
Cd63-ps	11.23336986	9.830972808	60.01229119	profile #4
Gm15328	0.21275575	0.129871961	0.429510295	profile #4
Gm15326	0.137669804	0.093040952	0.432507059	profile #4
A330040F15Rik	0.054961132	0.019012681	0.220924575	profile #4
Snhg12	2.5741941	2.546761396	5.345991431	profile #4
Gm15559	0.041619167	0.02410942	0.227083092	profile #4
Gt(ROSA)26So	2.785267243	2.423292322	5.323836928	profile #4
Chn1os3	0.027916193	0.017870544	0.124556577	profile #4
Gm15500	26.43535841	26.30789658	71.01305006	profile #4
Stamos	0.048823405	0.054993615	0.133534811	profile #4
Gm16023	0.1336739	0.143501123	0.293349818	profile #4

Mexis	0.293064086	0.207001101	3.700844111	profile #4
Gm16201	0.066711715	0.076390528	0.182460365	profile #4
Plxna4os1	0.118941979	0.13404084	1.817124184	profile #4
2410006H16Rik	13.224168	12.18715637	77.75875565	profile #4
A230103J11Rik	0.088235757	0.082863897	0.257298782	profile #4
AI662270	0.213926785	0.125919785	3.81709412	profile #4
Atg4a-ps	0.066708735	0.041764971	0.121325718	profile #4
A930001C03Ri	0.023050094	0.027135105	0.126086654	profile #4
Plcxd2	0.035763224	0.061050185	0.466037035	profile #4
5530601H04Rik	0.316646516	0.344894561	0.834099507	profile #4
Gm15133	0.055722344	0.052329925	0.456049574	profile #4
E230016M11Ri	0.120501401	0.091989496	0.21361118	profile #4
Gm14379	0.045322936	0.023517815	0.145630199	profile #4
4930404I05Rik	0.067421104	0.059527265	0.23239313	profile #4
Gm15501	17.06369381	15.7814185	40.39974926	profile #4
AV039307	0.132580177	0.17198776	0.426002359	profile #4
Gm16174	0.345407373	0.135540522	1.6794826	profile #4
Zfp111	0.337909948	0.241170816	0.801576876	profile #4
1500009L16Rik	0.637920332	0.723352189	1.65391639	profile #4
Gm25835	1.285683	0.613423012	3.223381969	profile #4
Gm15790	0.187968259	0.208369092	0.437239831	profile #4
Tnfsf13	0.649942531	0.432702974	1.058109708	profile #4
Galnt2	0.09938869	0.127247291	0.287417285	profile #4
B230216N24Ril	0.0395392	0.027927878	0.109603577	profile #4
Cbx6	7.792588029	7.183193219	15.65868487	profile #4
Cd300ld5	0.05519913	0.061190078	0.267501481	profile #4
Ier5l	5.521558862	3.835551414	21.91617802	profile #4
Gm16580	0.443338966	0.365336235	0.970045984	profile #4
Slc5a3	0.258715282	0.149786374	0.614800976	profile #4
Gm16118	0.062585643	0.064950672	0.251572445	profile #4
A930011G23Ri	0.603493361	0.604232631	1.641027125	profile #4
Shkbp1	1.660073071	1.635906811	6.284659623	profile #4
Tmem102	0.132100063	0.161822908	0.523915162	profile #4
Bcl2a1b	0.172691524	0.127622881	12.09210515	profile #4
Gm6493	0.368653538	0.29675111	0.612527468	profile #4
Galnt4	1.238352179	1.198599286	4.344152569	profile #4
Srpx	0.691612625	1.022234249	8.576238	profile #4
Kcne1l	0.220651909	0.236511748	1.302698531	profile #4
Ugt1a7c	0.100323993	0.051658962	2.657794287	profile #4
Pou3f1	0.077398716	0.068848725	0.165796709	profile #4
Gm10177	21.85422976	20.07861461	40.45556882	profile #4
Fbxw10	0.023368774	0.02707443	0.223133821	profile #4
Gm10612	0.027533961	0.032413617	0.135580763	profile #4
Tmem189	6.942816355	6.574241355	17.08558482	profile #4
Cfb	0.016383925	0.018454494	0.418235755	profile #4
Car15	0.146624033	0.103273087	0.233336874	profile #4
Eif4ebp3	0.252847626	0.292942419	0.51734656	profile #4
Mndal	7.501471265	5.453851341	12.11819196	profile #4
Snrpe	13.05905083	16.54632567	35.64600829	profile #4
A430057M04Ri	0.032277768	0.010331303	0.120999532	profile #4
Gm3571	0.93171965	0.624997031	1.806089247	profile #4
Tex9	0.115737774	0.110441242	0.222782093	profile #4
Samd15	0.11925847	0.0834811	0.237217487	profile #4
Myl6	49.75556904	47.67261108	210.8440737	profile #4
Pabpc4l	0.059790703	0.029687953	0.314588738	profile #4
Ccdc71l	7.154275881	6.676587986	16.351558	profile #4
Lrrc32	4.720623547	4.927273235	11.31217392	profile #4
Rpl6l	16.0265232	15.80179326	39.33865013	profile #4

Phf11c	0.319894235	0.255371245	0.544019048	profile #4
Gm5141	0.165694628	0.155607012	0.507949798	profile #4
Vgll3	0.599108153	0.564967514	6.229483022	profile #4
Gm6682	0.068773533	0.098439354	0.344849185	profile #4
Eid1	17.49015478	16.52501208	39.97610557	profile #4
Gcnt4	0.008366078	0.017364441	0.781867575	profile #4
Gm4202	2.273185769	1.988394373	5.699185583	profile #4
Gm10269	2.402481116	2.197068139	8.454772627	profile #4
Lsm5	4.191341513	3.686238656	11.93450514	profile #4
Gm3756	2.394868219	2.542423346	9.217370601	profile #4
Phf11b	0.862666964	1.012432751	15.20691698	profile #4
Gm9844	1.228485189	1.274908032	16.44377951	profile #4
Gbp11	0.017470155	0.019366251	0.067729944	profile #4
Peg10	0.112865537	0.185489844	0.87513549	profile #4
Bend4	0.133451886	0.11601728	0.723893752	profile #4
Gm4540	0.767383705	0.617712683	1.495360686	profile #4
Dynlt1a	0.286780572	0.238094107	5.061365268	profile #4
Fancf	1.012731967	0.731319214	2.210375339	profile #4
4930515G01Rik	0.037714241	0.041807495	0.342961633	profile #4
1110038B12Rik	1.127993027	0.96277928	7.226283035	profile #4
Smim40	0.052736353	0.054729191	0.784330861	profile #4
Gm3363	0.067961934	0.037669037	0.123604958	profile #4
Nlrp1c-ps	0.027208365	0.015761438	0.420621559	profile #4
BC051226	2.347441055	1.597567315	3.494775535	profile #4
Snord55	4.762497063	4.619463632	14.95589885	profile #4
Rab26os	0.856302553	0.54816187	3.011782789	profile #4
Mir5620	3.799842581	2.918961957	6.028142904	profile #4
Gm5873	0.279463094	0.204455121	0.475013137	profile #4
Rpl41	281.3323835	263.5263881	817.4489049	profile #4
Gm4705	2.125634283	1.914523467	5.557334797	profile #4
Zfp853	0.040660736	0.042197252	0.116133127	profile #4
Gm2237	0.142539549	0.084529104	0.632481014	profile #4
Rnasek	0.209195878	0.246270237	0.66956981	profile #4
Fsbp	0.019531086	0.01013457	0.078508152	profile #4
Gm9034	0.084693009	0.051874574	0.212772714	profile #4
D430019H16Ri	0.081281816	0.054062164	0.196950624	profile #4
Gm3604	0.313199146	0.306638428	0.651175035	profile #4
Rpl9-ps4	21.19989787	18.15477846	39.73888739	profile #4
Igha	0.035254617	0.020422522	0.411215146	profile #4
Itpripl2	9.749091662	9.730723579	30.33726312	profile #4
Rhox5	2.991494106	2.69658345	16.00575874	profile #4
Gm10130	1.264106047	1.20441461	3.063808604	profile #4
Gm1987	1.799726436	1.428268505	20.74533966	profile #4
Zfp748	0.606352008	0.429027751	1.284076943	profile #4
Gm17222	0.055524981	0.06432974	0.298916177	profile #4
Noc2l	9.678399184	9.179407395	18.61848811	profile #4
Rps7-ps3	50.05276491	55.42856411	129.1559792	profile #4
Gm21188	0.040493953	0.035752815	2.497566332	profile #4
Dynlt1f	1.573482998	1.938064933	8.416054041	profile #4
Sirpb1a	0.020202788	0.022755978	3.149579684	profile #4
Gm5451	1.039204912	1.035164401	2.204323088	profile #4
Vkorc1	2.0938266	1.314477882	9.266251715	profile #4
Ptrhd1	0.86955879	0.69240398	1.626048979	profile #4
Gm13238	0.054561842	0.056623663	0.109659784	profile #4
Dynlt1b	8.738626642	7.307540176	20.43698545	profile #4
LOC100041504	0.054244127	0.062845778	0.147983791	profile #4
Gm21992	0.044178101	0.038206278	0.125786222	profile #4
Zfp994	0.815671018	0.692256551	1.79856067	profile #4

Psmb9	4.01273346	3.782391808	10.42698696	profile #4
Lbhd1	0.323668448	0.317020047	0.845203866	profile #4
Zfp850	0.150201486	0.093051016	0.42450333	profile #4
5033403F01Rik	0.025362477	0.032471567	0.127435386	profile #4
Rps19-ps6	19.62731474	21.54393688	59.82725389	profile #4
Snhg18	3.408095687	3.043368142	13.1237523	profile #4
A230028O05Ri	0.030474928	0.037331863	0.107186255	profile #4
Gm16845	0.214706505	0.108324632	0.483310828	profile #4
Pvt1	0.066158645	0.09469652	0.458400804	profile #4
Gm4419	0.108801986	0.076633528	0.292865242	profile #4
Gm26760	0.381163068	0.373665953	0.773224534	profile #4
Gm26634	0.031124857	0.036060417	0.084911944	profile #4
Gm19705	0.181115501	0.133848387	1.191699624	profile #4
B230354K17Ril	1.503486825	1.07928673	2.576455605	profile #4
Gm38684	0.049859166	0.01444137	0.223678772	profile #4
Gm16982	0.032487204	0.041593351	0.11426382	profile #4
2700038G22Ril	0.261330798	0.170913707	0.873589091	profile #4
Snhg5	2.555368728	2.731682591	10.1703754	profile #4
4930525G20Ril	0.071239098	0.044358678	0.122081694	profile #4
E030030I06Rik	0.149358656	0.142523525	0.408504485	profile #4
Tnfsf12	4.687444511	4.198245213	11.168975	profile #4
F420014N23Ril	0.202714045	0.204286625	0.517980141	profile #4
Zfp87	0.888534848	0.524139453	2.577478205	profile #4
C030034L19Ril	0.048769089	0.036041443	0.125655784	profile #4
Mirg	0.035017748	0.064345244	0.607609028	profile #4
5830432E09Rik	0.062667561	0.085297688	0.79395534	profile #4
Gm16675	0.170916614	0.190044995	0.49073225	profile #4
E230029C05Ril	0.054251861	0.044373533	0.423948429	profile #4
Dleu2	0.726150542	0.616370995	1.350864566	profile #4
Gm10561	0.109516822	0.0841286	0.91492941	profile #4
4833412C05Rik	0.030507697	0.031660543	0.423482441	profile #4
5730420D15Ril	0.07957641	0.088213108	0.192818327	profile #4
5430416N02Ril	0.161733043	0.12806177	0.466436997	profile #4
Gm5976	0.128187461	0.10468664	0.214695359	profile #4
9330104G04Ril	0.1714244	0.125830361	0.341163771	profile #4
A930007I19Rik	0.108338306	0.138069613	0.403929956	profile #4
Gm26682	0.108801986	0.094541278	0.500903873	profile #4
Gm3650	0.061960627	0.05912511	0.122040836	profile #4
Gm9625	0.246228579	0.21836208	0.80608553	profile #4
4933404O12Ril	0.143414446	0.120782101	0.710985684	profile #4
Hdnr	0.241043537	0.171477577	0.418099842	profile #4
6720483E21Rik	0.041325525	0.039434336	0.152740413	profile #4
Ptprv	0.026924092	0.029846255	0.223456278	profile #4
2700099C18Rik	0.107816732	0.020250553	1.525678325	profile #4
Gm2076	0.043829286	0.053690985	0.093887235	profile #4
Bin2	0.454178813	0.581484912	14.36032304	profile #4
Gm5914	0.181508188	0.106837823	0.330956865	profile #4
Rps12-ps24	0.111701409	0.143011259	0.775272972	profile #4
Sowahc	0.577115399	0.814229422	3.427313491	profile #4
Gm7985	0.047181028	0.053143677	0.130786898	profile #4
Snhg6	9.358355759	8.461530617	21.01047006	profile #4
Mir6991	0.607974813	0.744770667	1.953525104	profile #4
Lockd	0.058822719	0.009413837	1.236422535	profile #4
Kctd12	2.228415386	2.659631754	8.091686262	profile #4
1500015A07Ril	0.757882127	0.569553553	1.192120296	profile #4
Mir6392	0.428561	0.408948674	1.688231571	profile #4
Zfp953	0.197924259	0.236083263	0.685689977	profile #4
Mir8102	0.338342867	0.317744294	2.125028793	profile #4

Gvin-ps4	0.013078326	0.012282107	0.046937709	profile #4
Tcf24	0.081198639	0.099468537	0.38758836	profile #4
Gvin-ps2	0.012881878	0.015164845	0.0469769	profile #4
Ms4a14	0.10469431	0.043460228	3.692823562	profile #4
Gm5525	0.143383105	0.083059893	0.321623478	profile #4
H3c4	0.306664637	0.348997853	0.675692559	profile #4
1700030C10Ril	0.171704702	0.188428668	0.443695493	profile #4
Rps10-ps2	103.207166	113.2021383	266.174805	profile #4
Gm8226	0.348004421	0.276732186	0.857490037	profile #4
Bcl2a1d	0.214528797	0.189411205	4.742884162	profile #4
1810006J02Rik	0.099956766	0.070403491	0.381163127	profile #4
Gm10640	0.036038052	0.033844028	0.196631336	profile #4
Dnah7c	0.010130502	0.012970076	0.028934224	profile #4
Ms4a4a	1.800178316	2.150814073	34.77719285	profile #4
Gm7901	0.086946627	0.110623561	0.428136372	profile #4
1600010M07Ril	0.027200497	0.030152659	0.568911659	profile #4
Gm5835	0.296249817	0.343227023	1.112637658	profile #4
9130401M01Ril	2.139086176	1.675903139	4.223111909	profile #4
Iqank1	0.08294729	0.077858343	0.163847373	profile #4
Gm20257	0.545618422	0.519050241	1.05090626	profile #4
Olfr1034	0.130760662	0.085430474	0.341647953	profile #4
Rpsa-ps1	0.19213651	0.117683853	0.666769757	profile #4
Pcdhga9	0.083806052	0.055997795	0.160779382	profile #4
Pcdhgc5	0.030831034	0.040535623	0.38130031	profile #4
Mannr	0.064373558	0.036254483	0.117376913	profile #4
Dchs2	0.02368074	0.009669655	0.194342805	profile #4
Pcdhgc3	1.222804406	1.185077894	2.963147344	profile #4
Zc3h11a	0.95163139	0.700190629	1.448313558	profile #4
Pcdhgb6	0.372520861	0.422948329	0.759055442	profile #4
Gm5845	0.058146467	0.065494908	0.214911134	profile #4
Gm7019	0.627162439	0.442986096	1.143550144	profile #4
Tstd1	0.907540941	0.713183847	1.682389939	profile #4
Pcdhga6	0.149048147	0.165224847	0.352403403	profile #4
Rps4x-ps	0.525297972	0.525941455	1.437019307	profile #4
Gm5869	0.101570971	0.082949796	0.54394096	profile #4
Snhg8	10.377624	11.49024851	24.06601582	profile #4
Rpl21-ps11	1.24128191	1.362182246	3.428506276	profile #4
Rpl36a-ps2	29.30021918	23.93366938	74.20995263	profile #4
Gm19710	0.329867344	0.218385931	0.557089281	profile #4
Gm6204	3.725009795	3.308281082	10.06449242	profile #4
Iglc1	0.101948432	0.120016057	0.401605748	profile #4
AI506816	2.755500938	2.362846881	22.2161324	profile #4
Gm4332	25.82553502	28.92982014	72.92692546	profile #4
Gm8013	0.184234792	0.191196783	0.49331442	profile #4
Gm43568	0.023079304	0.029548422	0.074157677	profile #4
D5Ertd605e	0.078738644	0.122571111	1.180668888	profile #4
Rpl7-ps7	0.405497016	0.44221734	1.711473808	profile #4
Dancr	0.374694151	0.338728685	1.147993559	profile #4
1110006O24Ril	0.421864734	0.496628944	1.225105395	profile #4
Gm9794	220.3715372	222.4628222	459.079422	profile #4
Gm4366	6.279084133	5.496123914	12.26931186	profile #4
Zfp862-ps	0.914706338	0.747011702	1.913683903	profile #4
C530044C16Ril	0.025983237	0.024401357	0.075550695	profile #4
6430584L05Rik	0.066310746	0.082579851	0.199909546	profile #4
Gm19187	0.047711028	0.049513965	0.10220236	profile #4
Snhg1	2.276035652	2.147191955	6.409850841	profile #4
Gm17825	0.533081771	0.393959295	0.807946768	profile #4
Gm9333	0.353847775	0.433986348	0.808150333	profile #4

Gm7972	0.014298606	0.016105637	0.039636066	profile #4
E230032D23Ril	0.025063744	0.032089099	0.198808011	profile #4
Nup62	8.141157386	9.049395723	17.2810081	profile #4
Gm7432	0.107007187	0.118621039	0.259284712	profile #4
5830408C22Ril	0.078965606	0.074158119	0.215426632	profile #4
Tincr	0.268294638	0.208824855	0.466957669	profile #4
Gm5086	0.08583141	0.08190349	2.081858201	profile #4
Gm5905	3.448684715	3.05612791	7.348098332	profile #4
Gm45871	0.88736941	0.782661418	1.794868237	profile #4
Gm45734	0.228854786	0.253693171	0.718683704	profile #4
Gm39822	0.093166018	0.091302884	0.430669115	profile #4
Rpl10-ps5	2.051137754	1.307106978	7.295743047	profile #4
Gm8162	0.139201768	0.081935774	0.479813183	profile #4
Rpl26-ps6	0.294181361	0.407064118	1.281048624	profile #4
Gm8942	0.705444878	0.681091123	2.215342517	profile #4
Gm5176	0.03832898	0.035995482	0.104565564	profile #4
Lilr4b	0.672271089	0.665866788	22.08296289	profile #4
A130077B15Ril	0.018172198	0.042785465	0.911203384	profile #4
BC024063	0.356557721	0.27983201	0.619178288	profile #4
Gm46209	0.340178362	0.282824304	1.082716851	profile #4
4933412E12Rik	0.579658897	0.604419403	1.563800776	profile #4
Gm32687	0.175099324	0.068710328	0.48537937	profile #4
4930455C13Ril	0.138594278	0.086771035	0.294077809	profile #4
Gm20337	0.076132803	0.048736395	0.306027303	profile #4
Gm7392	1.603643709	1.579341735	4.280855105	profile #4
D430020J02Rik	0.175901477	0.075087482	1.032464947	profile #4
Rps18-ps5	7.922703081	7.214582411	20.78477447	profile #4
Gm4811	0.192744502	0.144808055	0.380464757	profile #4
Zfp935	0.534407084	0.403346638	1.075380384	profile #4
Dio3os	0.177009898	0.123907182	0.487510721	profile #4
5033406O09Ril	0.178902328	0.097551534	1.610858175	profile #4
Gm6566	0.200809814	0.222604384	0.46845517	profile #4
Rpl17-ps3	98.47961801	98.39071766	243.142958	profile #4
Scamp4	0.039109903	0.021677317	0.132671837	profile #4
Gm9616	20.11255562	21.20888107	47.92412201	profile #4
H2bc9	0.137833794	0.097356682	0.254718973	profile #4
Gm3226	0.137957043	0.162406229	0.56598063	profile #4
Nupl1	1.539430916	1.345471326	2.760613294	profile #4
Gm46432	0.266822802	0.3268586	0.793673987	profile #4
Slfn4	0.053680924	0.249885214	7.530672627	profile #7
Ckmt1	0.03708701	0.053084684	0.091310602	profile #7
Lck	0.079010905	0.211106315	0.686262832	profile #7
Haao	0.07575219	0.180713817	1.119929705	profile #7
Abhd15	0.21033725	0.34925699	1.310497645	profile #7
Tcf7	0.193954464	0.302855704	0.863492468	profile #7
Ccl3	0.245585684	2.041799479	7.854905117	profile #7
Itgb7	0.217213021	0.532172989	5.251181832	profile #7
Ell2	1.239385587	1.580064877	2.894111265	profile #7
Angptl4	0.429363894	1.276786768	17.8624551	profile #7
Ccdc65	0.0489784	0.110336395	0.200938097	profile #7
Cd79a	0.484176306	0.860128804	1.439110234	profile #7
Rab3b	0.040036599	0.075198281	0.145632176	profile #7
Has1	0.090008916	0.21132276	0.937816677	profile #7
Il7r	0.03979885	0.146261072	4.248470534	profile #7
Klf5	0.049457568	0.121171288	0.323053517	profile #7
Fgfr4	0.024030625	0.037408066	0.051476287	profile #7
Dnajb1	5.768619161	8.80289175	15.22463557	profile #7
Itih3	0.016706806	0.037040104	0.239840854	profile #7

Zfp184	0.066936605	0.093061127	0.158262198	profile #7
Rad51c	0.171641089	0.298288083	0.790180786	profile #7
Id3	42.02051374	56.95153915	106.5645072	profile #7
4930432K21Rik	0.088441889	0.130009842	0.227342905	profile #7
Spib	0.098388407	0.153997409	0.46090841	profile #7
Nuak2	0.850125086	1.397102206	4.825672131	profile #7
Wnt2	0.056618497	0.12081439	0.208621052	profile #7
Pax5	0.06249374	0.098474713	0.133868583	profile #7
Trpv4	1.488426385	2.005175044	3.168726525	profile #7
Kif1a	0.100351684	0.146000241	0.298019014	profile #7
Naf1	1.989608987	2.64545237	4.272415385	profile #7
Slamf6	0.004341734	0.012232217	0.116867615	profile #7
Anxa9	0.133964075	0.236558422	0.875287513	profile #7
Eps8	0.890727648	1.314221965	3.586074412	profile #7
H3f3b	43.90066913	62.12210164	139.5223398	profile #7
Slpi	0.14426521	1.04801762	8.662193585	profile #7
Sdc4	6.900884115	9.862049715	21.8454172	profile #7
Hspb9	0.064779144	0.24334136	0.69747108	profile #7
Rac3	0.930703239	1.400483164	3.605288013	profile #7
Ikzf3	0.009723152	0.050452886	0.131907416	profile #7
Shroom1	0.132909904	0.248746323	0.504529352	profile #7
Slc13a3	0.032642943	0.076222073	0.340883575	profile #7
Wnt9b	0.104093976	0.156410648	0.383383265	profile #7
Dusp14	0.072539319	0.158953829	0.492147713	profile #7
Slc35g3	0.026302989	0.032221228	0.05286442	profile #7
Ccl4	0.071257058	1.155790997	7.113593939	profile #7
Esr1	0.024098354	0.042029422	0.179154236	profile #7
Rgs17	0.12271161	0.229821059	0.427156909	profile #7
Wasf1	0.081087225	0.185703392	0.413985988	profile #7
Tnfaip3	0.589286202	1.075739884	1.791497327	profile #7
Nts	1.055152595	1.734099668	5.883235622	profile #7
Dusp6	6.441801883	11.96816976	16.03819993	profile #7
Myb	0.00476416	0.009888382	0.023618605	profile #7
Ccn2	10.47221209	30.13965322	114.4893624	profile #7
Peli1	1.808556988	2.487017026	3.824968441	profile #7
Mdm1	0.764662776	0.937296418	1.616863181	profile #7
Nsg2	0.02262673	0.062706214	0.10288024	profile #7
Rgs9	0.007359884	0.016317354	0.033464253	profile #7
Pomc	0.280938319	0.372067988	0.616524277	profile #7
Cacng4	0.012946738	0.0457236	0.114752524	profile #7
Slc6a4	0.037570723	0.084178113	0.251502165	profile #7
Lrrc46	0.063709935	0.099176176	0.157922905	profile #7
Npas3	0.007084774	0.022057495	0.045529135	profile #7
4930447C04Rik	0.007738604	0.027330191	0.063496573	profile #7
Plek2	0.134422732	0.206521629	0.453157931	profile #7
Gcnt2	1.339108772	1.746437031	3.225860723	profile #7
Mak	0.006194634	0.020600875	0.072255138	profile #7
Edn1	2.531695557	3.265988246	6.435642932	profile #7
Adcy2	0.04467315	0.074282517	0.260537167	profile #7
Ddx4	0.062414942	0.086486308	0.14189554	profile #7
Fst	0.041816531	0.091631653	0.567413931	profile #7
Cldn10	0.01642541	0.060363504	0.158332795	profile #7
Jph4	0.027374656	0.044989115	0.059697362	profile #7
Ctnnd2	0.023856776	0.059744953	0.282029981	profile #7
Myc	0.761613903	2.065583679	7.885041825	profile #7
Gcsam	0.012429392	0.030452072	0.184858354	profile #7
Ypel1	0.119365066	0.171520221	0.341400362	profile #7
Osbpl11	5.046728978	6.545707609	12.39393545	profile #7

Muc13	0.014008636	0.029076008	0.041669191	profile #7
Thpo	0.076804015	0.120213546	0.223320779	profile #7
Cela1	0.04263795	0.197596699	10.35255963	profile #7
Cdkn1a	7.349734398	23.43223082	48.01944209	profile #7
Nus1	12.2719676	15.80808872	26.89793075	profile #7
1700010I14Rik	0.020017985	0.041548878	0.23817686	profile #7
Mmp25	0.041828928	0.080769774	0.364874241	profile #7
Efhb	0.017673679	0.039183725	0.06343029	profile #7
Pim1	9.647394112	13.6734594	21.56237924	profile #7
Cbs	0.012730552	0.04889676	0.140724042	profile #7
Tpsab1	0.022459245	0.049793645	0.081694968	profile #7
Tslp	0.581125524	0.769458639	1.37801325	profile #7
Ptpn2	3.100195088	4.187398599	7.132710859	profile #7
Slc27a6	0.051378153	0.104897277	0.533515808	profile #7
Cd5	0.022730623	0.064040295	0.289387346	profile #7
Cyp26a1	0.02652547	0.058808739	0.30939707	profile #7
Cd7	0.040851357	0.144273495	1.11730804	profile #7
Ribc1	0.179513593	0.228397967	0.436426636	profile #7
Sat1	9.375414979	17.54557874	60.35834196	profile #7
Baiap2	1.196467674	1.899196401	5.534392204	profile #7
Nptx1	0.009014694	0.016931745	0.040988418	profile #7
Atrip	0.02601232	0.039066277	0.104154197	profile #7
Gria4	0.007340934	0.016537334	0.040698476	profile #7
Tmeff2	0.042072156	0.081239436	0.178542543	profile #7
Cxcr2	0.061718712	0.171043132	1.749712445	profile #7
Rgs18	0.125725958	0.375589393	2.463700604	profile #7
Rgs1	0.019987587	0.108079713	0.570844388	profile #7
Slc45a3	1.069155863	1.758641127	2.285720197	profile #7
Rgs16	0.395763899	0.86860069	4.17041557	profile #7
F5	0.040095243	0.073334172	0.214721243	profile #7
Sele	0.555361608	0.954276024	1.409911504	profile #7
Cenpf	0.633847532	0.977967945	1.727831119	profile #7
Slc25a25	0.94513941	1.261344029	2.023587052	profile #7
Lcn2	1.039923825	1.578739622	3.571381652	profile #7
Grin1	0.004387447	0.005617246	0.008818003	profile #7
Itpka	0.050918139	0.121469903	0.211719384	profile #7
Rasgrp1	0.030398741	0.068109112	0.542532861	profile #7
Dusp2	0.138645795	0.468502766	1.547475369	profile #7
Acoxl	0.01455948	0.034279515	0.066368355	profile #7
Il1b	0.245485373	0.800023134	6.764434943	profile #7
Il1a	0.023447106	0.055204954	0.281648988	profile #7
Ccnl1	4.272145842	5.339449926	8.964234738	profile #7
Ptx3	0.17454907	0.711057101	7.036186616	profile #7
Sass6	0.128547296	0.212930676	0.628828362	profile #7
Sh2d2a	0.00779333	0.018348978	0.100815359	profile #7
Celf3	0.034372124	0.049778266	0.08328568	profile #7
Coro2a	0.064493156	0.218815083	2.756949085	profile #7
Plk3	1.57268849	2.45418848	5.551798146	profile #7
Csf3r	0.205196289	0.844412724	8.628813034	profile #7
Sema3a	0.071215952	0.116538243	0.243228056	profile #7
Tas1r1	0.016426706	0.038063074	0.059751751	profile #7
Angptl7	0.959343984	1.945072471	2.557001643	profile #7
Tnfrsf4	0.101724369	0.22649465	0.595295968	profile #7
Kcnip4	0.035201189	0.060090753	0.106122371	profile #7
Fosl2	2.189911292	5.385738296	11.8005294	profile #7
Nos1	0.008301424	0.023376355	0.03678955	profile #7
Ppbp	0.897485086	1.877399743	8.417775147	profile #7
Rasl11a	0.587381247	1.103242053	3.169268184	profile #7

Nfe2l3	0.015568311	0.044567565	0.229981092	profile #7
Rab43	0.27233968	0.433847404	1.076687848	profile #7
A2m	0.024246254	0.037743732	0.091370137	profile #7
Rasgef1a	0.056071128	0.186470152	0.301856336	profile #7
Clec4e	0.009322173	0.076819945	2.010576693	profile #7
Cd69	0.019615104	0.058597485	0.280118313	profile #7
Klrd1	0.036713238	0.103434325	1.402207069	profile #7
Klra13-ps	0.040882072	0.084853904	0.324280502	profile #7
Klra2	0.048771958	0.172246438	1.633083859	profile #7
Klrb1c	0.031766065	0.055204954	0.169336297	profile #7
Pglyrp1	0.115836246	0.34055926	0.954523647	profile #7
Lilra6	0.015175275	0.051279292	0.433405023	profile #7
Siglecg	0.026565691	0.068024082	0.169075946	profile #7
Furin	9.930647001	12.71053471	20.80118728	profile #7
Lrfn1	0.084002962	0.208550484	0.387622356	profile #7
Qprt	0.035561178	0.066792373	0.17548847	profile #7
Cd19	0.10536329	0.201083056	0.370587418	profile #7
Hpx	0.028391085	0.058927894	0.085591692	profile #7
Zfp300	0.039863467	0.091866918	0.213480013	profile #7
Smarca1	0.187818306	0.353735924	1.103550188	profile #7
Sytl4	0.290641618	0.40400332	0.842829049	profile #7
Col4a6	0.116103319	0.176547919	0.380845279	profile #7
Gabre	0.146593923	0.251793567	1.00235896	profile #7
Xlr4c	0.020736823	0.046715006	0.249092788	profile #7
Mt2	0.506645677	1.204650966	2.942307846	profile #7
1700030J22Rik	0.047092448	0.117934464	0.295773367	profile #7
Hsd11b2	0.025559597	0.048007018	0.209187957	profile #7
Smpd3	0.382308321	0.607795278	0.798452414	profile #7
Trpc6	0.025202272	0.061027477	0.15195651	profile #7
Cd3e	0.022688524	0.043300448	0.093081618	profile #7
Cd3d	0.02550115	0.090061636	0.212069334	profile #7
Rab27a	0.957809493	1.810380675	2.991537791	profile #7
Calml4	0.038739422	0.085887886	0.422741176	profile #7
Tent5a	1.801714631	3.484991334	9.515227497	profile #7
Stra6	0.016136441	0.030308085	0.072391491	profile #7
Dclk3	0.084974849	0.235162499	0.384265398	profile #7
Ttc21a	0.00859031	0.024591568	0.050759747	profile #7
Mctp2	0.007494873	0.013234707	0.051247268	profile #7
Pdxk	2.075388234	2.806645651	5.546992945	profile #7
Styk1	0.00789166	0.015061025	0.06994719	profile #7
Shf	2.008008157	2.555807366	4.448534091	profile #7
Stk36	0.026446007	0.072891956	0.318911162	profile #7
Rpgrip1l	0.188912708	0.255965618	0.583432969	profile #7
Trim69	0.024909777	0.057719587	0.096572569	profile #7
Tagap	0.166026932	0.584551549	1.83526842	profile #7
Asprv1	0.089756796	0.199848221	0.497615825	profile #7
Egr3	0.30909296	1.533459814	3.581738416	profile #7
Ephx4	0.025855551	0.031673115	0.051965147	profile #7
Tpsb2	0.590539828	0.857997774	1.433669364	profile #7
Ypel4	0.325617727	0.422402992	0.834404398	profile #7
Rnf43	0.007171342	0.015899343	0.03260698	profile #7
Ly6d	0.18963572	0.366178023	0.977210168	profile #7
Itga2b	0.398858753	0.630883844	0.917450665	profile #7
Fras1	0.014419189	0.019952095	0.047655994	profile #7
Dusp5	1.893006898	3.20435095	5.188109455	profile #7
B3galt1	0.204370148	0.317171586	0.530855365	profile #7
Celf5	0.014106076	0.024514378	0.068359707	profile #7
Rtkn	0.639679244	0.855153062	1.422571754	profile #7

Zc3h12b	0.058284438	0.094972866	0.340775423	profile #7
Pdzrn3	2.579631213	3.889803769	6.922773177	profile #7
Ccl7	2.348547241	5.918095153	55.71164185	profile #7
Ccl2	1.757677623	5.355862077	52.53840705	profile #7
Slc12a8	0.105827314	0.159015146	0.259837426	profile #7
Fbxl21	0.019616125	0.03684376	0.142593073	profile #7
Slc51a	0.058787073	0.102163668	0.158238751	profile #7
Zfp983	0.612099677	0.862251869	1.680907356	profile #7
Myrf	0.241684767	0.407286446	1.048785766	profile #7
Fam110c	0.015229812	0.03376551	0.204972476	profile #7
Elf1	3.336696076	4.509031636	9.683595346	profile #7
Greb1	0.019636601	0.036882219	0.057261065	profile #7
Slitrk2	0.015801326	0.027330728	0.105859692	profile #7
Nfkbid	0.21702066	0.394835434	1.744718257	profile #7
Lrfn3	0.457139759	0.632255071	1.231952768	profile #7
Trim17	0.022782782	0.047287427	0.114473543	profile #7
Prf1	0.054600997	0.102553692	0.277611844	profile #7
Cnnm4	0.785946618	1.026784713	1.784022719	profile #7
Serpine1	6.006109638	19.05928857	60.32945174	profile #7
H2-DMb2	0.189469074	0.701216827	2.601053113	profile #7
Gpr160	0.394190955	0.605844008	1.456941909	profile #7
Egr2	0.096882757	1.395054214	6.531966501	profile #7
Bank1	0.081670526	0.15321224	0.253655492	profile #7
Ccr7	0.093598762	0.237212093	1.145939195	profile #7
Dlec1	0.00635577	0.013191893	0.038321967	profile #7
Crygn	0.371455933	0.543514113	0.789220704	profile #7
Cd160	0.017800779	0.059198274	0.095829675	profile #7
1700003F12Rik	0.094845467	0.316767621	0.513734402	profile #7
Cfap157	0.260171939	0.351662372	0.673122449	profile #7
Lgi2	0.273812186	0.450722067	1.428544282	profile #7
Hlx	4.97548187	6.204243279	10.83263438	profile #7
Grin3a	0.007662673	0.012741494	0.022280531	profile #7
Sdk1	0.07677142	0.13544327	0.43007676	profile #7
Mcph1	0.444702037	0.593315352	1.109483469	profile #7
Slc44a3	0.009152578	0.032323882	0.090136944	profile #7
Fgl2	9.361236143	14.58556281	32.44185653	profile #7
Ptger4	0.723387601	1.380200755	2.516348772	profile #7
Stk32a	0.086053683	0.180010971	0.403477294	profile #7
Ccdc40	0.00705658	0.029292958	0.075579924	profile #7
Thbs1	0.914564558	4.512943652	54.94939404	profile #7
Il7	0.049030284	0.073635518	0.149000707	profile #7
Apoc1	0.062210468	0.210217527	0.804761315	profile #7
Cd79b	0.427243578	1.028062073	2.839199848	profile #7
Dlk1	0.008181826	0.018431659	0.044755524	profile #7
Atp1a3	0.031073909	0.140846371	4.072385899	profile #7
Cldn5	15.89828625	27.1730684	42.82368845	profile #7
Rpl21	9.488340542	17.92638542	30.36942935	profile #7
Sdk2	0.068779877	0.122771843	0.165882664	profile #7
Enc1	1.8760061	4.739025422	8.115150369	profile #7
Sytl3	0.010691751	0.020404923	0.052647554	profile #7
Zfp239	0.021212002	0.050603182	0.229791373	profile #7
Trem1	0.014205019	0.162159888	1.704009436	profile #7
Fcmr	0.108363268	0.188320126	0.369533257	profile #7
Muc1	0.060140228	0.162579296	0.641182579	profile #7
Lor	0.237924073	0.336908481	0.649082362	profile #7
Tox3	0.418480875	0.631701409	0.869268223	profile #7
Cilp2	0.065409817	0.096678622	0.137359592	profile #7
Rell2	0.014080736	0.031217932	0.113704492	profile #7

Efcab9	0.045090756	0.084691192	0.262218135	profile #7
Ctla2a	3.425235166	6.331257285	14.77295362	profile #7
Cdkn2a	0.039491969	0.177931559	1.516815567	profile #7
Dock5	0.86732211	1.199983046	1.838056214	profile #7
Phf11a	0.03143149	0.120725162	2.811145561	profile #7
Wdr72	0.025129393	0.036392793	0.051416757	profile #7
Kcng4	0.012160953	0.046417716	0.167669872	profile #7
Tnfrsf26	0.116916491	0.219597051	1.062358276	profile #7
Olfr1033	3.016331271	4.398880007	7.674882716	profile #7
Ttll13	0.043535902	0.072391502	0.097655636	profile #7
Pcdhb3	0.078665872	0.100715878	0.180691061	profile #7
Fpr1	0.032143683	0.078752185	0.828642	profile #7
Ptprcap	0.558064988	0.899830795	1.95735816	profile #7
Pcdhb8	0.048861351	0.062557164	0.11047814	profile #7
Nexmif	0.020390938	0.028349266	0.051920085	profile #7
Tmem215	0.029759328	0.076974821	0.169108417	profile #7
Gm5424	1.510130987	1.976044754	3.811341965	profile #7
Tent5b	0.39071426	1.062157904	2.435506796	profile #7
Nxph3	0.040550964	0.103834759	0.201033827	profile #7
Gprc5a	0.422182992	0.763318505	1.195210283	profile #7
Lrrc75b	0.122606105	0.211093211	0.315900224	profile #7
Dmrta2	0.015840037	0.071367484	0.395180534	profile #7
Kcne4	1.329444359	3.014539559	7.428345991	profile #7
Gpr141b	0.016745509	0.031958362	0.091599856	profile #7
Ust	0.528651587	0.812715737	2.107857319	profile #7
Cxcr5	0.05109032	0.098116368	0.177841921	profile #7
Zbtb33	1.554265743	1.946734487	3.431738914	profile #7
Arxes1	0.029294043	0.055906907	0.288495268	profile #7
Jaml	0.060527231	0.157781727	1.018269419	profile #7
Gm9828	0.072404518	0.10194358	0.200706995	profile #7
Gm6377	0.033652707	0.189623428	1.615412893	profile #7
Tprn	1.451508762	1.884787978	3.267352898	profile #7
Prss50	0.031788865	0.074845178	0.289814563	profile #7
Syt12	0.322701923	0.494370884	0.76943438	profile #7
Lingo1	0.239387445	0.346684771	0.510122804	profile #7
Stkld1	0.019424115	0.049737371	0.078078166	profile #7
Fhl4	0.079397285	0.111845276	0.178096512	profile #7
Ccdc158	0.006856491	0.016798436	0.027193169	profile #7
Gpr171	0.019127297	0.099250465	1.081001806	profile #7
Slc9b1	0.012546649	0.023944948	0.057193007	profile #7
Zfp831	0.009439092	0.021263973	0.051127233	profile #7
Gp1bb	0.098425493	0.376573508	1.503029783	profile #7
Tmem125	0.020106737	0.044577979	0.091422222	profile #7
P2ry10	0.057086837	0.127904436	0.702749318	profile #7
Flrt3	0.061234873	0.099862492	0.206127349	profile #7
Hspa14	0.248834172	0.365767759	0.777229798	profile #7
Lax1	0.06110393	0.141586721	0.328950197	profile #7
Btla	0.142108038	0.267668602	0.92368089	profile #7
Bmpr1b	0.01577647	0.043721861	0.071733169	profile #7
Epha3	0.008310595	0.02341392	0.112767544	profile #7
H1f3	0.034390494	0.051368489	0.109438249	profile #7
Klrc2	0.007322477	0.017940106	0.03137113	profile #7
Trim30b	0.012496256	0.057911295	0.398032021	profile #7
Gas1	6.893235998	10.91855671	21.5930891	profile #7
Tnfaip6	0.671972162	1.213931855	5.1770961	profile #7
Lipg	0.012221967	0.027533112	0.189424084	profile #7
Cd8a	0.025452069	0.072861903	0.22044075	profile #7
Pnma1	0.099681345	0.168502803	0.357753288	profile #7

Rnd1	0.873588449	1.946630511	3.68713872	profile #7
Txk	0.004224974	0.009517836	0.020804314	profile #7
Rbm3os	0.131487859	0.227427687	0.308312265	profile #7
A230083G16Ri	0.233524662	0.356540519	0.468382446	profile #7
Prr18	0.011981486	0.0248685	0.084282435	profile #7
Elapor2	0.00584549	0.011155963	0.032407665	profile #7
S100a8	0.674554187	6.202443658	21.21479472	profile #7
S100a9	1.773355862	10.48671342	32.01151749	profile #7
AI504432	0.045851934	0.127192453	1.055864709	profile #7
Gpr173	0.063415955	0.127979542	0.499629308	profile #7
Nfil3	4.689038014	6.750399681	9.584422276	profile #7
Gm6139	0.144755908	0.250376739	0.43640118	profile #7
Rpl15-ps6	0.152941979	0.359076885	0.764943856	profile #7
Phex	0.00735727	0.014041156	0.020122556	profile #7
Rps18-ps3	1.9285245	2.421406625	4.501822696	profile #7
Adamts17	0.115111151	0.168430571	0.242370155	profile #7
Kcnh1	0.017415729	0.066892049	0.116675257	profile #7
Gm8369	0.023196419	0.04551227	0.100023792	profile #7
Osm	0.061115213	0.311031386	1.544821055	profile #7
Nfe2	0.015490157	0.118250219	0.372138623	profile #7
Slc14a1	0.085223003	0.121609769	0.239606333	profile #7
Akap17b	0.411190695	0.521078928	0.979528663	profile #7
4930473A02Ril	0.098547739	0.185003359	0.258806259	profile #7
H4c3	0.05644462	0.127155968	0.500293014	profile #7
Spry3	0.010813902	0.030466629	0.083848705	profile #7
Olfr99	0.026450877	0.049681054	0.094931384	profile #7
Lmtk3	0.016375229	0.028457846	0.096792323	profile #7
Stat4	0.012146089	0.080786074	0.272449641	profile #7
3300002I08Rik	0.111076189	0.250353086	0.438513938	profile #7
Wscd2	0.003067418	0.011522686	0.044595212	profile #7
D630045J12Rik	0.065383433	0.108531636	0.305011354	profile #7
Ipcef1	0.018908469	0.039245991	0.199514301	profile #7
Aox3	0.032905295	0.051889326	0.097217942	profile #7
Snora41	0.356285292	0.66918874	3.107880847	profile #7
Snora31	4.341199246	6.706127983	14.80407079	profile #7
Snord49a	0.653189701	2.270303744	5.86069449	profile #7
Ifi208	0.008802698	0.016799717	0.024401283	profile #7
H2-Q10	0.068516021	0.172683939	0.601705156	profile #7
C730014E05Ril	0.019538703	0.036698344	0.052592855	profile #7
Col28a1	0.396401447	0.567391269	0.975965845	profile #7
H2bc11	0.099966713	0.235366263	0.461850061	profile #7
Prdm6	0.016245132	0.030512233	0.125961319	profile #7
Mmp27	0.020943252	0.079939288	1.915820925	profile #7
Treml2	0.041059737	0.157093539	2.199222683	profile #7
Rps8-ps1	0.409213816	0.698554998	2.410599888	profile #7
Hpcal1	10.2399531	13.36719313	25.34309518	profile #7
2410004P03Rik	0.039257496	0.062434912	0.141759903	profile #7
Klrg2	0.109946601	0.180692693	0.449919159	profile #7
Snx29	0.449721851	0.64516658	1.35294642	profile #7
Ang	2.290525394	3.608257275	10.24668599	profile #7
G530011O06Ri	0.019760361	0.068681458	0.395328127	profile #7
Uprt	1.061433265	1.667743813	2.877141458	profile #7
9030624G23Ril	0.020948623	0.046444496	0.244347886	profile #7
Wdr90	0.277756074	0.412588522	1.156355312	profile #7
F830208F22Rik	0.015775252	0.035537796	0.09321524	profile #7
Mob3b	0.102963476	0.156719686	0.448470548	profile #7
Osgin1	1.090440848	1.754695289	2.271113993	profile #7
Adh6b	0.030218243	0.046680051	0.068339209	profile #7

Gm12522	0.018976871	0.036216857	0.149511283	profile #7
Fat3	0.02585972	0.043691733	0.062869169	profile #7
Slc4a11	0.018521244	0.032705456	0.119796402	profile #7
Ctla2b	0.214902324	0.347858496	0.521249929	profile #7
Scn2a	0.022162893	0.031220398	0.077257953	profile #7
Smim41	1.576546719	2.963890342	3.861443853	profile #7
Gm10863	0.069463934	0.148224401	0.344371602	profile #7
Trbc2	0.184501027	0.527366513	2.107849823	profile #7
Igkc	1.768032274	2.573609327	7.074292808	profile #7
Gm26387	0.356285292	0.66918874	0.971984446	profile #7
Gm20900	0.180331512	0.34631752	0.80460382	profile #7
Gvin2	0.254502856	0.42297411	0.555341728	profile #7
Cntd1	0.028879147	0.04716937	0.114536079	profile #7
Il11ra2	0.032762307	0.083891131	0.129936876	profile #7
Zfp973	0.260407854	0.461873282	0.872289412	profile #7
Gm14391	0.006012485	0.020897708	0.038272975	profile #7
Fam186b	0.013353698	0.027716634	0.053677208	profile #7
H2-Q1	0.014880502	0.046328451	0.104675248	profile #7
Rbpj-ps3	0.130547282	0.367798391	0.979404174	profile #7
Rpl28-ps3	3.949055294	5.169610724	10.4150357	profile #7
Gm25777	1.119753774	1.655045927	2.532347357	profile #7
Rpsa-ps12	0.21089533	0.366506434	1.175327136	profile #7
Rpl10-ps6	0.530320709	0.756746145	1.446003126	profile #7
Rpl19-ps11	10.18578052	13.00332232	23.06107187	profile #7
Gm18859	0.057005647	0.126385325	0.409183033	profile #7
Gm8864	0.229131624	0.32368687	0.508126522	profile #7
Rpl17-ps10	109.1944673	158.1866059	307.6199339	profile #7
Gm12435	0.318889878	0.478180143	1.162909458	profile #7
Gm13408	0.812010316	1.229036613	3.48997747	profile #7
Rpl5-ps2	0.052783006	0.099139073	0.335994623	profile #7
Gm7429	0.270285394	0.380745318	0.614472925	profile #7
Rps6-ps3	0.246851136	0.347559645	0.729316039	profile #7
Gm12749	0.08115558	0.134945505	0.393289495	profile #7
Gm14584	1.16256422	1.594707233	2.790750148	profile #7
Gm14400	0.0199336	0.025520979	0.040063059	profile #7
Gm6451	0.322765607	0.436267336	0.835066135	profile #7
3010001F23Rik	0.025897389	0.048641472	0.092944935	profile #7
Crocc2	0.005967223	0.012385434	0.083951545	profile #7
Mir22hg	6.03866875	9.700599231	14.33727576	profile #7
Gm10785	0.087715581	0.206521629	0.432265982	profile #7
D630024D03Ri	0.060025091	0.112741434	0.261799791	profile #7
Rtl1	0.014793853	0.03279896	0.080718431	profile #7
Zfp652os	0.237755513	0.388334799	0.848831516	profile #7
Has2os	0.057775993	0.096069926	0.363986045	profile #7
Cd101	0.051121005	0.156558399	1.067692399	profile #7
Gm6286	0.167552114	0.289806147	0.505125775	profile #7
Gm13889	4.056551535	7.963922893	19.81125195	profile #7
Kantr	0.342874379	0.46039565	0.788217373	profile #7
Cd55os	0.057870786	0.128303396	0.192961637	profile #7
Gm25492	0.364570996	0.808278244	1.76675397	profile #7
Gm24727	0.326594851	0.724082593	3.165434196	profile #7
Mir1964	0.512749842	0.62811984	1.030537726	profile #7
A230009B12Ril	0.015068779	0.034916559	0.054081385	profile #7
Gm16184	0.445635989	0.636889401	1.044925687	profile #7
BC035044	0.026867574	0.139414321	0.546756816	profile #7
Hspa1b	0.924701009	2.231920658	4.24529222	profile #7
Catspere2	0.062598899	0.082886161	0.170776503	profile #7
Rpph1	0.133411401	0.3268586	3.217603682	profile #7

Edaradd	0.0138826	0.023455585	0.064138146	profile #7
Gm20939	0.058886244	0.103983378	0.435290744	profile #7
Rps2-ps6	0.211845309	0.331580006	0.963227829	profile #7
Rps19-ps4	0.10737365	0.238054551	0.520345347	profile #7
Erdr1	0.700033804	1.925406037	5.030603242	profile #7
1700113A16Ril	0.171797839	0.298560492	0.385360734	profile #7
4933433G15Ril	0.046896129	0.073002408	0.11624518	profile #7
4930539J05Rik	0.127572653	0.211829529	0.409912342	profile #7
1700109K24Ril	0.132874415	0.295851864	0.572959252	profile #7
Gm16617	0.06596025	0.14623828	0.414276301	profile #7
A330074K22Ri	0.056685004	0.08378296	0.240555411	profile #7
Mroh6	0.020886547	0.070578448	0.114251806	profile #7
Gm27219	0.126764578	0.220299016	0.284346363	profile #7
Gm8451	2.314658808	3.557030081	9.156843471	profile #7
Gm6501	0.252645131	0.475871527	0.700338138	profile #7
Ppp1ccb	4.523316404	6.132607658	9.203510721	profile #7
Gm8292	1.425141167	2.106422089	3.13194988	profile #7
Hoxd4	0.014436308	0.036965597	0.058028922	profile #7
Naaladl2	0.016351258	0.025453726	0.048637403	profile #7
Gm5841	0.02126072	0.040575523	0.062814312	profile #7
Gm5846	0.798240314	0.999523776	1.701010621	profile #7
Mir703	2.342655242	3.999067146	10.20137804	profile #7
Tsg101-ps	0.079118954	0.178235715	0.252460686	profile #7
Pagr1a	0.018881205	0.023129524	0.037947929	profile #7
Gm5586	0.23287843	0.342687358	0.699339752	profile #7
Gm10060	4.660596788	10.30840619	16.56751866	profile #7
Rpl32l	0.464005895	0.664157246	1.028171107	profile #7
Gm9182	0.14486569	0.414708515	0.642003555	profile #7
Gm7868	0.069161263	0.153335137	0.268130897	profile #7
Gm3379	0.793038112	1.299288391	2.464782987	profile #7
Gm7240	0.124420936	0.186571054	0.312050306	profile #7
4930404H11Ril	0.033096171	0.07337642	0.120386532	profile #7
Gm4900	0.026141423	0.066937703	0.157619099	profile #7
Gm35279	0.048989228	0.108612389	0.579140732	profile #7
