# Supplementary material for: Analysis of Time Series Gene Expression and DNA Methylation Reveals the Molecular Features of Myocardial Infarction Progression
Source: Front Cardiovasc Med. 2022 Jun 24;9:912454. doi: 10.3389/fcvm.2022.912454 (PMC9263976; doi:10.3389/fcvm.2022.912454)
Supplement: Supplementary Figure 1 — Bar chart of the number of up-regulated and down-regulated differential genes at different time points. [file Data_Sheet_1.ZIP › Supplementary materials1/Table S2.pdf]

.

	Gene ID	Chromosome	Methylated region	Fold change (MeDIP-seq)	p-value	Fold change (RNA-seq)	p-value
10m	Atcay	chr10	81232001-81232500	1.297	3.17E-03	-1.32	1.12E-05
	Thbs1	chr2	118109501-118110000	-1.282	1.91E-02	1.772	2.50E-02
1h	Cytip	chr2	58161501-58162000	-1.368	1.80E-02	1.439	2.40E-03
	Ppp1r15a	chr7	45528001-45528500	-1.112	3.80E-02	1.394	5.52E-29
6h	1700029J07Rik	chr8	45976501-45977000	1.027	3.40E-02	-1.67	4.47E-05
	Cd247	chr1	165787501-165788000	1.249	4.90E-02	-1.86	4.07E-04
	Chst4	chr8	110040001-110040500	1.061	2.80E-02	-3.651	1.43E-03
	Dgkb	chr12	37879001-37879500	1.369	4.10E-02	-1.01	2.30E-02
	Arc	chr15	74674001-74674500	-2.018	4.99E-03	4.917	2.75E-25
	Bid	chr6	120918001-120918500	-1.163	1.40E-02	1.165	7.73E-03
			120917501-120918000	-1.175	3.10E-03		
	C4b	chr17	34744501-34745000	-1.228	1.30E-02	1.164	5.73E-10
	Ccdc88b	chr19	6859501-6860000	-1.589	3.98E-03	1.031	3.41E-03
	Ccno	chr13	112985501-112986000	-1.681	1.17E-02	3.036	5.31E-07
	Cd24a	chr10	43577501-43578000	-1.055	2.07E-02	2.763	1.49E-12
	Coro2a	chr4	46567501-46568000	-1.318	3.83E-02	1.988	1.70E-05
	Cytip	chr2	58160501-58161000	-1.629	1.09E-02	3.958	2.64E-06
			58161001-58161500	-1.026	4.78E-02		
			58161501-58162000	-1.057	4.73E-02		
	Edn3	chr2	174759501-174760000	-1.484	1.48E-02	1.301	3.47E-04
	Fgr	chr4	132973001-132973500	-1.212	1.94E-02	4.690	3.66E-15
	Fry	chr5	150257501-150258000	-1.326	2.74E-02	1.176	8.80E-29
	Gdf15	chr8	70632501-70633000	-1.265	2.21E-03	3.871	2.23E-27
			70633001-70633500	-1.196	1.73E-02		
	Gdf6	chr4	9842001-9842500	-1.254	3.09E-02	1.348	6.24E-05
	Hspb1	chr5	135887001-135887500	-1.468	2.00E-02	2.317	3.71E-10
	Ier5l	chr2	30475001-30475500	-1.232	2.76E-02	1.450	3.19E-15
			30475501-30476000	-1.152	5.62E-03		
	Ifitm3	chr7	141011001-141011500	-1.467	2.13E-02	1.076	8.27E-14
	Il1r1	chr1	40224001-40224500	-1.192	3.68E-02	1.212	3.23E-08
	Itgal	chr7	127295001-127295500	-1.187	8.89E-03	2.041	8.98E-03
	Itgb2	chr10	77528001-77528500	-1.011	1.01E-02	1.719	3.80E-08
	Lman1l	chr9	57621501-57622000	-1.335	4.98E-02	3.233	6.97E-07
	Napsa	chr7	44571501-44572000	-1.699	7.12E-03	2.918	3.30E-07
	Oacyl	chr18	65696001-65696500	-1.400	7.48E-03	5.742	1.08E-04
	Prss46	chr9	110844001-110844500	-1.464	3.40E-03	4.267	1.71E-02
	Rnf125	chr18	20943501-20944000	-1.296	5.51E-03	1.572	2.17E-05
	Sema6b	chr17	56135001-56135500	-1.351	2.44E-02	1.575	1.31E-16
	Sphk1	chr11	116531001-116531500	-1.257	1.07E-02	5.174	3.65E-73
	Synpo	chr18	60625501-60626000	-1.616	2.98E-04	1.041	5.73E-16
	Tgfbr2	chr9	116177001-116177500	-1.444	7.16E-03	1.093	5.07E-25
	Trpm2	chr10	77971001-77971500	-1.351	1.55E-02	2.945	1.11E-06
24h	Akr1c14	chr13	4058501-4059000	1.307	1.68E-02	-1.181	8.44E-05
	Cd247	chr1	165787501-165788000	1.218	4.92E-02	-1.453	2.65E-03
	Gm5105	chr3	138069001-138069500	1.333	8.20E-03	-1.963	1.94E-02
	Lepr	chr4	101715501-101716000	1.272	9.31E-03	-1.093	7.23E-04
	Mettl24	chr10	40682001-40682500	1.202	2.72E-02	-1.466	4.28E-03
	Atp8b1	chr18	64662501-64663000	-2.397	6.83E-03	1.174	3.27E-08
	Bcat1	chr6	145077501-145078000	-1.329	9.85E-03	2.491	7.44E-09
	Cd48	chr1	171681501-171682000	-1.434	8.82E-03	1.575	7.67E-08
	Cdh1	chr8	106601001-106601500	-1.100	1.69E-02	8.797	3.41E-07
	Cebpe	chr14	54713501-54714000	-1.867	6.50E-03	5.973	4.88E-05
	Cklf	chr8	104249501-104250000	-2.277	1.79E-03	1.269	6.61E-04
	Dynll1	chr5	115302001-115302500	-1.293	3.39E-02	1.075	2.95E-12
	Gas2l3	chr10	89445001-89445500	-1.716	1.13E-03	1.442	2.61E-03
	Gas7	chr11	67532001-67532500	-1.559	1.35E-02	1.353	3.95E-08
	Gdf6	chr4	9842501-9843000	-1.347	1.39E-02	2.290	3.25E-08
	Glt1d1	chr5	127630501-127631000	-1.231	5.87E-03	4.104	4.66E-02
	Gm13889	chr2	93957501-93958000	-1.055	3.29E-02	2.474	5.63E-14
	Ifitm1	chr7	140966501-140967000	-1.013	3.10E-02	1.869	4.76E-03
	Lrmp	chr6	145121501-145122000	-1.090	3.92E-02	1.341	7.61E-07
	Nfam1	chr15	83031501-83032000	-1.377	6.93E-03	2.454	9.95E-10
	Pgs1	chr11	117984501-117985000	-2.138	1.60E-03	1.043	3.12E-06
	Prkcb	chr7	122288501-122289000	-1.661	2.96E-02	1.152	5.23E-04
	Prss46	chr9	110843501-110844000	-1.696	1.12E-02	4.101	1.23E-02
	Psat1	chr19	15927001-15927500	-1.116	4.05E-02	2.866	5.98E-25
	Rad51	chr2	119111001-119111500	-1.721	2.62E-02	1.811	1.10E-03
	S100a4	chr3	90602501-90603000	-1.893	6.33E-03	3.150	2.41E-04
	Sema3f	chr9	107712001-107712500	-1.216	4.03E-02	1.562	7.46E-11
	Serinc2	chr4	130277001-130277500	-1.203	1.05E-02	2.571	7.86E-06
	Susd1	chr4	59439001-59439500	-1.526	3.47E-02	1.109	4.24E-02
	Thbs1	chr2	118109501-118110000	-1.067	3.36E-02	5.574	3.00E-09
	Vill	chr9	119051001-119051500	-1.229	7.12E-03	3.000	3.89E-10
	Zfp954	chr7	7123001-7123500	-2.050	4.99E-02	1.119	2.54E-04
